# Plasma-Based Metabolomics Profiling of High-Risk Human Papillomavirus and their Emerging Roles in the Progression of Cervical Cancer

**DOI:** 10.1155/2022/6207701

**Published:** 2022-11-03

**Authors:** Aozheng Chen, Min Xu, Jing Chen, Tingting Chen, Qin Wang, Runjie Zhang, Jin Qiu

**Affiliations:** ^1^Hongqiao International Institute of Medicine, China; ^2^Obstetrics and Gynecology Department, Tongren Hospital, Shanghai Jiao Tong University School of Medicine, No. 1111, XianXia Road, Shanghai 200336, China

## Abstract

High-risk human papillomavirus (HR-HPV) is the main etiological factor for cervical cancer. Accumulating evidence has suggested the active role of metabolites in the initiation and progression of cancers. This study explored the plasma metabolic profiles of HPV-16 positive (HPV16 (+)), HPV-18 positive (HPV18 (+)), and HPV negative (CTL) individuals using a nontargeted metabolomics approach. C8 ceramide-1-Phosphate (d18 : 1/8 : 0) was found to inhibit cervical cancer cell proliferation and migration in vitro, evidenced by CCK8 experiments, a cell migration test, RT-qPCR, and western blotting. The underlying mechanism demonstrated that C8 inhibited proliferation and migration in cervical cancer cells via the MAPK/JNK1 signaling pathway. These findings may contribute to the clinical treatment of HR-HPV-induced cervical cancer by intervening in its initiation and progression.

## 1. Introduction

Cervical cancer is the fourth most common malignant tumor in women, and it results in over 300000 deaths worldwide each year [1, 2]. Cervical cancer is a multifactorial, complex multistep process related to the activation of protooncogenes or the inactivation of tumor suppressor genes. Almost 99.7% of cervical cancers in women are attributable to human papillomavirus infections [3]. Persistent infection with high-risk human papillomavirus (HR-HPV) is considered as the most vital epidemiologic risk factor for cervical cancer, and HPV types 16 and 18 contribute to over 70% of cervical cancer [4]. Moreover, HR-HPV leads to the dysfunction of basal epithelial cells and the occurrence of cervical cancer [5].

Tumor cells reprogram their metabolism to support cell growth, proliferation, and differentiation, thus, driving cancer progression [6]. Previous studies indicated that metabolites are the final products of various biological processes and maybe the biomarkers reflecting upstream events, such as environmental influences and genetic mutations [7]. Therefore, metabolomic variations are considered ideal biomarkers for the screening and diagnosing cancers [8, 9]. Metabolites are defined as biologically active metabolites by metabolomics [10]. Emerging studies have highlighted the functional role of metabolites in physiology and disease. A previous study indicated that mTOR kinase6 acts as an active entity in cell nutrients and energy [11], and an additional study revealed that *α*-ketoglutarate activates macrophages and regulates immunity [12]. Abnormal accumulation of fumarate and succinate, termed oncometabolite, causes potential transformation to malignancy [13]. Furthermore, metabolites such as lipids, amino acids, and bile acids regulate insulin sensitivity [14]. Moreover, lysophosphatidic acid can mediate ovarian cancer cell migration and metastasis by activating the AMPK pathway [15]. Likewise, HPV infections drive metabolic modifications, so altered metabolites may be potential markers for predicting the risk of cervical cancer [16, 17]. Numerous significant metabolites were elevated in plasma from both cervical dysplasia and cervical cancer in previous reports [16, 18]. However, the changes in plasma bioactive metabolites caused by HPV16/18 infection and their underlying mechanisms in cervical cancer initiation and progression remain largely unknown.

This article aimed to explore the difference in the global metabolite expression profiles between HR-HPV and the healthy control group with liquid chromatography-mass spectrometry (LC-MS). In addition to analyzing the global profiling between HR-HPV and the healthy control group, we aimed to validate further how these identified metabolites intervene in the oncogenic capacity of HR-HPV in vitro.

## 2. Materials and Methods

### 2.1. Clinical Samples

For this study, only individuals with either HPV16 or HPV18 positive and healthy controls were included. Clinical samples were randomly collected from female patients seen from July to September 2020 at the Gynecological Department, Tongren Hospital. This study was approved by the Ethics Committee of Tongren Hospital, Shanghai Jiao Tong University. (No. 2018-049-01) and recruited 20 patients with an HPV16-positive or HPV18-positive diagnosis and 10 healthy controls following a Thinprep Cytology test (TCT). Of the 10 female patients who tested HPV16 positive, 7 were diagnosed with chronic cervicitis, and the 3 had low-grade squamous intraepithelial lesions (LSILs). Of the 10 female patients who tested HPV18 positive, 5 had chronic cervicitis, 1 had LSIL, 2 had a high-grade squamous intraepithelial lesion (HSIL). The 10 healthy controls did not present any precursor lesion. All participants aged between 20 and 45 years old were not pregnant or experiencing menstruation (Table 1). All individuals were thoroughly informed and granted written informed consent prior to blood sampling.

### 2.2. Blood Sample Preparation

Trained gynecologists collected all the blood samples in the anticoagulant tubes following the standard hospital protocol and then centrifuged at 3000 rpm for 15 min at 4°C to collect plasma. Afterward, the supernatant was transferred into 1.5 mL tubes and stored at -80°C as plasma samples for liquid chromatography-mass spectrometry (LC-MS) detection before use. Each frozen plasma sample was thawed at room temperature. Approximately 100 *μ*L of the supernatant sample was added to 10 *μ*L of 2-chloro-L-phenylalanine (0.3 mg/mL) and Lyso PC17:0 (0.01 mg/mL diluted in methanol) as an internal standard and then vortexed for 10 s. Precold methanol and acetonitrile (1/2, v/v) were mixed, added to the sample, and then vortexed for 1 min. Samples were ultrasonically extracted in an ice water bath for 10 min and held at -20°C for 30 min. After centrifuging at 13000 rpm at 4°C for 10 min, 300 *μ*L of the supernatant was transferred into an LC-MS vial and evaporated to dryness. Next, 400 *μ*L of methanol and water (1/4, v/v) were applied to each sample, vortexed for 30 s, held at 4°C for 2 min, and then stored at -20°C. After 30 min, samples were centrifuged at 13000 rpm at 4°C for 10 min. The supernatants were aspirated using syringes, filtered through 0.22 *μ*m microfilters, transferred to LC vials.

### 2.3. Detection of Metabolic Profiling by LC-MS

We performed nontargeted metabolomics with LC-MS in the plasma samples to identify altered metabolites in the HPV16 positive, HPV18 positive, and healthy control groups. Metabolic profiles in both electrospray ionization (ESI)-positive and ESI-negative ion modes were generated using an ACQUITY UPLC I-Class system (Waters Corporation, Milford, USA) coupled with an AB SCIEX Triple TOF 5600 System (AB SCIEX, Framingham, MA, USA). The binary gradient elution systems consisted of water containing 0.1% formic acid (v/v, A) and acetonitrile containing 0.1% formic acid (v/v, B). Separation was achieved using the following conditions: 20% B for 2 min; 60% B for 4 min; 100% B for 11 min; 100% B for 13 min; 13.5 5% B for 13.5 min, and a final 5% B for 14.5 min. All the samples were analyzed at 4°C. The injection volume was 2 *μ*L. Data acquisition was performed in full scan mode (m/z ranges from 70 to 1000) combined with information-dependent acquisition (IDA) mode at collision energies of 10 eV (+) and -10 eV (-). The mass spectrometry parameters were as follows: ion source temperature of 550°C (+) to 550°C (-) throughout the acquisition; ion spray voltage from 5500 V (+) to 4500 V (-); curtain gas at 35 PSI; decluttering potential was set between 100 V (+) and -100 V (-); and interface heater temperature from 550°C (+) to 600°C (-). For the IDA analysis, the range of m/z was set to 25-1000, and the collision energy was 30 eV.

### 2.4. Data Analysis

Metabolites were identified and analyzed using public databases (http://www.hmdb.ca/;http://www.lipidmaps.org/) and self-built databases. The distinct tendency among groups was analyzed using principal component analysis (PCA) and orthogonal partial least-squares discriminant analysis (OPLS-DA) models. Statistical significance was observed at variable importance in projection (VIP) value > 1 and *P* value < 0.05. In addition, qualitative and metabolic pathway analyses of differential metabolites were investigated with the Kyoto Encyclopedia of Genes and Genomes (KEGG) online database. Furthermore, the human metabolome database (HMDB) IDs and KEGG IDs of the metabolites were entered into the ingenuity pathway analysis (IPA) server (IPA, Ingenuity® Systems, http://www.ingenuity.com) to analyze networks between metabolites.

### 2.5. Ceramide-1-Phosphate Purchase

C8 ceramide-1-phosphate (d18 : 1/8 : 0) (#860532P) was purchased from Sigma-Aldrich (St. Louis, MO, USA), and C18 ceramide-1-phosphate (d18 : 1/18 : 0) was synthesized by chemical method. The 50 mM stock solutions of C8 and C18 were prepared in dimethyl sulfoxide (DMSO). These solutions were then diluted to 0, 5, 10, 20, and 30 *μ*M before adding to the cultures, with the DMSO concentration maintained below 0.1%. Next, the same amount of DMSO was added to the controls.

### 2.6. Cell Culture

Two human cervical cancer cell lines HeLa (ATCC CCL-2) and GH354 (ATCC CRL-13003) were purchased from ATCC (Manassas, VA) and cultured in DMEM (Dulbecco's modified Eagle's medium) containing 10% fetal bovine serum (Gibco, New Zealand) and 500 *μ*L penicillin-streptomycin (Gibco, USA) following the manufacturer's instructions. All cells were cultured in a 37°C incubator with 5% CO_2_ under aseptic conditions. The growth of the cells was observed daily and the medium was changed according to the growth of cells. The cells were digested with 0.05% trypsin (ScienCell, USA) for inoculation when fused to 80%.

### 2.7. CCK8 Cell Proliferation Test

CCK8-assay was employed to evaluate the effect of the metabolites on the proliferation of cervical cancer cells. Cultured HeLa and GH354 cells suspended in a 100 *μ*L culture medium with 10% FBS were inoculated in a 96-well plate (3000 cells/well) with 0, 5, 10, 20, and 30 *μ*M C8 and C18. The cells with different concentrations of C8 and C18 were incubated for 24 hrs, 48 hrs, and 72 hrs. Before measuring the absorbance at 450 nm wavelength using a microplate reader, 10 *μ*L CCK8 (TargetMOL, China) solution was added to each well and incubated for 1 hour.

### 2.8. Cell Migration Assay

Different groups of cultured cells were plated in a 6-well plate with 1000 *μ*L of serum-free medium per well along with C8. After C8 intervention, the center of the 6-well plate bottom was cross-marked with a black marker pen. Then, one scratch was generated per well. Starting from the scratch area marking the midpoint, the microscope ruler selected the scratch area within 1.00 mm as the photographic area at an equal distance. The photographs were taken at 0 and 72 hours under an inverted microscope. The scratch area was calculated using Image J image analysis software, and the healing rate = (0 h scratch area − current scratch area)/0 h scratch area × 100%.

### 2.9. RNA Isolation and RT-qPCR Analysis

The cells with or without C8 intervention were used to isolate total RNA using TRIzol reagent (Invitrogen, Carlsbad, CA, USA) following the manufacturer's instructions and reverse transcribed using HiScript®II QRT SuperMix (Vazyme, Nanjing, China) for reverse transcription-quantitative PCR (RT-qPCR) to obtain cDNA chains. The target genes were amplified in three replicates using the SYBR Green PCR Master Mix (Vazyme, Nanjing, China). The primers specific for E-cadherin, N-cadherin, Vimentin, MMP9, and GAPDH were as follows: E-cadherin primers (E-cadherin F: ATTTTTCCCTCGACACCCGAT; R: TCCCAGGCGTAGACCAAGA), N-cadherin primers (N-cadherin F: AGCCAACCTTAACTGAGGAGT; R: GGCAAGTTGATTGGAGGGATG), Vimentin primers (Vimentin F: TGCCGTTGAAGCTGCTAACTA; R: CCAGAGGGAGTGAATCCAGATTA), MMP9 primers (MMP9 F: AGACCTGGGCAGATTCCAAAC; R: CGGCAAGTCTTCCGAGTAGT), and GAPDH primers (GAPDHF: ACAACTTTGGTATCGTGGAAGG; R: GCCATCACGCCACAGTTTC). The RT-qPCR procedures were as follows: 95°C for 5 seconds, followed by 40 cycles at 95°C for 10 s, and 60°C for 30 s. Quantified mRNA was normalized to GAPDH as a control. The relative expression of mRNA was determined by the 2-*ΔΔ*CT method.

### 2.10. Protein Extraction and Western Blot Analysis

A total of 5-10 mg of cells with or without C8 intervention extract were used for western blot analysis. Protein was extracted using protease inhibitors (Thermo Fisher Scientific, Waltham, MA, USA) and centrifuged to remove the cell pellet. The samples were heat-denatured at 95°C for 5 minutes with 5× SDS-PAGE loading buffer and fractionated on 10% SDS-PAGE gels (Bio-Rad, Hercules, CA, USA). GAPDH (HRP-60004, Proteintech, Manchester, UK) was used as a standard control protein. Following electrophoresis, proteins were transferred to a nitrocellulose membrane and blocked with 5% skim milk powder. The membranes were incubated at 4°C overnight with anti-E-cadherin (1 : 4000, 60335-1-lg, Proteintech, Manchester, UK), anti-N-cadherin (1 : 5000, 66219-1-lg, Proteintech, Manchester, UK), anti-MMP9 (1 : 500, 10375-2-AP, Proteintech, Manchester, UK), anti-Vimentin primary antibodies (1 : 2500, 60330-1-lg, Proteintech, Manchester, UK), anti-JNK1 (1 : 1000, 66210-1-lg, Proteintech, Manchester, UK), anti-Phospho-JNK1 (1 : 2000, 80024-1-RR, Proteintech, Manchester, UK), anti-ERK1/2 (1 : 1000, 16443-1-AP, Proteintech, Manchester, UK), anti-Phospho-ERK1/2 (1 : 2000, 28733-1-AP, Proteintech, Manchester, UK), anti-Bax (1 : 5000, 50599-2-lg, Proteintech, Manchester, UK), and anti-Bcl2 (1 : 1000, BF9103, Affinity Biosciences, Changzhou, China). Goat anti-rabbit IgG (1 : 5000, SA00001-2, Proteintech, Manchester, UK) and Goat anti-mouse IgG (1 : 5000, SA00001-2, Proteintech, Manchester, UK) horseradish peroxidase conjugated secondary antibodies were incubated with the membranes at 25°C for 1 h. Densitometric analysis was performed to quantitate western blot results using computerized image software (ImageJ).

### 2.11. Statistical Analysis

The univariate analysis of variance (ANOVA) quantified the differences between the HR-HPV infected and CTL groups with GraphPad Prism 6.0. The result was presented as the mean ± standard error (SE). *P* values were determined by one-way analysis of variance (ANOVA) with Tukey's post hoc correction for multiple group comparisons. All data analyses were processed using GraphPad Prism, version 6.0 (GraphPad Software, San Diego, CA). A two-sided *P* value of <0.05 was considered statistically significant. Significance was indicated as follows: ^∗^*P* < 0.05, ^∗∗^*P* < 0.01, ^∗∗∗^*P* < 0.001, and ^∗∗∗∗^*P* < 0.0001.

## 3. Results

### 3.1. HR-HPV Infection Accompanied by Distinct Metabolome Profiles

**Figure 1:**
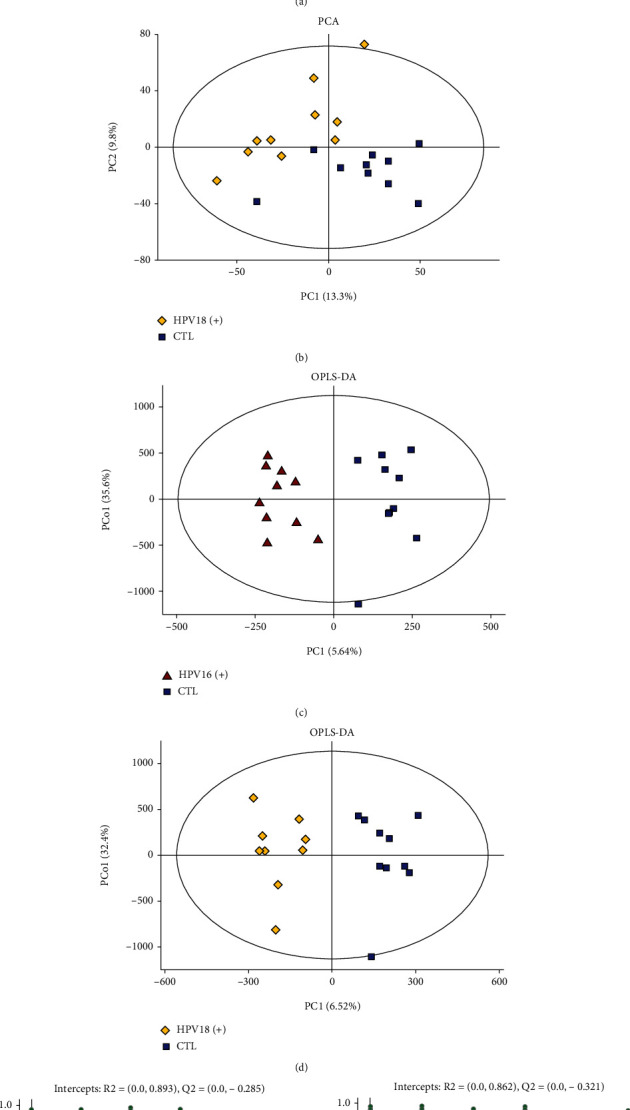
PCA score plots discriminating the metabolic profiles between the CTL and HR-HPV infection groups and permutation test of OPLS-DA models. (a). Principal component analysis (PCA) showed a distinct metabolome profile in the HPV16 (+) groups compared to the CTL group. The *X*-axis and *Y*-axis represent the first and second principal components, respectively. (b). PCA showed a distinct metabolome profile in the HPV18 (+) groups compared to the CTL group. (c, d). Statistical validation with permutation analysis (200 times) of the corresponding OPLS-DA model of HPV16 (+), HPV18 (+), and CTL. (e). Permutation tests were obtained from LC-MS/MS data of HPV16 (+) and CTL (f). Permutation tests were obtained from LC-MS/MS data of HPV18 (+) and CTL. The intercept values of the regression line and the *Y*-axis are R2 and Q2, respectively.

To examine the differences in metabolites in the CTL and HR-HPV infection groups, we conducted a multivariate assessment and OPLS-DA analysis. After the PCA model was established, a separation tendency was observed between the CTL group and the HPV16 (+) (Figure 1(a)) or HPV18 (+) group (Figure 1(b)). OPLS-DA models were obtained with principal predictive components and principal orthogonal components. OPLS-DA removed unassociated data from the dataset and verified the metabolic profile dissolution between groups (Figures 1(c) and 1(d)). We next performed a permutation examination of the OPLS-DA model. The R2 and Q2 intercept values were (0.0, 0.893) and (0.0, -0.285) between the CTL and HPV16 (+) infection groups, respectively (Figure 1(e)), and (0.0, 0.862), (0.0, -0.321) between the CTL and HPV18 (+) infection groups, respectively (Figure 1(f)).

### 3.2. Identification of Differential Metabolites in HR-HPV Infection and CTL Plasma

**Figure 2:**
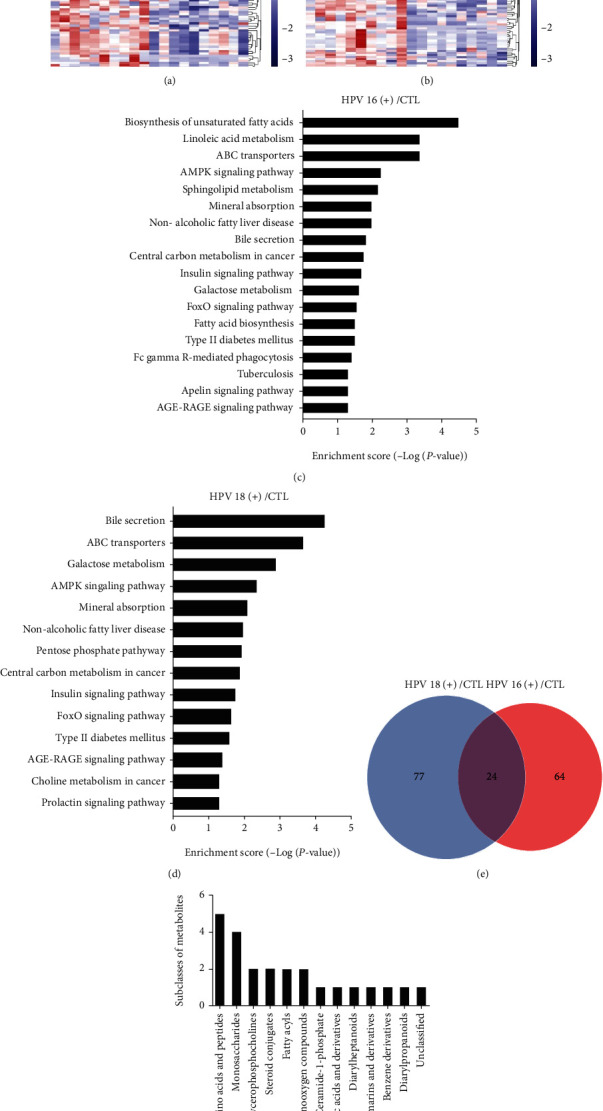
Analysis of HR-HPV infection plasma reveals HPV-related differences in metabolites. (a). Heatmap of differential metabolites with intensities determined by LC-MS. Hierarchical clustering analysis was performed to assess significantly upregulated and downregulated metabolites between HPV16 (+) and CTL plasma. (b). Hierarchical clustering analysis was performed to assess significantly upregulated and downregulated metabolites between HPV18 (+) and CTL plasma. Increased and decreased metabolite levels are depicted by red and blue colors, respectively. VIP, variable importance in projection. (c). Pathway clustering analysis of the HPV16 (+) samples, with significantly enriched pathway clusters identified based on *P* value < 0.05 and depicted on a logarithmic scale (log2). (d). Pathway clustering for metabolites enriched in HPV18 (+) samples by metabolomics analysis. (e). Number of shared metabolites among the high-risk positive groups visualized on a Venn diagram. (f). Number of shared metabolites that belong to amino acids and peptides, monosaccharides, glycerophosphocholines, steroid conjugates, fatty acid moieties, organooxygen compounds, ceramide-1-phosphate, carboxylic acids and derivatives, diarylheptanoids, coumarins and derivatives, benzene derivatives, diarylpropanoids, etc.

Overall, 7696 differentially expressed metabolites were detected by LC-MS, and the details of all the metabolites are provided in Supplementary Table 1. Among these differential metabolites, a total of 88 significantly differential metabolites with VIP > 1 and *P* < 0.05 were identified in the HPV16 (+) group, 31 of them were significantly upregulated, and 57 metabolites were significantly downregulated (Figure 2(a)). On the other hand, 101 significant differentially expressed metabolites were identified in the HPV18 (+) group, 26 of them were significantly upregulated and 75 metabolites were significantly downregulated (Figure 2(b)). Further pathway analysis (*P* < 0.05) revealed that most enriched metabolic pathways were in the high-risk groups (Figures 2(c) and 2(d)), and this indicated that HPV16 and HPV18 might have similar metabolic functions in the initiation and progression of cervical cancer. To evaluate similarities and differences in the datasets, we compared the number of shared and unique significant differentially expressed metabolites among the three groups by a Venn diagram. The Venn diagram illustrated 24 significant differential metabolites shared by HPV16 and HPV18 groups (Figure 2(e)), and all these 24 significant differential metabolites (*P* < 0.05) were presented in Table 2. They were roughly categorized into twelve distinct classes: amino acids and peptides, monosaccharides, glycerophosphocholines, steroid conjugates, fatty acid moieties, organooxygen compounds, ceramide-1-phosphate, carboxylic acids and derivatives, diarylheptanoids, coumarins and derivatives, benzene derivatives, and diarylpropanoids (Figure 2(f)). Among these classes, ceramide-1-phosphate draw our attention, as there has been an increasing body of evidence indicating ceramide-1-phosphate regulates cell proliferation, apoptosis, migration, and other life processes as well as invasion, metastasis, and clinical outcome of pancreatic cancer, lung cancer, and breast cancer [19–23]. Hence, we selected C18 Ceramide-1-Phosphate (d18 : 1/18 : 0) and C8 Ceramide-1-Phosphate (d18:1/8 : 0) to verify their biological functions. These two ceramide-1-phosphates expressed differentially in both HPV16 and HPV18 groups and were detected by LC-MS (Table 3).

### 3.3. C8 Inhibits the Proliferation and Migration of CC Cell Lines and Malignant Behavior-Related Molecules at the mRNA and Protein Levels

**Figure 3:**
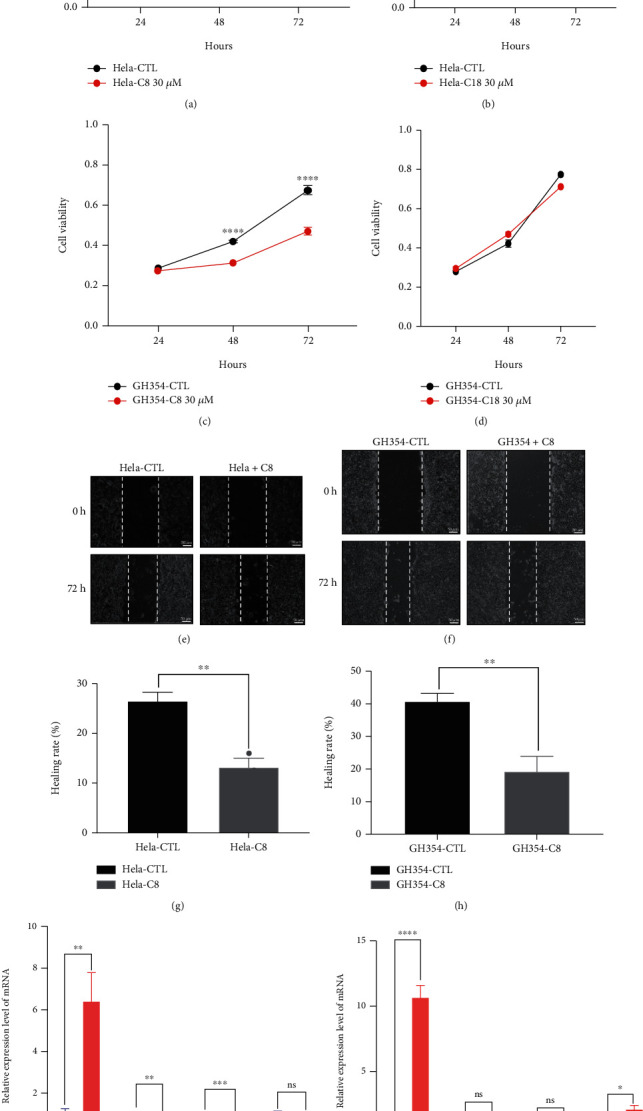
C8 altered the proliferation and migration of CC cell lines and malignant behavior related molecules at the mRNA levels. (a, c). C8 (concentration of 30 *μ*M) inhibited the proliferation of HeLa and GH354 cells. (b, d). C18 (concentration of 30 *μ*M) had no effect on the proliferation of HeLa and GH354 cells. (e–h). C8 inhibited cervical cancer cell migration and healing rate compared to the control group. Images are representative of 3 biological replicates. All values represent the mean ± standard deviation (SD). Scale bar in (e, g) is equal to 50 *μ*m(micrometer). (i, j). RT-qPCR analysis of the mRNA expression levels of E-cadherin, N-cadherin, Vimentin, and MMP9 under C8 intervention in HeLa and GH354 cells. Images are representative of 3 biological replicates. All values represent the mean ± standard deviation (SD). ^∗^*P* < 0.05; ^∗∗^*P* < 0.01; ^∗∗∗^*P* < 0.001.

**Figure 4:**
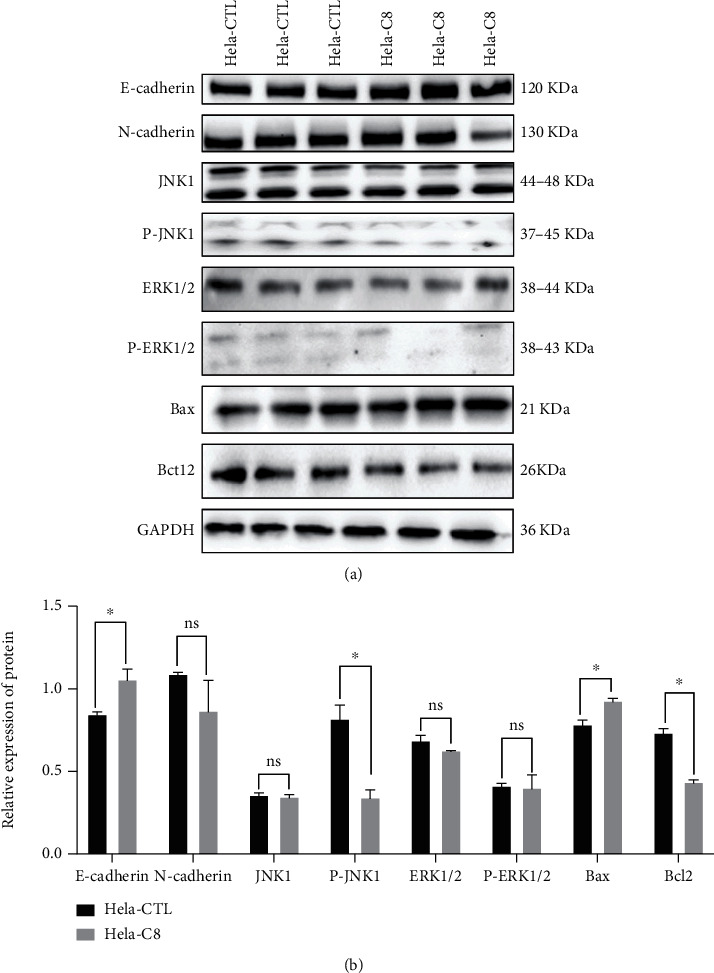
C8 altered malignant behavior related molecules and MAPK signaling in cervical cancer cell. (a). Western blotting analysis of E-cadherin, N-cadherin, JNK1, P-JNK1, ERK1/2, P-ERK1/2, Bax, and Blc2 protein levels in HeLa after treated C8 (concentration of 30 *μ*M). (b). Densitometric analysis of western blots, ^∗^*P* < 0.05; ^∗∗^*P* < 0.01; ^∗∗∗^*P* < 0.001, *n* = 3.

We conducted the assays to examine the proliferation and migration potency of C8 and C18 in HeLa and GH354 cervical cancer cell lines, C8 presented significant biological function at the concentration of 30 *μ*M. The proliferation rate of HeLa and GH354 cells treated with C8 of 30 *μ*M decreased, while C18 had no effect, based on CCK8 assay results (Figures 3(a)–3(d)). Comparing the migration index and healing rate of C8 (concentration of 30 *μ*M) treated cell lines and the control group at 0 and 72 hours revealed that the migration index and healing rate significantly diminished in the C8 group (Figures 3(e)–3(h)). To verify the observed effects of C8, we analyzed the expression levels of several molecules closely related to malignant behavior, especially epithelial-to-mesenchymal transition (EMT), by western blotting and RT-qPCR experiments. The RT-qPCR analysis revealed the changes in E-cadherin, N-cadherin, Vimentin, and MMP9 expression after C8 (concentration of 30 *μ*M) intervention in HeLa and GH354 cells. Specifically, E-cadherin was upregulated after C8 intervention in both HeLa and GH354 cells, while N-cadherin and Vimentin were downregulated after C8 intervention in HeLa cells (Figures 3(i) and 3(j). Western blot and densitometric analysis verified the changes in EMT again at the protein level.  Figure 4 presented significantly upregulated E-cadherin and downregulated N-cadherin after C8 intervention in HeLa and GH354 cells.

### 3.4. Interaction Network and Downstream Effects of Differential Metabolites

**Figure 5:**
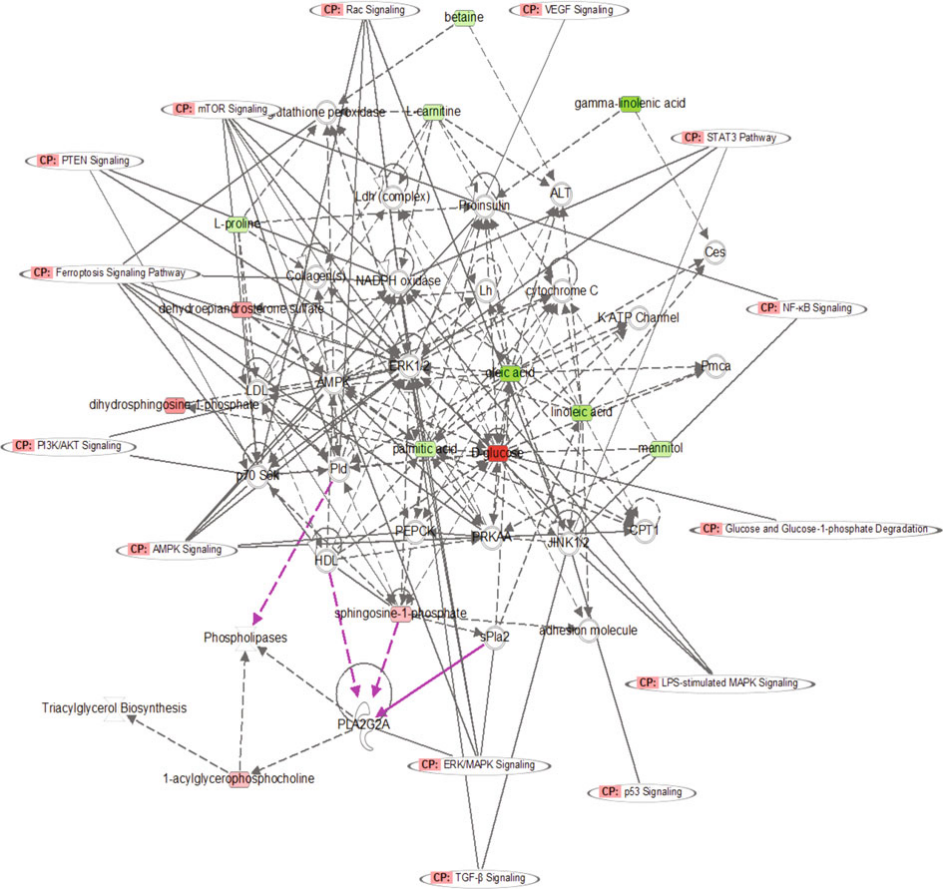
IPA analysis of metabolites related to biological networks and canonical pathways and function. The differential metabolites were closely associated with PI3K/AKT signaling, mTOR signaling, and PTEN signaling, and specific lipids were associated with ERK/MAPK signaling, TGF-*β* signaling, and PLA2G2A regulation. Lines represent the biological relationship between two nodes. Red symbols represent upregulated metabolites; green symbols represent downregulated metabolites. Purple lines point out PLA2G2A suggesting the significance of its associated signaling.

Differential metabolites were imported into IPA software for further biological pathway prediction to reveal potential targets and mechanisms. The results indicated that these differential metabolites had a close association with PI3K/AKT signaling, mTOR signaling, PTEN signaling, and specific lipids were associated with MAPK signaling, TGF-*β* signaling, and PLA2G2A regulation (Figure 5). Thus, whether C8 intervention modified the expression of molecules in this pathway was then explored by western blot. After C8 intervention, JNK1 remained at the same expression level, but P-JNK1 decreased. In addition, the expression level of Bax was upregulated, while Bcl2 was downregulated after C8 intervention. These results have suggested that C8 may exert biological functions by inhibiting the MAPK signaling pathway (Figures 4).

## 4. Discussion

HR-HPV is a potent human carcinogen, and persistent HR-HPV infection is a necessary risk factor for the cervix and cervical cancer [24]. It induces epithelial cell malignant transformation and suppresses the immune response by encoding oncoproteins [25]. For instance, the E6 and E7 proteins encoded by HR-HPV could promote cervical cancer [26, 27], the E6 protein encourages the growth of cervical cancer cells by targeting the P53 protein [28], and the E7 protein immortalizes human epithelial cells by targeting the pRb protein [29]. Rapid proliferation is a driving force for the massive energy required by malignant cells to adapt to metabolic modifications [30]. Driven by oncoproteins, metabolites can be used to characterize the molecular mechanisms of HR-HPV comprehensively [30]. In addition, the abnormal expression or activation of metabolic pathway-related enzymes is tightly associated with the occurrence of cancers [31]. Previous studies revealed that E6/E7 could also regulate the glycolytic pathway via elevated expression of hexokinase-II and act as a promotor in HPV-associated cervical lesions in serum [32].

With the rapid development of metabonomics technology, the function of metabolites has been identified [33]. Metabolites, as indicators are valuable for identifying biomarkers of cervical cancer. Studies have reported that in the study of metabonomics about cervical cancer, samples were blood (serum or plasma), vaginal secretions, tissues, and urine [34]. A study reported that combination of lysophosphatidylcholine (17 : 0), n-oleoyl threonine, bilirubin, tetracosahexaenoic acid (lysoPC(17 : 0)), and 12-hydroxydodecanoic acidare are satisfactory candidate biomarkers for cervical cancer diagnosis from cervical cancer plasma samples [35]. In a lipidomics study profiling of plasma, Nam et al. showed that compared to healthy controls and patients with CIN1, phosphatidylcholine, phosphatidylethanolamine, diglyceride, and free FAs are major lipid classes with significant differences in patients with CIN2/3 and cervical cancer [17]. The researchers retrieved plasma samples for nontargeted metabolome analysis and screened AMP, aspartate, glutamate, hypoxanthine, lactate, proline, and pyroglutamate to construct a linear prediction model combined with positive HPV status were correlated with substantial risk of cervical cancer [18]. The metabolic modulations by HPV infection is inextricably linked to cervical cancer progression [36, 37]. However, the metabolic profile in response to HPV16/18 infection has not yet been elucidated.

To further explore the potential biological functions of these metabolites with HR-HPV infection and its carcinogenic effects, nontargeted metabolomics analysis was executed to identify differential metabolic signatures between the HPV16 (+), HPV18 (+), and CTL groups. Among the differential metabolites shared by the HR-HPV infection and the control groups, the ceramide-1-phosphate extensively discussed in the cancer treatment aroused our attention. The ceramide-1-phosphate belongs to the ceramide phosphate (CerPs), they are members of the sphingolipids class and a component of eukaryotic cell membranes. They act as a bioactive lipid in apoptosis, inflammation, cell cycle arrest, and the heat shock response [14]. CerPs are transformed from ceramides by a specific ceramide kinase (CerK), and they can be dephosphorylated by phosphatidate phosphatase back to the ceramide. These CerPs are able to regulate cell proliferation, apoptosis, and migration [19, 20]. In addition, an increasing number of papers elucidated the clinical potential of the CerPs in cancer treatment. Ceramide synthase 2-C-ceramide axis was reported to limit the metastatic potential of ovarian cancer cells [38]. Ceramide-1-phosphate promoted stem cell mobility and enhanced cell migration and invasion of pancreatic cancer [21, 39]. Also, the CerPs in breast cancer patients were correlated with invasion and metastasis [23]. Moreover, the biological functions of Ceramide-1-phosphate vary depending on the side chain length and isoforms. Research shows that short-chain C2-ceramide-1-phosphate-or C8-ceramide-1-phosphate induces Ca^2+^ mobilization in CAPE cells, thyroid FRTL-5, or Jurkat T cells, but not in fibroblasts or neutrophils [40]. Long-chain C16-ceramide-1-phosphate fails to alter Ca^2+^ concentration in A549 cells [41]. Ceramide-1-phosphate with an acyl-chain of 6, 16, and 18 : 1 carbons efficiently activated cPLA2 in vitro, whereas C2-ceramide-1-phosphate failed to do so [42]. High-risk HPV E6 and E7 proteins can enter the host cell nucleus and are the key factors to maintain the malignant phenotype of HPV-positive cancer cells. HPV E6 and E7 proteins promote the migration, invasion, and epithelial-mesenchymal transition (EMT) abilities of cervical cancer cells through a series of action modes [43]. Therefore, we detected proliferation, migration, and EMT-related markers E-cadherin and N-cadherin by QPCR and WB. Our study indicated that CerPs were also implicated in cervical cancer progression, and C8 ceramide-1-phosphate (d18:1/8 : 0) was found to inhibit cell proliferation, migration, and malignant behaviour of cervical cancer in vitro.

Metabolites serve as controllers of biological processes and phenotypes [10], and tumorigenesis may change the overall metabolism of the human body. Metabolomics can help capture altered biological processes, such as amino acids and lipid metabolites [44]. The bioactive metabolomics drives phenotype modulation by participating in life activity and exerting biological activities via competitive inhibition, posttranslational modifications, and signal transduction [45]. Oncometabolite accumulation is a causal process in malignant conversion that contributes to propagating cancer. To directly examine the possible mechanism underlying the observed phenotypic changes, we analyzed the expression levels of molecules in the signaling pathway. IPA analysis in this study revealed that differential metabolites in HR-HPV infection and CTL plasma were enriched in PI3K/AKT signaling, mTOR signaling, PTEN signaling, MAPK signaling, TGF-*β* signaling, and PLA2G2A regulation. Following HPV infection, the host cells of HPV infection are immortalized and transformed which will alter expression of multiple genes and activate several signalling pathways, especially the PI3K/Akt/mTOR signalling pathway [46]. Moreover, In HPV-positive cells, phosphorylation of p38 and MAPK AP kinase2 (MK2) proteins was induced along with relocalization to the cytoplasm confirming the p38/MK2 pathway as a key regulator of the HPV life cycle [47]. In addition, it has also been reported that HPV16 E6 and HPV18 E6 oncoproteins activate MAPK signalling pathway to promote cell proliferation by upregulating p-PI3K [48]. Our further western blot results indicated that the C8 intervention inhibited proliferation and migration in cervical cancer cells via the MAPK/JNK1 signaling pathway. This ceramide-1-phosphate identified by our study is closely associated with tumorigenesis and metabolic phenotype changes in the progression of CC.

Notably, the nontarget metabolomics in our study also indicated a significant increase in LysoPC and PC, members of the fatty acid moieties class, in the HPV16 (+) and HPV18 (+) groups. This result suggested LysoPC and PC may also correlate with HR-HPV-induced oncogenesis, consistent with a previous study concluding PC and LysoPC were potential biomarkers for cervical cancer [49]. LysoPC originates from the cleavage of PC, the main component of oxidatively damaged low-density lipoprotein, was reported to aggregate inflammation and associated with the invasion, metastasis, and prognosis of tumors [24]. In addition, emerging evidence has indicated that enzymes participating in key lipid metabolism are potential therapeutic targets because they either inhibit lipid synthesis or stimulate their degradation [10]. For example, lactate dehydrogenase A was proven to be a key enzyme for lactic acid synthesis. It could also promote apoptosis leading to a decrease in the cell cycle when inhibited [50]. Furthermore, phosphoglycerate dehydrogenase, the enzyme of serine biosynthesis, has potential therapeutic value in lung adenocarcinoma [13]. The exciting consistency in the findings between our study and previous reports is worthy of more thorough research.

In conclusion, HR-HPV infection causes changes in metabolite profiles in humans, and these metabolites may contribute to the process by which HPV16/18 infection induces cervical cancer. This paper is the first to identify C8 as an important lipid metabolite that modulates cervical cancer cell function. In addition, we also identified that other lipids, such as LysoPC and PC, may also involve cervical cancer progression, requiring further investigation. These results refresh our understanding of how bioactive metabolites modify the oncogenic potential of HR-HPV, and provide new insights into the mechanism of the oncogenic process of HPV16/18 infection.

## Figures and Tables

**Table 1:** Characteristics of all the participants.

Characteristics	Group		
	HPV16 positive females	HPV18 postive females	Normal females
Average age ± SD (year)	32.6 ± 7.1	35.7 ± 15.4	40.8 ± 12.4
Chronic cervicitis	7	5	0
Low-grade squamous intraepithelial lesions	3	1	0
High-grade squamous intraepithelial lesions	0	2	0
Normal	0	2	10

**Table 2:** The significant dysregulated metabolites detected by LC-MS in HR-HPV infection groups (shared by HPV16 and HPV18). (*P* < 0.05).

Metabolites	Compound ID	Class	HPV16(+) to HPV(-)		HPV18(+) to HPV(-)	
			Fold change	*Pvalue*	Fold change	*Pvalue*
**Upregulated**						
(4S, 5S)-4,5-dihydroxy-2,6-dioxohexanoic acid	LMFA01050449	Fatty acyls	5.84	0.018	5.96	0.013
Monomethyl phenylphosphonate	87986	Benzene and substituted derivatives	4.78	0.012	4.82	0.011
Glucoheptonic acid	3365	Unclassified	4.28	0.010	4.30	0.007
2,3,4,5,6,7-Hexahydroxyheptanoic acid	HMDB0240292	Organooxygen compounds	3.43	0.007	3.95	0.008
D-glucose	HMDB0000122	Organooxygen compounds	3.40	0.011	3.30	0.009
3alpha-androstanediol glucuronide	LMST05010004	Sterol lipids	2.37	0.014	2.17	0.001
{3-[3-(3,4-dihydroxy-2-methoxyphenyl)prop-2-enoyl]phenyl}oxidanesulfonic acid	HMDB0126072	Linear 1,3-diarylpropanoids	1.83	0.016	2.08	0.013
Dehydroepiandrosterone sulfate	HMDB0001032	Steroids and steroid derivatives	1.78	0.028	1.68	0.004
3,4,5-trihydroxy-6-{[(6E)-3-oxo-1,7-diphenylhepta-4,6-dien-1-yl]oxy}oxane-2-carboxylic acid	HMDB0133920	Diarylheptanoids	1.65	0.038	1.59	0.006
LysoPC(P-16 : 0)	HMDB0010407	Glycerophospholipids	1.35	0.012	1.24	0.023
PC(O-10 : 1(9E)/0 : 0)	LMGP01060027	Glycerophospholipids	1.31	0.009	1.35	0.011
S-(9-deoxy-delta9,12-PGD2)-glutathione	HMDB0013058	Carboxylic acids and derivatives	1.18	0.041	1.24	0.005
**Downregulated**						
CerP(d18:1/18 : 0)	HMDB0010701	Sphingolipids	0.93	0.046	0.90	0.019
Aspartyl-threonine	HMDB0028763	Carboxylic acids and derivatives	0.90	0.007	0.85	0.002
Aspartyl-hydroxyproline	HMDB0028754	Carboxylic acids and derivatives	0.89	0.046	0.86	0.003
5-(4-hydroxyphenyl)pentanoic acid	HMDB0132270	Fatty acyls	0.84	0.003	0.71	≤0.001
Threoninyl-glycine	HMDB0029061	Carboxylic acids and derivatives	0.82	≤0.001	0.73	≤0.001
L-proline	HMDB0000162	Carboxylic acids and derivatives	0.81	0.014	0.82	0.041
D-mannitol	142	Carboxylic acids and derivatives	0.80	0.001	0.78	0.002
5-Hydroxylysine	HMDB0000450	Carboxylic acids and derivatives	0.76	0.007	0.69	0.001
Xylobiose	HMDB0029894	Carbohydrates and carbohydrate conjugates	0.74	0.009	0.69	0.002
Melibiitol	HMDB0006791	Fatty acyls	0.72	0.009	0.75	0.013
3,4,5-trihydroxy-6-{[8-(2-hydroxypropan-2-yl)-2-oxo-4-propyl-2H,8H,9H-furo[2,3-h]chromen-5-yl]oxy}oxane-2-carboxylic acid	HMDB0130128	Coumarins and derivatives	0.72	≤0.001	0.68	≤0.001
3,4,5-trihydroxy-6-{[4-methoxy-6-(3-oxoprop-1-en-1-yl)-2H-1,3-benzodioxol-5-yl]oxy}oxane-2-carboxylic acid	HMDB0128691	Organooxygen compounds	0.69	0.004	0.70	0.001

**Table 3:** The dysregulated sphingolipids detected by LC-MS in HR-HPV infection groups (shared by HPV16 and HPV18).

Metabolites	Subclass	HPV16(+) to HPV (-)		HPV18(+) to HPV (-)	
		Fold change	*P* value	Fold change	*P* value
SM(d18:1/16 : 0)	Phosphosphingolipids	1.008647363	0.960836567	1.148556083	0.4425021
PE-Cer(d14:1(4E)/21 : 0)	Phosphosphingolipids	0.994218985	0.980618739	0.971725372	0.917332112
PE-Cer(d15:1(4E)/20 : 0(2OH))	Phosphosphingolipids	1.026518539	0.825051177	0.763284427	0.030874248
PE-Cer(d14:2(4E,6E)/23 : 0)	Phosphosphingolipids	1.235600195	0.220069266	1.32418265	0.126840563
LysoSM(d18 : 0)	Phosphosphingolipids	1.15600553	0.38154612	1.063718638	0.664015868
CerP(d18:1/8 : 0)	Ceramides	0.989124633	0.298084598	0.97232671	0.582066007
CerP(d18:1/18 : 0)	Phosphosphingolipids	0.929021772	0.046445282	0.900844737	0.019284627
PE-Cer(d14:1(4E)/22 : 0)	Phosphosphingolipids	1.106493168	0.548411596	0.972063433	0.904319534
PE-Cer(d15:1(4E)/20 : 0)	Phosphosphingolipids	1.024621854	0.891435105	0.860938383	0.541710157
Sphingosine 1-phosphate	Phosphosphingolipids	1.278071996	0.025880223	1.2270139	0.077561076
Fumonisin C4	Sphingoid bases	0.961671783	0.698445871	0.870575355	0.149158739
Sphinganine 1-phosphate	Phosphosphingolipids	1.864757476	0.000578872	1.613256039	0.03946171
GlcCer(d15:1/18 : 0)	Neutral glycosphingolipids	1.013763546	0.910410253	1.042723407	0.804255575
SM(d18 : 0/16 : 0)	Phosphosphingolipids	0.994611391	0.965697302	1.01082142	0.946834371
Araliacerebroside	Glycosphingolipids	0.772097545	0.10844525	0.758894277	0.128499741
Scyphostatin A	Sphingoid bases	1.253647394	0.241779922	1.453491412	0.080949335
PI-Cer(t18 : 0/18:0(2OH))	Phosphosphingolipids	0.997887814	0.983753132	1.073429615	0.479910841
GlcCer(t18:1(8Z)/20 : 0(2OH[S]))	Neutral glycosphingolipids	1.027065099	0.728185535	0.872833743	0.219830931
GlcCer(t18:1(8Z)/16 : 0(2OH[S]))	Neutral glycosphingolipids	1.025729401	0.879659091	0.89589231	0.646495931
SM(d18:2/18:1)	Phosphosphingolipids	1.078895147	0.438313999	1.013775708	0.903558948
GlcCer(d18:0/26 : 0)	Neutral glycosphingolipids	0.861561787	0.057511869	0.814073191	0.00864914
Leucettamol A	Sphingoid bases	1.216677861	0.060461464	1.395757442	0.000331627
PE-Cer(d14:1(4E)/24 : 0(2OH))	Phosphosphingolipids	1.221487438	0.31415695	1.144323565	0.603339326
SM(d18:1/18:1(11Z))	Phosphosphingolipids	1.202363713	0.328745941	1.308235935	0.177725258

## Data Availability

The datasets generated and analysed during the current study are available from the corresponding author on reasonable request.

## Supplementary Materials

Supplementary Materials All the dysregulated metabolites detected by LC-MS in HR-HPV infection groups were shown in Supplementary Table 1.

## References

[1] Bray F., Ferlay J., Soerjomataram I., Siegel R., Torre L., Jemal A. (2018). Global cancer statistics 2018: GLOBOCAN estimates of incidence and mortality worldwide for 36 cancers in 185 countries. *CA: a cancer journal for clinicians*.

[2] Cohen P., Jhingran A., Oaknin A., Denny L. (2019). Cervical cancer. *Lancet (London, England)*.

[3] Walboomers J., Jacobs M., Manos M. (1999). Human papillomavirus is a necessary cause of invasive cervical cancer worldwide. *The Journal of pathology*.

[4] Wang R., Pan W., Jin L. (2020). Human papillomavirus vaccine against cervical cancer: opportunity and challenge. *Cancer letters*.

[5] Kuehn B. (2021). WHO launches global push to eliminate cervical cancer. *Journal of the American Medical Association*.

[6] Sun C., Li T., Song X. (2019). Spatially resolved metabolomics to discover tumor-associated metabolic alterations. *Proceedings of the National Academy of Sciences of the United States of America*.

[7] Nicholson J., Lindon J. (2008). Metabonomics. *Nature*.

[8] Cheung P., Ma M., Tse H. (2019). The applications of metabolomics in the molecular diagnostics of cancer. *Expert review of molecular diagnostics*.

[9] Zhou H., Li Q., Wang T. (2019). Prognostic biomarkers of cervical squamous cell carcinoma identified via plasma metabolomics. *Medicine*.

[10] Rinschen M., Ivanisevic J., Giera M., Siuzdak G. (2019). Identification of bioactive metabolites using activity metabolomics. *Nature reviews Molecular cell biology*.

[11] Zoncu R., Efeyan A., Sabatini D. (2011). mTOR: from growth signal integration to cancer, diabetes and ageing. *Nature reviews Molecular cell biology*.

[12] Liu P., Wang H., Li X. (2017). *α*-ketoglutarate orchestrates macrophage activation through metabolic and epigenetic reprogramming. *Nature Immunology*.

[13] Yang M., Soga T., Pollard P. (2013). Oncometabolites: linking altered metabolism with cancer. *The Journal of clinical investigation*.

[14] Yang Q., Vijayakumar A., Kahn B. (2018). Metabolites as regulators of insulin sensitivity and metabolism. *Nature reviews Molecular cell biology*.

[15] Kim E., Park J., Lim S. (2011). Activation of AMP-activated protein kinase is essential for lysophosphatidic acid-induced cell migration in ovarian cancer cells. *The Journal of biological chemistry*.

[16] Khan I., Nam M., Kwon M. (2019). LC/MS-based polar metabolite profiling identified unique biomarker signatures for cervical cancer and cervical intraepithelial neoplasia using global and targeted metabolomics. *Cancers*.

[17] Nam M., Seo S.-S., Jung S. (2021). Comparable plasma lipid changes in patients with high-grade cervical intraepithelial neoplasia and patients with cervical cancer. *Journal of Proteome Research*.

[18] Yang K., Xia B., Wang W. (2017). A comprehensive analysis of metabolomics and transcriptomics in cervical cancer. *Scientific Reports*.

[19] Gómez-Muñoz A. (2004). Ceramide-1-phosphate: a novel regulator of cell activation. *FEBS letters*.

[20] Presa N., Gomez-Larrauri A., Rivera I.-G., Ordoñez M., Trueba M., Gomez-Muñoz A. (2016). Regulation of cell migration and inflammation by ceramide 1-phosphate. *Biochimica et Biophysica Acta (BBA)-molecular and cell biology of lipids*.

[21] Rivera I.-G., Ordoñez M., Presa N. (2016). Ceramide 1-phosphate regulates cell migration and invasion of human pancreatic cancer cells. *Biochemical Pharmacology*.

[22] Gomez-Larrauri A., Ouro A., Trueba M., Gomez-Muñoz A. (2021). Regulation of cell growth, survival and migration by ceramide 1-phosphate - implications in lung cancer progression and inflammation. *Cellular Signalling*.

[23] Schwalm S., Erhardt M., Römer I., Pfeilschifter J., Zangemeister-Wittke U., Huwiler A. (2020). Ceramide kinase is upregulated in metastatic breast cancer cells and contributes to migration and invasion by activation of PI 3-kinase and Akt. *International journal of molecular sciences*.

[24] Qiu F., Su B., Li Z. (2019). New serum biomarker identification and analysis by mass spectrometry in cervical precancerous lesion and acute cervicitis in South China. *Cancer management and research*.

[25] Abudula A., Rouzi N., Xu L., Yang Y., Hasimu A. (2020). Tissue-based metabolomics reveals potential biomarkers for cervical carcinoma and HPV infection. *Bosnian journal of basic medical sciences*.

[26] Crosbie E., Einstein M., Franceschi S., Kitchener H. (2013). Human papillomavirus and cervical cancer. *Lancet (London, England)*.

[27] Ford M., Cannady K., Nahhas G. (2020). Assessing an intervention to increase knowledge related to cervical cancer and the HPV vaccine. *Advances in cancer research*.

[28] Xu L., Selk A., Garland S. (2019). Prophylactic vaccination against human papillomaviruses to prevent vulval and vaginal cancer and their precursors. *Expert review of vaccines*.

[29] Ajiro M., Zheng Z. (2015). E6^E7, a novel splice isoform protein of human papillomavirus 16, stabilizes viral E6 and E7 oncoproteins via HSP90 and GRP78. *MBio*.

[30] Müller-Schiffmann A., Beckmann J., Steger G. (2006). The E6 protein of the cutaneous human papillomavirus type 8 can stimulate the viral early and late promoters by distinct mechanisms. *Journal of virology*.

[31] Zhang B., Chen W., Roman A. (2006). The E7 proteins of low- and high-risk human papillomaviruses share the ability to target the pRB family member p130 for degradation. *Proceedings of the National Academy of Sciences of the United States of America*.

[32] Nilsson R., Jain M., Madhusudhan N. (2014). Metabolic enzyme expression highlights a key role for MTHFD2 and the mitochondrial folate pathway in cancer. *Nature Communications*.

[33] Wishart D. S. (2019). Metabolomics for investigating physiological and pathophysiological processes. *Physiological Reviews*.

[34] Li B., Sui L. (2021). Metabolic reprogramming in cervical cancer and metabolomics perspectives. *Nutrition & Metabolism*.

[35] Wishart D. S. (2016). Emerging applications of metabolomics in drug discovery and precision medicine. *Nature reviews Drug discovery*.

[36] Borgogna J., Shardell M., Santori E. (2020). The vaginal metabolome and microbiota of cervical HPV-positive and HPV- negative women: a cross-sectional analysis. *BJOG: An International Journal of Obstetrics & Gynaecology*.

[37] Kindt N., Descamps G., Lechien J. R. (2019). Involvement of HPV infection in the release of macrophage migration inhibitory factor in head and neck squamous cell carcinoma. *Journal of Clinical Medicine*.

[38] Zhang X., Sakamoto W., Canals D. (2021). Ceramide synthase 2‐C24:1‐ceramide axis limits the metastatic potential of ovarian cancer cells. *FASEB journal : official publication of the Federation of American Societies for Experimental Biology*.

[39] Kuc N., Doermann A., Shirey C. (2018). Pancreatic ductal adenocarcinoma cell secreted extracellular vesicles containing ceramide-1-phosphate promote pancreatic cancer stem cell motility. *Biochemical Pharmacology*.

[40] Gómez-Muñoz A., Gangoiti P., Granado M. H., Arana L., Ouro A. (2010). Ceramide-1-phosphate in cell survival and inflammatory signaling. *Sphingolipids as Signaling and Regulatory Molecules*.

[41] Pettus B. J., Bielawska A., Subramanian P. (2004). Ceramide 1-phosphate is a direct activator of cytosolic phospholipase A_2_. *Journal of Biological Chemistry*.

[42] Wijesinghe D. S., Subramanian P., Lamour N. F. (2009). Chain length specificity for activation of cPLA_2_*α* by C1P: use of the dodecane delivery system to determine lipid-specific effects. *Journal of lipid research*.

[43] Lai S. Y., Guan H. M., Liu J. (2020). Long noncoding RNA SNHG12 modulated by human papillomavirus 16 E6/E7 promotes cervical cancer progression via ERK/slug pathway. *Journal of cellular physiology*.

[44] Liu P., Zhu W., Chen C. (2020). The mechanisms of lysophosphatidylcholine in the development of diseases. *Life sciences*.

[45] Piazza I., Kochanowski K., Cappelletti V. (2018). A Map of Protein-Metabolite Interactions Reveals Principles of Chemical Communication. *Cell*.

[46] Zhang S., Wu X., Jiang T. (2009). The up-regulation of KCC1 gene expression in cervical cancer cells by IGF-II through the ERK1/2MAPK and PI3K/AKT pathways and its significance. *European journal of gynaecological oncology*.

[47] Satsuka A., Mehta K., Laimins L. (2015). p38MAPK and MK2 pathways are important for the differentiation-dependent human papillomavirus life cycle. *Journal of virology*.

[48] Chakrabarti O., Veeraraghavalu K., Tergaonkar V. (2004). Human papillomavirus type 16 E6 amino acid 83 variants enhance E6-mediated MAPK signaling and differentially regulate tumorigenesis by notch signaling and oncogenic Ras. *Journal of virology*.

[49] Zeng Q., Chen J., Li Y. (2017). LKB1 inhibits HPV-associated cancer progression by targeting cellular metabolism. *Oncogene*.

[50] Thompson C. (2009). Metabolic enzymes as oncogenes or tumor suppressors. *The New England journal of medicine*.

[51] Wang Q., Xu M., Chen T., Chen J., Zhang R., Qiu J. (2021). Plasma-Based Endogenous Metabolomics Profiling of High-Risk Human Papillomavirus and Their Emerging Roles in the Progression of Cervical Cancer.

