# Supplementary material for: Plasma-Based Metabolomics Profiling of High-Risk Human Papillomavirus and their Emerging Roles in the Progression of Cervical Cancer
Source: Biomed Res Int. 2022 Nov 3;2022:6207701. doi: 10.1155/2022/6207701 (PMC9649303; doi:10.1155/2022/6207701)
Supplement: Supplementary Materials — All the dysregulated metabolites detected by LC-MS in HR-HPV infection groups were shown in Supplementary Table 1. [file 6207701.f1.pdf]

Supplementary Table 1. All the dysregulated metabolites detected by LC-MS in HR-HPV infection groups.

No.	ID	Ion mode	Metabolites	Compound ID
1	0.80_103.0390m/z	pos	2,4-Dihydroxybutanoic acid	HMDB0000360
2	5.05_187.0073m/z	neg	p-Toluenesulfonic acid	HMDB0059933
3	3.01_164.0716m/z	neg	L-Phenylalanine	HMDB0000159
4	11.84_325.2376m/z	pos	13-Hydroxy-9-methoxy-10-oxo-11-octadecenoic acid	HMDB0040901
5	4.18_172.9915m/z	neg	Phenol sulphate	HMDB0060015
6	1.23_168.0299n	neg	Uric acid	HMDB0000289
7	0.87_187.0716m/z	neg	Serylthreonine	HMDB0029049
8	6.08_137.0236m/z	neg	Salicylic acid	HMDB0000840
9	2.30_183.0525n	pos	4-Pyridoxic acid	HMDB0000017
10	7.96_239.0926m/z	neg	3-Carboxy-4-methyl-5-propyl-2-furanpropionic acid	HMDB0061112
11	4.75_263.1030m/z	neg	Phenylacetylglutamine	HMDB0006344
12	6.02_174.0556m/z	neg	Indoleacetic acid	HMDB0000197
13	4.18_204.0902n	neg	L-Tryptophan	HMDB0000929
14	9.34_392.2917n	neg	Ursodeoxycholic acid	HMDB0000946
15	0.73_156.0774m/z	pos	L-Histidine	HMDB0000177
16	9.72_448.3061m/z	neg	Deoxycholic acid glycine conjugate	HMDB0000631
17	7.32_368.1659n	neg	Dehydroepiandrosterone sulfate	HMDB0001032
18	7.66_397.2048m/z	neg	Pregnanolone sulfate	3557
19	7.82_528.2628m/z	neg	Glycochenodeoxycholate-3-sulfate	HMDB0002497
20	0.92_426.0198m/z	neg	ADP	HMDB0001341
21	2.00_180.0668m/z	neg	L-Tyrosine	34
22	9.05_279.2317m/z	pos	Alpha-Linolenic acid	HMDB0001388

23	11.24_478.2936m/z	neg	LysoPE(18:1(11Z)/0:0)	HMDB0011505
24	4.52_212.0024m/z	neg	Indoxyl sulfate	HMDB0000682
25	8.38_291.2319m/z	pos	Androsterone	HMDB0000031
26	5.50_204.0663m/z	neg	Indolelactic acid	HMDB0000671
27	6.05_191.0830m/z	pos	Carveol	HMDB0036083
28	10.86_454.2963m/z	pos	LysoPE(16:0/0:0)	HMDB0011503
29	7.56_369.1735m/z	neg	5β-Androstan-3α-ol-17-one sulfate	3564
30	9.51_449.3140n	neg	Glycoursodeoxycholic acid	HMDB0000708
31	5.15_197.0802m/z	pos	a-Methyl-3,4-dihydroxyphenylpropionic acid	1515
32	6.02_340.1329m/z	pos	N-((4-hydroxyhexahydrocyclopenta[c]pyrrol-2(1H)-yl)carbamoyl)-4-methylbenzenesulfonamide	3117
33	7.07_514.2835m/z	neg	Taurocholic acid	HMDB0000036
34	2.20_267.0728m/z	neg	Inosine	HMDB0000195
35	1.98_113.0343m/z	pos	Uracil	HMDB0000300
36	0.87_201.0874m/z	neg	Alanyl-Hydroxyproline	HMDB0028688
37	8.12_465.3102n	pos	Glycocholic acid	HMDB0000138
38	1.44_130.0507m/z	pos	Pyrrolidonecarboxylic acid	HMDB0000267
39	4.73_121.0285m/z	neg	3-Hydroxybenzaldehyde	65898
40	8.14_498.2885m/z	neg	Taurochenodesoxycholic acid	HMDB0000951
41	1.21_130.0865m/z	pos	L-Pipecolic acid	HMDB0000070
42	7.97_557.1031m/z	pos	Medicarpin 3-O-(6'-malonylgluclside)	LMPK12070006
43	1.26_192.0262n	neg	Citric acid	HMDB0000094

44	3.83_206.1383m/z	pos	dexpanthenol	3978
45	6.11_151.0394m/z	neg	Vanillin	HMDB0012308
46	8.73_267.1835n	pos	Metoprolol	1250
47	11.93_568.3638m/z	neg	PE(21:0/0:0)	LMGP02050026
48	9.40_408.2905n	pos	Cholic acid	HMDB0000619
49	2.43_167.0557m/z	pos	7-Methylxanthine	58075
50	6.32_411.1828m/z	neg	17α,20β-Hydroxyprogesterone sulfate	3562
51	10.91_540.3321m/z	neg	PE(19:0/0:0)	LMGP02050028
52	1.36_135.0304m/z	neg	Hypoxanthine	HMDB0000157
53	11.37_295.2281m/z	pos	9-OxoODE	HMDB0004669
54	8.88_495.2945m/z	neg	3-alpha,20-alpha-dihydroxy-5-beta-pregnane 3-glucuronide	57956
55	0.77_325.1121m/z	pos	D-Maltose	HMDB0000163
56	11.90_481.3208n	pos	LysoPE(18:0/0:0)	HMDB0011130
57	0.80_182.0783n	neg	D-Mannitol	142
58	7.88_532.2184m/z	pos	3,4,5-trihydroxy-6-{[5-methoxy-2,2-dimethyl-6-(3-oxo-3-phenylpropyl)-2H-chromen-7-yl]oxy}oxane-2-carboxylic acid	HMDB0125913
59	7.02_482.2944m/z	pos	Taurodeoxycholic acid	HMDB0000896
60	0.67_129.0790n	pos	NIPECOTIC ACID	44223
61	10.99_375.2400n	pos	R-4-benzyl-3-((R)-3-hydroxy-2,2-dimethyloctanoyl)-5,5-dimethyloxazolidin-2-one	65464

62	11.56_747.5673m/z	neg	SM(d18:1/16:0)	LMSP03010003
63	3.86_384.1183m/z	pos	Succinyladenosine	HMDB0000912
64	4.80_153.0184m/z	neg	2,6-Dihydroxybenzoic acid	HMDB0013676
65	11.33_530.3273m/z	pos	LysoPE(22:4(7Z,10Z,13Z,16Z)/0:0)	HMDB0011523
66	9.51_449.3166n	pos	Chenodeoxycholic acid glycine conjugate	HMDB0000637
67	13.20_280.2405n	neg	Linoleic acid	HMDB0000673
68	5.75_443.1448m/z	neg	Doxycycline	2504
69	10.26_512.2994m/z	neg	1-Heptadecanoylglycerophosphoethanolamine	HMDB0061691
70	4.03_185.1279m/z	pos	N-Acetylisoputreanine	HMDB0094713
71	7.56_209.0806m/z	pos	3,4-dimethyl-5-carboxymethyl-2-furanpropanoic acid	LMFA01150048
72	10.13_385.0934m/z	pos	2-(1,3-Benzodioxol-5-yl)-7-hydroxy-3,5,6,8-tetramethoxy-4H-1-benzopyran-4-one	LMPK12113355
73	10.17_238.1935n	pos	Germacrenone	HMDB0035887
74	10.51_538.3155m/z	neg	PE(19:1(9Z)/0:0)	LMGP02050019
75	0.87_292.0906n	neg	Edetic Acid	HMDB0015109
76	11.03_459.2542m/z	pos	Astemizole	1122
77	5.86_151.0391m/z	neg	p-Hydroxyphenylacetic acid	HMDB0000020

78	7.34_530.2039m/z	pos	3,4,5-trihydroxy-6-{[5-methoxy-2,2-dimethyl-6-(3-oxo-3-phenylprop-1-en-1-yl)-2H-chromen-7-yl]oxy}oxane-2-carboxylic acid	HMDB0125912
79	2.28_103.0400m/z	neg	(R)-3-Hydroxybutyric acid	HMDB0000011
80	11.26_566.3479m/z	neg	PC(18:1(9Z)/0:0)	LMGP01050032
81	10.80_588.3319m/z	neg	LysoPC(20:4(5Z,8Z,11Z,14Z))	HMDB0010395
82	2.00_121.0652m/z	pos	2,3-Dihydrobenzofuran	HMDB0013815
83	4.20_179.0571m/z	neg	Theophylline	1458
84	0.89_254.9806m/z	neg	Ascorbic acid-2-sulfate	HMDB0060649
85	10.72_316.1910m/z	pos	(E)-Piperolein A	HMDB0030185
86	11.16_826.5625m/z	neg	PE(17:0/22:4(7Z,10Z,13Z,16Z))	LMGP02010560
87	11.46_554.3468m/z	neg	1-O-(2-methoxy-4Z-hexadecenyl)-sn-glycero-3-phosphocholine	LMGP01060043
88	11.74_508.3403m/z	neg	LysoPC(17:0)	HMDB0012108
89	12.90_253.2171m/z	neg	Trans-Hexa-dec-2-enoic acid	HMDB0010735
90	5.12_209.0454m/z	neg	Coumaric acid	HMDB0041592
91	6.99_498.2886m/z	neg	Tauroursodeoxycholic acid	HMDB0000874
92	9.04_243.1596m/z	neg	1,11-Undecanedicarboxylic acid	6622
93	11.93_582.4310m/z	pos	Alloxanthin	HMDB0036874
94	4.31_261.1453m/z	pos	gamma-Glutamylleucine	HMDB0011171
95	8.29_663.2674m/z	pos	Ustiloxin B	HMDB0041373
96	9.66_165.0558m/z	pos	2-Hydroxycinnamic acid	HMDB0002641

97	11.68_121.0281m/z	pos	4-Hydroxybenzoic acid	HMDB0000500
98	6.90_421.0587m/z	neg	(2-{9-hydroxy-2-oxo-2H,8H,9H-furo[2,3-h]chromen-8-yl}-2-[(3-methylbut-2-enoyl)oxy]propoxy)sulfonic acid	HMDB0128935
99	12.13_719.5359m/z	neg	PE-Cer(d14:1(4E)/21:0)	LMSP03020007
100	0.75_296.0369m/z	neg	L-Cysteinylglycine disulfide	HMDB0000709
101	0.75_359.0931m/z	pos	Enoxacin	HMDB0014610
102	10.78_564.3326m/z	neg	PC(18:2(2E,4E)/0:0)	LMGP01050034
103	4.19_146.0606m/z	pos	4-formyl Indole	96392
104	0.81_179.0555m/z	neg	D-Galactose	HMDB0000143
105	10.89_552.3303m/z	neg	LysoPE(0:0/20:1(11Z))	HMDB0011482
106	3.85_137.0600m/z	pos	Sylvopinol	HMDB0030570
107	3.94_152.0699m/z	pos	6-Acetyl-2,3-dihydro-2-(hydroxymethyl)-4(1H)-pyridinone	HMDB0035178
108	4.51_268.1547m/z	pos	2-hydroxy-3-[4-hydroxy-3-(3-methylbut-2-en-1-yl)phenyl]propanoic acid	HMDB0133182
109	7.29_391.1497m/z	pos	Melatonin glucuronide	HMDB0060830
110	10.04_239.1284m/z	neg	Cucujolide IV	LMFA07040036
111	5.22_173.0821m/z	neg	Suberic acid	4243
112	7.00_339.1253n	pos	Methylhydroxygliclazide	HMDB0014029
113	8.20_151.0759m/z	pos	4-Ipomeanol	HMDB0030472
114	0.77_145.0972m/z	pos	5-Hydroxylysine	HMDB0000450

115	1.21_362.0490m/z	neg	Guanosine monophosphate	HMDB0001397
116	1.22_156.0768m/z	pos	L-2-Amino-3-(1-pyrazolyl)propanoic acid	HMDB0034267
117	12.55_277.2172m/z	neg	Gamma-Linolenic acid	HMDB0003073
118	3.00_148.0517n	pos	Cinnamic acid	HMDB0000567
119	4.76_178.0508m/z	neg	Hippuric acid	HMDB0000714
120	10.27_467.3058n	pos	LysoPC(14:0/0:0)	HMDB0010379
121	2.85_191.0592n	pos	xi-2,3-Dihydro-2-oxo-1H-indole-3-acetic acid	HMDB0035514
122	4.67_246.1713m/z	pos	2-Methylbutyroylcarnitine	HMDB0000378
123	2.00_244.0691n	neg	Uridine	HMDB0000296
124	5.22_139.0399m/z	neg	Furfuryl acetate	HMDB0034246
125	5.22_183.0296m/z	neg	alpha-Furyl methyl diketone	HMDB0032920
126	10.61_285.2063m/z	neg	Hexadecanedioic acid	5642
127	11.48_592.3621m/z	neg	LysoPC(20:2(11Z,14Z))	HMDB0010392
128	4.01_232.1562m/z	pos	Butyrylcarnitine	HMDB0002013
129	3.76_220.1181m/z	pos	Pantothenic acid	HMDB0000210
130	4.82_160.1336m/z	pos	8-aminocaprylic acid	LMFA01100059
131	5.04_319.1674m/z	pos	5,8,11,14-Eicosatetraynoic acid	35289
132	0.83_191.1034m/z	pos	Alanyl-Threonine	HMDB0028697
133	11.28_526.3508m/z	neg	PC(O-16:0/0:0)	LMGP01060010
134	11.46_189.1645m/z	pos	alpha-Irone	HMDB0035631
135	8.24_273.2228m/z	pos	16-fluoro-7Z-hexadecenoic acid	96780
136	10.32_314.2447n	pos	(±)12,13-DiHOME	45199

137	10.55_339.2667m/z	pos	3-(2,4-Cyclopentadien-1-ylidene)-5alpha-androstan-17beta-ol	70417
138	10.95_329.2446m/z	pos	9Z,11Z,14Z-Eicosatrienoic acid	LMFA01031060
139	4.86_303.1419m/z	pos	arabinofuranosylguanine	HMDB0061067
140	5.36_165.0551m/z	neg	Dihydro-3-coumaric acid	4152
141	7.83_230.1755m/z	pos	12-hydroxy-3Z,6Z-dodecadienoic acid	LMFA01050171
142	8.12_601.2674m/z	pos	APC	HMDB0060661
143	0.88_235.0934m/z	pos	L-beta-aspartyl-L-threonine	62018
144	6.26_340.1328m/z	pos	7-Hydroxygliclazide	HMDB0014027
145	6.47_285.0398m/z	neg	Luteolin	HMDB0005800
146	9.83_473.3267m/z	pos	(23S,24S)-17,23-Epoxy-24,29-dihydroxy-27-norlanost-8-ene-3,15-dione	HMDB0035843
147	1.25_183.0153m/z	neg	5-hydroxy-2-oxo-4-ureido-2,5-dihydro-1H-imidazole-5-carboxylate	HMDB0059663
148	5.76_243.0876m/z	pos	Lumichrome	64742
149	10.38_517.3218n	pos	LysoPC(18:3(9Z,12Z,15Z))	HMDB0010388
150	10.47_452.2808m/z	pos	LysoPE(16:1(9Z)/0:0)	HMDB0011504
151	11.72_490.3664n	pos	Camelliagenin C	90001
152	6.01_178.0862m/z	pos	Matricaric acid	LMFA01030751
153	6.98_161.0596m/z	pos	Coniferaldehyde	HMDB0141782
154	10.11_512.2983m/z	neg	PE(17:0/0:0)	LMGP02050030
155	11.06_590.3464m/z	neg	LysoPC(20:3(5Z,8Z,11Z))	HMDB0010393

156	12.31_269.2486m/z	pos	17-hydroxy-heptadecanoic acid	LMFA01050053
157	2.21_294.1550m/z	pos	N-(1-Deoxy-1-fructosyl)isoleucine	HMDB0039780
158	5.20_510.1974m/z	pos	4,2'-Dihydroxy-3,4',6'-trimethoxychalcone 4-glucoside	LMPK12120326
159	11.79_283.2649m/z	pos	Elaidic acid	HMDB0000573
160	13.20_330.2796n	pos	MG(0:0/16:0/0:0)	HMDB0011533
161	3.00_100.0761m/z	pos	2-Piperidinone	HMDB0011749
162	4.77_134.0609m/z	neg	2-Phenylacetamide	HMDB0010715
163	5.45_236.0921m/z	neg	N-lactoyl-Phenylalanine	HMDB0062175
164	8.58_179.1074m/z	pos	1-(4-hydroxyphenyl)pentan-3-one	HMDB0132981
165	9.14_387.2544m/z	pos	7α-Hydroxy-3-oxochola-1,4-dien-24-oic Acid	42825
166	9.83_311.2220m/z	neg	(±)13-HpODE	64784
167	10.66_526.3146m/z	neg	PE(0:0/18:0)	LMGP02050038
168	10.74_473.2030m/z	pos	Cymorcin diglucoside	HMDB0039386
169	10.74_582.2165m/z	pos	3,4,5-trihydroxy-6-({12,13,17-trihydroxy-3,4-dimethoxy-11-oxotricyclo[12.3.1.1²,⁶]nonadeca-1(17),2,4,6(19),14(18),15-hexaen-5-yl}oxy)oxane-2-carboxylic acid	HMDB0133715
170	15.37_860.6180m/z	pos	PC(20:3(8Z,11Z,14Z)/22:4(7Z,10Z,13Z,16Z))	59862
171	6.06_300.1207n	pos	2-(3-Hydroxyphenyl)ethanol 1'-glucoside	HMDB0038332
172	1.21_243.0615m/z	neg	Pseudouridine	HMDB0000767

173	1.34_123.0544m/z	pos	Niacinamide	HMDB0001406
174	10.38_562.3149m/z	neg	LysoPC(18:3(6Z,9Z,12Z))	HMDB0010387
175	10.76_612.3314m/z	neg	LysoPC(22:6(4Z,7Z,10Z,13Z,16Z,19Z))	HMDB0010404
176	11.28_524.3353m/z	neg	LysoPC(P-16:0)	HMDB0010407
177	2.85_190.0506m/z	neg	5-Hydroxyindoleacetic acid	2975
178	5.60_479.1667m/z	pos	Cappariloside B	HMDB0034938
179	6.73_188.0715m/z	neg	Indole-3-propionic acid	HMDB0002302
180	8.14_339.2700m/z	pos	tetracosahexaenoic acid	LMFA01030804
181	11.02_666.4208m/z	pos	Tragopogonsaponin A	HMDB0037910
182	11.51_552.3671m/z	neg	LysoPC(P-18:0)	HMDB0013122
183	6.80_407.2060m/z	neg	Cortisol	HMDB0000063
184	0.80_492.1936m/z	pos	a-L-Arabinofuranosyl-(1->2)-[a-D-mannopyranosyl-(1->6)]-D-mannose	HMDB0039818
185	10.42_588.4357m/z	pos	LysoPC(24:1(15Z))	HMDB0010406
186	10.96_614.3466m/z	neg	PC(22:5(4Z,7Z,10Z,13Z,16Z)/0:0)	LMGP01050142
187	12.04_376.2474m/z	pos	TUBERSTEMONINE	84994
188	5.03_151.0395m/z	neg	Methyl 2-hydroxybenzoate	HMDB0034172
189	7.63_352.0792m/z	pos	Tyramine glucuronide	HMDB0010328
190	7.90_347.2230m/z	pos	Corticosterone	HMDB0001547
191	9.33_374.2825n	pos	15-hydroxy-tetracosa-6,9,12,16,18-pentaenoic acid	LMFA01050438
192	11.79_195.1374m/z	pos	8-Hydroxy-4,8-dimethyl-4E,9-decadienoic acid	LMFA01050373

193	12.43_403.3576m/z	pos	δ-Tocopherol	232
194	15.07_263.2369m/z	pos	Farnesyl acetone	265036
195	6.50_496.2088m/z	pos	CD 1790	HMDB0060809
196	0.79_111.0442m/z	pos	Osmundalactone	HMDB0031303
197	0.87_247.0936m/z	pos	Aspartyl-Hydroxyproline	HMDB0028754
198	10.41_556.3247m/z	neg	PS(O-18:0/0:0)	LMGP03060002
199	13.03_449.3647m/z	pos	6-Deoxodolichosterone	89853
200	2.00_611.1450m/z	neg	Oxidized glutathione	HMDB0003337
201	5.61_405.1759m/z	neg	Glycyl-Glutamine	HMDB0028839
202	8.48_185.1180m/z	neg	2,6-Nonadien-1-ol	HMDB0031262
203	0.89_189.0875m/z	pos	Glycyl-Hydroxyproline	HMDB0028842
204	10.41_166.0618n	pos	L-3-Phenyllactic acid	HMDB0000563
205	10.94_311.2216m/z	neg	9(S)-HpODE	36019
206	12.13_594.3779m/z	neg	LysoPC(20:1(11Z))	HMDB0010391
207	7.70_263.1281m/z	neg	γ-CEHC	44822
208	11.01_187.1695m/z	pos	Undecanoic acid	HMDB0000947
209	6.39_340.1326m/z	pos	6-Hydroxygliclazide	HMDB0014028
210	7.77_196.1473n	pos	7Z,9E-dodecadienoic acid	LMFA01030237
211	7.97_229.1439m/z	neg	Dodecanedioic acid	LMFA01170009
212	9.69_267.1239m/z	neg	(6S)-dehydrovomifoliol	LMPR0103050009
213	0.80_365.1073m/z	pos	Trehalulose	HMDB0039727
214	5.43_255.0874m/z	neg	3,4-Dihydroxyphenylvaleric acid	HMDB0029233
215	6.01_130.0660m/z	pos	Isoquinoline	HMDB0034244

216	6.23_393.1285m/z	pos	cyclic N-Acetylserotonin glucuronide	HMDB0060812
217	6.44_364.2265n	pos	Tetrahydrocortisone	1915
218	6.57_293.0626m/z	neg	Aspartyl-Aspartate	HMDB0028749
219	9.77_236.1775n	pos	2-Hydroxyacorenone	HMDB0030916
220	1.21_262.1299m/z	pos	O-methylmalonyl-L-carnitine	HMDB0062785
221	10.32_313.2379m/z	neg	12,13-DHOME	HMDB0004705
222	4.00_247.1292m/z	pos	Aspartyl-Leucine	HMDB0028757
223	6.39_542.2739n	pos	Cortolone-3-glucuronide	HMDB0010320
224	7.34_318.1304m/z	pos	Aspartylglycosamine	HMDB0000489
225	9.03_315.1358m/z	pos	Panaquinquecol 2	HMDB0038938
226	10.40_586.3157m/z	neg	LysoPC(20:5(5Z,8Z,11Z,14Z,17Z))	HMDB0010397
227	5.71_377.1364m/z	pos	6'',6''-Dimethyl-5''-hydroxy-4'',5''-dihydropyrano[2'',3'':2',3']-4'-hydroxy-6'-methoxychalcone	LMPK12120239
228	8.56_397.2062m/z	pos	1-Hexanol arabinosylglucoside	87865
229	4.11_349.1405m/z	pos	1-(1,2,3,4,5-Pentahydroxypent-1-yl)-1,2,3,4-tetrahydro-beta-carboline-3-carboxylate	HMDB0012492
230	4.25_118.0655m/z	pos	Indole	286
231	5.03_230.9964m/z	neg	(4-ethenyl-2,6-dihydroxyphenyl)oxidanesulfonic acid	HMDB0128010
232	6.57_414.3041m/z	pos	N-oleoyl methionine	LMFA08020110
233	8.39_359.2580m/z	pos	11beta-Hydroxy-6alpha,11-dimethylpregn-4-ene-3,20-dione	70607

234	8.94_615.2439m/z	neg	8-Acetylegelolide	HMDB0037772
235	9.20_467.1121m/z	pos	5,4'-Dihydroxy-3,6,3'-trimethoxy-7-prenyloxyflavone	LMPK12112976
236	11.78_365.2690m/z	neg	Pregnanediol	LMST02030202
237	4.11_367.1512m/z	pos	Tetrahydropentoxyline	HMDB0029992
238	5.83_245.0482m/z	neg	[3-(4-methoxyphenyl)propoxy]sulfonic acid	HMDB0135750
239	1.22_158.1172m/z	pos	2E,7-Octadienoic acid	LMFA01030795
240	10.53_374.2827n	pos	12alpha-Hydroxy-5beta-chol-3-en-24-oic Acid	LMST04010213
241	10.70_432.3108m/z	neg	Lithocholic acid glycine conjugate	HMDB0000698
242	11.87_889.5363m/z	pos	PGP(18:0/22:4(7Z,10Z,13Z,16Z))	HMDB0013513
243	2.17_173.0456m/z	neg	L-erythro-5-(1-Hydroxyethyl)-2(5H)-furanone	HMDB0033285
244	5.14_121.0285m/z	neg	Benzoic acid	HMDB0001870
245	5.38_317.1129m/z	neg	(2S,4R)-4-(9H-Pyrido[3,4-b]indol-1-yl)-1,2,4-butanetriol	HMDB0035191
246	7.32_435.1444m/z	neg	3,4,5-trihydroxy-6-{[(6E)-3-oxo-1,7-diphenylhepta-4,6-dien-1-yl]oxy}oxane-2-carboxylic acid	HMDB0133920
247	7.34_506.1799n	pos	Diosbulbinoside D	HMDB0030084
248	7.83_359.1612m/z	pos	Oryzalide B	HMDB0037592
249	9.52_105.0700m/z	pos	Styrene	HMDB0034240
250	0.91_234.0847n	neg	Aspartyl-Threonine	HMDB0028763
251	11.40_708.4947m/z	pos	PE(15:0/20:4(5Z,8Z,11Z,14Z))	HMDB0008904

252	7.54_649.2549m/z	pos	Endomorphin-1	HMDB0005773
253	9.88_309.1700m/z	neg	12-Oxo-2,3-dinor-10,15-phytodienoic acid	HMDB0032090
254	1.16_277.0924m/z	pos	3,4,5-trihydroxy-6-[(3-methylbut-2-enoyl)oxy]oxane-2-carboxylic acid	HMDB0128920
255	1.25_423.0118m/z	pos	5-(acetyloxy)-12,14-dihydroxy-9-oxo-8,17-dioxatetracyclo[8.7.0.0²,⁷.0¹¹,¹⁶]heptadeca-1(10),2(7),3,5,11(16),12,14-heptaen-13-yl acetate	HMDB0128415
256	10.58_447.2747m/z	neg	(+)-Scalarin	LMPR0105060001
257	10.79_543.3372n	pos	LysoPC(20:4(8Z,11Z,14Z,17Z))	HMDB0010396
258	11.13_622.2831m/z	pos	11-[(2R)-3-[2-amino-3-methyl-4-(2-methyl-1,3-thiazol-4-yl)but-3-en-1-yl]-2-methyloxiran-2-yl]-3-hydroxy-4,4,6,8-tetramethyl-5-oxo-7-(sulfooxy)undecanoic acid	HMDB0127547
259	11.42_254.1890n	pos	8-Hydroxy-4(6)-lactarene-5,14-diol	HMDB0035780
260	11.83_421.3333m/z	pos	3Alpha,7Alpha,12Alpha-trihydroxy-27-nor-5Beta-cholestan-24-one	84749
261	5.74_201.0222m/z	neg	4-ethylphenylsulfate	HMDB0062551

262	0.84_877.3001m/z	pos	6-Methoxymusizin 8-O-[b-D-glucopyranosyl-(1->6)-b-D-glucopyranosyl-(1->3)-b-D-glucopyranosyl-(1->6)-b-D-glucopyranoside]	HMDB0034614
263	11.15_270.2202n	pos	16-hydroxy-9E-hexadecenoic acid	LMFA01050192
264	4.06_195.0518m/z	neg	1,3-Dimethyluric acid	2822
265	4.91_321.1459m/z	pos	Gravelliferone	HMDB0030729
266	5.43_238.0853n	pos	3,4,5-Trimethoxycinnamic acid	6705
267	7.80_331.2648m/z	pos	Eicosapentaenoic Acid ethyl ester	45153
268	5.92_188.0712m/z	pos	5-Methoxyindoleacetate	HMDB0004096
269	8.67_251.0916m/z	neg	2,3-Dihydro-6-methoxy-2,2-dimethyl-4H-1-benzopyran-4-one	HMDB0041410
270	10.94_590.3460m/z	neg	LysoPC(20:3(8Z,11Z,14Z))	HMDB0010394
271	11.89_480.3094m/z	neg	PE(18:0/0:0)	LMGP02050001
272	12.28_554.3829m/z	neg	PC(O-18:0/0:0)	LMGP01060014
273	4.24_199.0405n	neg	p-CHLOROPHENYLALANINE	44308
274	5.62_194.1299n	pos	Neocnidilide	HMDB0034450
275	6.28_512.2687m/z	pos	N-[(3a,5b,7b)-7-hydroxy-24-oxo-3-(sulfooxy)cholan-24-yl]-Glycine	HMDB0002409

276	7.78_401.0866m/z	neg	3-{[3,4-dihydroxy-5-(hydroxymethyl)oxolan-2-yl]oxy}-5,7-dihydroxy-2-(3-hydroxyphenyl)-3,4-dihydro-2H-1-benzopyran-4-one	HMDB0132723
277	8.99_367.2966m/z	pos	N-palmitoyl glutamine	LMFA08020127
278	0.78_369.1198m/z	neg	Secologanin	LMPR0102070002
279	1.22_567.0401m/z	pos	({6-[2-(3,4-dihydroxyphenyl)-5,6,7-trihydroxy-4-oxo-4H-chromen-8-yl]-3,4,5-trihydroxyoxan-2-yl}methoxy)sulfonic acid	HMDB0129306
280	10.38_578.2133m/z	pos	5,9,11-trihydroxyprosta-6E,14Z-dien-1-oate	HMDB0062413
281	13.41_357.3019m/z	pos	MG(18:1(11Z)/0:0/0:0)	HMDB0011566
282	2.01_229.1559m/z	pos	Leucylproline	HMDB0011175
283	7.73_307.1304m/z	pos	(R)-2,4,5-Trimethoxydalbergiquinol	LMPK12100069
284	9.77_548.2125m/z	pos	6-[1-(3,4-dimethoxyphenyl)-2-{[2-(3,4-dimethoxyphenyl)ethyl]-C-hydroxycarbonimidoyl}ethoxy]-3,4,5-trihydroxyoxane-2-carboxylic acid	HMDB0135503
285	9.96_437.2898m/z	neg	3b,12a-Dihydroxy-5a-cholanoic acid	HMDB0000348
286	1.21_157.0741n	pos	N-Acetylproline	HMDB0094701

287	2.26_153.0660m/z	pos	N1-Methyl-2-pyridone-5-carboxamide	HMDB0004193
288	3.92_232.1561m/z	pos	Isobutyryl-L-carnitine	HMDB0000736
289	4.19_247.0686m/z	pos	5-Phosphoribosylamine	HMDB0001128
290	4.73_212.0012m/z	neg	Paracetamol sulfate	HMDB0059911
291	5.75_305.1139m/z	pos	Deoxyartemsinin	HMDB0060569
292	6.19_460.1721m/z	pos	Methacycline	HMDB0015066
293	6.36_597.2868m/z	pos	Taurochenodeoxycholate-7-sulfate	HMDB0002498
294	6.75_279.1228m/z	pos	Cyclocalopin C1	HMDB0039812
295	7.32_347.1614m/z	pos	(6E,8E,10R,12Z)-10-Hydroxy-3-oxooctadecatrienoic acid	HMDB0062357
296	9.51_257.1752m/z	neg	Tetradecanedioic acid	5833
297	1.21_176.0315n	neg	(4S,5S)-4,5-dihydroxy-2,6-dioxohexanoic acid	LMFA01050449
298	1.22_103.0459n	pos	(±)-2-Methylthiazolidine	HMDB0031682
299	11.65_440.3511n	pos	Momordol	86511
300	12.06_277.2164m/z	pos	(9R,13R)-15,16-dihydro-12-oxo-10-phytoenoic acid	LMFA02010004
301	12.39_393.3014m/z	pos	11beta,12beta-Dihydroxy-5beta-cholan-24-oic Acid	LMST04010386
302	2.14_345.1334m/z	neg	Gibberellin A3	6954
303	3.00_107.0499m/z	pos	Benzaldehyde	HMDB0006115
304	4.50_146.0608m/z	pos	1H-Indole-3-carboxaldehyde	HMDB0029737
305	5.26_173.1410n	pos	3R-aminononanoic acid	LMFA01100019

306	5.76_188.0711m/z	pos	Cinnamoylglycine	HMDB0011621
307	6.01_507.2599m/z	pos	11-Hydroxyprogesterone 11-glucuronide	HMDB0010364
308	0.93_305.0981m/z	neg	Glutamyl-Hydroxyproline	HMDB0028820
309	1.48_173.0083m/z	neg	D-Glucaro-1,4-lactone	HMDB0041862
310	10.22_513.3089n	pos	1-(2-methoxy-13-methyl-pentadecanyl)-sn-glycero-3-phosphoserine	LMGP03060009
311	10.41_560.2860m/z	pos	Physagulin C	HMDB0038535
312	10.67_277.1806m/z	neg	10-Deoxymethynolide	LMPK04000034
313	10.94_367.1585m/z	pos	2,3-Butanediol apiosylglucoside	HMDB0033063
314	5.04_512.2136m/z	pos	Harpagoside	LMPR0102070010
315	6.24_255.1137m/z	neg	Imazamethabenz	HMDB0034885
316	9.30_682.4068m/z	pos	PS(12:0/18:4(6Z,9Z,12Z,15Z))	LMGP03010056
317	9.66_294.1831n	neg	Tanacetol A	HMDB0035722
318	0.91_291.0882m/z	neg	7-hydroxy-6-(3-hydroxy-3-methylbut-1-en-1-yl)-2H-chromen-2-one	HMDB0130516
319	0.91_425.0792m/z	neg	Cysteineglutathione disulfide	HMDB0000656
320	10.53_299.2820n	pos	3-ketosphinganine	3428
321	12.85_328.2432n	pos	Docosahexaenoic acid	HMDB0002183
322	13.04_303.2329m/z	neg	Arachidonic acid	HMDB0001043
323	2.01_249.0580m/z	pos	2,3,4,5,6,7-Hexahydroxyheptanoic acid	HMDB0240292
324	4.41_261.0070m/z	neg	3-(3,5-dihydroxyphenyl)-1-propanoic acid sulphate	HMDB0061117

325	4.92_203.0013m/z	neg	O-methoxycatechol-O-sulphate	HMDB0060013
326	6.14_336.0635m/z	pos	(S)-Annocherine A	HMDB0038723
327	7.09_515.2932n	pos	Tauroursocholic acid	57999
328	10.19_475.2039m/z	pos	5,10-Methylene-THF	HMDB0001533
329	10.80_279.2322m/z	neg	10E,12Z-Octadecadienoic acid	34801
330	11.02_453.2891n	pos	Sambutoxin	95518
331	12.83_802.5636m/z	neg	PE(15:0/22:2(13Z,16Z))	LMGP02010471
332	3.85_181.0720m/z	pos	Theobromine	HMDB0002825
333	4.21_344.0908m/z	pos	1-Anilino-9,10-dioxo-2-anthroic acid	96354
334	4.83_177.0913m/z	pos	3-Hydroxyphenyl-valeric acid	HMDB0041666
335	5.18_379.1512m/z	pos	Myricanone	HMDB0030798
336	6.24_299.1020m/z	neg	fluvoxamino acid	HMDB0060950
337	6.60_168.0437n	pos	Vanillic acid	HMDB0000484
338	9.84_277.2173m/z	pos	(9S,13S)-15,16-dihydro-12-oxo-10-phytoenoic acid	LMFA02010005
339	0.77_120.0659m/z	pos	4-Amino-3-hydroxybutyrate	HMDB0061877
340	10.46_660.4573m/z	pos	PS(P-16:0/13:0)	LMGP03030002
341	12.32_531.3693m/z	pos	Acinospesigenin A	LMPR0106160001
342	5.01_303.1423m/z	pos	Valproic acid glucuronide	HMDB0000901
343	5.32_155.1072m/z	pos	9-hydroxy-5Z-nonenoic acid	LMFA01050232
344	7.40_375.1549m/z	pos	Gravacridonetriol	HMDB0029330
345	8.78_193.0866m/z	neg	Butylparaben	HMDB0032575
346	9.30_644.4021m/z	pos	Ponasteroside A	HMDB0034091

347	1.26_147.0291m/z	neg	2,3,4,5-Tetrahydroxypentanoic acid	HMDB0059750
348	10.58_309.2065m/z	neg	13(S)-HpOTrE	36052
349	11.05_480.3099m/z	neg	LysoPC(15:0)	HMDB0010381
350	14.34_673.5304m/z	pos	PE-Cer(d15:1(4E)/20:0(2OH))	LMSP03020074
351	4.31_227.9965m/z	neg	2-Hydroxyacetaminophen sulfate	HMDB0062547
352	4.81_321.1457m/z	pos	Nicotine glucuronide	HMDB0001272
353	5.32_277.0869m/z	pos	3',4'-dihydroxyflurbiprofen	2925
354	9.29_546.1881m/z	pos	2-amino-4-({1-[(carboxymethyl)-C-hydroxycarbonimidoyl]-2-{[2-hydroxy-3-(4-hydroxy-2-methoxyphenyl)-1-phenylpropyl]sulfanyl}ethyl}-C-hydroxycarbonimidoyl)butanoic acid	HMDB0130358
355	10.87_181.1595m/z	pos	5Z-Dodecenoic acid	HMDB0000529
356	11.18_311.1682m/z	neg	N-Undecylbenzenesulfonic acid	HMDB0032549
357	11.72_489.3575m/z	neg	Alisol A	71704
358	6.60_127.0395m/z	pos	4-HYDROXY-6-METHYLPYRAN-2-ONE	44653
359	7.63_350.0805m/z	pos	m-Carboxyphenyl phenylacetamidomethylphosphonate	66239
360	7.78_193.0503m/z	pos	2,10-dihydroxy-4,6,8-decatriynoic acid	LMFA01050235
361	8.62_181.1218m/z	pos	(+)-Myrtenyl formate	91711

362	9.31_187.1337m/z	neg	10-hydroxy capric acid	LMFA01050033
363	9.83_453.3700m/z	pos	23-phenyl tricosanoic acid	LMFA01140072
364	11.44_195.1752m/z	pos	2,6,10,10-Tetramethyl-1-oxaspiro[4.5]decan-6-ol	HMDB0037907
365	13.23_405.3379m/z	pos	2-Nor-1,3-seco-1α,25-dihydroxyvitamin D3	42605
366	4.41_114.0915m/z	pos	6-amino-hexanoic acid	LMFA01100035
367	6.29_191.0699m/z	pos	5-(3',4'-Dihydroxyphenyl)-gamma-valerolactone	HMDB0029185
368	7.19_319.1317m/z	pos	Tephroleocarpin B	LMPK12140183
369	8.32_326.2335m/z	pos	9-J1-PhytoP	LMFA02030069
370	1.25_139.0034m/z	pos	2,5-Furandicarboxylic acid	HMDB0004812
371	11.02_317.1382m/z	pos	2-Hydroxy-4,5,6-trimethoxydihydrochalcone	52412
372	11.68_149.0241m/z	pos	Benzoquinoneacetic acid	HMDB0002334
373	11.85_830.5932m/z	neg	PE(18:2(9Z,12Z)/21:0)	LMGP02010674
374	12.54_271.2285m/z	neg	Pentadecanal	HMDB0031078
375	5.45_171.0658m/z	neg	but-2-enoic acid	LMFA01030925
376	5.58_341.1145m/z	pos	6-Ketoestriol	HMDB0000530
377	7.84_255.1137m/z	pos	10-Hydroxycarbazepine	HMDB0060676
378	9.17_196.1465n	pos	Aleprylic acid	LMFA01030189
379	9.80_331.2284m/z	pos	11-deoxy-11-methylene-15-keto-PGD2	LMFA03010104
380	0.76_352.0709m/z	pos	Lansoprazole	HMDB0005008
381	0.77_241.0308m/z	pos	L-Cystine	HMDB0000192

382	0.77_265.0904m/z	pos	Xylobiose	HMDB0029894
383	0.91_897.2475m/z	neg	Rhamnetin 3-(3''''-p-coumaryl-rhamnosyl)(1->3)-rhamnosyl-(1->6)-galactoside	LMPK12112606
384	11.50_550.3509m/z	neg	LysoPC(P-18:1(9Z))	HMDB0010408
385	12.15_448.3564n	pos	Typhasterol	64145
386	12.53_223.2067m/z	pos	Selina-6-en-4-ol	265102
387	2.05_247.0525m/z	pos	2',2'-Difluorodeoxyuridine	HMDB0060727
388	2.14_277.0343m/z	neg	4-Hydroxy-8-methoxy-2H-furo[2,3-h]-1-benzopyran-2-one	HMDB0032659
389	2.35_203.0518m/z	pos	D-Glucose	HMDB0000122
390	4.80_287.0221m/z	neg	4-Hydroxy-5-(dihydroxyphenyl)-valeric acid-O-sulphate	HMDB0059978
391	8.08_631.2769m/z	pos	Brassica napus non-fluorescent chlorophyll catabolite 3	HMDB0040917
392	8.17_229.1440m/z	neg	Undecylenic acid	LMFA01030036
393	0.91_146.1180m/z	pos	Ethyl 4-pentenoate	HMDB0031603
394	10.77_500.2780m/z	neg	PE(0:0/20:4(5Z,8Z,11Z,14Z))	LMGP02050051
395	10.99_259.1681m/z	pos	CICUTOXIN	LMFA05000647
396	3.84_318.1139m/z	pos	(2R,4R)-tert-butyl 4-(hydroxymethyl)-2-phenylthiazolidine-3-carboxylate	65471
397	4.35_422.1453m/z	pos	cis-Resveratrol 3-O-glucuronide	HMDB0041711
398	9.29_231.1138m/z	pos	(E)-5,8-Megastigmadien-4-one	HMDB0034671

399	10.78_504.3094m/z	neg	PC(17:2(9Z,12Z)/0:0)	LMGP01050127
400	12.65_237.2215m/z	pos	(Z)-13-Hexadecenoic acid	HMDB0035877
401	2.24_276.1448m/z	pos	N-(1-Deoxy-1-fructosyl)leucine	HMDB0037840
402	3.00_188.0686m/z	pos	Triethanolamine	HMDB0032538
403	6.40_339.0978m/z	pos	1,3-Diacetoxy-4,6,12-tetradecatriene-8,10-diyne	HMDB0030922
404	8.59_407.2803m/z	pos	3a,7a,12a,19-Tetrahydroxy-5b-cholanoic acid	HMDB0000340
405	8.81_285.1251m/z	pos	11Z-Hexadecen-7,9-diynoic acid	LMFA01031108
406	8.87_583.2604m/z	pos	Biliverdin	HMDB0001008
407	9.19_347.1619m/z	neg	Enalaprilat	2533
408	10.25_307.1908m/z	neg	Methylgingerol	86546
409	10.43_302.3075m/z	pos	Sphinganine	HMDB0000269
410	10.60_178.1363n	pos	Quinceoxepine	HMDB0038107
411	11.73_494.3626m/z	pos	1-(2-methoxy-nonadecanyl)-sn-glycero-3-phosphoethanolamine	LMGP02060022
412	3.89_208.1338m/z	pos	Synephrine acetonide	HMDB0039939
413	4.22_245.0955m/z	pos	Methionyl-Hydroxyproline	HMDB0028974
414	5.50_205.0746n	pos	3-Indolehydracrylic acid	HMDB0059765
415	5.79_187.0972m/z	neg	4-ene-Valproic acid	LMFA01030982
416	6.25_254.1064n	pos	(R)-MHD	HMDB0060701
417	6.34_333.1452m/z	pos	Myrsinone	HMDB0040646
418	6.65_347.1613m/z	pos	(6E,8E,10S,12Z)-10-Hydroxy-3-oxooctadecatrienoic acid	HMDB0062359

419	7.12_347.1630m/z	pos	Corchorifatty acid D	HMDB0033243
420	8.73_109.1010m/z	pos	2,4,4-Trimethylcyclopentanone	HMDB0031197
421	8.73_151.1484m/z	pos	4-undecenal	LMFA06000066
422	9.66_221.1542m/z	neg	(1S,2S)-3-oxo-2-pentyl-cyclopentanebutanoic acid	LMFA02010016
423	9.76_584.3560m/z	neg	PS(O-20:0/0:0)	LMGP03060001
424	9.86_570.2734n	pos	Pipercyclobutanamide A	HMDB0036360
425	2.01_183.0405m/z	neg	5-Oxo-2(5H)-isoxazolepropanenitrile	HMDB0031338
426	4.24_162.0557m/z	neg	Beta-Tyrosine	HMDB0003831
427	4.92_510.1978m/z	pos	Okanin 3,4,3'-trimethyl ether 4'-glucoside	LMPK12120174
428	5.95_342.0976m/z	pos	5-hydroxy-2-{[1-hydroxy-3-(4-hydroxy-3-methoxyphenyl)prop-2-en-1-ylidene]amino}-4-methoxybenzoic acid	HMDB0137670
429	6.05_333.1453m/z	pos	(4E)-7-(4-hydroxy-3-methoxyphenyl)-1-phenylhept-4-en-3-one	HMDB0138247
430	7.06_231.1380m/z	pos	Marasmen-3-one	HMDB0036040
431	9.83_239.1285m/z	neg	Cucujolide IX	LMFA07040037
432	0.79_118.0865m/z	pos	5-Aminopentanoic acid	HMDB0003355
433	0.81_381.0981m/z	neg	3,4,5-trihydroxy-6-{3-[(2E)-3-phenylprop-2-enoyl]phenoxy}oxane-2-carboxylic acid	HMDB0135447

434	1.21_367.0117m/z	neg	{3,5,6-trihydroxy-2-[3-(sulfooxy)phenyl]-7H-chromen-7-ylidene}oxidanium	HMDB0130444
435	10.26_167.1437m/z	pos	(Z)-3-Methyl-3-decenoic acid	HMDB0031062
436	10.57_385.0915m/z	pos	Tetraphyllin B sulfate	HMDB0038486
437	10.64_705.1644m/z	pos	8-Hydroxyhesperetin 7-[6-acetylglucosyl-(1->2)-glucoside]	HMDB0041232
438	2.82_367.1522m/z	pos	Isoachifolidiene	HMDB0038690
439	5.48_199.0063m/z	neg	4-Vinylphenol sulfate	HMDB0062775
440	7.18_466.3046m/z	pos	N-docosahexaenoyl histidine	LMFA08020134
441	7.63_315.1356m/z	pos	Sugeonyl acetate	LMPR0103450004
442	7.74_255.1135m/z	pos	(S)-MHD	HMDB0060702
443	7.97_377.0408m/z	pos	Neotenone	LMPK12050454
444	8.91_398.2817n	pos	Δ-6 Testosterone Enanthate	64682
445	9.06_275.1228m/z	pos	3,7,7,10-Tetramethyl-12-thiabicyclo[9.1.0]dodeca-3,7-diene	HMDB0036469
446	0.77_431.1559m/z	neg	Benzyl gentiobioside	HMDB0041515
447	0.89_621.1270m/z	neg	Quercetin 3-(6"-ferulylglucoside)	LMPK12112145
448	10.52_314.1896n	pos	4-Oxoisotretinoin	871
449	10.74_477.2856n	neg	LysoPE(18:2(9Z,12Z)/0:0)	HMDB0011507

450	11.02_588.2390m/z	pos	2-amino-4-({1-[(carboxymethyl)-C-hydroxycarbonimidoyl]-2-[(2-hydroxy-5-oxo-1,7-diphenylheptyl)sulfanyl]ethyl}-C-hydroxycarbonimidoyl)butanoic acid	HMDB0133948
451	12.62_745.5522m/z	neg	PE-Cer(d14:2(4E,6E)/23:0)	LMSP03020041
452	12.87_195.1238m/z	pos	N'-nitrosonornicotine	HMDB0041940
453	13.79_815.6042n	pos	PS(P-20:0/19:1(9Z))	LMGP03030074
454	5.56_319.1316m/z	pos	Methyl (3b,11x)-3-Hydroxy-8-oxo-6-eremophilen-12-oate	HMDB0041229
455	6.11_153.0550m/z	pos	Phenoxyacetic acid	HMDB0031609
456	6.50_313.1546m/z	pos	3-hydroxy-1-methyl-4,5-diphenylpiperidine-2,6-dione	HMDB0134789
457	6.57_528.2628m/z	neg	N-[(3a,5b,7a)-3-hydroxy-24-oxo-7-(sulfooxy)cholan-24-yl]-Glycine	HMDB0002496
458	6.76_418.1446m/z	pos	3'-Amino-3'-deoxythimidine glucuronide	HMDB0060751
459	0.79_117.0793n	pos	Betaine	HMDB0000043
460	1.21_504.0907n	pos	3,4,5-trihydroxy-6-({5-hydroxy-13,14-dimethoxy-9-oxo-8,17-dioxatetracyclo[8.7.0.0²,⁷.0¹¹,¹⁶]heptadeca-1(10),2,4,6,11,13,15-heptaen-4-yl}oxy)oxane-2-carboxylic acid	HMDB0128466

461	10.51_493.3208n	pos	Tiamulin	43351
462	10.64_353.2335m/z	pos	15R-PGD2	36166
463	11.36_616.3619m/z	neg	LysoPC(22:4(7Z,10Z,13Z,16Z))	HMDB0010401
464	11.53_339.2531m/z	neg	15(S)-HpEDE	35361
465	11.94_668.2876m/z	neg	Leukotriene C5	HMDB0012993
466	2.00_118.0423n	pos	Benzofuran	HMDB0032929
467	2.07_337.0804m/z	pos	S-Nitrosoglutathione	HMDB0004645
468	2.44_316.0990m/z	pos	4-Hydroxyproline galactoside	HMDB0029354
469	3.01_147.0448m/z	neg	trans-Cinnamic acid	HMDB0000930
470	4.19_187.0667n	pos	N-butyryl-L-Homocysteine thiolactone	45311
471	5.58_315.2104m/z	pos	2,2-difluoro-hexadecanoic acid	LMFA01090042
472	6.00_451.1396m/z	neg	3,4,5-trihydroxy-6-{3-hydroxy-5-[(E)-2-(4-hydroxyphenyl)ethenyl]-2-[(1E)-3-methylbuta-1,3-dien-1-yl]phenoxy}oxane-2-carboxylic acid	HMDB0129028
473	7.14_195.1378m/z	pos	Dihydrojasmonic acid	HMDB0033601
474	7.53_167.9937m/z	pos	2(3H)-Benzothiazolethione	HMDB0030524
475	9.56_179.1071m/z	pos	5S,6S-epoxy-7-undecynoic acid	LMFA01070039
476	11.18_337.2396m/z	pos	Leukotriene B4	HMDB0001085
477	11.93_678.4872m/z	pos	PE(18:4(6Z,9Z,12Z,15Z)/P-16:0)	HMDB0009213
478	2.37_202.0583n	neg	Penmacric acid	HMDB0029436
479	3.79_188.9864m/z	neg	Pyrocatechol sulfate	HMDB0059724
480	3.91_211.1078m/z	pos	Pyroglutamylvaline	HMDB0094651

481	4.72_165.0189m/z	neg	Phthalic acid	HMDB0002107
482	4.75_129.0551m/z	pos	6-hydroxy-2-hexynoic acid	LMFA01050273
483	5.24_139.0391m/z	pos	Ascladiol	HMDB0029610
484	6.22_275.1028m/z	pos	(1xi,3S)-1,2,3,4-Tetrahydro-1-methyl-beta-carboline-1,3-dicarboxylic acid	HMDB0035115
485	8.12_429.2900n	pos	GANT 61	96517
486	9.43_478.2008m/z	pos	LTE4	LMFA03020002
487	0.75_298.0583m/z	pos	Lenalidomide	HMDB0014623
488	10.40_463.3055m/z	neg	24-northornasterol A	LMST01010320
489	11.06_546.3578m/z	pos	PC(20:3(8Z,11Z,14Z)/0:0)	LMGP01050133
490	11.26_506.3248m/z	neg	PC(17:1(10Z)/0:0)	LMGP01050002
491	11.81_250.1449m/z	neg	Tetranor-8-NO2-CLA	LMFA01120009
492	2.03_343.1267n	pos	N-(1-Deoxy-1-fructosyl)tyrosine	HMDB0037845
493	2.14_130.0867m/z	neg	L-Leucine	HMDB0000687
494	4.75_105.0333m/z	pos	3-(2-Furanyl)-2-propenal	HMDB0032918
495	4.83_243.1377m/z	pos	10-Hydroxy-3-methoxy-1,3,5,7-cadinatetraen-9-one	HMDB0036456
496	4.97_209.0451m/z	neg	(2E)-3-(2,4-dihydroxy-5-methoxyphenyl)prop-2-enoic acid	HMDB0125514
497	5.01_472.2570n	pos	PA(21:4(6Z,9Z,12Z,15Z)/0:0)	LMGP10050003
498	6.93_354.1118m/z	pos	Cyclopenta[c]pyrrole, benzoic acid deriv	3116
499	8.38_347.2229m/z	pos	Insignin A	LMST02030222
500	8.63_151.1125m/z	pos	Ethyl (4Z)-4,7-octadienoate	LMFA07010846

501	0.95_623.1379n	pos	(6-carboxy-3,4,5-trihydroxyoxan-2-yl)({4-[6,11-dihydroxy-7-(4-hydroxy-3,5-dimethoxyphenyl)-2,8-dioxatricyclo[7.3.1.0^{5,13}]trideca-1(13),4,6,9,11-pentaen-3-ylidene]cyclohexa-2,5-dien-1-ylidene})oxidanium	HMDB0134842
502	10.20_390.1378m/z	pos	Veranisatin C	HMDB0031756
503	10.73_524.2784m/z	neg	PE(0:0/22:6(4Z,7Z,10Z,13Z,16Z,19Z))	LMGP02050060
504	10.99_130.0864m/z	pos	(2R,3R,4R)-2-Amino-4-hydroxy-3-methylpentanoic acid	HMDB0029449
505	12.19_670.4923n	pos	PA(P-18:0/17:2(9Z,12Z))	LMGP10030038
506	2.79_247.1305m/z	pos	3-Methyl-5-pentyl-2-furanpropanoic acid	HMDB0112084
507	4.74_264.1149n	pos	CAY10587	45468
508	4.96_265.0333m/z	pos	5-Acetylamino-6-formylamino-3-methyluracil	HMDB0011105
509	5.51_237.0768m/z	pos	Austdiol	HMDB0030858
510	6.41_361.1538m/z	pos	phenyl [1-(N-succinylamino)pentyl]phosphonate	LMFA08020199
511	7.59_242.1761m/z	neg	N-Undecanoylglycine	HMDB0013286
512	8.15_495.3787m/z	pos	alpha-Tocopherol acetate	HMDB0034227
513	8.67_207.1021m/z	neg	(Z)-3-Oxo-2-(2-pentenyl)-1-cyclopenteneacetic acid	HMDB0030197

514	9.66_627.3304m/z	pos	25-Hydroxyvitamin D2-25-glucuronide	HMDB0010342
515	1.25_409.0231m/z	pos	{5-hydroxy-11-methoxy-16,18-dioxo-6,8,19-trioxapentacyclo[10.7.0.0²,⁹.0³,⁷.0¹³,¹⁷]nonadeca-1(12),2(9),10,13(17)-tetraen-4-yl}oxidanesulfonic acid	HMDB0126048
516	10.66_500.2777m/z	neg	PE(0:0/20:4(8Z,11Z,14Z,17Z))	LMGP02050052
517	10.70_480.3443m/z	pos	1-(2-methoxy-octadecanyl)-sn-glycero-3-phosphoethanolamine	LMGP02060021
518	11.09_295.2269m/z	neg	9R,10S-EpOME	43441
519	11.25_436.2821m/z	neg	PE(P-16:0e/0:0)	HMDB0011152
520	13.77_539.4314m/z	neg	Ambrettolic acid	LMFA01050106
521	2.04_277.0910m/z	pos	Dyphylline	HMDB0014789
522	4.60_197.1301m/z	pos	5,7alpha-Dihydro-1,4,4,7a-tetramethyl-4H-indene	HMDB0036683
523	5.04_361.1428m/z	pos	Demethylcalabaxanthone	HMDB0030656
524	5.20_453.0847m/z	neg	{[(3E)-4-{4-[(E)-2-(3,4-dihydroxyphenyl)ethenyl]-2,6-dihydroxyphenyl}-2-methylbut-3-en-1-yl]oxy}sulfonic acid	HMDB0129084
525	6.78_408.2759m/z	pos	Digoxigenin	LMST01120008
526	7.22_287.1409m/z	pos	2E,6Z,8Z,12E-hexadecatetraenoic acid	LMFA01030277
527	8.90_272.1293m/z	pos	Piperyline	68174

528	9.62_231.1137m/z	pos	2-Oxomelatonin	HMDB0060721
529	0.91_143.0815m/z	pos	4-Acetamido-2-aminobutanoic acid	HMDB0031411
530	12.27_463.3417m/z	neg	3alpha,7alpha,12alpha-Trihydroxy-24-methyl-5beta-Cholestan-26-oic acid	LMST04040002
531	15.45_550.4218n	pos	Cholestanyl glucoside	LMST01010366
532	4.18_247.1297m/z	pos	Aspartyl-Isoleucine	HMDB0028756
533	4.28_317.1495m/z	pos	3'-N'-Acetylfusarochromanone	HMDB0037499
534	4.73_377.1482m/z	pos	Riboflavin	HMDB0000244
535	5.93_286.2053m/z	pos	N-(p-Hydroxyphenethyl)actinidine	HMDB0030347
536	6.13_335.1256m/z	pos	2',3,4',5-Tetrahydroxy-4-prenylstilbene	HMDB0035478
537	6.84_182.1315n	pos	7-undecynoic acid	LMFA01030615
538	7.56_437.1593m/z	neg	3,4,5-trihydroxy-6-{[(1E)-5-oxo-1,7-diphenylhept-1-en-3-yl]oxy}oxane-2-carboxylic acid	HMDB0133936
539	8.17_448.3058m/z	neg	Chenodeoxyglycocholate	LMST05030003
540	8.32_353.2806m/z	pos	N-goshuyoyl lysine	LMFA08020265
541	0.77_243.0853n	neg	Cytidine	HMDB0000089
542	0.92_205.1199m/z	pos	2E,4E-undecadienoic acid	LMFA01030222
543	10.04_449.3518m/z	pos	LysoSM(d18:0)	HMDB0012082
544	10.30_586.3152m/z	neg	PC(20:5(5Z,8Z,11Z,14Z,17Z)/0:0)	LMGP01050050
545	10.90_324.2547m/z	pos	1,17-dihydroxy-androstan-3-one	LMST02020111
546	12.24_552.3669m/z	neg	PC(O-18:1(11Z)/0:0)	LMGP01060034

547	12.26_252.2100n	pos	8Z,10Z-hexadecadienoic acid	LMFA01030274
548	2.00_204.0631m/z	pos	4-Hydroxy-4-(3-pyridyl)-butanoic acid	HMDB0001119
549	4.47_293.1135m/z	neg	gamma-Glutamylphenylalanine	HMDB0000594
550	5.07_548.3601m/z	pos	3-keto Fusidic acid	HMDB0060745
551	5.36_629.1182m/z	neg	{[5-({4,5-dihydroxy-2-[4-(7-hydroxy-4-oxo-3,4-dihydro-2H-1-benzopyran-2-yl)phenoxy]-6-(hydroxymethyl)oxan-3-yl}oxy)-3,4-dihydroxyoxolan-3-yl]methoxy}sulfonic acid	HMDB0124936
552	5.97_255.1134m/z	neg	1-(9H-Pyrido[3,4-b]indol-1-yl)-1,4-butanediol	HMDB0035193
553	8.22_241.1439m/z	neg	2E,4E-dodecadienoic acid	LMFA01030230
554	9.44_309.1698m/z	neg	all-trans-7-hydroxyhexadeca-2,4,8,10-tetraenoic acid	LMFA01050502
555	9.60_242.2123m/z	pos	Alepric acid	LMFA01030191
556	0.87_248.1000n	neg	gamma-Glutamylthreonine	HMDB0029159
557	0.92_144.1024m/z	pos	xi-4-Hydroxy-4-methyl-2-cyclohexen-1-one	HMDB0033629
558	10.55_403.1357m/z	pos	8-Oxodiacetoxyscirpenol	HMDB0037056
559	10.98_263.2080m/z	pos	Lysyl-Valine	HMDB0028964

560	11.35_280.2405n	pos	11Z,15Z-octadecadienoic acid	LMFA01030300
561	12.39_391.2842m/z	neg	2E,6Z-dodecadienoic acid	LMFA01030231
562	6.39_335.1304m/z	pos	Estradiol-17beta 3-sulfate	LMST05020005
563	6.69_340.1326m/z	pos	N-oxide gliclazide	3119
564	7.27_622.3230m/z	neg	(3a,5b,7a,12a)-24-[(carboxymethyl)amino]-1,12-dihydroxy-24-oxocholan-3-yl-b-D-Glucopyranosiduronic acid	LMST05010053
565	7.86_427.1648m/z	pos	Ramiprilat	HMDB0060579
566	8.44_306.2565n	pos	8Z,12E,14Z-eicosatrienoic acid	LMFA01030388
567	9.16_568.3251n	pos	Deoxycholic acid 3-glucuronide	LMST05010046
568	9.40_318.3015m/z	pos	17-hydroxy stearic acid	LMFA02000135
569	0.96_176.0792n	neg	Threoninyl-Glycine	HMDB0029061
570	10.18_194.0819m/z	neg	2-(2,6-dihydroxy-3,4-dimethoxycyclohexylidene)acetonitrile	HMDB0125517
571	10.78_632.3188m/z	neg	OKHdiA-PE	LMGP20020041
572	10.97_592.2331m/z	pos	2-Hydroxyestrone-1-S-glutathione	HMDB0012623
573	12.54_333.1567m/z	pos	Glutaminyltryptophan	HMDB0028808
574	2.79_245.1137m/z	neg	gamma-Glutamylvaline	HMDB0011172
575	4.19_159.0922m/z	pos	Serotonin	HMDB0000259
576	4.21_362.1015m/z	pos	S-Lactoylglutathione	HMDB0001066
577	4.53_378.1181m/z	pos	Rosmarinic acid	HMDB0003572

578	4.69_243.1344m/z	pos	gamma-Glutamylisoleucine	HMDB0011170
579	4.70_183.0293m/z	neg	2-Methoxy-1,4-benzoquinone	HMDB0032576
580	5.54_376.1265n	pos	3,5-Pyridinedicarboxylic acid, 1,4-dihydro-2,6-dimethyl-4-(3-nitrophenyl)-, 2-hydroxyethyl methyl es	1507
581	5.60_361.1433m/z	pos	6,11-Dihydroxy-3-methyl-3-(4-methyl-3-pentenyl)-3H,7H-pyrano[2,3-c]xanthen-7-one	HMDB0040350
582	5.86_349.0034m/z	neg	{4-[2,3-dioxo-3-(2,4,6-trihydroxyphenyl)propyl]phenyl}oxidanesulfonic acid	HMDB0125403
583	6.06_255.1136m/z	neg	Naphazoline	HMDB0015656
584	6.84_405.1889m/z	neg	Tryptophyl-Arginine	HMDB0029077
585	7.90_237.0763m/z	pos	(S)-2,3-Dihydro-7-hydroxy-2-methyl-4-oxo-4H-1-benzopyran-5-acetic acid	94916
586	8.14_499.2994n	pos	5β-CHOLANIC ACID-3α, 12α-DIOL N-(2-SULPHOETHYL)-AMIDE	44688
587	9.31_663.3592m/z	neg	Neomenthol-glucuronide	HMDB0060012
588	9.40_408.2861n	neg	Allocholic acid	5491
589	0.78_371.1362m/z	neg	Loganin	LMPR0102070001
590	0.91_102.0549m/z	pos	Hydroxyethyl glycine	HMDB0061148
591	1.25_111.0077m/z	pos	5-Hydroxy-2-furoic acid	HMDB0059784
592	10.97_550.3516m/z	neg	CerP(d18:1/8:0)	LMSP02050012

593	11.06_608.3188m/z	neg	OOB-PE	LMGP20020034
594	11.71_482.3300m/z	pos	7-Dehydrocholesterol-3-sulfate ester	LMST03010067
595	13.33_628.5053m/z	pos	CerP(d18:1/18:0)	LMSP02050004
596	13.64_467.3736m/z	neg	(5Z,9Z)-2-methoxy-hexacosadienoic acid	LMFA01080023
597	14.94_124.0875m/z	pos	L-Histidinol	HMDB0003431
598	4.63_195.0893m/z	pos	Caffeine	HMDB0001847
599	4.71_279.0982m/z	pos	Obtusaquinol	LMPK12100067
600	5.04_480.2132m/z	pos	Nicardipine	HMDB0014760
601	5.79_271.1677m/z	pos	4-[3]-ladderane-butanoic acid	LMFA01140010
602	6.36_243.2111m/z	pos	6-[1]-ladderane hexanol	LMFA01140088
603	6.81_229.1671n	pos	N-Decanoylglycine	HMDB0013267
604	7.04_345.1464m/z	pos	5'-Carboxy-gamma-chromanol	HMDB0012799
605	8.73_175.1489m/z	pos	4-(4-Methyl-3-pentenyl)-3-cyclohexene-1-carboxaldehyde	HMDB0031733
606	9.20_631.2412m/z	neg	Ichangin 4-glucoside	HMDB0039340
607	9.52_527.2921m/z	pos	7',8'-Dihydro-8'-hydroxycitraniaxanthin	HMDB0036884
608	1.48_177.0399m/z	neg	Gluconolactone	HMDB0000150
609	11.14_221.1537m/z	neg	(1R,2R)-3-oxo-2-pentyl-cyclopentanebutanoic acid	LMFA02010017
610	13.55_688.5547n	pos	PE-Cer(d14:1(4E)/22:0)	103047
611	13.98_380.2189m/z	pos	PE(12:0/0:0)	LMGP02050005
612	2.05_233.0627m/z	pos	D-altro-D-manno-Heptose	HMDB0029952

613	2.09_260.1646m/z	pos	6E,8E,14E-Hexadecatriene-10,12-diynoic acid	LMFA01030704
614	4.03_125.0842n	pos	6-Acetyl-1,2,3,4-tetrahydropyridine	HMDB0030345
615	4.72_259.0131m/z	neg	3-hydroxy-2-methyl-2-[(sulfooxy)methyl]propanoic acid	HMDB0127651
616	5.04_245.1533m/z	pos	6E,8E,12E,14E-Hexadecatetraen-10-ynoic acid	LMFA01030703
617	6.15_377.1364m/z	pos	7,14,16-trihydroxy-3-(hydroxymethyl)-3,4,5,6,7,8,9,10,11,12-decahydro-1H-2-benzoxacyclotetradecin-1-one	HMDB0136047
618	6.80_315.1357m/z	pos	Ginsenoyne C	LMFA05000662
619	9.20_336.1073m/z	pos	3-(3-hydroxyphenyl)-1-(2,4,6-trihydroxy-3-methoxyphenyl)propane-1,2-dione	HMDB0128760
620	10.57_435.1395m/z	pos	Garcimangosone C	HMDB0036984
621	11.14_569.3519n	pos	LysoPC(22:5(7Z,10Z,13Z,16Z,19Z))	HMDB0010403
622	11.64_201.1858m/z	pos	8,8-Dimethoxy-2,6-dimethyl-2-octanol	HMDB0040219
623	11.79_403.2468m/z	pos	16,16-dimethyl-PGE2	36136
624	11.99_209.1911m/z	pos	cis-tetradec-11-enoic acid	LMFA01030770
625	12.85_256.2654m/z	pos	Palmitic amide	HMDB0012273
626	13.05_447.3487m/z	pos	KH 1049	57904
627	15.26_429.3735m/z	pos	13'-Hydroxy-alpha-tocopherol	HMDB0012559
628	2.00_163.0396m/z	neg	Phenylpyruvic acid	HMDB0000205

629	2.00_322.0784m/z	pos	Thymidine 3',5'-cyclic monophosphate	HMDB0001570
630	3.00_211.1085m/z	pos	Hydroxyprolyl-Proline	HMDB0028871
631	4.54_333.1444m/z	pos	(2E)-3-(3,4-dihydroxyphenyl)-N-[2-hydroxy-2-(4-hydroxyphenyl)ethyl]prop-2-enimidic acid	HMDB0136320
632	4.68_400.2218m/z	pos	Tofisopam	HMDB0015699
633	6.47_299.1392m/z	pos	3,6-dihydroxy-4,5-diphenyl-2,3,4,5-tetrahydropyridin-2-one	HMDB0134800
634	6.62_494.1953m/z	pos	Erythroskyrin	HMDB0030464
635	9.82_481.2800m/z	neg	(-)-Salvisyriacolide	LMPR0105040002
636	9.94_337.2374m/z	neg	12-epi Leukotriene B3	45638
637	9.96_309.1700m/z	neg	methyl 8-[2-(2-formyl-vinyl)-3-hydroxy-5-oxo-cyclopentyl]-octanoate	LMFA01050151
638	1.19_203.0193m/z	pos	4-Carboxy-2-oxo-3-hexenedioate	66310
639	1.96_307.1146m/z	pos	2-Phenylethyl beta-D-glucopyranoside	HMDB0029819
640	10.69_392.2919n	neg	3b,7a-Dihydroxy-5b-cholanoic acid	HMDB0000361
641	11.25_263.2378m/z	pos	Stearolic acid	LMFA01030455
642	13.04_445.3313m/z	neg	Trihydroxycoprostanoic acid	LMST01010246
643	13.20_335.1748m/z	pos	Quebrachidine	HMDB0030389
644	13.79_779.5503n	pos	PE(19:0/20:5(5Z,8Z,11Z,14Z,17Z))	LMGP02010786
645	2.00_189.0401m/z	neg	(E)-2-Methylglutaconic acid	HMDB0002266
646	4.51_137.0603m/z	pos	Phenylacetic acid	HMDB0000209

647	4.83_241.1185m/z	neg	Glutamylisoleucine	HMDB0028822
648	5.43_256.0958n	pos	3-hydroxy-3-(3,4,5-trimethoxyphenyl)propanoic acid	HMDB0141328
649	6.40_313.1184m/z	neg	meta-O-Dealkylated flecainide	HMDB0060831
650	6.74_130.0658m/z	pos	Indole-3-carbinol	HMDB0005785
651	7.32_452.1346m/z	neg	Norfluoxetine glucuronide	HMDB0061170
652	9.60_209.1546m/z	pos	Riesling acetal	HMDB0037562
653	9.69_290.1122n	neg	N-Succinyl-L,L-2,6-diaminopimelate	HMDB0012267
654	0.79_464.1155n	pos	6-{3,5-dihydroxy-2-[3,4,5-trihydroxy-6-(hydroxymethyl)oxan-2-yl]phenoxy}-3,4,5-trihydroxyoxane-2-carboxylic acid	HMDB0132676
655	0.84_629.1142m/z	neg	Myricetin 3-(2''-galloylrhamnoside)	LMPK12112663
656	0.89_187.0712m/z	pos	gamma-Glutamylglycine	HMDB0011667
657	1.96_200.9710m/z	neg	2-(sulfooxy)acetic acid	HMDB0124940
658	10.68_335.2215m/z	pos	8-iso PGF3α	36381
659	11.13_195.1750m/z	pos	7-tridecenoic acid	LMFA01030595
660	11.23_258.2423m/z	pos	Exaltolide	HMDB0034455
661	12.12_674.5392n	pos	PE-Cer(d15:1(4E)/20:0)	LMSP03020016
662	12.22_391.2843m/z	neg	2E,8E-dodecadienoic acid	LMFA01030232
663	12.68_391.2844m/z	neg	(Z)-4-Hydroxy-6-dodecenoic acid lactone	LMFA07040067

664	12.87_503.4113m/z	pos	1α,25-Dihydroxy-21-(3-hydroxy-3-methylbutyl)vitamin D(3)	42539
665	15.33_167.0333m/z	pos	4-Formylsalicylic acid	69795
666	2.29_365.1212m/z	pos	Citrusin D	HMDB0039334
667	3.21_291.0996m/z	pos	N-gamma-Glutamyl-S-(1-propenyl)cysteine	93225
668	4.39_315.1357m/z	pos	Ginsenoyne K	HMDB0040375
669	5.05_254.9916m/z	neg	D-Glucuronic acid 1-phosphate	HMDB0003976
670	5.05_359.1236m/z	neg	Dityrosine	HMDB0006045
671	5.39_347.1633m/z	pos	ent-9-L1-PhytoP	LMFA02030003
672	5.90_424.0794m/z	pos	2-([4-(2-Chlorophenyl)-3-ethoxycarbonyl-6-hydroxymethyl-2-picolinyl]methoxyacetic acid	976
673	6.39_377.1361m/z	pos	Lactacystin	64856
674	7.88_272.2168n	pos	Dodecanoic acid, trimethylsilyl ester	HMDB0094677
675	8.76_340.2538m/z	pos	5(S),6(R)-Lipoxin A4-d5	96397
676	0.77_385.1313m/z	pos	S-Adenosylhomocysteine	HMDB0000939
677	0.86_138.0537m/z	pos	Trigonelline	HMDB0000875
678	1.22_132.0245n	pos	3-Methyl sulfolene	HMDB0059667
679	10.46_277.1435m/z	neg	(S)-Bilobanone	HMDB0036110
680	10.46_498.3186m/z	neg	PC(O-14:0/0:0)	LMGP01060007
681	12.47_318.2793n	pos	Tridihexethyl	HMDB0014648
682	13.13_463.3415m/z	neg	3a,7a-Dihydroxy-5b-cholestan-26-al	HMDB0006894
683	2.21_321.1302m/z	pos	Gynocardin	HMDB0029913

684	3.77_290.1602m/z	pos	3-Methylglutarylcarnitine	HMDB0000552
685	4.82_289.1232m/z	pos	(1aalpha,2beta,3alpha,11calpha)-1a,2,3,11c-Tetrahydro-6,11-dimethylbenzo[6,7]phenanthro[3,4-b]oxirene-2,3-diol	HMDB0062223
686	5.45_510.2250m/z	pos	2-amino-4-({1-[(carboxymethyl)-C-hydroxycarbonimidoyl]-2-[(2,3-dihydroxy-2-pentyl-1-phenylpropyl)sulfanyl]ethyl}-C-hydroxycarbonimidoyl)butanoic acid	HMDB0126538
687	5.53_766.4565n	pos	PA(20:5(5Z,8Z,11Z,14Z,17Z)/22:6(4Z,7Z,10Z,13Z,16Z,19Z))	HMDB0115221
688	6.28_414.3011m/z	pos	N-Docosahexaenoyl GABA	HMDB0062332
689	6.57_321.0580m/z	neg	N-Formyl-L-aspartate	HMDB0060495
690	7.04_647.2707m/z	pos	2',7-Dihydroxy-4'-methoxy-8-prenylflavan 2',7-diglucoside	HMDB0036400
691	7.96_195.1027m/z	neg	11-oxo-undeca-5,8-dienoic acid	LMFA01060220
692	8.12_337.2513m/z	pos	delta2-THA	LMFA01030852
693	0.80_464.2014m/z	pos	PS(12:0/0:0)	LMGP03050008
694	10.03_547.2236m/z	pos	Aerobactin	HMDB0004051
695	10.45_616.4317m/z	pos	1-(2-methoxy-18Z-pentacosenyl)-sn-glycero-3-phosphoethanolamine	LMGP02060030
696	11.37_359.2919m/z	pos	Behenolic acid	LMFA01030686

697	12.30_358.3687m/z	pos	Behenic acid	HMDB0000944
698	12.42_300.2919m/z	pos	Sphingosine	HMDB0000252
699	13.51_445.3317m/z	neg	24-keto-25dehydrocholestanol	LMST01010300
700	3.23_282.1195m/z	pos	N6-Methyladenosine	HMDB0004044
701	5.14_165.0552m/z	neg	4-Formyl-3-(formylmethyl)-4-hexenoic acid	HMDB0039650
702	5.58_302.1097m/z	pos	Flutriafol	72509
703	6.23_378.2033m/z	pos	4-(dimethylamino)-1-(2-hydroxyphenyl)-3-methyl-2-phenylbutan-2-yl propanoate	HMDB0144291
704	6.50_315.1359m/z	pos	(4E)-1-(4-hydroxy-3-methoxyphenyl)dec-4-en-3-one	HMDB0137236
705	6.53_491.1896m/z	pos	5-Hydroxy-7,4'-dimethoxy-6,8-di-C-prenylflavanone 5-O-galactoside	LMPK12140572
706	6.86_307.1299m/z	pos	(S)-17-Hydroxy-9,11,13,15-octadecatetraynoic acid	HMDB0038995
707	7.02_198.1625n	pos	Linderic acid	LMFA01030038
708	7.70_264.1378n	pos	Abscisic acid	HMDB0035140
709	8.73_182.1676n	pos	(Z,Z)-3,6-Dodecadien-1-ol	HMDB0031102
710	8.87_514.3466m/z	pos	Postin	HMDB0005772
711	9.51_688.3949m/z	pos	1-(2-methoxy-5Z,19Z-hexacosadienyl)-sn-glycero-3-phosphoserine	LMGP03060031
712	9.73_339.2700m/z	pos	6,9,12,15,18,21-Tetracosahexaenoic acid	HMDB0013025

713	9.94_616.1814m/z	pos	Pyranocyanin A	HMDB0035420
714	0.79_389.1304m/z	pos	Semilepidinoside B	HMDB0033108
715	0.89_217.0836m/z	pos	5-(4-hydroxyphenyl)pentanoic acid	HMDB0132270
716	1.10_319.1156m/z	pos	Chavicol O-beta-glucopyranoside	HMDB0041617
717	11.64_417.3382m/z	pos	24,25-hydroxyvitamin D3	220
718	11.98_325.1835m/z	neg	2-Dodecylbenzenesulfonic acid	HMDB0031031
719	13.97_539.4319m/z	neg	16-hydroxy-9Z-hexadecenoic acid	LMFA01050193
720	2.00_147.0293m/z	neg	(S)-2-Hydroxyglutarate	4151
721	2.01_310.0468m/z	pos	4-hydroxy ketorolac	HMDB0060647
722	4.13_195.1136m/z	pos	5-[2H-Pyrrol-4-(3H)-ylidenemethyl]-2-furanmethanol	HMDB0040045
723	4.17_181.0721m/z	pos	Paraxanthine	HMDB0001860
724	4.52_179.1176m/z	pos	Glycinexylidide	HMDB0060679
725	4.67_202.0891m/z	pos	(+/-)-3-[(2-methyl-3-furyl)thio]-2-butanone	HMDB0032401
726	4.71_184.0375n	pos	3,4-Dihydroxymandelic acid	HMDB0001866
727	4.72_281.0999m/z	neg	S-aminomethyldihydrolipoamide	HMDB0006239
728	4.72_334.1419m/z	pos	Glutamyltryptophan	HMDB0028830
729	5.07_360.1088m/z	pos	5,15-dihydroxy-11-methoxy-6,8,20-trioxapentacyclo[10.8.0.0^{2,9}.0^{3,7}.0^{14,19}]icosa-1(12),2(9),10,14,16,18-hexaen-13-one	HMDB0140693

730	5.26_257.1540m/z	pos	4-Hydroxy-3-polyprenylbenzoate	HMDB0060388
731	5.53_299.1364m/z	pos	Carisoprodol	HMDB0014539
732	6.01_446.2283m/z	pos	AM-toxin I	71340
733	6.75_309.0933m/z	pos	Salicin	HMDB0003546
734	6.95_299.1406m/z	pos	Falcarindiol	LMFA05000658
735	7.10_159.1023m/z	neg	5-Hydroxyvalproic acid	HMDB0013898
736	7.30_215.1285m/z	neg	Isodecenoic acid	LMFA01030032
737	9.20_225.1479m/z	pos	(3S,5R,6R,7E)-3,5,6-Trihydroxy-7-megastigmen-9-one	HMDB0038736
738	9.60_195.1382m/z	pos	(+)-7-epi--9,10-dihydrojasmonic acid	LMFA02020005
739	0.80_195.0505m/z	neg	Gluconic acid	HMDB0000625
740	0.81_379.1007m/z	neg	(S)-a-Amino-2,5-dihydro-5-oxo-4-isoxazolepropanoic acid N2-glucoside	HMDB0029404
741	10.04_195.1387m/z	neg	Cystophorene	HMDB0030944
742	10.22_283.1909m/z	neg	2-Octenoic acid	HMDB0000392
743	10.63_312.1609m/z	pos	Piperettine	HMDB0034371
744	10.85_241.1802m/z	neg	4-(2,6,6-Trimethyl-1-cyclohexenyl)-2-butanol	HMDB0036172
745	13.20_858.5977m/z	pos	PS(O-18:0/21:0)	LMGP03020037
746	2.05_391.2077m/z	pos	19(R)-hydroxy-PGE2	3819
747	2.13_132.1025m/z	pos	Beta-Leucine	HMDB0003640
748	2.28_231.0449m/z	neg	Carbimazole	HMDB0014533

749	4.67_363.1186m/z	neg	2-iodo-hexadecanoic acid	LMFA01090037
750	6.28_299.1398m/z	pos	1,8-Heptadecadiene-4,6-diyne-3,10-diol	LMFA05000584
751	6.37_171.1496m/z	pos	7,8-diaminononanoic acid	LMFA01100062
752	6.74_189.0813n	pos	Propanoic acid, 2-(methoxyimino)-, trimethylsilyl ester	HMDB0094703
753	7.10_310.0748m/z	pos	Clitocine	HMDB0033718
754	7.18_253.1076m/z	neg	12-oxo-5E,8E,10Z-dodecatrienoic acid	LMFA01060095
755	8.13_464.3006m/z	neg	Sodium glycocholate	HMDB0032596
756	8.96_583.2544m/z	neg	Coriandrone B	HMDB0037775
757	9.69_223.1339m/z	neg	13-Oxo-9,11-tridecadienoic acid	LMFA01060202
758	0.81_723.2319m/z	neg	4'-Hydroxy-5,7-dimethoxyflavanone 4'-[2-(5-Cinnamoyl)-beta-D-apiofuranosyl]glucoside	LMPK12140594
759	0.87_204.1101n	neg	N6-Acetyl-5S-hydroxy-L-lysine	HMDB0033891
760	0.91_203.1515m/z	pos	Asymmetric dimethylarginine	HMDB0001539
761	1.16_229.1211m/z	pos	Zedoarol	HMDB0038202
762	1.42_297.0438m/z	pos	{[1-hydroxy-1-(1-oxo-1H-isochromen-3-yl)butan-2-yl]oxy}sulfonic acid	HMDB0130069
763	10.18_378.2413m/z	neg	Sphingosine 1-phosphate	HMDB0000277
764	10.55_392.2918n	neg	Isohyodeoxycholic acid	5634

765	10.61_337.2755m/z	pos	MG(0:0/18:2(9Z,12Z)/0:0)	HMDB0011538
766	10.66_361.2738m/z	pos	9-deoxy-9-methylene-16,16-dimethyl -PGE2	LMFA03010059
767	10.72_315.2535m/z	neg	(9S,10S)-9,10-dihydroxyoctadecanoate	HMDB0059633
768	10.80_619.2893m/z	neg	1-Arachidonoylglycerophosphoinositol	HMDB0061690
769	11.69_251.2007m/z	pos	(1R,2R)-3-oxo-2-pentyl-cyclopentanehexanoic acid	LMFA02010014
770	12.40_324.3275m/z	pos	9R,10S-Epoxy-3Z,6Z-heneicosadiene	LMFA12000320
771	12.71_535.3998m/z	neg	(1S,2S)-3-oxo-2-pentyl-cyclopentanehexanoic acid	LMFA02010015
772	13.79_255.2333m/z	neg	Palmitic acid	HMDB0000220
773	2.01_169.1014m/z	pos	2,4,6,8-Tridecatetrayne	HMDB0030929
774	4.82_340.2614m/z	pos	N-methyl arachidonoyl amine	LMFA08020007
775	6.41_300.2181m/z	pos	3-hydroxynonanoyl carnitine	HMDB0061635
776	7.35_345.1462m/z	pos	Tyrosyl-Tyrosine	86009
777	9.06_490.2773n	pos	12-Hydroxy-13-O-D-glucuronoside-octadec-9Z-enoate	HMDB0060118
778	9.52_435.2286n	pos	Valsartan	HMDB0014323
779	9.60_208.1462n	pos	(5alpha,8beta,9beta)-5,9-Epoxy-3,6-megastigmadien-8-ol	HMDB0034672
780	9.66_361.1621m/z	neg	Thyrotropin releasing hormone	HMDB0060080

781	0.94_351.0162m/z	neg	Thelephoric acid	HMDB0030552
782	1.19_100.0522n	pos	2-Methyltetrahydrofuran-3-one	HMDB0031178
783	1.24_326.0516n	neg	2,4-Dioxotetrahydropyrimidine D-ribonucleotide	66231
784	10.35_239.1647m/z	neg	(3S,6E)-Nerolidol	HMDB0041629
785	10.48_413.2899m/z	neg	Octadecyl fumarate	HMDB0038073
786	10.67_357.2804m/z	pos	3beta-Hydroxy-chol-5-en-24-oic Acid	LMST04010201
787	11.05_495.3366n	pos	LysoPC(0:0/16:0)	HMDB0240262
788	11.80_324.2883m/z	pos	11,14,17-eicosatrienoic acid	LMFA01030159
789	12.54_335.1550m/z	pos	13-dimethylarsinoyl-tridecanoic acid	LMFA00000034
790	13.46_151.0981m/z	pos	3-Methylpyrrolo[1,2-a]pyrazine	HMDB0033172
791	4.69_393.1649m/z	pos	3-iodo-octadecanoic acid	LMFA01090040
792	4.90_347.1263m/z	pos	Abyssinone II	LMPK12140037
793	5.37_283.1074m/z	pos	Furofoline	HMDB0034319
794	5.60_419.1847m/z	pos	Artocarpin	LMPK12110898
795	5.98_425.1615m/z	neg	Garcinone A	HMDB0029509
796	6.95_253.1344m/z	neg	(+)-Setoclavine	HMDB0033428
797	7.31_471.2406m/z	neg	3,16-DIDEOXYMEXICANOLIDE-3β-DIOL	43636
798	8.20_326.2329m/z	pos	16-J1-PhytoP	LMFA02030067
799	8.78_301.1187m/z	neg	Methyl 2-aminobenzoate	HMDB0029703
800	8.89_255.1593m/z	pos	Heptanoylcholine	HMDB0013239

801	9.61_663.3594m/z	neg	Unshuoside A	HMDB0029784
802	9.66_123.0442m/z	pos	Salicylaldehyde	66352
803	9.94_361.2381m/z	pos	Macrophorin A	86989
804	0.78_241.0926m/z	neg	D-erythro-D-galacto-octitol	HMDB0029953
805	1.23_153.0300m/z	neg	Hydantoin-5-propionic acid	HMDB0001212
806	10.72_449.2537m/z	neg	3alpha-androstanediol glucuronide	LMST05010004
807	11.18_430.3072n	neg	7alpha-Hydroxy-3-oxo-4-cholestenoate	HMDB0012458
808	11.44_363.2529m/z	neg	3-undecynoic acid	LMFA01030611
809	11.75_228.2331m/z	pos	9Z,11E-Tetradecadien-1-ol	LMFA05000187
810	11.93_508.3407m/z	neg	1-heptadecanoyl-sn-glycero-3-phosphocholine	24068
811	11.93_608.3223m/z	pos	OOB-PS	LMGP20040019
812	13.99_281.2488m/z	neg	Oleic acid	HMDB0000207
813	15.33_390.2797n	pos	Pregnan-20-one, 17-(acetyloxy)-3-hydroxy-6-methyl-, (3a,5b,6a)-	1090
814	4.22_234.1333m/z	pos	Hydroxypropionylcarnitine	LMFA07070074
815	4.74_370.2211n	pos	Octaethylene glycol	HMDB0094680
816	6.56_418.1463m/z	pos	Ethyl 2-hydroxy-3-(3-indolyl)propanoate glucoside	HMDB0034873
817	6.68_229.1448m/z	pos	Trp-P-1	73018
818	7.04_476.1961m/z	pos	N1,N10-Dicoumaroylspermidine	HMDB0033469
819	7.88_370.1812n	neg	5alpha-androstane-3alpha-ol-17-one sulfate	LMST05020001

820	7.96_262.0809n	neg	gamma-Glutamylaspartic acid	HMDB0030419
821	9.63_591.3188m/z	pos	I-Urobilin	HMDB0004160
822	9.66_378.1523m/z	neg	2-(Arabinosylamino)-3-(glucosylamino)propanenitrile	HMDB0039505
823	9.66_591.3187m/z	neg	Ovalicin	HMDB0038120
824	9.90_312.1954m/z	pos	N-desethyloxybutynin	HMDB0061042
825	1.23_192.0275n	pos	2,5-didehydro-D-gluconic acid	LMFA01050471
826	1.70_151.0251m/z	neg	Glycolic acid	HMDB0000115
827	10.22_828.4146n	pos	Digitoxigenin 3-[glucosyl-(1->6)-glucosyl-(1->4)-2,6-dideoxyribohexoside]	HMDB0034321
828	10.41_178.1599m/z	pos	1-Amino-3-hydroxymethyl-5-methyl-adamantane	HMDB0060703
829	10.75_223.1343m/z	pos	Imiquimod	85419
830	10.76_501.2914n	pos	Leukotriene D4-d5	96385
831	11.02_331.2644m/z	pos	12,15-Epoxy-13,14-dimethyleicosa-12,14,16-trienoic acid	HMDB0112073
832	12.34_239.1502m/z	pos	Formetanate	72452
833	12.49_391.2846m/z	neg	2E,8Z-dodecadienoic acid	LMFA01030233
834	14.99_283.2646m/z	neg	Stearic acid	189
835	4.19_227.1770m/z	pos	(-)-3,5-Cadinadiene	HMDB0030673
836	4.19_499.0855m/z	pos	Luteolin 7-methylglucuronide	LMPK12110646

837	5.03_186.1136m/z	neg	(±)-Pelletierine	HMDB0030325
838	5.43_211.0973m/z	neg	3,4-Methyleneazelaic acid	HMDB0059744
839	6.26_161.0728m/z	pos	3-Allyl-1-cyclohexene	HMDB0061784
840	6.39_315.1355m/z	pos	Cyclandelate	HMDB0015586
841	6.49_201.1129m/z	neg	2-Hydroxy-2,6,6-trimethylcyclohexanone	HMDB0037023
842	6.60_263.9982m/z	pos	3-Mercaptolactate-cysteine disulfide	HMDB0006512
843	6.82_527.2126m/z	neg	Acetyl-N-formyl-5-methoxykynurenamine	HMDB0004259
844	8.38_540.2580n	pos	Tetrahydroaldosterone-3-glucuronide	HMDB0010357
845	8.47_646.4210m/z	pos	1-(2-methoxy-19Z-hexacosenyl)-sn-glycero-3-phosphoethanolamine	LMGP02060033
846	9.30_440.2798m/z	pos	N-docosahexaenoyl glutamic acid	LMFA08020089
847	9.74_409.1650m/z	pos	1,2-Dihydro-5-hydroxy-2-(1-hydroxy-1-methylethyl)-4-(isobutyryl)-6-phenylfurano[2,3-h][1]benzopyran-8-one	LMPK12100015
848	0.03_277.0890m/z	pos	Galactosylglycerol	HMDB0006790
849	0.80_279.1934m/z	pos	Ipomeatetrahydrofuran	HMDB0040904
850	0.88_116.0712m/z	pos	L-Proline	HMDB0000162
851	10.81_468.3318m/z	neg	Linoelaidyl carnitine	HMDB0006461
852	11.46_494.3242m/z	neg	PC(16:0/0:0)	LMGP01050018
853	11.52_344.2222m/z	pos	Piperolein B	HMDB0030340

854	11.93_636.3501m/z	neg	Allodesmosine	HMDB0040704
855	12.35_559.5223m/z	pos	5-Hexatriacontanone	HMDB0030937
856	12.41_517.3892m/z	neg	Soyasapogenol D	HMDB0034507
857	13.39_445.3318m/z	neg	astrogorgiadiol B	LMST03020242
858	14.00_811.6129n	pos	PC(20:0/18:3(6Z,9Z,12Z))	LMGP01011794
859	14.47_251.2078m/z	pos	Hypusine	HMDB0011140
860	2.00_123.0445m/z	pos	Ethyl maltol	HMDB0031735
861	2.90_151.0615m/z	pos	1-Methylhypoxanthine	HMDB0013141
862	4.60_326.1948n	pos	Heptaethylene glycol	HMDB0061835
863	5.64_516.1643m/z	pos	2-amino-4-({1-[(carboxymethyl)-C-hydroxycarbonimidoyl]-2-{[1-hydroxy-1-(4-hydroxy-3-methoxyphenyl)-3-oxobutan-2-yl]sulfanyl}ethyl}-C-hydroxycarbonimidoyl)butanoic acid	HMDB0135691
864	6.55_246.0908m/z	pos	Asparaginyl-Methionine	HMDB0028737
865	6.73_679.2611m/z	pos	Wanepimedoside A	50446
866	7.29_223.1322m/z	pos	Annuionone B	88204
867	7.79_427.2138m/z	neg	7-Hydroxytacrine	2617
868	8.17_427.2142m/z	neg	Harmaline	3350
869	8.37_301.1201m/z	neg	Acetaminophen	HMDB0001859
870	9.34_145.1223m/z	pos	Caprylic acid	LMFA01010008
871	9.66_593.3348m/z	pos	Mesobilirubinogen	HMDB0001898

872	9.70_209.1184m/z	pos	Pyridine-2-azo-p-dimethylaniline	96366
873	0.76_104.1072m/z	pos	Choline	56
874	0.81_250.0931m/z	neg	N-Acetyl-D-fucosamine	70938
875	0.87_160.0612m/z	pos	1-Methylnicotinamide	HMDB0000699
876	1.22_203.1174n	pos	L-Acetylcarnitine	HMDB0000201
877	10.13_351.1833m/z	pos	Pefloxacin	HMDB0014630
878	10.39_195.1033m/z	pos	Butyl salicylate	HMDB0040730
879	10.71_167.1436m/z	pos	2,7-dimethyl-6-nonenoic acid	LMFA01020130
880	11.11_613.2512m/z	pos	OKOHA-PA	LMGP20070025
881	11.35_313.2374m/z	neg	9,10-DiHOME	35697
882	12.35_262.2312n	pos	2-(5,8-Tetradecadienyl)cyclobutanone	HMDB0037519
883	3.76_218.1028m/z	neg	Isovalerylalanine	HMDB0000747
884	3.91_312.1322m/z	pos	Hydroxylated N-acetyl desmethyl frovatriptan	HMDB0061151
885	4.05_197.0684m/z	pos	Demethylated antipyrine	HMDB0006240
886	4.19_227.0831m/z	pos	Dihydroaceanthrylene	73104
887	4.73_267.0422m/z	pos	5,6-Dihydro-5-hydroxy-6-methyl-2H-pyran-2-one	HMDB0034270
888	4.74_307.0932m/z	pos	3,4-dimethyl-5-carboxyethyl-2-furanpentanoic acid	LMFA01150055
889	5.24_184.0378n	pos	2-(3,4,5-trihydroxyphenyl)acetic acid	HMDB0130405
890	6.35_427.1775m/z	neg	Estrone 3-glucuronide	LMST05010011
891	7.59_289.2500m/z	pos	2-Tetradecylcyclobutanone	HMDB0037517

892	7.83_329.2324m/z	neg	9,10,13-TriHOME	HMDB0004710
893	7.83_567.3161m/z	neg	6alpha-Glucuronosylhyodeoxycholate	LMST05010016
894	7.97_198.0905n	pos	2-Amino-3-methylimidazo[4,5-f]quinoline	HMDB0029706
895	8.48_353.2794m/z	pos	N6-goshuyoyl lysine	LMFA08020268
896	9.06_489.2697m/z	neg	Valyl-Glutamine	HMDB0029125
897	9.14_195.1363m/z	pos	(1R,2S,3S,4R)-p-Menthane-2,3-diol	HMDB0035723
898	9.60_137.1324m/z	pos	(+)-Limonene	HMDB0004321
899	9.72_533.2869m/z	neg	Metoprolol acid	1254
900	0.67_239.0905m/z	pos	L-Histidinol phosphate	3355
901	0.80_769.2158m/z	pos	Isorhamnetin 3-rhamnosyl-(1->2)-galactoside-7-glucoside	LMPK12112331
902	1.25_321.0153m/z	pos	3-O-Methylcoumestrol	HMDB0029584
903	1.40_229.1558m/z	pos	delta-Methylionone	HMDB0031737
904	10.35_360.9914m/z	neg	Bismuth Subsalicylate	HMDB0015408
905	10.51_478.2927m/z	neg	PC(15:1(9Z)/0:0)	LMGP01050125
906	10.66_466.2929m/z	neg	PC(14:0/0:0)	LMGP01050012
907	10.87_291.1592m/z	neg	Botrydial	LMPR0103640001
908	11.73_397.2041m/z	pos	Droperidol	HMDB0014593
909	12.30_325.1836m/z	neg	4-Dodecylbenzenesulfonic Acid	HMDB0059915
910	12.43_465.3573m/z	neg	5beta-Cholestane-3alpha,7alpha,26-triol	LMST04030020
911	14.63_138.0677m/z	pos	Cyclohexylamine	HMDB0031404
912	2.06_231.0445m/z	neg	gamma-Glutamylcysteine	HMDB0001049

913	3.42_309.1083m/z	neg	gamma-Glutamyltyrosine	HMDB0011741
914	4.25_641.0919n	pos	6-Sinapoylglucoraphenin	HMDB0038405
915	4.60_372.2605m/z	pos	Sannamycin A	71872
916	5.04_347.1622m/z	pos	2,3-Epoxymenaquinone	HMDB0060359
917	5.51_516.1645m/z	pos	2-amino-4-({1-[(carboxymethyl)-C-hydroxycarbonimidoyl]-2-{[2-hydroxy-1-(4-hydroxy-3-methoxyphenyl)-3-oxobutyl]sulfanyl}ethyl}-C-hydroxycarbonimidoyl)butanoic acid	HMDB0135692
918	6.25_462.1757m/z	neg	Dihydroisomorphine-3-glucuronide	HMDB0060820
919	6.60_213.1125m/z	neg	6-Hydroxy-2,6-dimethyl-2,7-octadien-4-one	HMDB0034670
920	7.20_601.2697m/z	pos	Fosinopril	2971
921	7.65_319.1297m/z	pos	1-(4-hydroxyphenyl)-7-phenylheptane-3,5-dione	HMDB0141090
922	8.13_527.2955m/z	neg	1,7-Diphenyl-4-hepten-3-one	87991
923	8.52_369.1730m/z	neg	5a-Dihydrotestosterone sulfate	LMST05020042
924	8.85_279.0956m/z	pos	1-(beta-D-Ribofuranosyl)-1,4-dihydronicotinamide	HMDB0011648
925	8.99_255.1596m/z	neg	8-tridecynoic acid	LMFA01030601
926	9.03_181.1586m/z	pos	Homodihydrojasmone	HMDB0031181

927	9.11_362.9890m/z	neg	2-Oxo-4-phosphonobutanoate	63606
928	9.25_237.1850m/z	pos	Capsidiol	HMDB0002352
929	9.58_172.1704m/z	pos	decanamide	LMFA08010005
930	9.82_239.0911m/z	pos	3,4-dimethyl-5-carboxyethyl-2-furanacrylic acid	LMFA01150051
931	0.88_249.1099m/z	pos	Tuberonic acid	LMFA02020007
932	10.12_424.9907m/z	neg	XDP	65583
933	10.54_333.1346m/z	neg	N-Trimethyl-2-aminoethylphosphonate	63594
934	10.66_172.1700m/z	pos	Eucalyptol	HMDB0004472
935	10.81_528.3089m/z	neg	PE(22:4(7Z,10Z,13Z,16Z)/0:0)	LMGP02050014
936	11.48_465.2861m/z	neg	Homodeoxycholic acid	LMST04070031
937	11.61_293.2109m/z	pos	Colnelenic acid	LMFA10000002
938	12.04_388.2474m/z	pos	17-phenyl-trinor-PGF2alpha amide	LMFA03010123
939	12.92_311.1559m/z	pos	β-Gal-NONOate	45168
940	14.26_281.2479m/z	pos	Tetrabutylammonium	85164
941	2.00_400.1018n	neg	Sinapinic acid-O-glucuronide isomer	HMDB0060019
942	2.13_233.0473m/z	pos	9-Aminoacridine	43514
943	2.99_346.1256n	pos	Deutzioside	LMPR0102070036
944	5.22_315.0562m/z	pos	2-hydroxyflutamide	HMDB0060949
945	5.87_277.1438m/z	pos	Acetylpterosin C	HMDB0030764
946	6.00_383.1526m/z	neg	3b,16a-Dihydroxyandrostenone sulfate	LMST05020022
947	6.06_355.1265m/z	pos	S-(Hydroxymethyl)glutathione	HMDB0004662

948	6.08_299.1397m/z	pos	2,9-heptadecadien-4,6-diyn-1,8-diol	LMFA05000022
949	8.09_624.3389m/z	neg	(3a,5b,7b)-24-[(carboxymethyl)amino]-7-hydroxy-24-oxocholan-3-yl-b-D-glucopyranosiduronic acid,	6724
950	8.55_395.1884m/z	neg	9alpha-Fluoro-11beta,16alpha,17alpha,21-tetrahydroxypregn-4-ene-3,20-dione	70225
951	8.73_329.1414m/z	pos	Azatadine	HMDB0014857
952	9.01_237.1849m/z	pos	(2E,6E)-1-Hydroxy-2,6,10-farnesatrien-9-one	87259
953	9.30_432.2413m/z	pos	Undecylprodigiosin	69260
954	9.66_329.1519m/z	neg	3-Pyridinebutanoic acid	HMDB0001007
955	0.59_168.0293m/z	pos	Quinolinic acid	HMDB0000232
956	0.59_380.1007m/z	pos	fosphenytoin	4050
957	0.62_324.1475n	pos	p-Hydroxyphenylbutazone	1840
958	0.76_257.1047n	pos	Glycerophosphocholine	HMDB0000086
959	0.80_162.1134m/z	pos	L-Carnitine	HMDB0000062
960	0.81_344.1310n	neg	Melibiitol	HMDB0006791
961	0.83_114.0655m/z	pos	Creatinine	HMDB0000562
962	0.85_290.0684n	neg	TEPP	72768
963	0.92_230.0961m/z	pos	Ergothioneine	HMDB0003045
964	10.05_315.2787n	pos	(4OH,8Z,t18:1) sphingosine	53909

965	10.16_226.1214n	pos	epi-4'-hydroxyjasmonic acid	LMFA02020008
966	10.21_271.1908m/z	neg	5-Tetradecenoic acid	HMDB0000499
967	10.59_365.1457m/z	pos	Pyriminobac	72651
968	10.74_595.2892m/z	neg	PI(18:2(9Z,12Z)/0:0)	LMGP06050010
969	10.75_305.1383m/z	pos	8-hydroxy-2-(hydroxymethyl)-2-(4-methylpent-3-en-1-yl)-2H-chromene-5-carboxylic acid	HMDB0134718
970	10.84_314.2458n	pos	18-hydroxy-9R,10S-epoxy-stearic acid	LMFA02000003
971	10.91_608.3187m/z	neg	PHHdiA-PE	LMGP20020017
972	10.91_625.3079m/z	neg	HOMIDIUM	44366
973	10.98_419.2551m/z	pos	2-Hydroxy-4-oxo-5,12-heneicosadien-1-yl acetate	HMDB0039403
974	11.22_171.1015m/z	pos	1,4-Ipomeadiol	87001
975	11.25_325.2374m/z	neg	Bovinic acid	HMDB0003797
976	11.26_634.3343m/z	neg	Pentasine	HMDB0029803
977	11.26_714.3451m/z	pos	Fumonisin C4	LMSP01080030
978	11.61_334.2518n	pos	(±)5(6)-EET methyl ester	62931
979	11.75_464.3517n	pos	Hongguanggenin	LMST01080073
980	11.87_377.2696m/z	neg	3-Hydroxy-1-phenyl-1-hexadecanone	HMDB0035677
981	12.07_473.3643m/z	pos	Maslinic Acid	44861
982	12.85_327.2327m/z	neg	DHA	LMFA01030185
983	14.24_310.3121m/z	pos	Stearoylethanolamide	HMDB0013078
984	15.00_138.0665m/z	pos	Isoniazid	848

985	15.22_401.3440m/z	pos	Calcidiol	HMDB0003550
986	15.56_187.1482m/z	pos	7-Ethyl-5,6-dihydro-1,4-dimethylazulene	91006
987	4.18_142.0657m/z	neg	Tryptophanol	HMDB0003447
988	4.35_291.1352m/z	pos	(E)-octadeca-15,17-dien-9,11,13-triynoic acid	LMFA01031146
989	4.75_179.0586n	pos	N-benzoylglycinate	HMDB0062583
990	4.78_263.1432m/z	pos	Alnustone	HMDB0031664
991	5.22_212.1649m/z	pos	Perillyl acetate	HMDB0037231
992	5.30_439.1595m/z	neg	Gibberellin A43	HMDB0035046
993	5.47_381.1172m/z	neg	3,4,5-trihydroxy-6-{[4-hydroxy-5-(3-methoxyphenyl)pentanoyl]oxy}oxane-2-carboxylic acid	HMDB0127784
994	5.57_273.1687m/z	pos	1-Octen-3-yl glucoside	HMDB0032959
995	6.19_145.1224m/z	pos	Hexyl acetate	LMFA07010184
996	7.08_384.3009m/z	pos	Bepridil	HMDB0015374
997	7.59_310.1622m/z	neg	Arginyl-Histidine	HMDB0028711
998	7.93_567.3162m/z	neg	(3a,5b,7a)-23-Carboxy-7-hydroxy-24-norcholan-3-yl-b-D-Glucopyranosiduronic acid	HMDB0002430
999	7.97_502.1818n	pos	Limonexic acid	HMDB0036075
1000	9.64_376.2252m/z	neg	PC(O-10:1(9E)/0:0)	LMGP01060027
1001	9.72_257.1752m/z	neg	4-(3-Hydroxybutyl)-3,3,5-trimethylcyclohexanone	HMDB0039805

1002	9.93_942.1863m/z	pos	7-Methyl-3-oxo-6-octenoyl-CoA	HMDB0060421
1003	9.96_391.2844m/z	neg	Hyodeoxycholic acid	5701
1004	0.67_285.0245m/z	pos	4-(1,2-dithiin-3-yl)-2-(prop-1-en-2-yloxy)but-3-yn-1-yl acetate	LMFA12000363
1005	0.87_132.0672m/z	pos	6-Methyladenine	HMDB0002099
1006	0.92_455.1513m/z	neg	Trioxsalen	HMDB0015575
1007	1.22_269.1262m/z	pos	Hypaphorine	67522
1008	1.24_203.0138m/z	neg	aluminum acetate	4000
1009	10.22_494.2575m/z	pos	Epothilone A	LMPK04000040
1010	10.28_475.3267m/z	neg	Sorbitan stearate	LMFA07011020
1011	10.35_284.2946m/z	pos	Stearamide	LMFA08010003
1012	10.43_335.2212m/z	pos	PGJ2	LMFA03010019
1013	10.58_487.3269m/z	neg	Menthyl ethylene glycol carbonate	HMDB0036141
1014	10.64_335.1700m/z	pos	1,5-Dibutyl methyl hydroxycitrate	HMDB0040461
1015	10.87_452.2773m/z	neg	LysoPE(0:0/16:0)	HMDB0011473
1016	10.97_171.1012m/z	pos	Azelaic acid	LMFA01170054
1017	11.08_527.1929m/z	neg	1'-Acetoxyeugenol acetate	HMDB0036544
1018	11.28_464.3133m/z	neg	N-oleoyl histidine	LMFA08020133
1019	11.38_267.1951m/z	pos	4-Hydroxy-3-methoxy-2,10-bisaboladien-9-one	89616
1020	11.46_399.2540m/z	pos	Colupox b	HMDB0039598
1021	11.68_205.0871m/z	pos	Mipafox	72759
1022	11.84_251.2011m/z	pos	4,7,10-hexadecatrienoic acid	LMFA01030134

1023	12.19_483.3679m/z	neg	14-hydroxy-5Z-tetradecenoic acid	LMFA01050181
1024	12.30_223.2066m/z	pos	9-Pentadecenoic acid	HMDB0029765
1025	12.35_151.0977m/z	pos	2-Aminobenzimidazole	68671
1026	13.12_563.5535m/z	pos	dirithromycin	3988
1027	13.34_503.4124m/z	pos	Lyso-PAF C-16-d4	96524
1028	14.98_129.1274m/z	pos	2,5-Diethyltetrahydrofuran	86355
1029	15.71_129.1274m/z	pos	Caprylaldehyde	LMFA06000028
1030	2.00_200.0560m/z	neg	Ethosuximide M5	2600
1031	2.14_246.0562m/z	neg	Tinidazole	HMDB0015047
1032	2.14_479.2292m/z	neg	11-Deacetylvaltrate 11-(3-hydroxy-3-methylbutanoate)	HMDB0033654
1033	2.83_279.1008m/z	pos	Glutamylmethionine	HMDB0028825
1034	3.01_351.1316m/z	neg	N-Benzooxazol-2-yl-guanidine	96363
1035	3.81_195.1235m/z	pos	Tetraethylene glycol	HMDB0094708
1036	4.49_259.0130m/z	neg	{[2,3,4-trihydroxy-5-(hydroxymethyl)oxolan-2-yl]methoxy}sulfonic acid	HMDB0125613
1037	5.32_109.1011m/z	pos	(E)-2-Octenal	75309
1038	5.46_494.1956m/z	pos	20-Hydroxy-leukotriene E4	LMFA03020062
1039	5.81_439.1602m/z	neg	p-Hydroxyl-ethotoin	HMDB0061174
1040	6.89_365.2432m/z	pos	S-4-benzyl-3-((S)-3-hydroxy-2,2-dimethyloctanoyl)oxazolidin-2-one	65461

1041	6.93_505.2641m/z	neg	(3b,9R)-5-Megastigmene-3,9-diol 9-[apiosyl-(1->6)-glucoside]	LMFA13010056
1042	7.48_630.2099m/z	pos	7-Hydroxyfluphenazine glucuronide	2779
1043	7.96_517.1391m/z	neg	4-O-Methyl-a-D-glucosyl-(1->2)-b-D-xylosyl-(1->4)-D-xylose	HMDB0039742
1044	7.97_311.0731m/z	pos	2-(hydroxymethyl)-6-(2,4,6-trihydroxyphenyl)oxane-3,4,5-triol	HMDB0132663
1045	8.02_511.2896m/z	neg	(25S)-11alpha,20,26-trihydroxyecdysone	LMST01010158
1046	8.77_299.1393m/z	pos	INDOPROFEN	44065
1047	9.24_270.2068m/z	neg	Tridecanoylglycine	HMDB0013317
1048	9.51_631.2404m/z	neg	3,4,5-trihydroxy-6-(5-hydroxy-4-{2-hydroxy-3-[4-hydroxy-3-(3-methylbut-2-en-1-yl)phenyl]propanoyl}-2-(3-methylbut-2-en-1-yl)phenoxy)oxane-2-carboxylic acid	HMDB0133199
1049	9.78_215.1649m/z	neg	12-Hydroxydodecanoic acid	HMDB0002059
1050	9.86_201.0545m/z	pos	2,2',3-Trihydroxydiphenylether	66734
1051	0.59_163.9875m/z	pos	Ciliatine	HMDB0011747
1052	0.61_277.0963m/z	pos	Rosoxacin	HMDB0014955
1053	0.62_175.0225m/z	pos	Oxypurinol	HMDB0000786
1054	0.64_247.0867m/z	pos	Lamivudine	HMDB0014847

1055	0.73_169.9851m/z	pos	Iminoaspartic acid	LMFA01170110
1056	0.75_479.0588m/z	pos	3,5-Dihydroxyphenyl 1-O-(6-O-galloyl-beta-D-glucopyranoside)	93891
1057	0.82_252.1071m/z	pos	Muramic acid	3381
1058	0.97_203.0522m/z	pos	myo-Inositol	144
1059	1.17_116.0718m/z	neg	Pyrrolidine	HMDB0031641
1060	1.22_219.0191m/z	pos	Methionine sulfoximine	HMDB0029430
1061	1.43_268.0096m/z	pos	Flonicamid	72269
1062	10.37_295.1908m/z	neg	2S-hydroxy-3-(10Z-tetradecenoyloxy)-propanoic acid	LMFA07010692
1063	10.41_437.1964m/z	pos	2-(2,4-dihydroxy-5-methoxyphenyl)-3-(3,7-dimethylocta-2,6-dien-1-yl)-7-hydroxy-4H-chromen-4-one	HMDB0129645
1064	10.61_619.2893m/z	neg	N-Desmethylcitalopram	HMDB0014021
1065	10.93_526.2933m/z	neg	LysoPE(0:0/22:5(7Z,10Z,13Z,16Z,19Z))	HMDB0011495
1066	11.02_452.2778m/z	neg	PE(0:0/16:0)	LMGP02050036
1067	11.89_183.1741m/z	pos	Cyclododecanone	HMDB0031341
1068	12.03_365.2684m/z	neg	5alpha-Pregnane-3alpha,20alpha-diol	HMDB0060409
1069	12.34_457.3524m/z	neg	N1,N8-Diacetylspermidine	HMDB0041947
1070	12.70_333.3020m/z	pos	Panamine	68571
1071	15.74_137.1329m/z	pos	(+)-Comphene	LMPR0102120011
1072	2.00_450.1123m/z	pos	Galβ1-4Glcβ-Sp	3607

1073	2.12_448.2114m/z	pos	2,2-Dimethyl-3,4-bis(4-methoxyphenyl)-2H-1-benzopyran-7-ol acetate	70521
1074	3.42_311.1258m/z	pos	4'-Methoxymucidin	HMDB0030019
1075	4.07_399.2399m/z	pos	Forasartan	HMDB0015434
1076	4.11_301.0924m/z	neg	2-Hydroxyadenine	HMDB0000403
1077	4.93_289.1227m/z	pos	R-Soterenol	69207
1078	5.44_299.1396m/z	pos	Annolobine	67612
1079	6.19_229.1443m/z	pos	3-Hydroxydodecanedioic acid	HMDB0000413
1080	6.43_591.3187m/z	pos	Boc-Phe(NMe)-Pro-Phe(NMe)-Gly-OMe	65475
1081	7.32_487.1015m/z	neg	Citicoline	HMDB0001413
1082	8.77_195.1036m/z	pos	2-Amino-3,4-dimethylimidazo[4,5-f]quinoline	HMDB0029707
1083	9.40_274.9767m/z	neg	Sodium citrate	69553
1084	9.40_372.2681n	pos	3alpha-Hydroxy-5beta-chola-8,14-dien-24-oic Acid	LMST04010299
1085	9.52_428.2083m/z	pos	3-Isomangostin	HMDB0041521
1086	0.66_275.0554m/z	neg	3,4-Dihydroxybenzaldehyde	HMDB0059965
1087	0.81_162.0522n	neg	3-Hydroxymethylglutaric acid	HMDB0000355
1088	0.85_391.0090m/z	neg	3,3'-Biflaviolin	72005
1089	1.16_199.0235m/z	pos	5-Carboxymethyl-2-hydroxymuconate	63509

1090	1.25_307.0266m/z	pos	{5-[(E)-2-(3,5-dihydroxyphenyl)ethenyl]-2-hydroxyphenyl}oxidanesulfonic acid	HMDB0128521
1091	10.23_446.2570m/z	pos	RO 40-5966 (Methylmibefradil Metabolite)	1280
1092	10.27_453.2845n	neg	PC(13:0/0:0)	LMGP01050001
1093	10.64_548.2879m/z	pos	Ergosine	67481
1094	10.67_241.1802m/z	neg	Ipurolic acid	LMFA01050081
1095	10.90_445.3346m/z	pos	24,25-epoxy-cholesterol(d6)	41655
1096	11.41_171.1012m/z	pos	Nonate	HMDB0011717
1097	11.68_579.2970m/z	pos	Salannin	67197
1098	11.69_141.1138m/z	pos	Metenamine	HMDB0029598
1099	12.32_293.2469m/z	pos	Androstan-3α,17β-diol	63341
1100	12.40_417.2997m/z	neg	(+)-Dysideapalaunic acid	LMPR0105040001
1101	13.20_785.5960n	pos	PC(18:1(14Z)/18:1(14Z))	LMGP01010853
1102	15.95_109.1015m/z	pos	(3xi,5Z)-1,5-Octadien-3-ol	HMDB0030966
1103	4.18_265.0267m/z	pos	Anthralin	1030
1104	4.18_429.1533m/z	neg	Kinetin	HMDB0012245
1105	4.47_294.0946n	pos	6-O-(3R,4-dihydroxy-2-methylene-butanoyl)-beta-D-glucopyranose	LMSL05000003
1106	4.84_245.1847m/z	pos	Leucyl-Leucine	HMDB0028933
1107	5.87_494.1960m/z	pos	Lipoxin E4	LMFA03040006
1108	5.97_255.1135m/z	pos	Nepafenac	HMDB0015678
1109	6.52_369.1731m/z	neg	17β-Hydroxy-5α-androstan-3-one sulfate	3565

1110	6.75_337.14 10m/z	neg	Fusarochromanone	HMDB0033514
1111	7.59_334.23 88m/z	pos	Cafestol	HMDB0035710
1112	7.86_274.18 83m/z	pos	Valyl-Arginine	HMDB0029121
1113	7.86_591.25 66m/z	pos	Bipindogulomethyloside	HMDB0030623
1114	7.88_437.15 97m/z	neg	PF-3845	45514
1115	7.88_450.16 49m/z	pos	PE(6:0/6:0)[U]	40503
1116	8.01_144.99 15m/z	neg	Tetrafluoroethylene	73011
1117	8.05_285.12 50m/z	neg	Schradan	72766
1118	8.59_181.15 81m/z	pos	2-lauroleic acid	LMFA01030037
1119	8.73_147.11 68m/z	pos	2-Methyl-3-(2-pentenyl)-2-cyclopenten-1-one	HMDB0037294
1120	9.44_237.15 04m/z	pos	Ferimzone	72381
1121	9.45_253.14 39m/z	neg	C75	44901
1122	9.52_280.09 54n	pos	Gravolenic acid	89591
1123	9.79_317.11 79m/z	pos	5-Methoxy-2,2-dimethyl-8-phenyl-2H,6H-benzo[1,2-b:5,4-b']dipyran-6-one	48615
1124	9.90_296.22 22m/z	pos	9-Decenoylcarnitine	LMFA07070048
1125	0.67_301.14 24m/z	pos	2-[5-(3,3-dimethyloxiran-2-yl)-3-methylpent-2-en-1-yl]benzene-1,3,5-triol	HMDB0133086
1126	0.80_165.03 99m/z	neg	L-Lyxonate	5524
1127	0.81_411.10 97m/z	neg	EUGENITOL	44043
1128	0.81_423.04 71m/z	neg	Propane, 1,1,1,3,3-pentafluoro-2-(fluoromethoxy)-3-methoxy-(Compound B)	2425

1129	0.87_293.1028m/z	pos	(E)-1-O-Cinnamoyl-beta-D-glucose	HMDB0030293
1130	0.88_256.0139m/z	neg	Phosphophosphinate	69259
1131	1.21_366.0408n	neg	{3-[3-(3,4-dihydroxy-2-methoxyphenyl)prop-2-enoyl]phenyl}oxidanesulfonic acid	HMDB0126072
1132	1.21_430.2307m/z	pos	Deoxypyridinoline	HMDB0000569
1133	1.30_180.9152m/z	neg	3-Iodopropanoic acid	HMDB0031252
1134	10.35_380.2563m/z	neg	Sphinganine 1-phosphate	HMDB0001383
1135	10.66_543.3048m/z	neg	1,1,2-Triphenylpropane	69935
1136	10.84_517.2442m/z	neg	Lucidenic acid K	HMDB0035890
1137	11.28_466.3283m/z	neg	PC(O-15:0/0:0)	LMGP01060009
1138	11.35_592.3603m/z	neg	PC(20:2(11Z,14Z)/0:0)	LMGP01050132
1139	11.50_594.3778m/z	pos	POV-PC	45370
1140	11.56_296.2360n	pos	9(R)-HODE	45660
1141	11.56_433.2349m/z	neg	N-Desmethylaminopyrine	85278
1142	11.66_580.3613m/z	neg	LysoPE(0:0/22:1(13Z))	HMDB0011491
1143	11.76_703.5807m/z	pos	GlcCer(d15:1/18:0)	LMSP0501AA46
1144	11.87_330.2776n	pos	MG(16:0/0:0/0:0)	LMGL01010009
1145	12.57_455.2478m/z	neg	Sulfolithocholic acid	HMDB0000907
1146	12.67_227.2017m/z	neg	(Z)-4-Hepten-1-ol	LMFA05000571
1147	13.18_705.5909m/z	pos	SM(d18:0/16:0)	LMSP03010004
1148	13.79_856.5861m/z	pos	PC(20:5(5Z,8Z,11Z,14Z,17Z)/22:4(7Z,10Z,13Z,16Z))	59961

1149	2.01_231.0857m/z	pos	8-METHYLCAFFEINE	84980
1150	2.08_241.1576m/z	pos	5,8,11-heptadecatriynoic acid	LMFA01030479
1151	2.31_257.1146m/z	pos	(2Z)-3-hydroxy-2-(phenylmethylidene)heptanoic acid	HMDB0133171
1152	4.18_261.0403m/z	neg	4-hydroxy-7-methyl-5H-furo[2,3-g]isochromen-5-one	HMDB0138947
1153	4.55_275.1480m/z	pos	Hexazinone	68694
1154	4.59_245.0115m/z	neg	{[1-(2-hydroxyphenyl)-3-oxopropan-2-yl]oxy}sulfonic acid	HMDB0134069
1155	4.78_430.2665m/z	pos	Sisomicin	43332
1156	5.24_326.2551m/z	pos	1,2,3-Tris(1-ethoxyethoxy)propane	HMDB0037162
1157	6.06_229.0984m/z	pos	trans,trans-1,4-Diphenyl-1,3-butadiene	70315
1158	6.06_299.1014m/z	neg	7-Methylhypoxanthine	HMDB0003162
1159	6.15_238.9964m/z	neg	2-Keto-3-deoxy-6-phosphogluconic acid	HMDB0001376
1160	6.55_244.0949m/z	pos	Carbofuran	HMDB0031770
1161	7.34_413.1410m/z	pos	Todatriol glucoside	HMDB0037260
1162	7.90_315.1365m/z	pos	(1E)-1-(4-hydroxy-3-methoxyphenyl)dec-1-en-3-one	HMDB0137237
1163	7.97_235.0648m/z	pos	Eflornithine	2531
1164	8.54_291.2173m/z	pos	Glycerol 1-(5-hydroxydodecanoate)	94704

1165	8.56_260.2585m/z	pos	Pentadecylic acid	LMFA01010015
1166	8.60_285.2585m/z	pos	18-fluoro-octadecanoic acid	LMFA01090045
1167	8.98_236.9805m/z	neg	L-Ascorbate 6-phosphate	63182
1168	9.16_567.3162m/z	neg	(3alpha,5beta,7alpha)-23-Carboxy-7-hydroxy-24-norcholan-3-yl-beta-D-Glucopyranosiduronic acid	LMST05010028
1169	9.26_255.1595m/z	neg	Cyclohexanecarboxylic acid	HMDB0031342
1170	9.52_267.1251m/z	pos	Nevirapine	HMDB0014383
1171	9.65_617.2627m/z	pos	BEBEERINE	44247
1172	9.88_404.2104m/z	pos	AKB48	85060
1173	0.78_219.0458m/z	neg	3-Hydroxyflavone	HMDB0031816
1174	0.78_410.9428m/z	neg	Etidronic acid	2629
1175	1.15_363.1039m/z	pos	trans-isoeugenol-O-glucuronide	HMDB0060021
1176	1.43_360.0357m/z	pos	2-{[hydroxy({3,4,8,9,10-pentahydroxy-6-oxo-6H-benzo[c]chromen-1-yl})methylidene]amino}acetic acid	HMDB0126665
1177	10.05_583.2620m/z	pos	Geldanamycin	45547
1178	10.39_151.1119m/z	pos	Cuminyl alcohol	HMDB0031817
1179	10.68_268.1009m/z	pos	2-[(5-Methylsulfinyl)-4-penten-2-ynylidene]-1,6-dioxaspiro[4.4]non-3-ene	HMDB0032670
1180	11.02_313.2745m/z	pos	Ricinoleic Acid methyl ester	45190

1181	11.02_502.2928m/z	neg	LysoPE(0:0/20:3(11Z,14Z,17Z))	HMDB0011484
1182	11.05_276.1366m/z	pos	Nefopam	1482
1183	11.05_771.4731m/z	pos	PA(P-18:0/22:6(4Z,7Z,10Z,13Z,16Z,19Z))	LMGP10030090
1184	11.36_297.2429m/z	neg	Ricinoleic acid	35485
1185	12.27_329.1620m/z	pos	Verapamil metabolite D-617	HMDB0013962
1186	13.20_757.5387n	pos	1-(8-[5]-ladderane-octanyl)-2-(8-[3]-ladderane-octanyl)-sn-glycerophosphoethanolamine	LMGP02040014
1187	13.60_865.5346m/z	pos	PG(18:0/22:4(7Z,10Z,13Z,16Z))	LMGP04010883
1188	15.46_360.3258m/z	pos	5-Decanoyl-2-nonylpyridine	HMDB0035516
1189	15.63_187.1483m/z	pos	2,4-di-tert-butylphenol	85271
1190	15.92_279.0956m/z	pos	5-Ethyl-5-(1-methyl-3-carboxypropyl)barbituric acid	1764
1191	3.00_382.1888m/z	pos	dibucaine	3979
1192	5.28_571.1593m/z	pos	3,4,5-trihydroxy-6-{[4-hydroxy-7-methoxy-2,2-dimethyl-6-(3-phenylpropanoyl)-3,4-dihydro-2H-1-benzopyran-5-yl]oxy}oxane-2-carboxylic acid	HMDB0125834
1193	5.44_303.1763m/z	pos	12alpha-Fluoro-11beta,17beta-dihydroxyandrosta-1,4-dien-3-one	70655
1194	5.97_378.2030m/z	pos	Pipercide	HMDB0033449
1195	6.06_241.0962m/z	pos	Trisphaeridine	69349
1196	6.09_315.1363m/z	pos	[8]-Shogaol	HMDB0031463
1197	6.35_331.1286m/z	neg	Flecainide meta-O-dealkylated	2702

1198	6.36_250.0224n	pos	(S)-Spirobrassinin	HMDB0035974
1199	6.84_361.2032m/z	pos	Aprindine	HMDB0015498
1200	6.99_195.1372m/z	pos	Traumatin	LMFA01060093
1201	7.02_294.1836n	pos	Gingerol	HMDB0005783
1202	7.34_213.1605m/z	pos	2-Methyl-2-phenyl-undecane	HMDB0013820
1203	7.45_203.0014m/z	pos	4-Sulfobenzoate	65735
1204	7.56_452.2304n	pos	Hoechst 33342	68903
1205	7.88_454.1502m/z	neg	Acetylcorynoline	73548
1206	7.88_507.1646m/z	pos	8-(2-{[(2E)-3-(3,4-dihydroxyphenyl)prop-2-enoyl]oxy}propan-2-yl)-2-oxo-2H,8H,9H-furo[2,3-h]chromen-9-yl 3-methylbut-2-enoate	HMDB0128938
1207	8.28_471.2403m/z	neg	Oxatomide	HMDB0240225
1208	9.20_734.2034m/z	pos	Pelargonidin 3-(2G-xylosylrutinoside)	LMPK12010023
1209	9.60_245.0801m/z	pos	(2E,11Z)-Wyerone acid	HMDB0030946
1210	9.66_236.1051m/z	neg	HC Blue No.1	72968
1211	0.61_219.0557m/z	pos	11-Hydroxycanthin-6-one	67521
1212	0.66_259.0778m/z	neg	4-Fluorocyclohexadiene-cis,cis-1,2-diol	71224
1213	0.76_180.0638n	pos	D-Mannose	HMDB0000169
1214	0.77_162.0522n	pos	2-Hydroxy-2-ethylsuccinic acid	HMDB0059758
1215	0.78_277.0685m/z	neg	5-Hydroxypyrazinamide	HMDB0001978

1216	0.81_533.1882m/z	neg	Isosyringinoside	95276
1217	1.00_367.1221m/z	pos	Maltitol	HMDB0002928
1218	1.21_189.1247m/z	pos	(-)-Nopol	86655
1219	1.22_102.0551m/z	pos	L-Threonine	HMDB0000167
1220	1.22_309.0523m/z	pos	7-hydroxy-6-methoxy-3-phenyl-3,4-dihydro-2H-1-benzopyran-4-one	HMDB0132860
1221	1.22_351.1118m/z	pos	Biapenem	68951
1222	1.23_398.0872m/z	neg	Acenocoumarol	HMDB0015487
1223	10.20_295.1930m/z	pos	Sodium Tetradecyl Sulfate	HMDB0014607
1224	10.41_473.2683m/z	pos	Usambarine	67552
1225	10.56_315.2534m/z	neg	Pelargonic acid	LMFA01010009
1226	10.75_253.1802m/z	pos	1-Hydroxyepiacorone	HMDB0030918
1227	10.87_101.0961m/z	pos	4-Methylpentanal	HMDB0001318
1228	11.02_309.2416m/z	pos	8-methoxy-13-hydroxy-9,11-octadecadienoic acid	LMFA01080002
1229	11.20_313.2373m/z	neg	11-Cyclohexylundecanoic acid	LMFA01140007
1230	11.50_213.1481m/z	pos	10-Hydroxy-3,7-dimethyl-2E,6E-decadienoic acid	LMFA01030787
1231	11.54_441.2506m/z	pos	Idanpramine	71884
1232	12.06_493.3880m/z	pos	25-acetoxy-ergosta-3beta,5alpha,6beta-triol	LMST01031059
1233	12.08_302.2468n	pos	MG(0:0/14:0/0:0)	HMDB0011530
1234	12.97_465.3575m/z	neg	5alpha-cholestan-3alpha,12alpha,16alpha-triol	LMST01010335

1235	14.21_913.5364m/z	pos	PG(22:2(13Z,16Z)/22:6(4Z,7Z,10Z,13Z,16Z,19Z))	LMGP04010786
1236	14.97_107.0497m/z	pos	4-Methylcatechol	64945
1237	2.00_383.1199m/z	neg	Acetyl-maltose	65717
1238	2.13_261.0523m/z	neg	5-Fluorouridine	71273
1239	2.28_191.0139m/z	neg	1-Propene, 1,3,3,3-tetrafluoro-2-(fluoromethoxy)-1-methoxy-, (Z)- (Compound C)	2426
1240	2.83_277.0856m/z	neg	gamma-Glutamylmethionine	HMDB0034367
1241	4.13_302.2010m/z	pos	Latrepirdine	HMDB0240240
1242	4.18_333.0394m/z	neg	4,7-dihydroxy-3-[(4-hydroxy-2-oxo-2H-chromen-3-yl)methyl]-2H-chromen-2-one	HMDB0143111
1243	4.29_196.1099n	pos	4-(3-hydroxybutyl)-2-methoxyphenol	HMDB0135672
1244	4.52_379.2074m/z	pos	Pentosidine	HMDB0003933
1245	4.64_188.0926m/z	neg	Proline betaine	HMDB0004827
1246	5.12_339.0017m/z	neg	Bis(4-nitrophenyl)phosphate	66043
1247	5.62_275.2350m/z	pos	13-Heptadecyn-1-ol	HMDB0013813
1248	5.85_285.0336m/z	pos	Mannitol 1-phosphate	HMDB0001530
1249	6.25_353.0314m/z	neg	Repenone	LMPK12060079
1250	6.73_415.2142m/z	neg	Isoelemicin	HMDB0029867
1251	6.74_223.1704m/z	pos	7-oxo-11Z-Tetradecenoic acid	LMFA01060187
1252	6.77_371.1878m/z	neg	16-phenyl-tetranor-PGE2	36137
1253	7.70_186.2224m/z	pos	1-Dodecene	HMDB0059874

1254	7.87_358.9939m/z	neg	2-(Fluoromethoxy)-1,1,3,3,3-pentafluoro-1-propene (Compound A)	2424
1255	8.62_111.0440m/z	pos	Catechol	HMDB0000957
1256	9.62_309.1699m/z	neg	Valdiate	HMDB0040980
1257	9.75_195.1039m/z	pos	Chrysoidine free base	70207
1258	9.77_181.1593m/z	pos	Methyl 10-undecenoate	HMDB0029585
1259	9.78_366.2439m/z	pos	9-Fluoro-17beta-hydroxy-6alpha,17-dimethylandrost-4-ene-3,11-dione	70584
1260	9.84_288.2937m/z	pos	Oleic acid (d5)	45774
1261	0.62_445.3660m/z	pos	3-beta-Hydroxy-4-beta-methyl-5-alpha-cholest-7-ene-4-alpha-carboxylate	HMDB0011662
1262	0.66_261.0401m/z	neg	ISOBERGAPTENE	43733
1263	0.69_145.0976m/z	neg	3-Aminopropionaldehyde	HMDB0001106
1264	0.78_217.0486m/z	neg	Sodium (±)-2-(4-methoxyphenoxy)propionate	94786
1265	0.78_399.0061m/z	neg	Sevoflurane	2422
1266	0.80_279.0037m/z	pos	2-C-Methyl-D-erythritol 2,4-cyclodiphosphate	64016
1267	0.96_157.0105m/z	pos	L-Malic acid	HMDB0000156
1268	1.22_414.9869m/z	pos	5-Iodotubercidin	45255
1269	10.23_295.1196m/z	pos	Methionyl-Glutamate	85874
1270	10.30_335.2222m/z	pos	5-trans-PGE2	LMFA03010148
1271	10.77_552.3103m/z	neg	LysoPE(0:0/24:6(6Z,9Z,12Z,15Z,18Z,21Z))	HMDB0011499

1272	10.94_333.2040m/z	neg	2-Dimethylamino-5,6-dimethylpyrimidin-4-ol	68685
1273	11.11_303.2339m/z	pos	11,12-Epoxyeicosatrienoic acid	HMDB0004673
1274	11.17_375.2460m/z	pos	17-phenyl trinor Prostaglandin F2α-d4	96575
1275	11.24_297.2427m/z	neg	cis-9,10-Epoxystearic acid	36008
1276	11.56_459.2582m/z	pos	[6]-Gingerdiol 4'-O-beta-D-glucopyranoside	HMDB0036122
1277	11.64_337.1672m/z	pos	Norfloxacin	HMDB0015192
1278	11.67_461.3258m/z	neg	1alpha,25-dihydroxyvitamin D3	LMST03020258
1279	11.68_297.2440m/z	pos	12,13-DiHOME(9)	LMFA02000164
1280	11.89_304.2613n	pos	1-O-(2R-hydroxy-tetradecyl)-sn-glycerol	LMGL01020061
1281	11.91_646.2843m/z	pos	OXONITINE	43770
1282	12.16_336.3278m/z	pos	Anandamide (20:l, n-9)	LMFA08040010
1283	12.18_732.5601m/z	pos	Araliacerebroside	HMDB0033621
1284	13.28_899.5018m/z	pos	PI(16:0/20:3(5Z,8Z,11Z))	61172
1285	15.99_153.0460m/z	pos	AMT	64865
1286	2.35_249.0583m/z	pos	Glucoheptonic acid	3365
1287	4.19_409.1891m/z	pos	WIN 55212-2	43434
1288	4.63_138.0656m/z	pos	2-Pyridylamide oxime	45425
1289	4.72_263.0335m/z	neg	Ammonium sulfate	92202
1290	5.19_307.1153m/z	pos	3-carboxy-4-methyl-5-(4-hydroxypentyl)-2-furanpropanoic acid	LMFA01150068

1291	5.34_255.0842m/z	pos	Glycerol 1-propanoate diacetate	HMDB0031640
1292	5.55_317.1125m/z	neg	Fluvoxamine acid	2951
1293	5.64_177.1194m/z	pos	1-(4-Fluorobenzyl) piperazine	96603
1294	5.96_460.1726m/z	pos	Pralatrexate	85196
1295	6.01_560.3106m/z	pos	LysoPC(18:1(11Z))	HMDB0010385
1296	6.02_219.0581m/z	pos	3-(Isothiocyanatomethyl)-1-methoxy-1H-indole	HMDB0038463
1297	6.03_213.0175m/z	neg	Deoxyribose 5-phosphate	HMDB0001031
1298	6.05_269.1292m/z	neg	N-Methylformamide	58023
1299	6.06_331.1287m/z	neg	Pilosine	68426
1300	6.32_187.0657m/z	pos	Eupolauridine	HMDB0030182
1301	7.14_267.1719m/z	pos	Tributyl phosphate	70062
1302	7.27_647.2696m/z	pos	CAY10626	64842
1303	7.34_368.1686n	pos	(2S)-2-Butanol O-[b-D-Apiofuranosyl-(1->6)-b-D-glucopyranoside]	HMDB0041255
1304	7.41_601.2720m/z	pos	PKODiA-PA	LMGP20070008
1305	7.53_183.1021m/z	neg	9,10-dihydroxy-2-decenoic acid	LMFA01050428
1306	7.59_299.1381m/z	pos	Balsoxine	68395
1307	7.91_313.1185m/z	neg	N-Butyl-beta-carboline-3-carboxylate	69712
1308	7.97_336.0556m/z	pos	Phosphoribosylformylglycineamidine	HMDB0000999
1309	8.05_292.9833m/z	neg	2-Deoxy-D-ribose 1,5-bisphosphate	66127
1310	8.32_251.1285m/z	neg	Methyl sorbate	86362

1311	8.32_313.1185m/z	neg	N,N'-Diacetylbenzidine	72940
1312	8.38_399.2196m/z	neg	Naphthyl dipeptide	69283
1313	8.65_511.3322n	pos	Scyphostatin A	LMSP01080060
1314	8.73_431.3045n	pos	Malyngamide H	65446
1315	9.12_608.3436m/z	neg	b-D-Glucopyranosiduronic acid, (3a,5b)-24-[(carboxymethyl)amino]-24-oxocholan-3-yl	6694
1316	9.15_253.1438m/z	neg	Eremopetasinorol	HMDB0029668
1317	9.24_326.2165m/z	pos	(R)-1-O-b-D-glucopyranosyl-1,3-octanediol	86223
1318	9.58_392.2917n	neg	3a,7a-Dihydroxycholanoic acid	HMDB0000384
1319	9.66_335.2107n	pos	Ankorine	64423
1320	9.79_237.1491m/z	neg	2,4,5,7alpha-Tetrahydro-1,4,4,7a-tetramethyl-1H-inden-2-ol	HMDB0036684
1321	0.63_174.9559m/z	neg	Calcium formate	72387
1322	0.67_309.2058m/z	pos	Diplodiatoxin	87705
1323	0.68_255.0645m/z	pos	5-L-Glutamyl-taurine	HMDB0004195
1324	0.80_207.0675m/z	neg	2-Cyanopyridine	65730
1325	0.83_331.0566m/z	pos	6-[2-(2H-1,3-benzodioxol-5-yl)-1-hydroxyethyl]-4-methoxy-5,6-dihydro-2H-pyran-2-one	HMDB0131687

1326	0.83_642.1063n	pos	(5-{4-[2-(3,4-dihydroxyphenyl)-3,5,7-trihydroxy-3,4-dihydro-2H-1-benzopyran-8-yl]-3,7-dihydroxy-3,4-dihydro-2H-1-benzopyran-2-yl}-2-hydroxyphenyl)oxidanesulfonic acid	HMDB0135073
1327	0.92_287.1983m/z	pos	Rizatriptan	HMDB0015088
1328	0.92_307.1163m/z	pos	Histidinyl-Isoleucine	HMDB0028888
1329	1.21_187.0420m/z	neg	F-Honaucin A	65489
1330	1.22_166.0861m/z	pos	3,4-Dihydro-2H-1-benzopyran-2-one	HMDB0036626
1331	1.24_383.0064m/z	neg	dTDP	HMDB0001274
1332	1.27_162.1117m/z	pos	Malonyl-Carnitin	HMDB0062496
1333	1.28_318.9975m/z	neg	(2-Furanylmethyl) methyl disulfide	HMDB0036807
1334	1.48_260.9933m/z	neg	(2E)-4-hydroxy-3-methylbut-2-en-1-yl trihydrogen diphosphate	53272
1335	10.17_278.2111m/z	pos	2-Polyprenyl-6-methoxyphenol	HMDB0060355
1336	10.20_399.1273m/z	pos	(1x,2x)-Guaiacylglycerol 3-glucoside	HMDB0040600
1337	10.22_281.1409m/z	pos	Lycodine	67981
1338	10.23_413.1995m/z	pos	Ketanserin	889
1339	10.47_454.3314m/z	pos	Cervonyl carnitine	HMDB0006510
1340	10.76_242.1536n	pos	AMPROLIUM	43586
1341	10.95_569.3514n	pos	Janthitrem C	HMDB0040684
1342	11.02_327.0804m/z	pos	N-Acetylaspartylglutamic acid	HMDB0001067

1343	11.18_453.3210m/z	neg	27-Norcholestanehexol	HMDB0002157
1344	11.21_237.1847m/z	pos	Acorusnol	HMDB0030920
1345	11.21_889.5346m/z	pos	PG(20:2(11Z,14Z)/22:4(7Z,10Z,13Z,16Z))	LMGP04010587
1346	11.26_666.2715m/z	neg	Neoacrimarine B	HMDB0040385
1347	11.56_597.3044m/z	neg	AGC	43439
1348	11.81_635.3803m/z	neg	Madlongiside C	HMDB0037904
1349	11.89_816.6096m/z	pos	PS(15:0/24:0)	HMDB0112340
1350	11.93_640.2928m/z	neg	S-(9-deoxy-delta9,12-PGD2)-glutathione	HMDB0013058
1351	12.27_402.2284n	pos	Cinitapride	85593
1352	13.20_337.1697m/z	pos	H-1152	45068
1353	14.02_492.4070m/z	pos	20,24-Epoxy-25,26-dihydroxydammaran-3-one	HMDB0039692
1354	15.47_497.2781m/z	pos	N-desmethylimatinib	HMDB0013862
1355	2.14_383.1069m/z	neg	Alanyl-Cysteine	HMDB0028684
1356	3.01_311.0181m/z	neg	Blighinone	HMDB0030643
1357	4.35_142.0868m/z	pos	5-Acetamidopentanoate	63464
1358	5.43_335.0091m/z	neg	3-[3-methoxy-4-(sulfooxy)phenyl]-2-oxopropanoic acid	HMDB0126595
1359	5.80_171.1009m/z	pos	cis- and trans-Ethyl 2,4-dimethyl-1,3-dioxolane-2-acetate	88254
1360	5.83_387.1832m/z	neg	Argentine	68550
1361	6.05_347.0058m/z	neg	Ellagic acid	HMDB0002899

1362	6.09_214.1450m/z	neg	N-Nonanoylglycine	HMDB0013279
1363	6.19_510.2556m/z	pos	N-Ac-Tyr-Val-Ala-Asp-CHO	45363
1364	6.25_241.0958m/z	pos	(E)-Monocrotophos	HMDB0031805
1365	6.43_464.2866m/z	pos	N-arachidonoyl histidine	LMFA08020131
1366	6.44_315.2377m/z	pos	Sannamycin B	71873
1367	6.80_362.2109n	pos	Isohumulone A	HMDB0030026
1368	7.34_464.3003m/z	pos	17-phenoxy trinor Prostaglandin F2α isopropyl ester	45293
1369	7.44_505.0376m/z	neg	5-(3'-Carboxy-3'-oxopropenyl)-4,6-dihydroxypicolinate	63550
1370	7.63_111.0442m/z	pos	Hydroquinone	505
1371	7.83_295.2272m/z	pos	Dihydroxychaulmoogric acid	LMFA01140029
1372	7.86_613.3585m/z	neg	Cuscutic resinoside A	LMSL05000008
1373	7.88_499.1291m/z	neg	6-[(6-{[3,4-dihydroxy-2,5-bis(hydroxymethyl)oxolan-2-yl]oxy}-3,4,5-trihydroxyoxan-2-yl)methoxy]-3,4,5-trihydroxyoxane-2-carboxylic acid	HMDB0125617
1374	7.91_480.2190m/z	pos	Leukotriene E3	LMFA03020074
1375	7.96_297.0467m/z	neg	6-(carboxymethoxy)-3,4,5-trihydroxyoxane-2-carboxylic acid	HMDB0124938
1376	8.02_497.2736m/z	neg	DIHYDROFLAVOPEREIRINE	84953
1377	8.04_287.1423m/z	pos	Fenothiocarb sulfoxide	68827
1378	8.30_151.1121m/z	pos	Perillyl aldehyde	LMPR0102090010
1379	8.44_221.0811m/z	neg	2,2-dimethyl-2H-chromen-5-ol	HMDB0126379

1380	8.48_167.1074m/z	neg	Methyl propenyl ketone	58028
1381	8.71_424.1535m/z	pos	O-Methylandrocymbine	64398
1382	9.53_431.1699m/z	neg	(2S,4S,6S)-2-[2-(4-Hydroxy-3-meyhoxyphenyl)ethyl]tetrahydro-6-(4,5-dihydroxy-3-methoxyphenyl)-2H-pyran-4-yl 4-acetate	HMDB0030503
1383	9.66_390.1429m/z	pos	Tryptophyl-Tyrosine	HMDB0029095
1384	9.69_352.0991m/z	neg	Aspergillomarasmine A	HMDB0029450
1385	9.90_252.9989m/z	pos	3-hydroxy-2-(hydroxymethyl)-2-[(sulfooxy)methyl]propanoic acid	HMDB0127656
1386	0.76_132.1018m/z	pos	L-Isoleucine	HMDB0000172
1387	0.81_441.0959m/z	neg	4-Hydroxylevamisole Glucuronide	728
1388	0.83_979.1616m/z	neg	Calcium Gluceptate	85364
1389	0.87_132.0299m/z	neg	L-Aspartic acid	HMDB0000191
1390	0.96_197.0067m/z	neg	Sodium glucuronate	69532
1391	0.96_353.0317m/z	pos	[2-hydroxy-1-(7-hydroxy-1-oxo-1H-isochromen-3-yl)butoxy]sulfonic acid	HMDB0130071
1392	1.21_503.0756m/z	neg	Hypericin	HMDB0034271
1393	1.24_165.0045m/z	neg	Hydroxymalonate	65746
1394	1.28_427.0088m/z	neg	Olsalazine-O-sulfate	HMDB0060600
1395	1.61_129.0548m/z	pos	Adipic acid	HMDB0000448
1396	10.09_227.1647m/z	neg	2-Dodecenal	HMDB0031020

1397	10.13_428.1104n	pos	7,14-dihydroxy-6-methoxy-4,12-dimethyl-5-(3-methylbut-2-enoyl)-10-oxo-2,9-dioxatricyclo[9.4.0.0³,⁸]pentadeca-1(11),3(8),4,6,12,14-hexaene-15-carboxylic acid	HMDB0139005
1398	10.34_480.2210m/z	pos	Retrocalamin	HMDB0038159
1399	10.35_310.0906m/z	pos	Tetraphyllin B	HMDB0029914
1400	10.81_454.9964m/z	neg	3,5-Dinitrosalicylic acid	68986
1401	10.84_405.1489m/z	pos	HDMBOA-Glc	HMDB0037265
1402	10.87_179.1436m/z	pos	Dihydro-5-(2-octenyl)-2(3H)-furanone	87239
1403	10.98_129.1021m/z	pos	N-Acetyl-2,3-dihydro-1H-pyrrole	87456
1404	11.61_459.3119m/z	pos	Pubesenolide	89500
1405	12.62_245.2262m/z	pos	6,10,14-Trimethyl-5,9,13-pentadecatrien-2-one	89972
1406	12.62_337.2737m/z	pos	PGF2alpha methyl ether	LMFA03010073
1407	12.72_313.2376m/z	neg	1-methylpropyl-tridecanolide	LMFA07040053
1408	13.30_672.5305m/z	pos	PC(O-14:0/O-14:0)	40197
1409	13.79_352.2029m/z	pos	4-Chloromethandienone	HMDB0004634
1410	15.89_568.4324m/z	pos	1-(2-methoxy-tricosanyl)-sn-glycero-3-phosphoethanolamine	LMGP02060027
1411	2.17_268.1061m/z	pos	OR-1896	HMDB0060861
1412	3.01_207.0469n	neg	Benzthiazuron	72806

1413	4.15_304.15 18m/z	pos	N5-Acetyl-N2-gamma-L-glutamyl-L-ornithine	HMDB0039423
1414	4.19_275.06 24m/z	pos	Hydroxyflutamide	674
1415	4.25_103.05 42m/z	pos	Phenylacetaldehyde	HMDB0006236
1416	4.49_142.06 57m/z	neg	(R)-Boschniakine	HMDB0030267
1417	5.03_188.12 91m/z	pos	Phenformin	HMDB0015050
1418	5.06_135.04 33m/z	pos	3-Hydroxyphenylacetic acid	HMDB0000440
1419	5.31_329.18 83m/z	pos	16alpha-Fluoro-17alpha-hydroxyandrost-4-en-3-one	70567
1420	5.43_139.02 48m/z	pos	5-Diazouracil	70092
1421	5.48_351.16 15m/z	neg	Cinnamyl acetate	HMDB0029699
1422	5.50_426.15 07n	neg	Chitobiose	HMDB0003556
1423	6.29_433.20 61m/z	neg	Alanyl-Gamma-glutamate	HMDB0028701
1424	6.56_274.08 99m/z	pos	N-(1-Deoxy-1-fructosyl)alanine	HMDB0038662
1425	6.81_195.13 58m/z	pos	Capric acid	HMDB0000511
1426	6.81_335.18 78m/z	pos	Clidinium	HMDB0014909
1427	7.78_237.07 74m/z	pos	Oxaburimamide	69231
1428	7.94_586.36 14m/z	pos	Fumonisin AK1	HMDB0033397
1429	7.96_151.11 23m/z	neg	8-Methylnonenoate	HMDB0012183
1430	7.97_513.26 83m/z	neg	3,17-Androstanediol glucuronide	LMST05010037
1431	8.43_165.02 00m/z	neg	Demethylphosphinothricin	63600
1432	8.48_144.99 12m/z	neg	Flupropanate	72616
1433	9.03_424.15 22m/z	pos	Kreysigine	64402

1434	9.24_479.2850m/z	pos	STROPHANTHIDIN SEMICARBAZIDE	44254
1435	9.34_197.1541m/z	pos	3-oxo-dodecanoic acid	LMFA01060091
1436	9.38_265.1069m/z	pos	3-carboxy-4-methyl-5-(4-oxopentyl)-2-furanpropanoic acid	LMFA01150067
1437	9.44_306.2649m/z	pos	(S)-10,16-Dihydroxyhexadecanoic acid	HMDB0037798
1438	9.55_455.2460m/z	neg	Metyrapol	1258
1439	9.65_238.9963m/z	neg	6-Phosphonoglucono-D-lactone	HMDB0001127
1440	9.85_195.1387m/z	neg	Cyclohexanone	58147
1441	9.91_467.1175m/z	pos	b-D-Xylopyranosyl-(1->4)-a-L-rhamnopyranosyl-(1->2)-L-arabinose	HMDB0041221
1442	0.62_265.0199m/z	pos	3-(Phosphoacetylamido)-L-alanine	66116
1443	0.68_239.1630m/z	pos	5-Nonyltetrahydro-2-oxo-3-furancarboxylic acid	HMDB0030993
1444	0.75_203.0199m/z	neg	2-Maleylacetate	HMDB0060348
1445	0.79_243.1158m/z	pos	Ethiofencarb	HMDB0031782
1446	0.80_102.0916m/z	pos	Ammonium isovalerate	92812
1447	0.87_159.1134m/z	neg	N(6)-Methyllysine	HMDB0002038
1448	0.97_255.0587m/z	neg	Ammelide	73558
1449	1.18_463.0908m/z	pos	Adifoline	67357
1450	10.25_414.0104n	neg	Alginic acid	HMDB0029940
1451	10.36_251.1309m/z	pos	DMABA-d6 NHS ester	96492

1452	10.66_703.1688m/z	pos	6-{4-[(1E)-3-[(6-{[3,4-dihydroxy-2,5-bis(hydroxymethyl)oxolan-2-yl]oxy}-3,4,5-trihydroxyoxan-2-yl)methoxy]-3-oxoprop-1-en-1-yl]-2-hydroxyphenoxy}-3,4,5-trihydroxyoxane-2-carboxylic acid	HMDB0125601
1453	10.86_263.2353m/z	pos	Palmitaldehyde	LMFA06000088
1454	11.22_233.1539m/z	neg	Acorusdiol	HMDB0030919
1455	11.23_330.3378m/z	pos	Arachidic acid	HMDB0002212
1456	11.48_532.3440m/z	pos	Flavidulol C	HMDB0039150
1457	11.90_373.2851m/z	pos	DL-PDMP (hydrochloride)	43407
1458	11.94_653.3389m/z	neg	azaperone	3933
1459	12.03_254.2488m/z	pos	13Z-Hexadecen-11-yn-1-ol	LMFA05000202
1460	12.20_592.4784m/z	pos	Glucosyl 25-hydroxyhexacosanoate	LMFA13010053
1461	12.66_184.0736m/z	pos	4-Trimethylammoniobutanoic acid	966
1462	12.81_327.2073m/z	pos	Atherosperminine	HMDB0030304
1463	13.12_531.2784m/z	pos	Cinncassiol D2 glucoside	HMDB0034679
1464	13.20_339.1656m/z	pos	1H-1,2,4-Triazole-1-propanoic acid, 4,5-dihydro-3-(1-hydroxyethyl)-5-oxo-4-(2-phenoxyethyl)-	1477
1465	14.11_563.4346m/z	pos	Nb-Pentacosanoyltryptamine	HMDB0040820
1466	2.13_305.1654m/z	pos	Akuammicine	67361

1467	2.13_317.1178m/z	pos	Karanjachromene	LMPK12111544
1468	2.13_410.2566m/z	pos	Trospium	HMDB0014354
1469	2.48_290.1369m/z	pos	Codonopsine	68144
1470	3.15_169.0989m/z	pos	2-Octanol	LMFA05000620
1471	4.18_665.1818m/z	neg	Ethalfluralin	72592
1472	4.19_421.1992m/z	pos	Sarcostin	71890
1473	4.19_650.2201n	pos	5-Hydroxy-7,3',4'-trimethoxy-8-methylisoflavone 5-O-neohesperidoside	LMPK12050358
1474	4.25_400.0797n	pos	Theaflagallin	HMDB0033287
1475	4.44_511.0562m/z	pos	{4-[3-(4-hydroxy-3,6-dioxo-2-{[3,4,5-trihydroxy-6-(hydroxymethyl)oxan-2-yl]oxy}cyclohexa-1,4-dien-1-yl)-3-oxoprop-1-en-1-yl]phenyl}oxidanesulfonic acid	HMDB0126625
1476	4.91_474.2947m/z	pos	PC(O-14:1(1E)/0:0)	40397
1477	5.11_295.0155m/z	pos	N1,N4-Diacetylsulfanilamide	2528
1478	5.33_253.0707m/z	neg	L-Phosphoarginine	HMDB0029438
1479	5.55_485.1659m/z	neg	Cytarabine	HMDB0015122
1480	5.83_146.0614m/z	pos	3-Fluorocatechol	71213
1481	6.18_365.2341m/z	pos	Indoramin	830
1482	6.25_464.1932m/z	pos	Dihydroisomorphine-6-glucuronide	HMDB0061137
1483	6.37_496.2113m/z	pos	10,11-Dihydro-12R-hydroxy-leukotriene E4	HMDB0012501
1484	6.40_375.1182m/z	neg	Miraxanthin-III	67062

1485	6.45_181.1589m/z	pos	Allyl nonanoate	LMFA07010778
1486	6.45_589.3029m/z	pos	D-Urobilin	HMDB0004161
1487	6.89_200.1289m/z	neg	Methylisopelletierine	HMDB0030326
1488	7.56_507.1662m/z	pos	8-(1-hydroxy-2-{[(2E)-3-(4-hydroxyphenyl)prop-2-enoyl]oxy}propan-2-yl)-2-oxo-2H,8H,9H-furo[2,3-h]chromen-9-yl 3-methylbut-2-enoate	HMDB0128939
1489	7.59_180.1744m/z	pos	Hexylbenzene	HMDB0061815
1490	7.61_512.2729m/z	pos	Ustiloxin D	HMDB0041054
1491	7.91_385.0939m/z	pos	Dihydrorhodamine 123	64881
1492	7.94_590.3458m/z	pos	PHOOA-PE	LMGP20020023
1493	7.94_624.3386m/z	neg	dihydro-α-ergocryptine	4042
1494	8.01_301.1198m/z	neg	Dopamine quinone	64470
1495	8.29_357.2817m/z	pos	2-thio-Acetyl MAGE	45189
1496	8.38_247.0795m/z	pos	Temurin	HMDB0004328
1497	9.11_165.0915m/z	neg	2Z,4E,6Z-Decatrienoic acid	LMFA01031053
1498	9.15_385.2373m/z	neg	7alpha-Hydroxy-3,12-dioxo-5beta-cholan-24-oic Acid	LMST04010194
1499	9.21_236.2378m/z	pos	(3E,7E)-4,8,12-Trimethyl-1,3,7,11-tridecatetraene	HMDB0034498
1500	9.51_511.3016m/z	neg	1-Stearoylglycerophosphoglycerol	HMDB0061697
1501	9.62_177.0558m/z	pos	4-Methylumbelliferone	HMDB0059622
1502	9.76_300.9919m/z	neg	Erythrityl Tetranitrate	85532

1503	9.83_181.1221m/z	pos	2,2,7,7-Tetramethyl-1,6-dioxaspiro[4.4]nona-3,8-diene	HMDB0030007
1504	0.64_301.0013m/z	pos	Bentazone-sodium	72395
1505	0.73_212.0202m/z	neg	o-Nitrobenzoate	71157
1506	0.77_307.0789m/z	neg	4-Fluorophenylacetic acid	1288
1507	0.79_113.0713m/z	pos	L-cis-3-Amino-2-pyrrolidinecarboxylic acid	86241
1508	0.80_398.0839n	pos	3,4,5-trihydroxy-6-{[4-methoxy-6-(3-oxoprop-1-en-1-yl)-2H-1,3-benzodioxol-5-yl]oxy}oxane-2-carboxylic acid	HMDB0128691
1509	0.84_627.1364m/z	neg	Valdecoxib	HMDB0005033
1510	0.97_203.0676m/z	neg	1-(Hydroxymethyl)-5,5-dimethyl-2,4-imidazolidinedione	HMDB0031670
1511	1.11_182.9544m/z	neg	Cyanosulfurous acid anion	HMDB0012916
1512	1.20_145.0134m/z	neg	Oxoglutaric acid	HMDB0000208
1513	1.21_232.0200n	neg	Chrycolide	HMDB0035145
1514	1.23_323.0123m/z	neg	N-Nitrosothiazolidine-4-carboxylic acid	HMDB0040192
1515	10.01_385.0935m/z	pos	1-hydroxy-2-(beta-D-glucosyloxy)-9,10-anthraquinone	LMFA13010061
1516	10.04_115.9623m/z	pos	Rhodanine	66618
1517	10.16_484.3253m/z	pos	Galactosylsphingosine	HMDB0000648
1518	10.22_441.2127m/z	pos	Merodesmosine	HMDB0030407
1519	10.45_572.4022m/z	pos	Epoxomicin	45091

1520	10.53_253.1787m/z	pos	3(4->5)-Abeo-4,11:4,12-diepoxy-3-eudesmanol	HMDB0032122
1521	10.53_447.2187m/z	pos	Dulciol A	HMDB0029990
1522	10.63_279.0946m/z	pos	Triphenylphosphine oxide	64889
1523	10.63_425.2791m/z	pos	alpha-N-(3-hydroxyhexadecanoyl) L-ornithine	LMFA08020242
1524	10.77_489.2770m/z	neg	Tropacocaine	68627
1525	10.97_237.1851m/z	pos	Geranyl tiglate	HMDB0032292
1526	10.97_431.3157m/z	neg	4-cholesten-7α,12α,24-triol-3-one	HMDB0062399
1527	11.18_293.2483m/z	pos	3b,17b-Dihydroxyetiocholane	HMDB0000369
1528	11.19_179.1421m/z	pos	Nicotine imine	HMDB0001010
1529	11.52_492.3443m/z	neg	PC(P-17:0/0:0)	LMGP01070007
1530	11.76_243.2120m/z	pos	6-[5]-ladderane-1-hexanol	LMFA05000063
1531	11.93_311.1656m/z	pos	5-Hydroxy-7-(4-hydroxy-3-methoxyphenyl)-1-phenyl-3-heptanone	86319
1532	12.00_429.3711m/z	pos	Nervonoylacetone	91136
1533	12.07_529.3539m/z	pos	16-Glutaryloxy-1alpha,25-dihydroxyvitamin D3	LMST03020634
1534	12.08_660.4237m/z	pos	PS(14:0/14:1(9Z))	LMGP03010094
1535	12.24_507.3702n	pos	PC(O-18:1(9Z)/0:0)	LMGP01060039
1536	12.35_302.2468m/z	pos	Anandamide (18:4, n-3)	LMFA08040006
1537	13.20_221.2259m/z	pos	Hexadecenal	HMDB0060482
1538	13.41_283.1756m/z	pos	Hexaethylene glycol	HMDB0061822
1539	14.40_519.4070m/z	pos	Ganoderiol C	HMDB0037781

1540	15.99_159.1178m/z	pos	N-butyryl-L-Homoserine lactone-d5	96403
1541	2.00_164.0483n	pos	m-Coumaric acid	HMDB0001713
1542	2.00_307.0881m/z	pos	3-METHYLCHOLANTHRENE	44182
1543	2.06_191.0143m/z	neg	1-Propene, 1,3,3,3-tetrafluoro-2-(fluoromethoxy)-1-methoxy-, (E)-	2427
1544	2.67_340.1970m/z	pos	Butyl (S)-3-hydroxybutyrate glucoside	HMDB0031694
1545	2.83_235.0817m/z	neg	6-(2-Hydroxyethoxy)-6-oxohexanoic acid	HMDB0061681
1546	2.99_118.0652m/z	pos	Benzeneacetonitrile	HMDB0034171
1547	3.54_316.0982m/z	pos	Dihydrosanguinarine	64356
1548	4.18_508.1116m/z	neg	Carboxyltolmetin Glucuronide	2875
1549	4.49_263.0351m/z	neg	2,4,6-Octatriynoic acid	LMFA01030961
1550	5.06_375.0194m/z	neg	Kaempferol 7,4'-dimethyl ether 3-O-sulfate	LMPK12112596
1551	5.14_280.9951m/z	neg	METHAMIDOPHOS	44566
1552	5.51_321.0976m/z	pos	3,4,5-trihydroxy-6-{[(2E)-2-methyl-3-phenylprop-2-enoyl]oxy}oxane-2-carboxylic acid	HMDB0133621
1553	5.64_199.0976m/z	pos	Propionylcholine	HMDB0013305
1554	5.90_181.1592m/z	pos	cis-4-Decenyl acetate	HMDB0032214
1555	5.99_221.0833m/z	pos	SK&F 91581	69228
1556	6.25_323.1001m/z	pos	5-NITRO-2-PHENYLPROPYLAMINOBENZOIC ACID [NPPB]	44306
1557	6.33_100.0759m/z	pos	L-Valine	HMDB0000883

1558	6.35_287.0426m/z	neg	Furamizole	69954
1559	6.74_285.0347m/z	pos	Sorbitol-6-phosphate	58327
1560	6.95_151.1123m/z	pos	trans-4,5-epoxy-2(E)-decenal	HMDB0013105
1561	7.04_299.1388m/z	neg	Bifenazate	72390
1562	7.13_275.0500m/z	pos	Methionyl-Cysteine	HMDB0028970
1563	7.28_442.1628m/z	pos	Hydroxydomperidone(M2b)	2377
1564	7.66_519.2205m/z	pos	Glaucarubin	HMDB0035626
1565	7.78_315.1381m/z	pos	1-Dehydro-9-fluoro-11-oxotestololactone	70857
1566	7.83_168.9898m/z	neg	Thiazole	HMDB0029713
1567	8.01_274.1865m/z	pos	Arginyl-Valine	HMDB0028722
1568	8.03_463.2700m/z	pos	Retinyl beta-glucuronide	HMDB0010340
1569	8.87_151.1232m/z	pos	1,2,3,4-Tetrahydroisoquinoline	HMDB0012489
1570	8.93_334.2375m/z	pos	CARBETAPENTANE	43477
1571	9.16_225.1967m/z	pos	N,N'-Dicyclohexylurea	34503
1572	9.16_611.3785m/z	neg	W123	64710
1573	9.39_639.3855m/z	pos	PI(22:0/0:0)	LMGP06050024
1574	9.66_356.1702m/z	neg	Histidinyl-Arginine	HMDB0028879
1575	9.90_176.9900m/z	pos	3-dimethylarsinoyl-propionic acid	LMFA00000038
1576	0.62_262.0945n	pos	L-cis-Cyclo(aspartylphenylalanyl)	HMDB0031360
1577	0.73_154.0615m/z	neg	3-(Pyrazol-1-yl)-L-alanine	65565
1578	0.84_315.0410m/z	neg	Calcium levulinate anhydrous	72192

1579	0.89_389.0502m/z	neg	3,4,5-trihydroxy-6-({8-methoxy-2-oxo-2H-furo[2,3-h]chromen-4-yl}oxy)oxane-2-carboxylic acid	HMDB0135849
1580	0.89_797.2682m/z	neg	Oxmetidine	HMDB0041970
1581	0.97_239.1626m/z	pos	3-Hydroxydodecanoic acid	5376
1582	1.23_221.0118m/z	neg	Thiazafluron	72835
1583	1.42_144.0241m/z	pos	3-Methylthiopropanamine	65962
1584	1.71_243.2322m/z	pos	Methyl tetradecanoate	86999
1585	10.08_397.2224m/z	neg	PGI2	LMFA03010087
1586	10.70_275.1650m/z	neg	Phytuberin	67898
1587	10.70_479.3357m/z	neg	7-oxo-11E-Tetradecenoic acid	LMFA01060186
1588	10.74_561.2553m/z	neg	Eriojaposide B	HMDB0038029
1589	10.77_995.6119m/z	neg	PIM1(17:0/18:0)	LMGP15010012
1590	10.84_460.2831m/z	pos	AGELASINE	43731
1591	10.90_512.3571m/z	pos	Makisterone A	71399
1592	11.03_580.2947m/z	pos	19-Hydroxycinnzeylanol 19-glucoside	HMDB0036856
1593	11.11_425.0529m/z	pos	Quercetin 7,3',4'-trimethyl ether 3-sulfate	LMPK12112657
1594	11.26_583.3371m/z	neg	Thioperamide	45306
1595	11.37_293.2116m/z	pos	12(13)Ep-9-KODE	HMDB0013623
1596	11.40_580.3625m/z	pos	POB-PC	LMGP20010021
1597	11.83_636.3900m/z	pos	desferrioxamine G	LMFA08020171
1598	11.89_341.3062m/z	pos	Glycidyl stearate	73128

1599	11.91_191.1793m/z	pos	(E)-6,10-Dimethyl-9-methylene-5-undecen-2-one	HMDB0035862
1600	11.91_682.4434m/z	pos	7-Epizucchini factor A	HMDB0036405
1601	12.54_243.2133m/z	pos	2-N-Undecyltetrahydrothiophene	68825
1602	13.20_181.1594m/z	pos	2,6-Dimethyl-5-heptenal propyleneglycol acetal	HMDB0032235
1603	2.12_348.2037m/z	pos	Palustrine	68425
1604	2.38_160.0607m/z	neg	Glucosamine	HMDB0001514
1605	4.31_132.1016m/z	pos	(±)-erythro-Isoleucine	HMDB0033923
1606	6.56_111.0437m/z	pos	2-Hydroxy-cis-hex-2,4-dienoate	69013
1607	7.39_411.1820m/z	neg	Ethyl trans-p-methoxycinnamate	HMDB0030762
1608	8.39_399.3476m/z	pos	Axillarenic acid	LMFA01050418
1609	8.44_523.2907m/z	pos	14-O-(beta-D-glucopyranosyl)-7S,14R-dihydroxy-7,9,13,17-tetramethyl-2E,4E,8E,10E,12E,16E-octadecahexaenoic acid	LMFA13010034
1610	8.56_305.1771m/z	pos	PARAROSANILINE	43540
1611	8.83_323.1616m/z	neg	Leucyl-phenylalanine	HMDB0013243
1612	8.94_213.1169m/z	pos	Burimamide	66645
1613	9.12_277.0969m/z	pos	AG-490	45246
1614	9.22_350.2090m/z	neg	Sphingosine 1-phosphate (d16:1-P)	HMDB0060061
1615	9.80_409.2749m/z	pos	Apo-10'-violaxanthal	93624
1616	9.91_370.0081n	neg	Sedoheptulose 1,7-bisphosphate	156

1617	0.22_396.0880m/z	pos	Niazimicin A	HMDB0031944
1618	0.62_410.3628m/z	pos	Stearoylcarnitine	HMDB0000848
1619	0.68_313.0373m/z	pos	{[(3E)-4-(5-hydroxy-1-oxo-1H-isochromen-3-yl)but-3-en-2-yl]oxy}sulfonic acid	HMDB0130051
1620	0.68_317.1174m/z	pos	Calopogoniumisoflavone A	47558
1621	0.76_431.0816m/z	pos	Desacetylcefotaxime	HMDB0041868
1622	0.81_357.0284m/z	neg	{[(3E)-4-(5-hydroxy-1-oxo-1H-isochromen-3-yl)but-3-en-1-yl]oxy}sulfonic acid	HMDB0130058
1623	0.97_434.9881m/z	neg	FOSFOSAL	44294
1624	0.97_542.0528m/z	neg	6,11-dihydroxy-7-(4-hydroxy-3,5-dimethoxyphenyl)-3-[4-hydroxy-3-(sulfooxy)phenyl]-2λ^4,8-dioxatricyclo[7.3.1.$0^{5,13}$]trideca-1(13),2,4,6,9,11-hexaen-2-ylium	HMDB0134859
1625	1.21_400.0996m/z	pos	De-O-methylsimmondsin	95628
1626	1.25_201.0378m/z	neg	Chrycorin	HMDB0035149
1627	10.08_617.3100m/z	pos	8'-Hydroxydihydroergotamine	2261
1628	10.23_503.2964m/z	pos	CRUSTECDYSONE	43863
1629	10.28_367.2492m/z	pos	15S-HETE-d8	36332
1630	10.60_536.2884m/z	pos	Geranylfarnesyl diphosphate	62877
1631	10.74_526.2980m/z	pos	Pederin	71091
1632	10.83_437.1974m/z	pos	Obacunone	HMDB0035858

1633	10.93_351.2186m/z	pos	Propantheline	HMDB0014920
1634	11.01_447.3466m/z	pos	13'-Carboxy-gamma-tocopherol	HMDB0012557
1635	11.05_540.3346m/z	neg	(25R)-3alpha,7alpha-dihydroxy-5beta-cholestan-27-oyl taurine	LMST05040008
1636	11.37_253.2172m/z	pos	3-keto palmitic acid	35745
1637	11.60_494.3625m/z	pos	PE(P-20:0/0:0)	LMGP02070004
1638	11.84_418.3096n	pos	11'-Carboxy-alpha-chromanol	HMDB0012515
1639	12.04_780.1506m/z	pos	Malvidin 3-(6-malonylglucoside) 5-glucoside	93459
1640	12.10_492.3828n	pos	Xeniasterol-a	LMST01031064
1641	12.97_899.4992m/z	pos	Hoduloside VIII	95138
1642	14.24_293.2836m/z	pos	Paullinic acid	HMDB0035159
1643	15.47_549.4104m/z	pos	Monoanhydroescholtzxanthin	LMPR01070026
1644	15.67_138.0656m/z	pos	6-Aminonicotinamide	44444
1645	2.05_365.1217m/z	pos	3,4,5-trihydroxy-6-{5-methoxy-2-[(1E)-3-oxopent-1-en-1-yl]phenoxy}oxane-2-carboxylic acid	HMDB0132990
1646	2.14_263.0463m/z	neg	Necatorine	HMDB0039035
1647	3.00_244.0396n	pos	{3-[(1E)-3-hydroxy-2-methylprop-1-en-1-yl]phenyl}oxidanesulfonic acid	HMDB0133648
1648	4.19_433.1475m/z	pos	cis-3-Hexenyl b-primeveroside	HMDB0031690
1649	4.19_531.1050m/z	pos	Americanin B	92272
1650	5.04_722.2140m/z	pos	Pteroyltriglutamic acid	6381

1651	5.22_313.1573m/z	pos	Phenylalanylphenylalanine	HMDB0013302
1652	5.24_268.0395m/z	pos	N-(3-oxo-hexanoyl)-homoserine thiolactone	LMFA08020240
1653	5.97_299.1395m/z	pos	Oxoassoanine	69383
1654	6.00_213.0551m/z	neg	3-[(1E)-but-1-en-1-yl]-5,6-dihydroxy-1H-isochromen-1-one	HMDB0130045
1655	6.01_132.0809m/z	pos	3-Methylindole	HMDB0000466
1656	6.18_558.2930m/z	pos	LysoPC(18:2(9Z,12Z))	HMDB0010386
1657	6.29_369.1726m/z	neg	Cyclomammein	HMDB0030711
1658	6.78_535.0404m/z	pos	PU-H71	96497
1659	7.90_399.2196m/z	neg	Nevskin	HMDB0030162
1660	7.96_324.0678m/z	neg	N-(7-Mercaptoheptanoyl)threonine 3-O-phosphate	63904
1661	8.08_427.1670m/z	pos	5-Methoxy-7,8-diprenylflavone	48532
1662	8.14_583.2663m/z	neg	TMC-1C	LMFA08020205
1663	8.65_187.1334m/z	neg	cis-p-Menthane-1,7,8-triol	HMDB0034783
1664	8.88_425.1984m/z	neg	Fludrocortisone	HMDB0014825
1665	9.01_223.1703m/z	pos	L-Menthyl acetoacetate	88396
1666	9.03_343.2941m/z	pos	N-pentadecanoyl-L-Homoserine lactone	64795
1667	9.38_425.1419m/z	pos	Benzyl beta-primeveroside	HMDB0041190
1668	9.40_446.3271m/z	pos	25-Hydroxyvitamin D3-26,23-lactone	HMDB0060126
1669	9.52_385.1670m/z	pos	16alpha-hydroxydehydroepiandrosterone 3-sulfate	HMDB0062611

1670	9.66_295.2277m/z	pos	13-L-Hydroperoxylinoleic acid	HMDB0003871
1671	9.66_609.3506m/z	neg	Halocins	HMDB0038173
1672	9.84_389.2710m/z	pos	Gentian Violet	4055
1673	0.61_279.0488m/z	pos	Caffeoylmalic acid	HMDB0029318
1674	0.66_207.9744m/z	neg	2-Thiothiazolidine-4-carboxylic acid	HMDB0041805
1675	0.77_302.0402n	pos	Quercetin	HMDB0005794
1676	0.80_148.0366n	neg	D-2-Hydroxyglutaric acid	HMDB0000606
1677	0.80_196.0721m/z	neg	N-Acetylhistidine	HMDB0032055
1678	0.80_245.0440m/z	neg	Phosphatidyl glycerol	186
1679	0.82_275.0985m/z	pos	Canavaninosuccinate	62849
1680	0.83_657.1958m/z	neg	Isradipine Metabolite (3,5-Pyridinedicarboxylic acid, 4-(2,1,3-benzoxadiazol-4-yl)-1,4-dihydro-2,6-d	873
1681	0.83_679.1772m/z	neg	Sulindac sulfide	2605
1682	0.99_258.0316m/z	pos	Sulfamethoxazole sodium	71876
1683	1.17_232.0403m/z	neg	7-Methylpyrido[3,4-c]psoralen	73152
1684	1.22_203.1408m/z	pos	3,6,8-dodecatrien-1-ol	LMFA05000058
1685	1.46_310.9942m/z	neg	Pyrroloquinoline quinone	HMDB0013636
1686	10.04_421.3208m/z	pos	Nb-Palmitoyltryptamine	HMDB0040815
1687	10.22_516.1553m/z	pos	Furcelleran	92382
1688	10.25_215.1648m/z	neg	Undecanal	HMDB0030941

1689	10.27_587.2261m/z	pos	2-{2,6-dihydroxy-4-[6-hydroxy-7-(3-methylbut-2-en-1-yl)-1-benzofuran-2-yl]-3-methoxyphenyl}-6-(2,4-dihydroxyphenyl)-4-methylcyclohex-3-ene-1-carboxylic acid	HMDB0126228
1690	10.41_526.2944m/z	neg	LysoPE(22:5(4Z,7Z,10Z,13Z,16Z)/0:0)	HMDB0011524
1691	10.65_632.3183m/z	neg	PHOOA-PS	LMGP20040005
1692	10.79_551.3184m/z	pos	24S-methylcholest-22E-en-3beta,4beta,5alpha,6alpha,8beta,14alpha,15alpha,25,28-nonol	84029
1693	10.87_277.1442m/z	neg	(2Z)-2-[(3-hydroxyphenyl)methylidene]octanal	HMDB0134125
1694	10.98_427.3195m/z	pos	1-O-(2R-hydroxy-eicosanyl)-sn-glycerol	LMGL01020066
1695	11.18_901.5008m/z	pos	PGP(18:1(11Z)/22:6(4Z,7Z,10Z,13Z,16Z,19Z))	HMDB0013531
1696	11.21_321.2443m/z	pos	14,15-Epoxy-5,8,11-eicosatrienoic acid	HMDB0002283
1697	11.70_480.3092m/z	neg	1-(2-methoxy-6Z-heptadecenyl)-sn-glycero-3-phosphoethanolamine	LMGP02060019
1698	11.87_841.5711n	pos	PI-Cer(t18:0/18:0(2OH))	103166
1699	11.90_319.2856m/z	pos	1-O-(2R-hydroxy-pentadecyl)-sn-glycerol	LMGL01020062

1700	12.11_377.2692m/z	neg	Adrenic Acid	LMFA01030178
1701	12.64_404.3162m/z	pos	Gamma-linolenyl carnitine	HMDB0006318
1702	15.90_297.0468m/z	neg	3,4,5-trihydroxy-6-[(2-hydroxyacetyl)oxy]oxane-2-carboxylic acid	HMDB0124939
1703	2.00_279.0382m/z	neg	5-Hydroxypyrazinoic acid	2198
1704	2.00_306.0570m/z	neg	Aminoparathion	HMDB0001504
1705	2.01_169.0615m/z	neg	Methylimidazole acetaldehyde	HMDB0004181
1706	4.19_220.0832n	pos	5-Hydroxy-L-tryptophan	HMDB0000472
1707	4.44_244.1560m/z	pos	Pinacidil-N-Oxide	1942
1708	4.82_385.3197m/z	pos	Macamide	LMFA08020159
1709	5.04_289.0085m/z	pos	Diflunisal	2029
1710	5.64_389.0900m/z	neg	4-Methoxybenzenepropanol 1-(2-sulfoglucoside)	HMDB0034757
1711	6.18_539.2490m/z	neg	7,11-Bisdeacetylvaltrate 7-(3-methylpentanoate) 11-(3-hydroxy-3-methylbutanoate)	HMDB0033655
1712	6.49_159.1021m/z	neg	3-hydroxy caprylic acid	LMFA01050021
1713	7.32_452.1252n	neg	5-[6-hydroxy-2-(4-hydroxyphenyl)-4-[(E)-2-(4-hydroxyphenyl)ethenyl]-1-benzofuran-3-yl]benzene-1,3-diol	HMDB0140527
1714	7.34_505.1388m/z	pos	Acridinium NHS ester	64846
1715	7.43_415.2138m/z	neg	gamma-Asarone	HMDB0029872

1716	7.97_505.1632m/z	pos	Eriotriochin	LMPK12050186
1717	8.01_151.1126m/z	pos	Methyl octynecarboxylate	87543
1718	8.37_369.1723m/z	neg	16-phenoxy tetranor Prostaglandin A2	45407
1719	8.55_209.1543m/z	pos	(S,E)-Lyratol propanoate	HMDB0031869
1720	8.55_305.0809m/z	neg	2,3-Dihydro-2,3-dihydroxy-4-(4-hydroxyphenyl)-1H-phenalen-1-one	HMDB0032848
1721	8.74_477.3710m/z	pos	11-keto-Octacosanoic acid	LMFA01060204
1722	8.95_446.2897m/z	neg	3a,7b,12a-Trihydroxyoxocholanyl-Glycine	LMST05030007
1723	9.29_450.3230m/z	pos	5-trans Latanoprost	45709
1724	9.30_174.9917m/z	pos	3-Sulfinoalanine	HMDB0000996
1725	9.78_171.1010m/z	pos	(+/-)-Ethyl 3-acetoxy-2-methylbutyrate	HMDB0032258
1726	0.68_237.1482m/z	pos	4-Heptyloxybenzoic acid	69931
1727	0.77_127.0380m/z	pos	5-Hydroxymethyl-2-furancarboxaldehyde	HMDB0034355
1728	0.77_565.0628m/z	pos	({6-[5,6-dihydroxy-2-(4-hydroxyphenyl)-7-methoxy-4-oxo-4H-chromen-8-yl]-3,4,5-trihydroxyoxan-2-yl}methoxy)sulfonic acid	HMDB0127146
1729	0.78_234.0504n	neg	Benzo[b]naphtho[2,1-d]thiophene	73542
1730	0.80_263.0842m/z	pos	FKGK 11	45480
1731	0.85_607.1367m/z	neg	(±)-Conen	86377

1732	0.91_643.1082m/z	neg	Fusaroskyrin	HMDB0034314
1733	0.96_433.0149m/z	neg	Allura red AC	HMDB0032884
1734	1.01_241.0694m/z	pos	Dinotefuran	72315
1735	1.21_154.0617m/z	neg	2-Oxoarginine	HMDB0004225
1736	1.21_265.0877m/z	pos	4-Methylburimamide	69230
1737	1.21_319.1223m/z	pos	Didemethylcitalopram	HMDB0060472
1738	1.23_407.0345m/z	pos	Thiamine pyrophosphate	2832
1739	1.24_381.0277m/z	neg	[4-(5,7-dihydroxy-6-methoxy-4-oxo-3,4-dihydro-2H-1-benzopyran-2-yl)phenyl]oxidanesulfonic acid	HMDB0128600
1740	1.43_319.0725m/z	pos	Ipriflavone	HMDB0032987
1741	1.43_426.0849m/z	pos	[4-(5-hydroxy-7-methoxy-8-methyl-4-oxo-4H-chromen-3-yl)-2-methoxyphenyl]oxidanesulfonic acid	HMDB0129590
1742	1.43_786.2574m/z	pos	Aquifoliunine EIII	89387
1743	1.48_277.0891m/z	pos	(2R)-1-O-beta-D-Galactopyranosylglycerol	HMDB0038664
1744	1.65_243.2323m/z	pos	Dodecyl propionate	88298
1745	1.98_298.9983m/z	pos	[2-hydroxy-6-methoxy-4-(prop-2-en-1-yl)phenyl]oxidanesulfonic acid	HMDB0135261
1746	1.98_380.1592n	pos	N-(1-Deoxy-1-fructosyl)tryptophan	HMDB0037847
1747	10.17_379.0930m/z	pos	Dolichin B	LMPK12070015
1748	10.46_361.2622m/z	pos	JWH 210-d9	96637

1749	10.54_265.1482m/z	neg	6,7-Dihydro-4-(hydroxymethyl)-2-(p-hydroxyphenethyl)-7-methyl-5H-2-pyrindinium	89304
1750	10.84_172.1695m/z	pos	(R)-(+)-alpha-Terpineol	LMPR0102090028
1751	10.87_100.0763m/z	pos	5-amino-pentanoic acid	LMFA01100040
1752	10.93_271.2274m/z	neg	(R)-3-Hydroxy-hexadecanoic acid	HMDB0010734
1753	11.90_780.1519m/z	pos	Malonylmalvin	LMPK12010388
1754	12.11_447.3491m/z	pos	24-hydroxy-cholesterol(d6)	41656
1755	12.27_483.3682m/z	neg	3-Oxotetradecanoic acid	HMDB0010730
1756	12.41_277.2173m/z	pos	13-Oxo-ODE	LMFA02000016
1757	13.72_416.0400m/z	pos	6-Methylthioguanosine monophosphate	71266
1758	13.83_574.4609n	pos	DG(15:0/18:4(6Z,9Z,12Z,15Z)/0:0)	HMDB0007077
1759	2.00_424.1347m/z	neg	Moricizine sulfoxide	1346
1760	2.04_262.0399m/z	pos	(2-phenyl-7H-chromen-7-ylidene)oxidanium	HMDB0133410
1761	2.04_275.1134m/z	pos	Pantoyllactone glucoside	95683
1762	2.12_516.2187m/z	pos	Epothilone C	71028
1763	4.41_245.9950m/z	pos	4-Methyl-5-(2-phosphoethyl)-thiazole	3360
1764	4.50_453.0513m/z	pos	Calcium gluconate	66832
1765	4.72_187.0098m/z	neg	Dimethyl sulfone	7236
1766	4.74_366.0665m/z	pos	Dehydro-L-(+)-ascorbic acid dimer	45119
1767	5.25_202.1082m/z	neg	Lentiginosine	68160

1768	5.37_351.2005m/z	pos	trans-p-Menthane-1,7,8-triol 8-glucoside	90206
1769	5.43_313.0439m/z	neg	5'-Phosphoribosyl-N-formylglycinamide	HMDB0001308
1770	5.62_245.0672m/z	neg	(Z)-3-(1-Formyl-1-propenyl)pentanedioic acid	HMDB0033091
1771	5.83_532.3361m/z	pos	LysoPE(0:0/20:0)	HMDB0011481
1772	6.06_399.1138m/z	neg	Tegafur	69467
1773	6.18_543.2823m/z	pos	Dipyridamole	2341
1774	6.57_494.2294m/z	pos	Verruculogen	73523
1775	6.69_172.1697m/z	pos	2E-geraniol	LMPR0102010016
1776	6.81_275.2362m/z	pos	3a,17a-Dihydroxy-5b-androstane	HMDB0000383
1777	6.88_317.1193m/z	pos	8-Hydroxyguanosine	HMDB0002044
1778	6.89_170.9870m/z	neg	3-Hydroxy-4-aminopyridine sulfate	HMDB0061120
1779	6.94_506.2730n	pos	4-Megastigmene-6a,9R-diol 9-[apiosyl-(1->6)-glucoside]	HMDB0041580
1780	7.50_451.0677m/z	neg	3,5,7-Tris(acetyloxy)-2-[4-(acetyloxy)-3-hydroxyphenyl]-4H-1-benzopyran-4-one	50719
1781	7.54_139.1121m/z	pos	(3Z,6Z)-3,6-Nonadienal	HMDB0031152
1782	8.28_641.3530m/z	neg	Arginyl-Phenylalanine	HMDB0028716
1783	8.51_631.2433m/z	pos	Somniferine	HMDB0038585

1784	9.17_282.2070m/z	pos	N-(3-hydroxy-dodecanoyl)-homoserine lactone	LMFA08030013
1785	9.27_100.0760m/z	pos	N-Methyl-a-aminoisobutyric acid	6508
1786	9.52_541.3063m/z	pos	Deoxynojirimycin Tetrabenzyl Ether	45345
1787	0.02_184.9342m/z	neg	Thiocarbamic acid	HMDB0061934
1788	0.61_182.0667m/z	pos	8-Hydroxy-7-methylguanine	58348
1789	0.62_148.9522m/z	neg	Trifluoromethanesulfonic acid	91649
1790	0.66_389.0350m/z	neg	4-iodo-SAHA	64756
1791	0.78_399.1246m/z	neg	Cyclomorusin	HMDB0030687
1792	0.80_232.0468m/z	neg	1-(Malonylamino)cyclopropanecarboxylic acid	87876
1793	0.92_959.3199m/z	neg	3,4,5-trihydroxy-6-{[8-(2-hydroxypropan-2-yl)-2-oxo-4-propyl-2H,8H,9H-furo[2,3-h]chromen-5-yl]oxy}oxane-2-carboxylic acid	HMDB0130128
1794	0.93_229.1227m/z	pos	Curcolone	HMDB0038188
1795	0.95_660.0856m/z	pos	UDP-N-acetyl-3-(1-carboxyvinyl)-D-glucosamine	63224
1796	1.12_167.1046m/z	pos	(3R,5Z)-5-Octene-1,3-diol	HMDB0030368
1797	1.21_136.0611m/z	pos	FAPy-adenine	HMDB0004816
1798	1.21_343.0521m/z	neg	Monomethyl phenylphosphonate	87986
1799	1.21_507.1934m/z	pos	Maltotriitol	44774
1800	1.21_511.1312m/z	pos	dexamethasone phosphate	3977

1801	10.20_253.1785m/z	pos	3-[[5-Methyl-2-(1-methylethyl)cyclohexyl]oxy]-1,2-propanediol	HMDB0036133
1802	10.27_251.1993m/z	pos	Methoprene acid	43446
1803	10.76_423.0417m/z	pos	Bis(glycerophospho)-glycerol	66064
1804	10.76_629.3210m/z	neg	Cytotrienin A	44850
1805	10.79_287.1363m/z	pos	Subaphylline	HMDB0033463
1806	10.87_191.1442m/z	pos	10-fluoro-capric acid	96778
1807	10.98_463.3407m/z	pos	Brassinolide	LMST01140001
1808	11.22_610.3717m/z	pos	PC(16:0/5:0(COOH))	82379
1809	11.35_483.3676m/z	neg	L-Menthyl (R,S)-3-hydroxybutyrate	HMDB0032370
1810	12.00_341.2682m/z	neg	1-Ethylcarbamyl-4-methylpiperazine	2006
1811	12.83_283.1768m/z	pos	Oxymetazoline	HMDB0015070
1812	13.79_397.2219m/z	pos	Isopetasoside	HMDB0029622
1813	14.46_893.5660m/z	pos	PG(20:2(11Z,14Z)/22:2(13Z,16Z))	LMGP04010586
1814	2.00_582.1790m/z	pos	Apiin	HMDB0030843
1815	2.12_353.0945m/z	pos	CAY10415	63083
1816	4.18_145.1219m/z	pos	(3R,7R)-1,3,7-Octanetriol	HMDB0033625
1817	4.18_159.0932m/z	neg	Tryptamine	HMDB0000303
1818	4.25_455.0075m/z	pos	3-hydroxy-2-[3-methoxy-4-(sulfooxy)phenyl]-5-sulfino-3,4-dihydro-2H-1-benzopyran-7-olate	HMDB0127802
1819	4.35_316.1765m/z	pos	Toxin T2 tetrol	HMDB0036159

1820	4.53_279.9887m/z	neg	2-Methyl-4-amino-5-hydroxymethylpyrimidine diphosphate	3426
1821	4.66_475.2228m/z	pos	GW 9578	45313
1822	5.12_480.1812m/z	pos	Vilazodone	85563
1823	5.29_247.0891m/z	pos	2,4-Diphenyl-1-butene	HMDB0032563
1824	5.31_460.2784m/z	pos	PE(P-16:0/0:0)	LMGP02070001
1825	5.36_151.1125m/z	pos	(+/-)-Dihydromintlactone	88269
1826	6.23_170.0364m/z	pos	Tolylacetonitrile	65797
1827	6.47_171.1023m/z	neg	2-Octen-4-one	HMDB0031301
1828	7.39_515.2839m/z	neg	L-Olivosyl-oleandolide	LMPK04000032
1829	7.65_471.2481m/z	pos	Maprotiline glucuronide	1067
1830	8.07_629.3965m/z	pos	Ubiquinone 6	HMDB0036062
1831	8.41_240.1965m/z	pos	N-Lauroylglycine	HMDB0013272
1832	8.48_516.3223m/z	pos	Oleyloxyethyl Phosphorylcholine	63061
1833	8.59_100.0760m/z	pos	Norvaline	85259
1834	9.30_368.9883m/z	neg	dUDP	HMDB0001000
1835	9.30_409.1677m/z	pos	Bakkenolide D	HMDB0034998
1836	9.30_599.2536m/z	pos	Tiliacorine	67873
1837	9.33_445.3463m/z	pos	26:2(5Z,9Z)(25Me)	LMFA01020359
1838	9.70_213.1488m/z	neg	12-hydroxy-3Z-dodecenoic acid	LMFA01050170
1839	9.94_292.9845m/z	neg	D-Ribose 1-diphosphate	66359
1840	0.77_210.1331m/z	pos	5(S),6(R)-7-trihydroxymethyl Heptanoate	44893
1841	0.80_385.1117m/z	neg	Toxoflavine	71343

1842	0.82_283.0886m/z	pos	CAY10554	45242
1843	0.85_403.0681m/z	neg	AG-82	45247
1844	0.91_171.0769m/z	neg	meso-2,6-Diaminoheptanedioate	63450
1845	0.95_363.1070m/z	pos	5,8-dihydroxy-2-(1-hydroxy-3-methoxy-4-oxocyclohexyl)-3,7-dimethoxy-4H-chromen-4-one	HMDB0129296
1846	0.97_133.0135m/z	neg	Pyruvic acid	HMDB0000243
1847	1.17_317.1017m/z	neg	Guibourtinidol-7-methyl ether	LMPK12020008
1848	1.18_636.2209m/z	pos	Neoacrimarine E	95232
1849	1.19_396.2387m/z	pos	Eremopetasitenin C1	HMDB0032770
1850	1.19_404.3010m/z	pos	Mangalkanyl glucoside	HMDB0036015
1851	1.20_355.0760m/z	neg	Cysteinyl-Glycine	85689
1852	1.23_279.0339m/z	neg	2'-Hydroxybiphenyl-2-sulfinate	66515
1853	1.25_411.0430m/z	pos	Ceftibuten	HMDB0015485
1854	1.28_305.0073m/z	neg	5-O-(1-Carboxyvinyl)-3-phosphoshikimate	3454
1855	1.72_221.0851m/z	pos	Isopropyl β-D-ThiogalactoPyranoside	44762
1856	10.21_249.1855m/z	neg	(+)-1(10),4-Cadinadiene	HMDB0035084
1857	10.24_560.2057m/z	pos	Flocoumafen	72474
1858	10.27_500.2879m/z	pos	Mycalamide B	71499
1859	10.32_279.2332m/z	pos	9(S)-HODE	45662
1860	10.35_236.1639m/z	pos	5,7,9,11,13-tetradecapentaenoic acid	LMFA01030258

1861	10.39_468.9945m/z	neg	Pamidronate	1692
1862	10.63_548.0101n	neg	6-{[1-carboxy-8-hydroxy-3,5-dioxo-9-(sulfooxy)-1H,2H,3H,5H-cyclopenta[c]isochromen-7-yl]oxy}-3,4,5-trihydroxyoxane-2-carboxylic acid	HMDB0133722
1863	10.64_603.2876n	pos	Neocasomorphin (1-5)	HMDB0060144
1864	10.74_544.2654m/z	neg	PS(20:4(5Z,8Z,11Z,14Z)/0:0)	LMGP03050007
1865	10.75_337.2755m/z	pos	MG(18:2(9Z,12Z)/0:0/0:0)	HMDB0011568
1866	10.81_656.3183m/z	neg	OKOOA-PS	LMGP20040029
1867	10.90_293.1404n	pos	Koenimbine	HMDB0030210
1868	11.05_557.3216m/z	neg	Tiocarbazil	72659
1869	11.53_285.2260m/z	pos	9-Thiastearic Acid	45061
1870	11.71_341.3064m/z	pos	MG(18:0/0:0/0:0)	HMDB0011131
1871	11.75_608.3209m/z	pos	OKHdiA-PA	LMGP20070026
1872	11.94_283.2636m/z	neg	2-Methyl-4-heptanone	85275
1873	12.35_431.3531m/z	pos	4alpha-Carboxy-5alpha-cholesta-8-en-3beta-ol	HMDB0012166
1874	12.51_461.3312m/z	pos	Dynorphin A (6-8)	HMDB0012932
1875	13.20_810.6062m/z	pos	GlcCer(t18:1(8Z)/20:0(2OH[S]))	LMSP05010044
1876	14.66_754.5460m/z	pos	GlcCer(t18:1(8Z)/16:0(2OH[S]))	LMSP05010042
1877	2.00_477.1073m/z	neg	1-Nitro-5-hydroxy-6-glutathionyl-5,6-dihydronaphthalene	HMDB0060327
1878	4.18_158.0966m/z	pos	8-Isoquinoline methanamine (hydrochloride)	45036
1879	4.18_186.0560m/z	neg	Indoleacrylic acid	HMDB0000734

1880	4.21_265.1237m/z	pos	Ohobanin	LMPK12120414
1881	4.22_304.1762m/z	pos	Phenylalanyl-Arginine	HMDB0028989
1882	4.24_283.0094m/z	neg	Thien-2-ylacetate	65796
1883	4.25_469.0568m/z	pos	(3-{8,8-dimethyl-2-oxo-2H,8H-pyrano[2,3-f]chromen-3-yl}-2-hydroxy-6-methoxyphenyl)oxidanesulfonic acid	HMDB0135728
1884	4.25_478.2023m/z	pos	Gentamicin A2	71865
1885	4.29_136.0215m/z	pos	Benzothiazole	88833
1886	4.41_310.0268m/z	pos	Lamivudine-monophosphate	HMDB0060641
1887	4.73_242.0645m/z	pos	1-Isothiocyanato-7-(methylsulfinyl)heptane	HMDB0038441
1888	5.78_269.1497m/z	neg	Aspergillic acid	68394
1889	6.06_170.0363m/z	pos	Benzenepropanenitrile	HMDB0034236
1890	6.23_387.0640m/z	pos	6'',6''-Dimethyl-3',4'-methylenedioxypyrano[2'',3'':7,8]flavone	48477
1891	6.25_409.2753m/z	pos	(23R)-1α-hydroxy-25,27-didehydrovitamin D3 26,23-lactone	42528
1892	7.06_237.1850m/z	pos	Lubiminol	HMDB0029604
1893	7.12_633.2572m/z	pos	Glucosyl-limonin	66514
1894	7.32_380.1154m/z	pos	Ethacrynic acid M2	2586
1895	8.02_257.2301m/z	pos	2-N-Dodecyltetrahydrothiophene	68819
1896	8.04_610.3592m/z	neg	JSTX-3	69746
1897	8.08_648.3392m/z	pos	Hydroxydestruxin B	HMDB0040135

1898	8.52_353.1784m/z	pos	Etiocholanolone sulfate	LMST05020044
1899	8.56_299.2405m/z	pos	2-mercapto-octadecanoic acid	LMFA01130002
1900	9.39_401.3444m/z	pos	22b-Hydroxycholesterol	HMDB0004035
1901	9.71_364.0854m/z	pos	4-Methylthiobutyl-desulfoglucosinolate	64514
1902	0.88_189.0874m/z	neg	Threoninyl-Alanine	HMDB0029054
1903	0.89_143.0819m/z	neg	N6-Hydroxy-L-lysine	63466
1904	0.94_361.0874m/z	neg	Meticillin	HMDB0015541
1905	1.08_457.1713m/z	pos	6-Feruloylglucose 2,3,4-trihydroxy-3-methylbutylglycoside	HMDB0036214
1906	1.10_257.2483m/z	pos	4,8,12-trimethyl-tridecanoic acid	LMFA01020249
1907	1.22_295.0643m/z	pos	(2-hydroxy-1,2-diphenylethoxy)sulfonic acid	HMDB0135202
1908	1.28_464.9595m/z	neg	Inositol 1,3,4-trisphosphate	HMDB0001143
1909	1.48_244.9532m/z	neg	Thioxanthine monophosphate	HMDB0060876
1910	10.13_452.3172m/z	pos	17-phenyl trinor PGF2α cyclohexyl amide	64899
1911	10.35_597.2766m/z	pos	Herbimycin	68926
1912	10.52_677.1489m/z	pos	3,4'-Dihydroxy-7,3',5'-trimethoxyflavone 3-galactosyl-(1->4)-xyloside	LMPK12111575
1913	10.84_451.2117m/z	pos	Sofalcone	HMDB0042013
1914	10.86_313.2746m/z	pos	MG(i-16:0/0:0/0:0)	HMDB0072844
1915	11.05_239.1645m/z	pos	Geranyl acetoacetate	92981
1916	11.05_599.4429m/z	pos	PA(P-16:0/15:1(9Z))	LMGP10030006

1917	11.49_355.2236m/z	pos	3,6-Ditigloyloxytropan-7-ol	HMDB0029325
1918	11.65_191.1433m/z	pos	beta-Damascenone	85264
1919	11.71_630.4835m/z	pos	PE(14:0/P-16:0)	HMDB0008850
1920	12.54_337.1568m/z	pos	4-Oxoretinal	HMDB0012794
1921	13.20_727.5725m/z	pos	SM(d18:2/18:1)	LMSP03010047
1922	13.40_469.3324m/z	pos	(3beta,15alpha,22S,24E)-3,15,22-Trihydroxylanosta-7,9(11),24-trien-26-oic acid	HMDB0035295
1923	13.78_489.3569m/z	pos	Pitheduloside I	89673
1924	13.92_545.4223m/z	pos	PAF C-16-d4	96561
1925	2.14_362.1212m/z	neg	N-(1-Deoxy-1-fructosyl)histidine	HMDB0037839
1926	2.85_146.0604m/z	pos	4-Oxo-1-(3-pyridyl)-1-butanone	HMDB0062406
1927	5.01_381.0749m/z	neg	Brassicanal A	68795
1928	5.75_180.9882m/z	neg	3,5-DINITROCATECHOL (OR-486)	44188
1929	6.24_138.0189m/z	neg	2-Aminomuconic acid	HMDB0001241
1930	7.20_236.1141m/z	pos	Penciclovir	HMDB0014444
1931	7.32_465.1241m/z	neg	Secogalioside	69151
1932	7.34_425.1406m/z	pos	Benzyl O-[arabinofuranosyl-(1->6)-glucoside]	95898
1933	7.34_432.1686m/z	pos	Glisoxepide	85491
1934	7.37_334.1311m/z	pos	O-Methylisoetharine sulfate	846
1935	7.47_295.1306m/z	neg	Caffeoylputrescine	3380
1936	7.60_464.3002m/z	neg	N-palmitoyl tyrosine	LMFA08020098
1937	7.91_491.1174m/z	neg	Glycitin	HMDB0002219

1938	7.95_409.2741m/z	pos	Azafrin	67076
1939	7.97_304.1050m/z	pos	S-Glutaryldihydrolipoamide	HMDB0006832
1940	8.38_303.1422m/z	pos	Arabinoside	87702
1941	8.38_539.2479m/z	neg	Flumethasone Pivalate	HMDB0014801
1942	8.42_237.1853m/z	pos	10alpha-4,5-Seco-11-eudesmene-4,5-dione	HMDB0032681
1943	8.97_527.2924m/z	pos	Desmosine	HMDB0000572
1944	9.29_663.2295m/z	neg	Nirurin	LMPK12140614
1945	9.70_179.1441m/z	neg	11-Dodecenoic acid	HMDB0032248
1946	9.78_194.0819m/z	neg	2-Aminopropiophenone	2011
1947	9.82_624.2407m/z	pos	Tigecycline	69251
1948	0.67_375.0072m/z	pos	beta-D-3-[5-Deoxy-5-(dimethylarsinyl)ribofuranosyloxy]-2-hydroxy-1-propanesulfonic acid	HMDB0032686
1949	0.71_824.7334m/z	pos	GlcCer(d18:0/26:0)	41621
1950	0.76_608.1864m/z	pos	AKLAVINE	43581
1951	0.78_368.1228m/z	neg	Bialaphos	63603
1952	0.80_403.1158m/z	pos	Gibberellin A55	HMDB0033420
1953	0.82_390.1993m/z	pos	Clavamycin F	71660
1954	0.93_411.0328m/z	neg	2-Hydroxy-4-trifluoromethyl benzoic acid	HMDB0060715
1955	0.96_201.0007m/z	pos	S-2-Propenyl 2-propene-1-sulfonothioate	HMDB0039940
1956	0.96_448.9857m/z	neg	C.I. Pigment Yellow 100	HMDB0037722

1957	0.98_295.0204m/z	neg	Blue pigment	63889
1958	1.19_580.1677m/z	pos	3,4,5-trihydroxy-6-{[7-hydroxy-4-oxo-2-phenyl-8-(3,4,5-trihydroxyoxan-2-yl)-4H-chromen-5-yl]oxy}oxane-2-carboxylic acid	HMDB0127263
1959	10.08_237.1842m/z	pos	(4S)-Dihydrocurcumenone	HMDB0033083
1960	11.33_528.3092m/z	neg	LysoPE(0:0/22:4(7Z,10Z,13Z,16Z))	HMDB0011493
1961	11.35_608.3926m/z	pos	OON-PA	LMGP20070023
1962	11.53_340.2627n	pos	PGE1 alcohol	LMFA03010053
1963	11.64_519.4059m/z	pos	(20R)-24-Hydroxygeminivitamin D3	42557
1964	12.45_416.3688m/z	pos	(+/-)N-(2-fluro-ethyl)-2,16,16-trimethyl-5Z,8Z,11Z,14Z-tricosatetraenoyl amine	LMFA08020067
1965	13.20_133.1008m/z	pos	(S)-2-methyl-5-(1-Methylethenyl)-2-cyclohexen-1-one	HMDB0061788
1966	13.65_405.3742m/z	pos	27:3(5Z,9Z,20Z)	LMFA01030903
1967	14.00_766.5783m/z	pos	2-Hydroxy-C18-cerebroside	475
1968	15.83_281.0534m/z	pos	2-Amino-3-hydroxy-5-nitrobenzophenone	1572
1969	4.39_181.0611m/z	pos	Nicotinuric acid	HMDB0003269
1970	4.50_152.0172m/z	pos	Thiobenzamide S,S-dioxide	71173
1971	4.50_226.0176m/z	pos	N-acetyl-2-carboxy Benzenesulfonamide	45011

1972	5.41_379.1936m/z	neg	9-Fluoro-17-methyl-11-oxotestosterone	70211
1973	5.66_173.0826m/z	neg	2-Propylglutaric acid	2998
1974	6.38_433.2066m/z	neg	Alanyl-Glutamine	HMDB0028685
1975	6.41_280.1164m/z	neg	Saxitoxin	69686
1976	7.12_179.1429m/z	pos	Propofol	2155
1977	7.23_249.1136m/z	pos	SB 200646	69188
1978	7.59_137.1326m/z	pos	2-decenal	75331
1979	8.10_345.0907m/z	pos	Piretanide	1978
1980	8.45_427.2144m/z	neg	4-Hydroxytacrine	2618
1981	8.55_307.0987m/z	pos	Isomethiozin	72849
1982	9.32_322.1221m/z	pos	N-[[(hexahydrocyclopenta[c]pyrrol-2(1H)-yl)amino]carbonyl]-4-(hydroxymethyl)-Benzenesulfonamide	3115
1983	9.52_445.2361m/z	pos	7-hydroxycoumarinyl-γ-Linolenate	64760
1984	9.59_534.3205m/z	pos	PS(20:1(11Z)/0:0)	LMGP03050020
1985	9.62_252.1724m/z	pos	Tridecanamide	LMFA08010012
1986	0.02_247.9432m/z	pos	Bismuth	HMDB0002196
1987	0.62_333.1412m/z	pos	Zanamivir	HMDB0014698
1988	0.69_403.0304m/z	neg	Distemonanthin	LMPK12113389
1989	0.81_453.0316m/z	neg	L-Glutamyl 5-phosphate	63480
1990	0.84_635.2136m/z	neg	Catechin 7,4'-dimethyl ether	47359
1991	0.92_209.0658m/z	neg	Sedoheptulose	3344

1992	0.98_428.9707m/z	neg	DNDS	LMPK13090017
1993	1.22_177.9941m/z	pos	3-AMINOPROPANESULPHONIC ACID	43475
1994	1.29_250.9550m/z	neg	Methyl methanethiosulfonate	87955
1995	1.96_167.0206m/z	neg	Diethyl disulfide	HMDB0029572
1996	10.46_167.1434m/z	pos	Limonene aldehyde	86834
1997	10.51_643.1746m/z	pos	2-{4-[(6-carboxy-3,4,5-trihydroxyoxan-2-yl)oxy]-3-hydroxyphenyl}-3,7-dihydroxy-5-{[3,4,5-trihydroxy-6-(hydroxymethyl)oxan-2-yl]oxy}-1λ⁴-chromen-1-ylium	HMDB0125133
1998	10.55_256.1916m/z	neg	N-lauroyl glycine	LMFA08020261
1999	10.77_281.1390m/z	neg	Eremopetasidione	HMDB0040778
2000	10.84_179.1099m/z	pos	Methional diethyl acetal	HMDB0041608
2001	10.89_531.3290m/z	pos	3α,7α,12α-trihydroxy-27-carboxymethyl-5β-cholestan-26-oic acid	43164
2002	10.90_268.1518m/z	pos	Pivaloylcarnitine	HMDB0041993
2003	10.90_296.1486m/z	pos	11-Hydroxy-12-methoxydihydrokawain	HMDB0030766
2004	11.15_459.3106m/z	pos	Polyporusterone C	HMDB0038497
2005	11.54_435.3127m/z	pos	NH125	45320
2006	11.54_457.2345m/z	neg	1-Lyso-2-arachidonoyl-phosphatidate	HMDB0012496
2007	11.64_861.6227n	pos	PC(22:2(13Z,16Z)/20:4(5Z,8Z,11Z,14Z))	LMGP01012058

2008	11.87_457.3528m/z	pos	Byrsonic acid	LMFA01050424
2009	12.48_487.4001m/z	pos	Arachidonyl carnitine	HMDB0006455
2010	12.68_367.1916m/z	pos	Armillarivin	HMDB0038917
2011	12.91_535.4026m/z	pos	Nb-Tricosanoyltryptamine	HMDB0040818
2012	13.01_433.3644m/z	pos	2-Hydroxyhexadecanoylcarnitine	LMFA07070113
2013	13.80_842.6001m/z	pos	PE(16:0/24:0)	HMDB0008947
2014	15.75_297.0844m/z	pos	flunixin	4044
2015	2.02_195.0404m/z	neg	(S)-ACPA	69626
2016	2.27_247.0494m/z	pos	L-Kynurenine	HMDB0000684
2017	5.31_680.4425m/z	pos	Prostaglandin E2-biotin	44962
2018	5.48_534.3524m/z	pos	1-O-(2-methoxyhexadecyl)-sn-glycerol-3-phosphocholine	LMGP01060042
2019	5.70_239.0856m/z	neg	Methiuron	72828
2020	5.82_144.0449m/z	neg	4-(3-Pyridyl)-3-butenoic acid	6236
2021	7.59_403.0070m/z	neg	Ribavirin 5'-diphosphate	2298
2022	8.16_367.1565m/z	neg	Porson	87208
2023	8.16_566.2770m/z	neg	Dihydrodeoxystreptomycin	2507
2024	9.30_333.1272m/z	pos	Mannopine	71286
2025	9.35_346.9916m/z	neg	Phenyl sulfate	1828
2026	9.39_201.1491m/z	neg	p-Menthan-3-ol	HMDB0035765
2027	9.51_506.2444m/z	pos	Malyngamide L	65450
2028	9.66_351.1337m/z	neg	3-Oxo-14,15-dehydrorhazinilam	HMDB0039105
2029	9.67_478.2001m/z	pos	NVP-AEW541	45579

2030	9.90_254.9964m/z	pos	[(7-hydroxy-1-oxo-1H-isochromen-3-yl)methoxy]sulfonic acid	HMDB0128634
2031	0.04_379.0868m/z	pos	Bay-K-8644	69687
2032	0.14_341.0234m/z	pos	3-Deoxy-D-manno-octulosonate 8-phosphate	63652
2033	0.69_929.7825n	pos	PC(24:0/22:0)	60221
2034	0.73_698.4743m/z	neg	PE(18:3(9Z,12Z,15Z)/15:0)	HMDB0009153
2035	0.80_202.0613m/z	pos	(S)-F-Willardiine	69624
2036	0.81_261.0720m/z	neg	8-Hydroxyalanylclavam	71633
2037	0.83_135.0291m/z	neg	L-Lactic acid	HMDB0000190
2038	0.92_445.0393n	pos	D-Gluconic acid Mn(II) salt	92165
2039	1.08_712.2386m/z	pos	6-Methoxy-2-naphthylacetic acid	HMDB0060787
2040	1.21_401.1650m/z	neg	BAY-41-8543	64714
2041	1.23_671.1053m/z	neg	(5-{8-[3,7-dihydroxy-2-(3-hydroxyphenyl)-3,4-dihydro-2H-1-benzopyran-4-yl]-3,5,7-trihydroxy-3,4-dihydro-2H-1-benzopyran-2-yl}-2-hydroxyphenyl)oxidanesulfonic acid	HMDB0135098
2042	1.97_305.0976m/z	neg	Fluconazole	HMDB0014342
2043	10.18_463.2181m/z	neg	2-Oxo-10-methylthiodecanoic acid	64510
2044	10.43_619.3263m/z	pos	PKOOA-PG	LMGP20060006
2045	10.55_350.1244m/z	neg	Rhizocticin A	63578

2046	10.63_442.9945m/z	neg	Melarsoprol	66778
2047	10.71_506.3597m/z	pos	LysoPC(18:0)	HMDB0010384
2048	10.76_361.2767m/z	pos	MG(0:0/20:4(8Z,11Z,14Z,17Z)/0:0)	HMDB0011549
2049	10.77_579.0067m/z	neg	5-(4-Acetoxy-3-oxo-1-butynyl)-2,2'-bithiophene	93147
2050	11.29_309.1729m/z	neg	7-hydroxygranisetron	HMDB0061062
2051	11.80_363.2533m/z	neg	2,2,7,7-Tetramethyl-1,6-dioxaspiro[4.4]non-3-ene	86658
2052	12.10_427.3300m/z	pos	1-dimethylarsinoyl-tricosane	LMFA00000033
2053	12.12_261.1875m/z	pos	2-ethyl-1,5-dimethyl-3,3-diphenylpyrrolinium (EDDP)	HMDB0060931
2054	12.27_361.2271m/z	pos	N-Didesmethyl-tamoxifen	HMDB0061086
2055	12.68_369.1871m/z	pos	9-(beta-D-Ribofuranosyl)zeatin	HMDB0030388
2056	14.43_850.6660m/z	pos	PC(22:0/P-18:1(11Z))	HMDB0008556
2057	14.54_868.5122n	pos	PI(15:0/22:6(4Z,7Z,10Z,13Z,16Z,19Z))	LMGP06010128
2058	2.01_269.0474n	neg	Sulfamethoxazole N4-hydroxylamine	HMDB0013852
2059	2.06_219.0085m/z	neg	SQ 26180	71381
2060	4.31_319.1026m/z	neg	Glutaminyl-Glutamate	85710
2061	4.51_348.9948m/z	pos	Diflubenzuron	70053
2062	4.70_525.0340m/z	neg	Quercetin 3-(3''-sulfatoglucoside)	LMPK12112165
2063	5.24_406.0646m/z	pos	Gluconasturtiin	HMDB0038423
2064	5.34_339.0421m/z	pos	Sodium 3-ethyl-7-isopropyl-1-azulenesulfonate	69570

2065	5.39_315.1332m/z	pos	Longifolonine	67811
2066	5.79_561.2025m/z	neg	Juzirine	HMDB0033954
2067	5.93_240.0425m/z	neg	p-Nitrophenyl-O-ethyl ethylphosphonate	69527
2068	6.02_267.0038m/z	pos	Mevalonic acid-5P	HMDB0001343
2069	6.29_528.2625m/z	neg	N-[(3a,5b,7b)-7-hydroxy-24-oxo-3-(sulfoxy)cholan-24-yl]-Glycine	LMST05030012
2070	7.25_253.1345m/z	neg	Ergoline-8-methanol, 10-methoxy-, (8b)-	1516
2071	7.88_427.1305m/z	neg	Farnesyl pyrophosphate	403
2072	8.13_512.2687m/z	neg	Sulfoglycolithocholic acid	LMST05030015
2073	8.14_511.3738m/z	pos	Isorenieratene/ (Leprotene)	LMPR01070124
2074	8.19_214.9935m/z	neg	Salithion	72740
2075	8.39_465.2472m/z	neg	Etiocholan-3alpha-ol-17-one 3-glucuronide	LMST05010014
2076	9.15_209.1543m/z	neg	7-methoxy-dodec-4-enoic acid	LMFA01080012
2077	9.17_400.3380n	pos	7a-Hydroxy-cholestene-3-one	HMDB0001993
2078	9.90_319.2976m/z	pos	Thromboxane	HMDB0003208
2079	9.94_320.2364n	pos	19(R)-HETE	45085
2080	0.03_205.0870m/z	pos	Edrophonium	HMDB0015145
2081	0.61_455.2702m/z	pos	Tafluprost ethyl amide	96555
2082	0.62_319.1246m/z	pos	gamma-Glutamyl-beta-(isoxazolin-5-on-2-yl)alanine	95006
2083	0.69_199.0692m/z	neg	Diphenylmethylphosphine	518
2084	0.69_393.0860m/z	pos	Bensulfuron-methyl	68703

2085	0.76_261.01 14m/z	pos	tert-Butylbicyclophosphorothionate	73434
2086	0.80_487.08 20m/z	pos	Quercetin 3-galactoside	HMDB0030775
2087	0.81_463.06 79m/z	neg	11-O-Demethylpradinone I	66535
2088	0.81_818.30 19m/z	neg	Docetaxel M4	741
2089	0.83_349.02 22m/z	pos	Mefluidide	72517
2090	0.96_188.07 38m/z	pos	2,5-Dimethyl-3-furanthiol acetate	HMDB0032234
2091	1.17_333.07 41m/z	neg	Phenylacetothiohydroximate	64521
2092	10.70_433.3 225n	pos	N-heneicosanoyl taurine	LMFA08020250
2093	10.75_312.1 375m/z	pos	Tolazamide	HMDB0014977
2094	10.75_753.1 631m/z	pos	Isorientin 4'-O-glucoside 2''-O-p-hydroxybenzoagte	48980
2095	10.77_466.9 967m/z	neg	5-Hydroxy-1-(4-sulfophenyl)-4-[(E)-(4-sulfophenyl)diazenyl]-1H-pyrazole-3-carboxylic acid	HMDB0059914
2096	10.80_585.3 425m/z	pos	PI(P-18:0/0:0)	LMGP06070002
2097	10.85_614.3 456m/z	neg	OHOOA-PE	LMGP20020046
2098	10.87_474.3 631m/z	pos	N-nervonoyl taurine	LMFA08020257
2099	10.90_515.3 186m/z	pos	IC202B	LMFA08020183
2100	11.27_281.1 608m/z	pos	Pentoxifylline alcohol	1768
2101	11.54_433.3 044m/z	pos	N-oleoyl glutamine	75510
2102	11.57_262.2 300n	pos	6-[3]-ladderane-1-hexanol	LMFA05000065
2103	11.78_185.1 177m/z	neg	3-Oxodecanoic acid	HMDB0010724
2104	12.32_517.3 889m/z	neg	Theonellasterol D	LMST01040182
2105	12.43_495.3 901m/z	pos	Leucettamol A	LMSP01080040

2106	14.43_343.2644m/z	pos	Docosahexaenoic Acid methyl ester	44958
2107	2.27_205.9973m/z	pos	2,3-Dimercaptopropane-1-sulfonic acid	68691
2108	4.09_205.0295m/z	neg	Hydroxyanthraquinone	65876
2109	4.18_649.2123m/z	neg	Citalopram (propionic acid derivative)	1785
2110	4.29_337.1526m/z	pos	Ergoline-1-carboxaldehyde, 8-(hydroxymethyl)-10-methoxy-6-methyl-, (8b)-	1520
2111	4.49_197.0423m/z	neg	Cystamine	55
2112	4.69_158.0960m/z	pos	N-Acetyltranylcypromine	2896
2113	5.43_339.0378m/z	neg	Gallic acid	HMDB0005807
2114	5.67_340.2710m/z	pos	(-)-Ormosanine	68570
2115	6.16_375.1183m/z	neg	N-Acetylserotonin glucuronide	HMDB0060833
2116	6.29_363.2194m/z	pos	PF-750	45298
2117	7.16_314.2341m/z	pos	3-hydroxydecanoyl carnitine	HMDB0061636
2118	7.59_225.1492m/z	neg	Tricycloekasantalol	HMDB0039339
2119	7.61_331.1750m/z	neg	7-Hydroxyterpineol 8-glucoside	HMDB0033019
2120	7.78_409.2750m/z	pos	(23S)-1alpha-hydroxy-25,27-didehydrovitamin D3 26,23-lactone	LMST03020599
2121	7.88_415.1632m/z	pos	O-Desmethylcarvedilol	HMDB0013949
2122	7.97_617.1280m/z	pos	Rhoifolin	HMDB0038848
2123	8.01_303.1420m/z	pos	(R)-Lisofylline	45571

2124	8.30_299.2413m/z	pos	Octadecanoic acid, 2-mercapto-; alpha-Mercaptostearic acid	74887
2125	8.77_157.0858m/z	pos	Ethyladipic acid	45928
2126	9.07_625.3578m/z	neg	ecdysone 25-O-D-glucopyranoside	LMST01010192
2127	9.33_472.2714m/z	pos	Isomigrastatin	71110
2128	9.48_479.3002m/z	neg	3alpha,7alpha,12alpha-Trihydroxy-5beta-23E-cholestan-26-oic acid	LMST04030199
2129	9.77_276.1958m/z	pos	(all-E)-1,8,10-Heptadecatriene-4,6-diyne-3,12-diol	HMDB0039737
2130	0.59_238.2019m/z	pos	1,2-Bis(1-ethoxyethoxy)propane	HMDB0037163
2131	0.64_402.3710m/z	pos	Type IV cyanolipid 22:0 ester	LMFA07030004
2132	0.69_775.3430m/z	neg	beta-D-Glucopyranosyl-11-hydroxyjasmonic acid	HMDB0039964
2133	0.72_912.5596m/z	pos	PI(17:1(9Z)/22:6(4Z,7Z,10Z,13Z,16Z,19Z))	LMGP06010245
2134	0.81_783.1894m/z	neg	Tiotropium	HMDB0015479
2135	0.82_543.1096m/z	pos	6-Caffeoylsucrose	90768
2136	0.93_318.0620m/z	pos	beta-nicotinamide D-ribonucleotide	HMDB0059645
2137	1.23_545.0445m/z	neg	Orange B	HMDB0037522
2138	10.12_271.0891m/z	pos	5,6-Dihydrouridine	5483
2139	10.22_459.2014m/z	neg	Armillaripin	HMDB0030404
2140	10.27_558.2067m/z	pos	Zafirlukast	HMDB0014689
2141	10.38_579.3051m/z	neg	Diisopentyl thiomalate	LMFA07010835

2142	10.43_311.2222m/z	pos	Undecanoylcholine	LMFA07011030
2143	10.49_591.3533m/z	neg	S-Farnesyl Thioacetic Acid	43440
2144	10.54_478.9972m/z	neg	Ponceau SX	73193
2145	10.76_568.2681m/z	neg	PS(22:6(4Z,7Z,10Z,13Z,16Z,19Z)/0:0)	LMGP03050013
2146	10.78_623.4445m/z	pos	OH-Spheroidenone	LMPR01070151
2147	10.90_523.3046m/z	pos	Ciclesonide	85511
2148	11.11_191.1461m/z	pos	2-(Octylthio)ethanol	72625
2149	11.26_580.4222m/z	pos	Cholesterol glucuronide	LMST05010043
2150	11.37_407.2594m/z	pos	Nandrolone phenpropionate	HMDB0015119
2151	11.54_540.3959m/z	pos	Oleic Acid-biotin	45287
2152	12.31_283.1768m/z	pos	Ropinirole	2361
2153	15.09_703.5786m/z	pos	Campesteryl elaidate	91433
2154	15.10_610.4374m/z	pos	Arachidonic Acid Leelamide	45136
2155	15.46_431.3524m/z	pos	5alpha,6beta-dihydroxy-24-methylenecholestan-3-one	LMST01031062
2156	2.26_233.0614m/z	pos	Thiolactomycin	68888
2157	3.80_145.1222m/z	pos	2-Ethylhexanoic acid	HMDB0031230
2158	4.55_273.0067m/z	neg	{4-[(1E)-3-oxoprop-1-en-1-yl]phenyl}oxidanesulfonic acid	HMDB0135289
2159	4.73_152.0172m/z	pos	1,2-Benzisothiazol-3(2H)-one	HMDB0034413
2160	5.28_446.3017m/z	pos	Alpha-linolenyl carnitine	58390
2161	5.31_232.0610m/z	neg	3-amino-2-naphthoic acid	34509
2162	5.71_488.3085m/z	pos	LysoPE(20:2(11Z,14Z)/0:0)	HMDB0011513

2163	5.81_256.1550m/z	neg	N-Butylscopolamine metabolite	2399
2164	6.06_620.3876m/z	pos	1-(2-methoxy-docosanyl)-sn-glycero-3-phosphoserine	LMGP03060021
2165	6.57_385.1671m/z	neg	5-Deoxymyricanone	87204
2166	6.91_498.2737m/z	pos	Alangimarckine	64421
2167	7.66_550.2446m/z	neg	Endoxifen O-glucuronide	HMDB0060622
2168	8.35_651.2667m/z	pos	Myricanol 5-[arabinosyl-(1->6)-glucoside]	HMDB0036527
2169	8.41_550.2207m/z	pos	2-amino-4-({1-[(carboxymethyl)-C-hydroxycarbonimidoyl]-2-[(1,3-dihydroxy-2-pentyl-1-phenylpropan-2-yl)sulfanyl]ethyl}-C-hydroxycarbonimidoyl)butanoic acid	HMDB0126539
2170	9.46_212.1462m/z	pos	2-Heptyl-4,5-dimethylthiazole	94633
2171	9.46_329.1699n	neg	Hydroxyethylpromethazine	HMDB0240229
2172	9.60_440.3389m/z	pos	Cholic Acid Methyl Ester	73574
2173	0.61_267.0297m/z	pos	4-phenylbutanic acid-O-sulphate	HMDB0059983
2174	0.70_838.7064m/z	neg	PC(24:0/P-18:0)	HMDB0008785
2175	0.93_313.0643m/z	neg	Vat Yellow 4	73222
2176	0.96_231.0611m/z	neg	Kifunensine	45180
2177	1.21_273.0993n	pos	L-Thyronine	HMDB0000667

2178	1.21_566.2027n	pos	6-{[10-butanoyl-3-hydroxy-6-(2-hydroxypropyl)-2,2-dimethyl-8-oxo-2H,3H,4H,8H-pyrano[3,2-g]chromen-5-yl]oxy}-3,4,5-trihydroxyoxane-2-carboxylic acid	HMDB0130103
2179	1.22_233.9840m/z	pos	Carbocysteine sulfoxide	1527
2180	10.11_212.1460m/z	pos	4-Ethyl-2-heptylthiazole	94654
2181	10.12_382.0098n	neg	Olsalazine sulfate	1631
2182	10.38_498.2616m/z	neg	LysoPE(20:5(5Z,8Z,11Z,14Z,17Z)/0:0)	HMDB0011519
2183	10.51_275.1294m/z	pos	SB 206553	69189
2184	10.57_503.2124m/z	pos	8-Hydroxyondansetron glucuronide	1654
2185	10.68_292.1530m/z	pos	2-(1-hydroxy-3-phenylpropyl)-5-methoxybenzene-1,3-diol	HMDB0140889
2186	10.78_539.3183m/z	pos	27-nor-24S-methylcholestan-3beta,4beta,5alpha,6alpha,7beta,8beta,14alpha,15alpha,24-nonol	84028
2187	10.94_477.3208m/z	neg	Rockogenin	LMST01080017
2188	10.98_225.0661m/z	pos	2-Amino-5-nitrobenzophenone	1570
2189	11.05_267.1957m/z	neg	(-)-alpha-Bisabolol	LMPR0103060001
2190	11.05_268.1514m/z	pos	Isovalerylcarnitine	HMDB0000688
2191	11.84_535.3996m/z	neg	Aragusteroketal	LMST01110016
2192	11.89_276.1602m/z	neg	(2E,4E,6Z)-2,4,6-Decatrienoic acid dehydropiperidide	HMDB0033530
2193	12.62_431.3156m/z	neg	Shibic acid	LMFA01030831

2194	12.64_790.5941m/z	pos	PS(O-18:0/19:1(9Z))	LMGP03020033
2195	12.70_326.3077m/z	pos	Eicosadienoic acid	HMDB0005060
2196	12.85_437.0330m/z	pos	Sulfasalazine	HMDB0014933
2197	13.20_784.1713m/z	pos	Cyanidin 3-O-dimalonyl-laminaribioside	86120
2198	2.00_292.0827m/z	neg	Dhurrin	63619
2199	4.19_483.2518m/z	pos	1-(4Z,7Z,10Z,13Z,16Z,19Z-docosahexaenoyl)-glycero-3-phosphate	HMDB0062307
2200	4.25_398.1171n	pos	Methyl acrylate-divinylbenzene, completely hydrolyzed, copolymer	HMDB0032389
2201	4.25_683.0670m/z	neg	4,13,14-trihydroxy-9-oxo-8,17-dioxatetracyclo[8.7.0.0²,⁷.0¹¹,¹⁶]heptadeca-1(10),2,4,6,11(16),12,14-heptaen-5-yl acetate	HMDB0128437
2202	5.03_295.1268n	neg	N6,N6-Dimethyladenosine	65975
2203	5.26_310.2030n	pos	aminopentamide	3998
2204	5.31_327.2131m/z	pos	2,15,16-trihydroxy palmitic acid	35476
2205	5.83_672.3077m/z	neg	Bouillonamide B	65413
2206	6.12_179.1425m/z	pos	Methyl 2-undecynoate	LMFA07010946
2207	6.18_346.2174n	pos	(+)-Calycanthine	HMDB0029561
2208	6.99_467.1888m/z	neg	Glucosylgalactosyl hydroxylysine	HMDB0000585
2209	7.04_464.3003m/z	neg	Stearidonyl carnitine	LMFA07070106

2210	7.72_471.2397m/z	neg	DIHYDRO-7-DESACETYLDEOXYGEDUNIN	43755
2211	7.88_489.1159m/z	neg	L-2-Aminoadipate adenylate	HMDB0006941
2212	7.97_302.0764n	pos	Hesperetin	HMDB0005782
2213	8.32_315.1371m/z	pos	Buddledin A	67850
2214	8.44_318.1238m/z	pos	Desethylene-N-acetylnorfloxacin	1603
2215	9.06_199.0975m/z	pos	Glu-P-1	72965
2216	0.59_294.0792m/z	pos	Suprofen S-oxide	HMDB0060924
2217	0.72_956.4788m/z	neg	CDP-DG(16:1(9Z)/18:2(9Z,12Z))	HMDB0115952
2218	0.77_301.1129m/z	neg	Echothiophate	HMDB0015190
2219	0.80_462.0509m/z	pos	Glucolimnanthin	66955
2220	0.83_351.0179m/z	neg	(-)-Epicatechin sulfate	HMDB0012467
2221	0.83_449.1490m/z	neg	Methyl 3-carbazolecarboxylate	HMDB0029744
2222	0.84_649.1056m/z	neg	Pelargonidin 3-O-3'',6''-O-dimalonylglucoside	64249
2223	0.89_427.0987m/z	pos	2-(3,4-dimethoxyphenyl)-5,7-dihydroxy-3,6,8-trimethoxy-4H-chromen-4-one	HMDB0136121
2224	0.93_190.0173m/z	neg	3,4-Dehydrothiomorpholine-3-carboxylate	66183
2225	1.23_177.0352m/z	pos	1-(5-Methyl-2-thienyl)-1-propanone	HMDB0040231
2226	1.23_301.0142m/z	neg	Erosnin	HMDB0029465
2227	1.23_695.0656m/z	neg	Bisnorbadioquinone A	HMDB0033267
2228	1.46_281.0353m/z	neg	Tazobactam	HMDB0015544

2229	10.03_198.1281m/z	pos	trans-Stilbene	96322
2230	10.08_409.2737m/z	pos	(23R)-1alpha-hydroxy-25,27-didehydrovitamin D3 26,23-lactone	LMST03020600
2231	10.12_428.0092n	neg	Ticarcillin disodium	69762
2232	10.16_566.3842m/z	pos	PC(10:0/10:0)	LMGP01010380
2233	10.76_272.1620m/z	pos	N,O-Didesmethylvenlafaxine	HMDB0060852
2234	11.34_449.3612m/z	pos	Hexacosanedioic acid	LMFA01170040
2235	12.15_467.3736m/z	pos	6alpha-Hydroxycastasterone	LMST01030128
2236	12.39_441.3575m/z	neg	DG(8:0/15:0/0:0)	HMDB0092917
2237	13.05_359.1749m/z	pos	4-Chloro-17alpha-methyl-17beta-hydroxy-4-androsten-3-one	70830
2238	13.79_450.3701m/z	pos	C-8 Ceramine	43406
2239	14.06_647.5166m/z	pos	CE(15:1)	HMDB0060056
2240	14.15_715.5783m/z	pos	PE-Cer(d14:1(4E)/24:0(2OH))	LMSP03020064
2241	2.15_411.1293m/z	neg	2,3-dihydro-2-oxo-1H-Benzimidazole-1-propanoic acid	2386
2242	3.18_241.0997m/z	pos	(±)-Anisoxide	87068
2243	4.19_430.1617n	pos	3,7-Dihydroxy-2-[3-(4-hydroxy-3-methylbutyl)-4-methoxyphenyl]-5,6-dimethoxy-4H-1-benzopyran-4-one	LMPK12112858
2244	4.35_111.1168m/z	pos	(Z)-5-Octen-1-ol	HMDB0031298

2245	5.04_234.0817m/z	pos	Bis(1-aziridinyl)morpholinophosphine sulfide	73052
2246	5.25_295.1772n	pos	Serratanidine	67995
2247	6.67_234.9857m/z	neg	1,3-Benzenedisulfonamide	69857
2248	6.92_313.2262m/z	pos	Normethadone	1185
2249	6.95_526.2467m/z	neg	N-acetyl-LTE4	LMFA03020008
2250	7.50_345.1441m/z	neg	coumarin-SAHA	64949
2251	7.66_482.1811m/z	neg	Adefovir Dipivoxil	85417
2252	9.86_263.2118m/z	pos	TOLPERISONE	43502
2253	0.61_229.0760m/z	pos	Pyrantel	2191
2254	0.71_817.7289n	pos	PC(O-20:0/O-20:0)	40244
2255	0.72_949.4867n	neg	CDP-DG(18:2(9Z,11Z)/i-14:0)	HMDB0116054
2256	0.78_225.0759m/z	neg	threo-Syringoylglycerol	87513
2257	0.81_552.9761m/z	neg	Inosine triphosphate	3589
2258	0.83_659.1726m/z	neg	alpha-Viniferin	HMDB0030603
2259	1.18_367.1217m/z	pos	1-O-alpha-D-Glucopyranosyl-D-mannitol	HMDB0029911
2260	1.19_517.0973m/z	pos	4',5-Dihydroxy-3,3'-dimethoxy-6,7-methylenedioxyflavone 4'-glucuronide	92577
2261	1.21_427.0403m/z	neg	CDP-Ethanolamine	HMDB0001564
2262	10.00_660.1675m/z	neg	Pelargonidin 3,5-di-(6-acetylglucoside)	LMPK12010045
2263	10.11_434.2118m/z	pos	Formylmethionyl-leucyl-phenylalanine methyl ester	68923

2264	10.26_580.2869m/z	neg	POB-PS	LMGP20040017
2265	10.48_450.2616m/z	neg	LysoPE(0:0/16:1(9Z))	HMDB0011474
2266	10.60_876.4945m/z	pos	PE(22:5(4Z,7Z,10Z,13Z,16Z)/22:6(4Z,7Z,10Z,13Z,16Z,19Z))	61028
2267	10.80_699.0086m/z	neg	{3-hydroxy-5-oxo-2-[3-(sulfooxy)phenyl]-5H-chromen-7-yl}oxidanium	HMDB0125156
2268	10.89_508.3429m/z	pos	Gymnodimine	HMDB0041430
2269	10.99_208.1837n	pos	2-Cyclotetradecen-1-one	HMDB0039884
2270	11.22_409.2946m/z	pos	3alpha,9alpha,11beta-Trihydroxy-5beta-cholan-24-oic Acid	LMST04010104
2271	11.37_362.1902m/z	pos	Thaumatin b, recombinant	HMDB0032529
2272	12.35_149.1317m/z	pos	2,4-Undecadienal	HMDB0031359
2273	12.62_340.3242m/z	pos	(±)-CP 47,497-C8-homolog-d7	96643
2274	12.66_798.5414m/z	pos	PE(22:5(4Z,7Z,10Z,13Z,16Z)/P-18:1(11Z))	61033
2275	13.20_147.1170m/z	pos	1-(1-Methoxy-1-methylethyl)-4-methylbenzene	HMDB0029653
2276	13.20_763.5999m/z	pos	3-Demethylubiquinone-9	HMDB0060370
2277	13.37_567.4550m/z	pos	36:4(21Z,24Z,27Z,30Z)	LMFA01030829
2278	13.88_447.3471m/z	neg	Linalyl isobutyrate	86967
2279	14.15_591.4637m/z	neg	13-HODE	HMDB0061708
2280	2.12_418.3193m/z	pos	Philanthotoxin 343	69713
2281	5.53_393.2538m/z	pos	RCS-8	64777
2282	5.83_480.2829m/z	pos	Perindopril erbumine	66719

2283	6.62_464.2198m/z	pos	Diprenorphine	69224
2284	6.75_333.2532m/z	pos	Bremazocine	69221
2285	6.81_228.1597m/z	neg	Tecostanine	68068
2286	7.32_757.3075m/z	neg	Kasugamycin	71964
2287	7.57_222.1128m/z	neg	Phenmetrazine	HMDB0014968
2288	7.65_319.1305m/z	neg	Carboxyprimaquine	2082
2289	7.88_471.1307n	neg	2-amino-4-({2-[(2-carboxy-2-hydroxy-1-phenylethyl)sulfanyl]-1-[(carboxymethyl)-C-hydroxycarbonimidoyl]ethyl}-C-hydroxycarbonimidoyl)butanoic acid	HMDB0126549
2290	8.41_256.1912m/z	neg	Ethyl menthane carboxamide	92642
2291	8.62_651.2679m/z	pos	(3a,5b,7b,12a)-(1,3-dihydro-5-nitro-1,3-dioxo-2H-isoindol-2-yl)methyl ester-3,7,12-trihydroxy-Cholan	6688
2292	8.76_468.2760m/z	pos	Prostaglandin E2 p-acetamidophenyl ester	45604
2293	9.43_191.1058m/z	pos	Tris(1-aziridinyl)phosphine oxide	73220
2294	9.51_506.2635m/z	neg	Isodesmosine	5707
2295	9.60_213.1855m/z	pos	10-Undecenyl acetate	87350
2296	9.96_209.1540m/z	neg	5-Ethyl-3-methyl-2E,4E,6E-nonatriene	LMFA11000051
2297	0.63_134.9585m/z	neg	(E)-S-1-Propenyl thiosulfate	HMDB0041079

2298	0.65_390.9973m/z	neg	(E)-4-(6-methyl-1,2-dithiin-3-yl)but-1-en-3-yn-1-ol	LMFA12000367
2299	0.72_844.5806m/z	pos	PE(22:2(13Z,16Z)/20:3(5Z,8Z,11Z))	HMDB0009562
2300	0.72_992.5403m/z	pos	Asiaticoside B	86571
2301	0.73_975.2969n	neg	trans-tetradec-2-enoyl-CoA	LMFA07050396
2302	0.75_306.1550m/z	pos	Histidinyl-Lysine	85796
2303	0.76_205.0168m/z	neg	Thiacremonone	HMDB0035901
2304	0.81_387.1107m/z	neg	CRESOPYRINE	43914
2305	0.83_367.9953m/z	pos	NAc-DNP-Cys	69126
2306	0.87_292.2019m/z	pos	Allyxycarb	72707
2307	0.92_262.0668m/z	neg	Propetamphos	72453
2308	1.21_484.1197m/z	pos	Lucuminamide	HMDB0031697
2309	10.14_388.9912m/z	neg	6-Hydroxy-5-[(4-sulfophenyl)azo]-2-naphthalenesulfonic acid	HMDB0034022
2310	10.35_452.9964m/z	neg	Nitroglycerin	HMDB0014865
2311	10.63_333.2065m/z	neg	Dehydroepiandrosterone	HMDB0000077
2312	10.76_977.5641m/z	neg	PIM1(18:0/16:2(9Z,12Z))	LMGP15010017
2313	10.86_465.3537m/z	pos	DG(8:0/0:0/15:0)	HMDB0092918
2314	10.87_468.3494m/z	pos	N-(7Z,10Z,13Z-16Z-docosatetraenoyl) dopamine	LMFA08020274
2315	11.03_314.0319m/z	pos	(2S)-2-amino-3-[4-hydroxy-3-(sulfooxy)phenyl]-2-methylpropanoic acid	HMDB0142153
2316	11.07_596.2686m/z	pos	Z-Gly-Pro-Leu-Gly-Pro	65925

2317	11.11_643.1887m/z	pos	Malvidin 3-glucoside-4-vinylcatechol	LMPK12010436
2318	11.78_249.1491m/z	neg	(6R,7S)-6,7-Epoxy-1,3-tetradecadiyne	HMDB0031776
2319	11.81_295.2268m/z	neg	(±)9-HODE	35487
2320	12.02_483.3675m/z	neg	6-Ketomyristic acid	HMDB0030982
2321	12.19_522.3621m/z	pos	3-O-alpha-L-rhamnopyranosyl-3-hydroxydecanoyl-3-hydroxydecanoic acid	LMFA13030001
2322	12.70_515.3756m/z	pos	16-Acetylpriverogenin A	HMDB0034529
2323	12.92_503.3386m/z	pos	Medicagenic acid	HMDB0034551
2324	2.08_258.0919m/z	neg	Glutaminylmethionine	HMDB0028803
2325	4.18_317.0607m/z	neg	Pyriftalid	72291
2326	4.18_507.0956m/z	neg	farnesyl triphosphate	LMPR0103010023
2327	4.18_635.2485m/z	pos	Dehydroisocoproporphyrinogen	HMDB0002242
2328	4.24_650.0315m/z	neg	Adenophostin A	69662
2329	4.64_333.1445m/z	pos	(2E)-N-[2-(3,4-dihydroxyphenyl)-2-hydroxyethyl]-3-(4-hydroxyphenyl)prop-2-enimidic acid	HMDB0136318
2330	5.11_534.3167m/z	pos	POV-PE	LMGP20020030
2331	7.61_469.1120m/z	pos	Luteolin 5-galactoside	LMPK12110638
2332	7.65_469.2232m/z	neg	Licoricidin	LMPK12080045
2333	8.14_647.2394m/z	pos	Bis(5-hydroxynoracronycine)	88197
2334	8.54_399.2196m/z	neg	Lucidone B	HMDB0035834

2335	8.99_397.2042m/z	neg	Pregnanalone sulfate (Allopregnanolone sulfate)	3560
2336	9.52_147.0668m/z	pos	Butyl isopropyl disulfide	HMDB0033050
2337	9.60_682.4146m/z	pos	Elatoside G	HMDB0041347
2338	9.70_351.0752m/z	neg	4-phenyl-5-methyl-1,2,3-Thiadiazole	45304
2339	0.70_795.7351n	neg	TG(15:0/17:1(9Z)/15:0)	46630
2340	0.72_964.5470m/z	pos	CDP-DG(16:0/18:0)	58604
2341	0.73_780.4187m/z	neg	Leucomycin A6	69773
2342	0.75_320.0425m/z	pos	Nitrazepam	HMDB0015534
2343	0.82_660.1845n	pos	Isophylloflavanine	LMPK12020080
2344	0.92_337.1324m/z	pos	2,9-Dimethyl-2,9-diazatricyclo[10.2.2.25,8]octadeca-5,7,12,14,15,17-hexaene-3,10-diol, 9CI	HMDB0038832
2345	1.21_285.1158m/z	pos	1-dimethylarsinoyl-decane	LMFA11000719
2346	1.21_689.2123m/z	pos	Cellotetraose	63206
2347	10.39_502.2926m/z	neg	LysoPE(0:0/20:3(5Z,8Z,11Z))	HMDB0011485
2348	10.41_643.2729m/z	pos	Lanceotoxin A	67237
2349	10.42_509.3370m/z	pos	Ambolic acid	HMDB0035713
2350	10.54_552.0186n	neg	Myricatin	HMDB0029513
2351	10.98_178.1599m/z	pos	3-Amino-1-hydroxy-5,7-dimethyl-adamantane	HMDB0060738
2352	11.01_195.1326m/z	pos	ARCAINE	43946
2353	11.23_727.5625m/z	pos	Cholesteryl 11-hydroperoxy-eicosatetraenoate	41724

2354	11.46_515.3727m/z	pos	Oleanderolide 3-acetate	HMDB0034647
2355	11.93_282.1667m/z	pos	L-Hexanoylcarnitine	HMDB0000756
2356	11.94_607.3727m/z	neg	CPX	44518
2357	12.70_707.5025m/z	pos	PA(O-16:0/22:6(4Z,7Z,10Z,13Z,16Z,19Z))	82236
2358	12.92_459.3112m/z	pos	Polyporusterone B	HMDB0038496
2359	13.13_489.3572m/z	neg	Schleicherastatin 5	HMDB0035804
2360	13.76_579.4114m/z	pos	Vecuronium	3000
2361	13.79_763.5388n	pos	PS(16:0/18:0)	LMGP03010906
2362	13.97_540.4388n	pos	TG(8:0/8:0/a-13:0)[rac]	HMDB0071246
2363	14.78_593.4793m/z	neg	Cohibin A	HMDB0031168
2364	4.25_443.0555m/z	pos	{4-[3-(5-hydroxy-2,2-dimethyl-2H-chromen-6-yl)propanoyl]phenyl}oxidanesulfonic acid	HMDB0125896
2365	4.64_386.2396m/z	pos	talinolol	96207
2366	5.04_310.0520n	pos	{3-[2-(3,5-dihydroxyphenyl)ethyl]phenyl}oxidanesulfonic acid	HMDB0129951
2367	5.20_452.0765m/z	neg	(beta-1-O-[N-(2-methyl-3-chlorophenyl-4-hydroxy)anthraniloyl]-D-glucupyranuronic acid)	HMDB0060035
2368	5.81_458.2077m/z	pos	Hericerin	HMDB0038587
2369	6.06_287.1396m/z	neg	(±)-Rollipyrrole	HMDB0037554
2370	6.09_409.2758m/z	pos	(23S)-1α-hydroxy-25,27-didehydrovitamin D3 26,23-lactone	42527

2371	6.11_385.1666m/z	neg	3-[4-hydroxy-2-methoxy-3-(3-methylbut-2-en-1-yl)phenyl]-1-(3-hydroxyphenyl)propan-1-one	HMDB0124820
2372	7.02_488.1911m/z	neg	Terbinafine metabolite glucuronide	2748
2373	7.77_172.1699m/z	pos	N,2,3-Trimethyl-2-(1-methylethyl)butanamide	91344
2374	7.86_255.1247m/z	neg	Jasmonic acid	HMDB0032797
2375	8.02_663.2677m/z	pos	3'-Deoxystreptomycin 6-phosphate	2500
2376	8.12_618.3912m/z	pos	LysoPC(22:0)	61707
2377	8.16_459.1394m/z	neg	BENZANTHRONE	44054
2378	8.78_599.2489m/z	neg	Toonacilin	67202
2379	9.56_562.2262m/z	pos	Physalin E	89851
2380	0.73_868.3550m/z	neg	Virginiamycin	HMDB0030520
2381	0.73_977.2913n	neg	Malvidin 3-O-(6-O-(4-O-feruloyl-alpha-rhamnopyranosyl)-beta-glucopyranoside)-5-beta-glucopyranoside	LMPK12010409
2382	0.82_364.1160m/z	pos	MONOCROTALINE	44128
2383	0.87_645.0887m/z	neg	Wy 14643	43447
2384	0.92_578.0792m/z	neg	CDP-3,6-dideoxy-D-glucose	63230
2385	1.21_266.0560n	pos	Methoxybrassinin	HMDB0033351
2386	1.21_444.0215m/z	neg	Bufferin	69567
2387	10.11_431.3156m/z	neg	1-Phenyl-1,3-eicosanedione	HMDB0032925

2388	10.27_585.2253m/z	pos	2-amino-4-({1-[(carboxymethyl)-C-hydroxycarbonimidoyl]-2-{[3-hydroxy-4-(7-methoxy-2-oxo-2H-chromen-8-yl)-2-methylbutan-2-yl]sulfanyl}ethyl}-C-hydroxycarbonimidoyl)butanoic acid	HMDB0132694
2389	10.66_645.2133m/z	pos	Phaeophorbide b	HMDB0031149
2390	10.67_376.1430m/z	pos	Bitertanol	68944
2391	10.93_446.2569m/z	pos	CAY10594	45492
2392	11.11_398.2366m/z	pos	(±)14(15)-EET-SI	45177
2393	11.26_681.4390m/z	pos	13-sophorosyloxydocosanoic acid	LMFA13020004
2394	11.75_527.3839m/z	pos	LysoPE(20:0/0:0)	HMDB0011511
2395	11.80_321.2186m/z	neg	Etidocaine	66669
2396	12.03_293.1781m/z	neg	Granisetron	HMDB0015026
2397	15.61_431.3521m/z	pos	Schleicherastatin 6	90794
2398	2.00_597.1505m/z	pos	Viniferal	HMDB0029790
2399	2.12_399.1146m/z	pos	6-({2-[(acetyloxy)methyl]-4,5,6-trihydroxyoxan-3-yl}oxy)-3,4,5-trihydroxyoxane-2-carboxylic acid	HMDB0125228
2400	2.14_218.0617m/z	neg	N-Benzylphthalimide	69922
2401	2.65_249.0199m/z	neg	Coumestrol	48332
2402	3.01_446.0649m/z	neg	5-Hydroxylansoprazole sulfone	923

2403	4.93_232.0604m/z	neg	N-Phenylacetylaspartic acid	HMDB0029355
2404	5.22_285.0407m/z	pos	Bracteatin	LMPK12130044
2405	6.08_335.1247m/z	neg	Dinocton 6	72683
2406	6.27_236.0954m/z	neg	Trihomomethionine	64500
2407	6.80_424.1963m/z	neg	O-Desmethyltramadol glucuronide	HMDB0060856
2408	7.51_313.1186m/z	neg	Lysergic acid	66676
2409	7.65_465.1897m/z	neg	Hydrocortisone sodium succinate	66842
2410	7.97_260.0821m/z	pos	Indirubin-3'-monoxime	45531
2411	7.98_450.1706m/z	pos	6-Hydroxyfluvastatin	HMDB0014037
2412	8.27_517.2466m/z	pos	2-O-Methyl-L-fucose	89534
2413	8.52_491.1922m/z	pos	3,4,5-trihydroxy-6-({2-[6-(2-methylbut-3-en-2-yl)-7-oxo-2H,3H,7H-furo[3,2-g]chromen-2-yl]propan-2-yl}oxy)oxane-2-carboxylic acid	HMDB0130297
2414	0.62_452.3482n	pos	5b-Cholestane-3a,7a,12a,23S,25-pentol	LMST01010241
2415	0.63_679.3140m/z	neg	Asteriidoside L	84931
2416	0.63_687.3045m/z	neg	Physagulin E	94248
2417	0.73_708.4587m/z	neg	PE(14:0/20:5(5Z,8Z,11Z,14Z,17Z))	HMDB0008840
2418	0.84_361.0876m/z	neg	Bumetanide M1	1395
2419	0.97_197.0539m/z	neg	4,4'-Thiodianiline	73015
2420	1.21_433.1519m/z	pos	BRUCINE	43921
2421	1.22_205.9895m/z	pos	Thioguanine	HMDB0014496

2422	10.06_411.2014m/z	neg	11-dehydro-TXB3	LMFA03030009
2423	10.20_213.0658m/z	neg	Phenobarbital	HMDB0015305
2424	10.75_556.2144m/z	pos	Nalbuphine-3-glucuronide	1402
2425	10.78_288.1369m/z	pos	Benzosimuline	88044
2426	10.98_285.2435m/z	pos	Avocadyne	HMDB0035473
2427	11.13_289.1447m/z	pos	Sodium lauryl sulfate	58284
2428	11.33_203.1440m/z	pos	Tetradecan-7,9-diynoic acid	LMFA01031094
2429	11.72_455.3595m/z	pos	Solacapine	68604
2430	11.76_640.2922m/z	neg	S-(PGA2)-glutathione	HMDB0013062
2431	12.85_383.1773m/z	pos	JWH 200 4-hydroxyindole metabolite	96658
2432	13.20_475.3061m/z	pos	Pubescenol	HMDB0030085
2433	13.45_463.3420m/z	neg	20a,22b-Dihydroxycholesterol	HMDB0006763
2434	13.98_780.1590m/z	pos	3'-Deoxystreptomycin 3'α,6-bisphosphate	2518
2435	14.27_860.6135m/z	pos	PC(22:0/18:4(6Z,9Z,12Z,15Z))	HMDB0008536
2436	14.53_601.5220m/z	pos	DG(14:1(9Z)/22:2(13Z,16Z)/0:0)	HMDB0007059
2437	15.83_223.0651m/z	pos	1-(1-Propenylthio)propyl propyl disulfide	88933
2438	2.01_270.0342m/z	neg	Brassica oleracea Alkaloid	89404
2439	2.14_351.0765m/z	neg	N4-Acetylsulfadoxine	2543

2440	4.19_510.1329m/z	pos	2-amino-4-({1-[(carboxymethyl)-C-hydroxycarbonimidoyl]-2-[(2,3-dihydroxy-2-methyl-1-phenylpropyl)sulfanyl]ethyl}-C-hydroxycarbonimidoyl)butanoic acid	HMDB0133654
2441	4.72_214.0335m/z	neg	Brassicanal B	93257
2442	5.27_258.0558m/z	neg	PPM-18	64815
2443	5.37_349.2261m/z	pos	JWH 018 2-hydroxyindole metabolite-d9	96646
2444	6.37_418.1454m/z	pos	Meperidinic acid glucuronide	1135
2445	6.73_230.9858m/z	neg	4-Hydroxyriluzole	2323
2446	9.02_239.0577m/z	neg	trans-Ferulic acid	HMDB0000954
2447	9.05_248.0807m/z	neg	Adenosine	HMDB0000050
2448	0.62_415.0420m/z	pos	5,8-dihydroxy-2-(1-hydroxy-3-methoxy-4-oxocyclohexa-2,5-dien-1-yl)-3,7-dimethoxy-4H-chromen-4-one	HMDB0129273
2449	0.63_663.3342m/z	neg	Minosaminomycin	71965
2450	0.63_797.2733m/z	neg	Difloxacin	68932
2451	0.69_417.0459m/z	neg	NORSTICTIC ACID	43603
2452	0.83_976.1360m/z	pos	1,4-Dihydroxy-2-naphthoyl-CoA	63987
2453	1.23_129.0198m/z	pos	Di-2-propenyl disulfide, 9CI	HMDB0033966
2454	10.12_258.0813m/z	pos	2-Amino-6-(benzylthio)purine	70755

2455	10.12_448.0117n	neg	3,6-dihydroxy-2-[3-methoxy-4-(sulfooxy)phenyl]-5-sulfino-3,4-dihydro-2H-1-benzopyran-7-olate	HMDB0127804
2456	10.13_261.1832m/z	pos	Guaidiol	HMDB0033228
2457	10.67_345.1678m/z	neg	Spenolimycin	71966
2458	10.79_300.1367m/z	pos	Acrifoline	67964
2459	10.91_481.3123m/z	neg	Latanoprost-d4	96388
2460	10.98_826.5626m/z	neg	PC(16:0/20:4(5Z,8Z,11Z,14Z))	39120
2461	12.62_773.5827m/z	neg	SM(d18:1/18:1(11Z))	HMDB0012100
2462	14.76_663.4597m/z	pos	PG(a-13:0/i-16:0)	HMDB0116646
2463	2.00_552.3932m/z	pos	Dendrogenin A	LMST05050025
2464	2.13_650.2376m/z	pos	Tri-N-acetylchitotriose	HMDB0006698
2465	3.02_299.9733m/z	neg	5-Trifluoromethyl-2,4-disulfamoylaniline	3181
2466	4.19_841.3274m/z	pos	Dermorphin	73515
2467	4.25_104.0501m/z	pos	Aminofluoropropionic acid	690
2468	4.38_231.1729m/z	pos	2-Decylfuran	88266
2469	6.62_230.9858m/z	neg	5-Hydroxyriluzole	2324
2470	6.76_262.0649m/z	pos	ZLJ-6	45512
2471	7.75_565.2999m/z	neg	24,25-Diacetylvulgaroside	HMDB0041366
2472	9.50_284.1850n	pos	Melanostatin	58299
2473	9.52_213.0702m/z	pos	Hexanethioic acid S-propyl ester	HMDB0039466
2474	9.60_247.0858m/z	pos	Perlolyrine	HMDB0030327
2475	9.93_944.1857m/z	pos	Salviadelphin	LMPK12010302

2476	0.73_769.4229n	neg	Niddamycin	69327
2477	0.77_430.1585m/z	neg	Minoxidil-O-glucuronide	1320
2478	0.80_395.0941m/z	neg	4-Methyldibenzothiophene	94701
2479	0.80_441.0682m/z	pos	Glucoiberin	HMDB0038406
2480	0.81_198.0706m/z	neg	1-aminopyrene	96142
2481	0.82_630.1227n	pos	2'-C-Methylmyricetin 3-rhamnoside-5'-gallate	50846
2482	0.93_237.0494m/z	neg	4,5-diphenyl-1,2,3-Thiadiazole	45303
2483	1.23_447.0489m/z	neg	Cysteinyl-Cysteine	HMDB0028772
2484	1.26_303.0316m/z	neg	IPSP	72751
2485	1.47_213.0121m/z	neg	Cyclobrassinone	HMDB0034209
2486	10.43_263.1644m/z	neg	Nookatone	HMDB0013687
2487	10.52_606.3029m/z	neg	PKHdiA-PE	LMGP20020016
2488	10.86_876.4960m/z	pos	PE(22:5(7Z,10Z,13Z,16Z,19Z)/22:6(4Z,7Z,10Z,13Z,16Z,19Z))	HMDB0009672
2489	10.99_144.1013m/z	pos	1-Aminocyclohexanecarboxylic acid	6589
2490	11.05_523.3030m/z	pos	Ganoderic acid beta	HMDB0033234
2491	11.53_405.2633m/z	neg	1b-Hydroxycholic acid	HMDB0000307
2492	11.83_622.4083m/z	pos	Enniatin B	71071
2493	11.94_627.4755m/z	pos	PA(O-16:0/17:2(9Z,12Z))	LMGP10020010
2494	13.00_501.3961m/z	pos	OLEANANOIC ACID ACETATE	44453
2495	13.14_682.3719m/z	pos	OKOHA-PC	LMGP20010031
2496	14.43_883.5035m/z	pos	Pitheduloside B	HMDB0034865

2497	14.47_515.3783m/z	pos	Hexafluronium	85446
2498	2.14_394.1403m/z	neg	Anisessine	68452
2499	4.67_347.1267m/z	pos	3,6-Dimethoxy-6",6"-dimethylpyrano[2,3:7,8]flavone	LMPK12111585
2500	4.90_345.1058m/z	neg	meta-O-Dealkylated flecainide lactam	HMDB0060832
2501	5.33_350.1498m/z	neg	Arginyl-Methionine	85630
2502	5.80_327.2283m/z	pos	Boc-DVal(NMe)-Val-OMe	65479
2503	5.86_285.2792m/z	pos	ethyl hexadecanoate	LMFA07010471
2504	6.51_198.1318m/z	pos	Cycloate	72550
2505	6.80_282.1635m/z	pos	Xanomeline	69206
2506	6.91_479.2249m/z	neg	Obtustyrene	HMDB0030672
2507	6.97_268.9845m/z	neg	4-thiodimethylarsenobutanoic acid	LMFA00000041
2508	7.12_397.1668m/z	neg	Ro 31-7549	45539
2509	7.57_331.2607m/z	pos	Sclareol	HMDB0036827
2510	8.08_327.0977m/z	neg	Nifedipine	HMDB0015247
2511	8.28_660.3995m/z	pos	PC(10:0/14:0)[U]	39128
2512	8.95_447.1331m/z	neg	Sinapic acid	HMDB0032616
2513	9.30_252.1742m/z	pos	3-methyl-4-(methylamino)-1,2-diphenylbutan-2-ol	HMDB0144299
2514	9.51_484.2817m/z	neg	LysoPE(0:0/20:3(8Z,11Z,14Z))	HMDB0011486
2515	9.84_433.3243m/z	pos	24-Noroleana-3,12-diene	265081
2516	0.01_103.9528m/z	pos		
2517	0.01_104.9269n	neg		

2518	0.01_108.9367n	pos
2519	0.01_122.8932m/z	neg
2520	0.01_124.8905m/z	neg
2521	0.01_143.8647m/z	neg
2522	0.01_150.9821m/z	neg
2523	0.01_156.9388m/z	neg
2524	0.01_167.8825m/z	neg
2525	0.01_169.8942n	neg
2526	0.01_171.9125n	neg
2527	0.01_178.9776m/z	neg
2528	0.01_180.9127m/z	neg
2529	0.01_182.9648m/z	pos
2530	0.01_189.8594m/z	neg
2531	0.01_197.9010m/z	neg
2532	0.01_206.9725m/z	neg
2533	0.01_216.8798m/z	neg
2534	0.01_241.8962m/z	neg
2535	0.01_243.8988m/z	neg
2536	0.01_245.8919m/z	neg
2537	0.01_250.9068m/z	neg
2538	0.01_252.9960m/z	neg
2539	0.01_261.8726m/z	neg
2540	0.01_307.8553m/z	neg
2541	0.01_316.8651m/z	neg
2542	0.01_320.8838m/z	neg

2543	0.01_326.8451m/z	neg
2544	0.01_336.8537m/z	neg
2545	0.01_341.8378m/z	neg
2546	0.02_120.9670m/z	pos
2547	0.02_122.9644m/z	pos
2548	0.02_137.9644m/z	pos
2549	0.02_140.9441m/z	neg
2550	0.02_142.8746n	neg
2551	0.02_142.8747m/z	neg
2552	0.02_145.8622m/z	neg
2553	0.02_152.9780m/z	neg
2554	0.02_179.8406n	neg
2555	0.02_228.9595m/z	neg
2556	0.02_240.9049m/z	pos
2557	0.02_297.8285m/z	neg
2558	0.03_100.9384m/z	pos
2559	0.03_116.9279m/z	neg
2560	0.03_116.9765m/z	pos
2561	0.03_126.9674m/z	pos
2562	0.03_152.9630m/z	pos
2563	0.03_163.9371m/z	neg
2564	0.03_171.8741m/z	neg
2565	0.03_173.8690m/z	neg
2566	0.03_177.8435n	neg
2567	0.03_206.9181m/z	pos

2568	0.03_213.8938m/z	pos
2569	0.03_213.9228n	neg
2570	0.03_217.8621m/z	pos
2571	0.03_224.8944m/z	pos
2572	0.03_249.9387m/z	pos
2573	0.03_256.9542m/z	neg
2574	0.03_263.8685m/z	neg
2575	0.03_270.8948m/z	pos
2576	0.03_281.8952m/z	pos
2577	0.03_289.8897n	pos
2578	0.03_301.8613m/z	neg
2579	0.03_610.1878m/z	pos
2580	0.03_684.2070m/z	pos
2581	0.04_102.0133m/z	pos
2582	0.04_104.0088m/z	pos
2583	0.04_113.9637m/z	pos
2584	0.04_130.0087m/z	pos
2585	0.04_138.9650m/z	pos
2586	0.04_171.9713n	pos
2587	0.04_181.9863m/z	pos
2588	0.04_201.9240m/z	neg
2589	0.04_213.1135m/z	pos
2590	0.04_230.8912m/z	pos
2591	0.04_234.8568m/z	neg
2592	0.04_241.8874m/z	pos

2593	0.04_244.9133n	pos
2594	0.04_247.8424m/z	neg
2595	0.04_258.8876m/z	pos
2596	0.04_273.8968n	pos
2597	0.04_287.8915m/z	pos
2598	0.04_293.8576m/z	pos
2599	0.04_295.8718n	pos
2600	0.04_297.8863n	pos
2601	0.04_519.1421m/z	pos
2602	0.05_124.9667m/z	pos
2603	0.05_181.8380n	neg
2604	0.06_203.8550m/z	neg
2605	0.06_232.8621m/z	neg
2606	0.06_249.8483m/z	neg
2607	0.06_322.8769m/z	pos
2608	0.08_151.8883m/z	neg
2609	0.08_163.8458m/z	neg
2610	0.08_281.8572m/z	neg
2611	0.08_324.8487m/z	neg
2612	0.09_157.8623m/z	neg
2613	0.09_159.8594m/z	neg
2614	0.09_179.9908m/z	pos
2615	0.09_207.8361m/z	neg
2616	0.09_209.8332m/z	neg
2617	0.09_262.8682m/z	pos

2618	0.10_128.9516m/z	pos
2619	0.10_213.8848m/z	neg
2620	0.11_129.9594n	pos
2621	0.12_161.9255m/z	neg
2622	0.12_259.8764m/z	neg
2623	0.12_307.8688m/z	pos
2624	0.13_125.8721m/z	neg
2625	0.13_262.0511m/z	pos
2626	0.14_127.8693m/z	neg
2627	0.14_177.9137m/z	pos
2628	0.14_239.9022m/z	neg
2629	0.14_275.8904m/z	pos
2630	0.14_334.0361m/z	pos
2631	0.15_117.9356m/z	pos
2632	0.15_172.8636m/z	pos
2633	0.16_158.9617m/z	pos
2634	0.16_185.8873n	neg
2635	0.16_186.9909m/z	pos
2636	0.16_206.8473n	neg
2637	0.17_102.9342m/z	pos
2638	0.17_224.9170m/z	neg
2639	0.18_105.9351m/z	pos
2640	0.18_144.9238n	pos
2641	0.18_207.9298m/z	neg
2642	0.18_208.9444m/z	pos

2643	0.18_234.8639m/z	pos
2644	0.18_303.8866m/z	pos
2645	0.18_320.8772m/z	pos
2646	0.20_278.8579m/z	neg
2647	0.22_171.9843m/z	pos
2648	0.22_186.9913m/z	pos
2649	0.22_195.9151m/z	pos
2650	0.22_786.2605m/z	pos
2651	0.23_145.9302m/z	neg
2652	0.28_179.9910m/z	pos
2653	0.35_178.9211m/z	pos
2654	0.38_179.9907m/z	pos
2655	0.53_179.9932m/z	pos
2656	0.57_126.9680m/z	pos
2657	0.57_150.9194n	neg
2658	0.57_217.8621m/z	pos
2659	0.58_105.9351m/z	pos
2660	0.58_113.9638m/z	pos
2661	0.58_170.8830m/z	neg
2662	0.58_194.9049m/z	neg
2663	0.58_194.9447m/z	pos
2664	0.58_225.9250m/z	neg
2665	0.58_258.8880m/z	pos
2666	0.58_294.8694m/z	pos
2667	0.58_308.8698m/z	neg

	0.58_324.84	
2668	86m/z	neg
	0.59_101.95	
2669	01m/z	pos
	0.59_105.03	
2670	33m/z	pos
	0.59_137.96	
2671	45m/z	pos
	0.59_142.97	
2672	80m/z	pos
	0.59_146.93	
2673	75n	pos
	0.59_156.89	
2674	06m/z	pos
	0.59_180.91	
2675	22m/z	neg
	0.59_183.91	
2676	43m/z	neg
	0.59_197.90	
2677	12m/z	neg
	0.59_205.91	
2678	01m/z	pos
	0.59_208.93	
2679	57m/z	neg
	0.59_210.93	
2680	22m/z	pos
	0.59_222.90	
2681	49m/z	pos
	0.59_228.89	
2682	77m/z	pos
	0.59_230.89	
2683	31m/z	pos
	0.59_239.88	
2684	04m/z	pos
	0.59_241.89	
2685	70n	pos
	0.59_243.23	
2686	36m/z	pos
	0.59_247.89	
2687	37m/z	neg
	0.59_251.90	
2688	25m/z	pos
	0.59_255.82	
2689	22m/z	neg
	0.59_265.87	
2690	90m/z	neg
	0.59_275.90	
2691	48m/z	neg
	0.59_281.85	
2692	50m/z	neg

2693	0.59_292.8880n	neg
2694	0.59_299.8915m/z	pos
2695	0.59_327.8583m/z	neg
2696	0.59_338.8906m/z	neg
2697	0.59_342.8712m/z	pos
2698	0.59_353.8536m/z	pos
2699	0.59_367.8585m/z	pos
2700	0.59_379.8632m/z	neg
2701	0.59_492.8335m/z	neg
2702	0.59_509.8199m/z	neg
2703	0.59_786.2585m/z	pos
2704	0.61_109.6770m/z	pos
2705	0.61_131.0196m/z	pos
2706	0.61_152.9175m/z	neg
2707	0.61_157.8977m/z	pos
2708	0.61_164.0858n	pos
2709	0.61_165.9379m/z	neg
2710	0.61_171.9462m/z	pos
2711	0.61_179.9031m/z	pos
2712	0.61_182.4060m/z	pos
2713	0.61_188.9182m/z	pos
2714	0.61_192.9571m/z	neg
2715	0.61_201.8881m/z	pos
2716	0.61_215.8689m/z	neg
2717	0.61_220.8768m/z	pos

2718	0.61_231.1068m/z	pos
2719	0.61_235.9029m/z	pos
2720	0.61_238.1119m/z	pos
2721	0.61_246.8878n	pos
2722	0.61_249.9202m/z	pos
2723	0.61_253.9640n	pos
2724	0.61_260.9762m/z	pos
2725	0.61_261.0456m/z	pos
2726	0.61_271.9174m/z	pos
2727	0.61_279.5493m/z	pos
2728	0.61_292.8811n	pos
2729	0.61_297.1409m/z	pos
2730	0.61_300.8826n	pos
2731	0.61_307.8890m/z	pos
2732	0.61_310.8696m/z	neg
2733	0.61_327.8674n	pos
2734	0.61_336.8970n	pos
2735	0.61_348.9175m/z	neg
2736	0.61_376.8593m/z	pos
2737	0.61_397.3628m/z	pos
2738	0.61_416.8657n	pos
2739	0.61_421.8403m/z	neg
2740	0.61_431.0194m/z	pos
2741	0.61_440.8649n	pos
2742	0.61_454.1050n	pos

2743	0.61_467.8339m/z	neg
2744	0.61_468.8504m/z	neg
2745	0.61_508.3617m/z	pos
2746	0.61_543.3294n	pos
2747	0.61_559.3426m/z	pos
2748	0.61_618.3139m/z	pos
2749	0.61_625.3159m/z	pos
2750	0.62_114.9876m/z	pos
2751	0.62_126.8793m/z	neg
2752	0.62_142.9604n	pos
2753	0.62_145.0160m/z	pos
2754	0.62_154.9672m/z	pos
2755	0.62_166.9246m/z	neg
2756	0.62_191.9457m/z	neg
2757	0.62_199.9665n	pos
2758	0.62_215.9448n	pos
2759	0.62_216.9786n	pos
2760	0.62_217.9329n	neg
2761	0.62_231.9093m/z	pos
2762	0.62_231.9227n	pos
2763	0.62_236.9302m/z	neg
2764	0.62_240.9934n	pos
2765	0.62_245.9381n	pos
2766	0.62_261.9380n	pos
2767	0.62_280.9057m/z	neg

2768	0.62_289.9325n	pos
2769	0.62_294.8862m/z	neg
2770	0.62_313.8873n	pos
2771	0.62_321.9035m/z	neg
2772	0.62_357.4035m/z	pos
2773	0.62_359.9425n	pos
2774	0.62_360.9393m/z	neg
2775	0.62_369.8890m/z	pos
2776	0.62_372.8962m/z	pos
2777	0.62_378.8722n	pos
2778	0.62_391.4039n	pos
2779	0.62_394.3815m/z	pos
2780	0.62_396.8517n	pos
2781	0.62_408.8654m/z	neg
2782	0.62_410.8634m/z	neg
2783	0.62_422.3816m/z	pos
2784	0.62_424.8454m/z	neg
2785	0.62_426.9018m/z	pos
2786	0.62_429.8515m/z	pos
2787	0.62_435.8691n	pos
2788	0.62_440.8301m/z	neg
2789	0.62_444.3624n	pos
2790	0.62_450.3626n	pos
2791	0.62_456.3781n	pos
2792	0.62_459.3610m/z	pos

2793	0.62_459.8835n	pos
2794	0.62_471.8479n	pos
2795	0.62_477.8518n	neg
2796	0.62_478.8711m/z	neg
2797	0.62_482.8531n	pos
2798	0.62_487.3582m/z	pos
2799	0.62_494.8475m/z	neg
2800	0.62_501.3515n	pos
2801	0.62_502.3528m/z	pos
2802	0.62_509.3372n	pos
2803	0.62_512.8244m/z	neg
2804	0.62_516.3502m/z	pos
2805	0.62_528.8633m/z	pos
2806	0.62_538.8272m/z	neg
2807	0.62_544.8448m/z	pos
2808	0.62_551.8280n	pos
2809	0.62_551.8328m/z	pos
2810	0.62_552.3356m/z	pos
2811	0.62_553.3356m/z	pos
2812	0.62_555.8313m/z	pos
2813	0.62_557.8495n	pos
2814	0.62_560.3354m/z	pos
2815	0.62_566.3318n	pos
2816	0.62_573.3380m/z	pos
2817	0.62_574.3173n	pos

2818	0.62_581.3306m/z	pos
2819	0.62_612.8156m/z	pos
2820	0.62_627.8019n	pos
2821	0.62_631.3114n	pos
2822	0.62_637.8115m/z	pos
2823	0.62_639.2959n	pos
2824	0.62_646.3084m/z	pos
2825	0.62_665.3009n	pos
2826	0.62_668.7965m/z	pos
2827	0.62_668.8269n	neg
2828	0.62_695.7844n	pos
2829	0.62_696.2872n	pos
2830	0.62_701.7626m/z	neg
2831	0.62_704.2760n	pos
2832	0.62_708.7749n	pos
2833	0.62_711.2899m/z	pos
2834	0.62_742.7785m/z	pos
2835	0.62_763.7724m/z	pos
2836	0.62_770.7712m/z	pos
2837	0.62_772.7751n	pos
2838	0.62_778.7621m/z	pos
2839	0.62_782.7537n	pos
2840	0.62_804.7619m/z	pos
2841	0.62_809.7191n	pos
2842	0.62_811.7501n	pos

2843	0.62_840.7433m/z	pos
2844	0.62_854.7430m/z	pos
2845	0.62_855.7367n	pos
2846	0.62_866.7462n	pos
2847	0.62_87.0198n	pos
2848	0.62_875.2700n	neg
2849	0.62_974.2331n	neg
2850	0.63_114.9878m/z	neg
2851	0.63_118.9657m/z	neg
2852	0.63_130.9656m/z	neg
2853	0.63_131.9753m/z	neg
2854	0.63_136.9238m/z	neg
2855	0.63_139.9555n	neg
2856	0.63_146.9604m/z	neg
2857	0.63_175.9658m/z	neg
2858	0.63_178.9484m/z	neg
2859	0.63_195.9386m/z	neg
2860	0.63_244.9585m/z	neg
2861	0.63_260.9262n	neg
2862	0.63_261.9520m/z	neg
2863	0.63_272.9584m/z	neg
2864	0.63_277.9268n	neg
2865	0.63_288.0446m/z	neg
2866	0.63_288.9364m/z	neg
2867	0.63_289.9424m/z	neg

	0.63_292.93	
2868	04m/z	neg
	0.63_303.97	
2869	19m/z	neg
	0.63_304.91	
2870	38m/z	neg
	0.63_325.89	
2871	63m/z	neg
	0.63_377.86	
2872	67m/z	neg
	0.63_402.91	
2873	55m/z	neg
	0.63_417.59	
2874	67m/z	neg
	0.63_418.89	
2875	39m/z	neg
	0.63_433.52	
2876	42m/z	neg
	0.63_438.86	
2877	28m/z	neg
	0.63_451.86	
2878	07m/z	neg
	0.63_455.85	
2879	24m/z	neg
	0.63_461.87	
2880	26n	neg
	0.63_505.87	
2881	86m/z	neg
	0.63_532.87	
2882	38m/z	neg
	0.63_548.85	
2883	22m/z	neg
	0.63_564.82	
2884	98m/z	neg
	0.63_581.81	
2885	90m/z	neg
	0.63_585.81	
2886	20m/z	neg
	0.63_589.81	
2887	12n	neg
	0.63_609.85	
2888	47n	neg
	0.63_614.33	
2889	37m/z	neg
	0.63_622.32	
2890	40m/z	neg
	0.63_624.85	
2891	55n	neg
	0.63_630.31	
2892	49m/z	neg

2893	0.63_649.8358n	neg
2894	0.63_650.8155m/z	neg
2895	0.63_671.3241m/z	neg
2896	0.63_675.3137n	neg
2897	0.63_682.8038m/z	neg
2898	0.63_686.7989m/z	neg
2899	0.63_689.7882m/z	neg
2900	0.63_695.2944m/z	neg
2901	0.63_711.7782m/z	neg
2902	0.63_720.8155m/z	neg
2903	0.63_727.8122m/z	neg
2904	0.63_728.3135m/z	neg
2905	0.63_731.2961m/z	neg
2906	0.63_736.3037m/z	neg
2907	0.63_739.7867m/z	neg
2908	0.63_740.2845m/z	neg
2909	0.63_744.2937m/z	neg
2910	0.63_744.7983n	neg
2911	0.63_751.7903m/z	neg
2912	0.63_752.2838m/z	neg
2913	0.63_764.7996m/z	neg
2914	0.63_777.8113m/z	neg
2915	0.63_779.2917n	neg
2916	0.63_782.7706m/z	neg
2917	0.63_785.3024m/z	neg

2918	0.63_785.7930m/z	neg
2919	0.63_792.7910m/z	neg
2920	0.63_793.2930m/z	neg
2921	0.63_801.2831m/z	neg
2922	0.63_804.7769n	neg
2923	0.63_806.2610m/z	neg
2924	0.63_808.7691m/z	neg
2925	0.63_809.2735m/z	neg
2926	0.63_814.7321m/z	neg
2927	0.63_820.7993n	neg
2928	0.63_824.7468m/z	neg
2929	0.63_836.2810n	neg
2930	0.63_844.2711n	neg
2931	0.63_850.2815m/z	neg
2932	0.63_852.7602m/z	neg
2933	0.63_858.2717m/z	neg
2934	0.63_861.7754n	neg
2935	0.63_866.2619m/z	neg
2936	0.63_869.7478m/z	neg
2937	0.63_883.2622n	neg
2938	0.63_897.7451m/z	neg
2939	0.63_901.2712m/z	neg
2940	0.63_903.2545m/z	neg
2941	0.63_909.2510n	neg
2942	0.63_914.7481m/z	neg

2943	0.63_915.2598m/z	neg
2944	0.63_923.2508m/z	neg
2945	0.63_931.2408m/z	neg
2946	0.63_931.7527n	neg
2947	0.63_938.7265m/z	neg
2948	0.63_940.2498n	neg
2949	0.63_958.2447m/z	neg
2950	0.63_966.2395n	neg
2951	0.63_980.2386m/z	neg
2952	0.63_988.2291m/z	neg
2953	0.63_996.2196m/z	neg
2954	0.63_997.7181m/z	neg
2955	0.64_146.9968m/z	pos
2956	0.64_151.0358m/z	pos
2957	0.64_157.9208n	pos
2958	0.64_167.0145m/z	pos
2959	0.64_182.9032m/z	pos
2960	0.64_186.9237m/z	pos
2961	0.64_202.9353m/z	pos
2962	0.64_214.2615m/z	pos
2963	0.64_219.8994m/z	pos
2964	0.64_233.0697m/z	pos
2965	0.64_243.9635m/z	pos
2966	0.64_295.8900n	pos
2967	0.64_316.8821m/z	pos

2968	0.64_332.8920m/z	pos
2969	0.64_361.8814n	pos
2970	0.64_365.8742n	pos
2971	0.64_386.8939m/z	pos
2972	0.64_409.8577m/z	pos
2973	0.64_430.8634m/z	pos
2974	0.64_463.8478n	pos
2975	0.64_466.8479m/z	pos
2976	0.64_491.8265m/z	pos
2977	0.64_494.8303n	pos
2978	0.64_498.8435m/z	pos
2979	0.64_500.8495n	pos
2980	0.64_515.8489m/z	pos
2981	0.64_559.8190n	pos
2982	0.64_565.8278n	pos
2983	0.64_602.8178n	pos
2984	0.64_621.7877m/z	pos
2985	0.64_652.7911n	pos
2986	0.64_674.8217m/z	pos
2987	0.64_702.7996m/z	pos
2988	0.64_709.7917n	pos
2989	0.65_135.9014m/z	neg
2990	0.65_174.4202m/z	neg
2991	0.65_200.9700m/z	neg
2992	0.65_214.9349m/z	neg

2993	0.65_504.97 69m/z	neg
2994	0.65_588.83 69m/z	neg
2995	0.65_617.84 55n	neg
2996	0.65_633.82 45n	neg
2997	0.66_158.97 81m/z	neg
2998	0.66_179.97 85m/z	neg
2999	0.66_242.94 27m/z	neg
3000	0.66_245.06 22m/z	neg
3001	0.66_259.93 30m/z	neg
3002	0.66_329.02 59m/z	neg
3003	0.66_356.92 26m/z	neg
3004	0.66_372.89 99m/z	neg
3005	0.66_375.01 89m/z	neg
3006	0.66_390.89 55n	neg
3007	0.66_408.93 18m/z	neg
3008	0.66_424.90 97m/z	neg
3009	0.66_440.88 74m/z	neg
3010	0.66_457.87 67m/z	neg
3011	0.66_465.00 05m/z	neg
3012	0.66_476.91 79m/z	neg
3013	0.66_479.01 59m/z	neg
3014	0.66_487.88 39m/z	neg
3015	0.66_492.89 66m/z	neg
3016	0.66_502.85 58m/z	neg
3017	0.66_508.87 31m/z	neg

3018	0.66_544.9018m/z	neg
3019	0.66_560.8840m/z	neg
3020	0.66_570.8417m/z	neg
3021	0.66_576.8626m/z	neg
3022	0.66_593.8515m/z	neg
3023	0.66_593.8619n	neg
3024	0.66_594.9598m/z	neg
3025	0.66_644.8493m/z	neg
3026	0.66_647.8875n	neg
3027	0.66_708.8383n	neg
3028	0.66_712.8213m/z	neg
3029	0.66_768.7860m/z	neg
3030	0.66_780.8018m/z	neg
3031	0.66_836.7789m/z	neg
3032	0.67_181.9671n	neg
3033	0.67_181.9692n	pos
3034	0.67_186.9301m/z	neg
3035	0.67_202.1188n	pos
3036	0.67_243.9399n	pos
3037	0.67_249.9539n	neg
3038	0.67_265.9312n	neg
3039	0.67_267.0566m/z	neg
3040	0.67_276.0522n	pos
3041	0.67_311.9274n	pos
3042	0.67_333.9184n	neg

3043	0.67_379.9113n	pos
3044	0.67_387.9092n	neg
3045	0.67_394.8874m/z	neg
3046	0.67_397.8910m/z	pos
3047	0.67_430.9257m/z	neg
3048	0.67_446.9037m/z	neg
3049	0.67_462.8771m/z	neg
3050	0.67_469.8841n	pos
3051	0.67_480.8466m/z	pos
3052	0.67_486.8673m/z	pos
3053	0.67_502.8523m/z	pos
3054	0.67_538.8795m/z	pos
3055	0.67_546.8395m/z	neg
3056	0.67_566.9005m/z	neg
3057	0.67_571.8602n	pos
3058	0.67_582.8784m/z	neg
3059	0.67_595.8361n	pos
3060	0.67_598.8519m/z	neg
3061	0.67_606.8657m/z	pos
3062	0.67_615.1430m/z	pos
3063	0.67_615.8372n	neg
3064	0.67_622.8297m/z	pos
3065	0.67_638.8123m/z	pos
3066	0.67_653.9048n	neg
3067	0.67_666.9876m/z	pos

3068	0.67_686.8179m/z	pos
3069	0.67_702.8686m/z	neg
3070	0.67_718.8512m/z	neg
3071	0.67_734.8229m/z	neg
3072	0.67_764.3474m/z	neg
3073	0.67_809.8085n	neg
3074	0.67_814.3169m/z	neg
3075	0.67_821.8250n	neg
3076	0.67_870.7977m/z	neg
3077	0.68_154.0725n	pos
3078	0.68_168.0884n	pos
3079	0.68_249.9532n	pos
3080	0.68_281.9084n	pos
3081	0.68_304.0637n	pos
3082	0.68_340.9346m/z	pos
3083	0.68_348.9907m/z	pos
3084	0.68_373.9145n	pos
3085	0.68_389.0200m/z	pos
3086	0.68_401.9092n	pos
3087	0.68_408.9217m/z	pos
3088	0.68_456.8483m/z	pos
3089	0.68_475.9019n	pos
3090	0.68_510.9878m/z	pos
3091	0.68_516.9987m/z	pos
3092	0.68_521.9068n	pos

3093	0.68_525.8589n	pos
3094	0.68_541.1233m/z	pos
3095	0.68_557.0979m/z	pos
3096	0.68_599.0007m/z	pos
3097	0.68_605.8722n	pos
3098	0.68_612.8834m/z	pos
3099	0.68_673.8598n	pos
3100	0.68_677.8167n	pos
3101	0.68_690.8181m/z	pos
3102	0.68_708.3454m/z	pos
3103	0.68_741.8471n	pos
3104	0.68_775.8233n	pos
3105	0.68_776.3342m/z	pos
3106	0.68_784.3224m/z	pos
3107	0.68_818.3212m/z	pos
3108	0.68_826.8070m/z	pos
3109	0.68_851.8056m/z	pos
3110	0.68_865.8002n	pos
3111	0.68_886.3038m/z	pos
3112	0.68_893.7866n	pos
3113	0.68_899.8016n	pos
3114	0.68_935.7827m/z	pos
3115	0.68_958.7798m/z	pos
3116	0.68_961.7749n	pos
3117	0.68_962.2917m/z	pos

3118	0.68_967.7877n	pos
3119	0.68_988.2822m/z	pos
3120	0.69_112.9846m/z	neg
3121	0.69_129.9751m/z	neg
3122	0.69_135.9753n	pos
3123	0.69_180.9726m/z	neg
3124	0.69_197.9625m/z	neg
3125	0.69_203.9636n	pos
3126	0.69_213.0849m/z	neg
3127	0.69_235.9115n	pos
3128	0.69_244.9254m/z	pos
3129	0.69_248.9601m/z	neg
3130	0.69_254.9178m/z	neg
3131	0.69_264.9317m/z	neg
3132	0.69_271.9517n	pos
3133	0.69_280.8255m/z	neg
3134	0.69_313.2387m/z	pos
3135	0.69_316.9470m/z	neg
3136	0.69_326.8900m/z	pos
3137	0.69_332.9202m/z	neg
3138	0.69_333.9326m/z	neg
3139	0.69_339.9392n	pos
3140	0.69_341.9265n	neg
3141	0.69_349.0586m/z	neg
3142	0.69_350.9902m/z	pos

3143	0.69_384.9340m/z	neg
3144	0.69_390.8895m/z	neg
3145	0.69_394.8759m/z	pos
3146	0.69_400.9074m/z	neg
3147	0.69_416.8810m/z	neg
3148	0.69_430.9161m/z	pos
3149	0.69_444.1149n	pos
3150	0.69_452.9211m/z	neg
3151	0.69_462.8612m/z	pos
3152	0.69_463.8934n	pos
3153	0.69_468.8948m/z	neg
3154	0.69_471.0177m/z	neg
3155	0.69_475.9102n	neg
3156	0.69_485.0339m/z	neg
3157	0.69_485.8754n	neg
3158	0.69_491.8885n	pos
3159	0.69_493.8679n	neg
3160	0.69_531.0185m/z	pos
3161	0.69_536.8804m/z	neg
3162	0.69_543.9027n	pos
3163	0.69_552.8513m/z	neg
3164	0.69_588.8964m/z	neg
3165	0.69_598.8396m/z	pos
3166	0.69_604.8704m/z	neg
3167	0.69_611.8903n	pos

3168	0.69_620.8441m/z	neg
3169	0.69_631.1161m/z	pos
3170	0.69_656.8835m/z	neg
3171	0.69_662.3614n	pos
3172	0.69_669.8470n	pos
3173	0.69_672.8576m/z	neg
3174	0.69_675.8314n	pos
3175	0.69_676.3501n	pos
3176	0.69_679.8782n	pos
3177	0.69_683.8286n	pos
3178	0.69_688.8265m/z	neg
3179	0.69_693.3402m/z	pos
3180	0.69_704.8060m/z	neg
3181	0.69_724.8699m/z	neg
3182	0.69_727.8398n	pos
3183	0.69_729.8054n	pos
3184	0.69_732.8320m/z	neg
3185	0.69_733.3611m/z	neg
3186	0.69_734.8152m/z	pos
3187	0.69_740.8452m/z	neg
3188	0.69_744.3376n	pos
3189	0.69_744.8468m/z	pos
3190	0.69_746.7968m/z	neg
3191	0.69_749.7879n	pos
3192	0.69_749.8359n	neg

3193	0.69_752.3273n	pos
3194	0.69_756.8188m/z	neg
3195	0.69_760.8212m/z	pos
3196	0.69_763.8401n	pos
3197	0.69_766.8225m/z	neg
3198	0.69_768.3552n	neg
3199	0.69_770.8552m/z	pos
3200	0.69_772.3313n	pos
3201	0.69_778.3309n	pos
3202	0.69_778.8257m/z	pos
3203	0.69_783.3301m/z	neg
3204	0.69_786.3208n	pos
3205	0.69_791.8131n	neg
3206	0.69_793.8420n	neg
3207	0.69_806.3260m/z	neg
3208	0.69_810.3368n	neg
3209	0.69_811.8167n	pos
3210	0.69_817.3240m/z	neg
3211	0.69_821.3273m/z	pos
3212	0.69_825.3115m/z	neg
3213	0.69_828.8098m/z	pos
3214	0.69_83.9613n	pos
3215	0.69_830.7675m/z	neg
3216	0.69_831.7834n	pos
3217	0.69_831.8280n	pos

3218	0.69_833.8048n	neg
3219	0.69_835.8303n	neg
3220	0.69_836.7825m/z	pos
3221	0.69_837.8136n	pos
3222	0.69_843.7826n	pos
3223	0.69_843.8245n	neg
3224	0.69_845.2853m/z	pos
3225	0.69_846.3181n	pos
3226	0.69_847.7964m/z	neg
3227	0.69_850.7995m/z	neg
3228	0.69_852.3199n	neg
3229	0.69_854.3039n	pos
3230	0.69_858.7831m/z	neg
3231	0.69_859.7620n	pos
3232	0.69_863.8270n	pos
3233	0.69_871.2942m/z	pos
3234	0.69_877.3305m/z	neg
3235	0.69_880.3129n	pos
3236	0.69_880.8191m/z	pos
3237	0.69_898.7482m/z	neg
3238	0.69_901.7840n	neg
3239	0.69_903.7748n	pos
3240	0.69_905.8220n	pos
3241	0.69_906.3168n	pos
3242	0.69_908.7676m/z	neg

3243	0.69_911.3145m/z	neg
3244	0.69_911.8018n	neg
3245	0.69_913.8016n	pos
3246	0.69_914.3061n	pos
3247	0.69_919.3051m/z	neg
3248	0.69_922.2956n	pos
3249	0.69_923.7775n	neg
3250	0.69_945.8050n	neg
3251	0.69_947.2759m/z	pos
3252	0.69_947.8403n	neg
3253	0.69_948.8047m/z	pos
3254	0.69_953.2917m/z	neg
3255	0.69_956.2819n	pos
3256	0.69_966.7369m/z	neg
3257	0.69_971.7607n	pos
3258	0.69_972.7461m/z	neg
3259	0.69_973.8083n	pos
3260	0.69_979.3002m/z	neg
3261	0.69_979.7590m/z	neg
3262	0.69_979.7971n	neg
3263	0.69_981.7884n	pos
3264	0.69_982.2933n	pos
3265	0.69_987.2830m/z	neg
3266	0.69_990.2807n	pos
3267	0.69_995.2788m/z	neg

3268	0.69_995.7680n	neg
3269	0.69_997.7673n	pos
3270	0.69_998.2671n	pos
3271	0.70_238.9309m/z	neg
3272	0.70_240.9281m/z	neg
3273	0.70_245.9024n	neg
3274	0.70_248.1934m/z	neg
3275	0.70_271.9010n	neg
3276	0.70_279.8876n	neg
3277	0.70_308.9111m/z	neg
3278	0.70_315.9689m/z	neg
3279	0.70_331.8879n	neg
3280	0.70_366.8730m/z	neg
3281	0.70_374.9062m/z	neg
3282	0.70_380.8543m/z	neg
3283	0.70_405.8380n	neg
3284	0.70_413.8752n	neg
3285	0.70_432.8628m/z	neg
3286	0.70_434.8607m/z	neg
3287	0.70_448.8393m/z	neg
3288	0.70_461.8752n	neg
3289	0.70_473.8244n	neg
3290	0.70_483.8419n	neg
3291	0.70_500.8485m/z	neg
3292	0.70_508.7938m/z	neg

3293	0.70_516.8254m/z	neg
3294	0.70_534.7973m/z	neg
3295	0.70_545.8762n	neg
3296	0.70_548.7817m/z	neg
3297	0.70_551.8229n	neg
3298	0.70_560.8058m/z	neg
3299	0.70_568.8275m/z	neg
3300	0.70_574.7848m/z	neg
3301	0.70_576.7811m/z	neg
3302	0.70_586.8112m/z	neg
3303	0.70_590.7599m/z	neg
3304	0.70_592.7562m/z	neg
3305	0.70_602.7834m/z	neg
3306	0.70_608.7343m/z	neg
3307	0.70_610.8226m/z	neg
3308	0.70_613.8649n	neg
3309	0.70_616.7693m/z	neg
3310	0.70_619.8085n	neg
3311	0.70_626.7995m/z	neg
3312	0.70_629.8004n	neg
3313	0.70_634.7400m/z	neg
3314	0.70_644.7674m/z	neg
3315	0.70_651.7607n	neg
3316	0.70_661.7937n	neg
3317	0.70_668.7840m/z	neg

3318	0.70_677.7639n	neg
3319	0.70_684.7666m/z	neg
3320	0.70_702.7280m/z	neg
3321	0.70_719.7587n	neg
3322	0.70_729.7773n	neg
3323	0.70_745.7548n	neg
3324	0.70_789.7859n	neg
3325	0.70_854.6931m/z	neg
3326	0.70_990.6580m/z	neg
3327	0.71_186.9140m/z	pos
3328	0.71_190.9125m/z	pos
3329	0.71_196.0938n	pos
3330	0.71_209.9088n	pos
3331	0.71_211.9054n	pos
3332	0.71_216.9241m/z	pos
3333	0.71_236.8944m/z	pos
3334	0.71_266.8433m/z	pos
3335	0.71_342.8671m/z	pos
3336	0.71_345.8854n	pos
3337	0.71_352.8994m/z	pos
3338	0.71_387.8688n	pos
3339	0.71_397.8985n	pos
3340	0.71_416.8032m/z	pos
3341	0.71_422.8855m/z	pos
3342	0.71_445.8339n	pos

3343	0.71_452.8425m/z	pos
3344	0.71_454.8319m/z	pos
3345	0.71_455.8619n	pos
3346	0.71_481.8631n	pos
3347	0.71_488.8752m/z	pos
3348	0.71_513.8297n	pos
3349	0.71_523.8486n	pos
3350	0.71_544.7588m/z	pos
3351	0.71_555.7986n	pos
3352	0.71_556.8617m/z	pos
3353	0.71_564.8114m/z	pos
3354	0.71_573.7706n	pos
3355	0.71_591.8297n	pos
3356	0.71_597.7784n	pos
3357	0.71_603.7290n	pos
3358	0.71_622.7686m/z	pos
3359	0.71_624.8490m/z	pos
3360	0.71_626.8389m/z	pos
3361	0.71_632.8018m/z	pos
3362	0.71_633.8137n	pos
3363	0.71_651.7923n	pos
3364	0.71_657.8307n	pos
3365	0.71_659.8166n	pos
3366	0.71_663.7761n	pos
3367	0.71_665.7688n	pos

3368	0.71_667.7655n	pos
3369	0.71_691.7720n	pos
3370	0.71_693.7718n	pos
3371	0.71_706.7307m/z	pos
3372	0.71_717.7795n	pos
3373	0.71_719.7867n	pos
3374	0.71_724.7912m/z	pos
3375	0.71_736.7654m/z	pos
3376	0.71_768.7901m/z	pos
3377	0.71_775.7469n	pos
3378	0.71_782.7493m/z	pos
3379	0.71_783.7752n	pos
3380	0.71_791.7715n	pos
3381	0.71_804.7415m/z	pos
3382	0.71_807.7523n	pos
3383	0.71_827.7529n	pos
3384	0.71_829.7484n	pos
3385	0.71_833.7532n	pos
3386	0.71_837.7473m/z	pos
3387	0.71_875.7427n	pos
3388	0.71_878.7733m/z	pos
3389	0.71_894.7206m/z	pos
3390	0.71_901.7397n	pos
3391	0.71_905.7275n	pos
3392	0.71_908.7029m/z	pos

3393	0.71_909.7244n	pos
3394	0.71_918.7231m/z	pos
3395	0.71_936.7028m/z	pos
3396	0.71_937.7440n	pos
3397	0.71_951.7148n	pos
3398	0.71_955.6928n	pos
3399	0.71_962.7050m/z	pos
3400	0.71_969.6876n	pos
3401	0.71_982.6995m/z	pos
3402	0.71_988.6943m/z	pos
3403	0.71_998.6927m/z	pos
3404	0.72_104.9530m/z	neg
3405	0.72_112.8956m/z	pos
3406	0.72_114.8931m/z	pos
3407	0.72_170.9437m/z	neg
3408	0.72_177.0531n	pos
3409	0.72_199.8786n	pos
3410	0.72_206.8923m/z	pos
3411	0.72_210.8888m/z	pos
3412	0.72_212.8455m/z	pos
3413	0.72_218.8734m/z	neg
3414	0.72_220.8704m/z	neg
3415	0.72_226.8639m/z	pos
3416	0.72_228.9021m/z	neg
3417	0.72_230.8991m/z	neg

3418	0.72_231.8338n	pos
3419	0.72_240.8446m/z	pos
3420	0.72_253.8875n	neg
3421	0.72_264.8451m/z	pos
3422	0.72_276.8316m/z	neg
3423	0.72_278.8287m/z	neg
3424	0.72_286.8605m/z	neg
3425	0.72_288.8576m/z	neg
3426	0.72_296.8880m/z	neg
3427	0.72_369.8065n	pos
3428	0.72_409.2738m/z	pos
3429	0.72_418.7989m/z	pos
3430	0.72_442.8140m/z	pos
3431	0.72_601.7304n	pos
3432	0.72_610.7391m/z	pos
3433	0.72_617.7129n	pos
3434	0.72_620.6284m/z	neg
3435	0.72_625.7191n	neg
3436	0.72_628.6618m/z	neg
3437	0.72_633.6967n	neg
3438	0.72_644.6890m/z	pos
3439	0.72_645.7126n	pos
3440	0.72_649.6728n	neg
3441	0.72_651.6790n	pos
3442	0.72_655.7401n	pos

3443	0.72_659.6992n	neg
3444	0.72_660.6681m/z	pos
3445	0.72_663.6610n	neg
3446	0.72_665.6474n	neg
3447	0.72_671.7191n	pos
3448	0.72_673.6748n	neg
3449	0.72_675.6704n	neg
3450	0.72_676.6465m/z	pos
3451	0.72_679.6369n	neg
3452	0.72_679.6597n	pos
3453	0.72_686.6292m/z	neg
3454	0.72_687.6964n	pos
3455	0.72_698.6366m/z	neg
3456	0.72_699.6530n	pos
3457	0.72_703.6724n	pos
3458	0.72_705.6627n	pos
3459	0.72_707.6306n	neg
3460	0.72_709.6326n	neg
3461	0.72_711.6085m/z	neg
3462	0.72_712.6251m/z	neg
3463	0.72_713.6988n	pos
3464	0.72_721.6325n	neg
3465	0.72_723.6062n	neg
3466	0.72_729.6772n	pos
3467	0.72_733.6295n	neg

3468	0.72_737.5920m/z	neg
3469	0.72_740.6084m/z	neg
3470	0.72_740.6679m/z	pos
3471	0.72_749.6115n	neg
3472	0.72_756.5893m/z	neg
3473	0.72_756.6522m/z	pos
3474	0.72_759.6076n	pos
3475	0.72_764.6808m/z	pos
3476	0.72_765.5939n	neg
3477	0.72_771.6576n	pos
3478	0.72_782.5791m/z	neg
3479	0.72_791.5897n	neg
3480	0.72_797.6265n	pos
3481	0.72_799.5904n	neg
3482	0.72_801.5889n	pos
3483	0.72_804.5467m/z	neg
3484	0.72_807.5726n	neg
3485	0.72_813.6111n	pos
3486	0.72_815.5936n	neg
3487	0.72_824.5740m/z	neg
3488	0.72_845.5956n	pos
3489	0.72_847.5376m/z	neg
3490	0.72_849.5365m/z	neg
3491	0.72_853.6007n	neg
3492	0.72_855.5342m/z	neg

3493	0.72_855.5919n	pos
3494	0.72_859.5734n	neg
3495	0.72_866.5425m/z	neg
3496	0.72_871.5978n	pos
3497	0.72_879.5815n	pos
3498	0.72_881.5185m/z	neg
3499	0.72_882.5235m/z	neg
3500	0.72_885.5925n	pos
3501	0.72_887.5785n	pos
3502	0.72_899.5991n	pos
3503	0.72_904.5671m/z	pos
3504	0.72_907.5194n	neg
3505	0.72_908.5058m/z	neg
3506	0.72_913.5763n	pos
3507	0.72_915.5827n	pos
3508	0.72_920.5631m/z	pos
3509	0.72_933.4971n	neg
3510	0.72_935.5001n	neg
3511	0.72_940.4887m/z	neg
3512	0.72_940.5616m/z	pos
3513	0.72_943.5202n	neg
3514	0.72_943.5703n	pos
3515	0.72_945.5567n	pos
3516	0.72_947.5609n	pos
3517	0.72_951.5077n	neg

3518	0.72_965.5004n	neg
3519	0.72_967.5622n	pos
3520	0.72_972.5339m/z	pos
3521	0.72_973.5039n	neg
3522	0.72_973.5498n	pos
3523	0.72_977.4940n	neg
3524	0.72_982.4620m/z	neg
3525	0.72_985.4909n	neg
3526	0.72_993.4781n	neg
3527	0.72_994.5333m/z	pos
3528	0.72_997.5393n	pos
3529	0.73_110.0724m/z	pos
3530	0.73_110.8981m/z	neg
3531	0.73_141.9415n	pos
3532	0.73_150.8859m/z	neg
3533	0.73_152.8832m/z	neg
3534	0.73_157.0888n	pos
3535	0.73_170.0934m/z	pos
3536	0.73_177.0258m/z	pos
3537	0.73_192.9974m/z	pos
3538	0.73_196.8726m/z	pos
3539	0.73_198.8634m/z	pos
3540	0.73_208.8446m/z	neg
3541	0.73_210.8415m/z	neg
3542	0.73_212.8388m/z	neg

3543	0.73_226.8155m/z	neg
3544	0.73_256.8221m/z	pos
3545	0.73_266.8031m/z	neg
3546	0.73_268.8003m/z	neg
3547	0.73_271.8046n	neg
3548	0.73_272.7946m/z	neg
3549	0.73_284.7737m/z	neg
3550	0.73_286.7712m/z	neg
3551	0.73_288.7685m/z	neg
3552	0.73_300.7476m/z	neg
3553	0.73_301.7945n	neg
3554	0.73_321.7581n	neg
3555	0.73_325.7692n	neg
3556	0.73_326.7594m/z	neg
3557	0.73_342.7320m/z	neg
3558	0.73_379.7184n	neg
3559	0.73_384.7224m/z	neg
3560	0.73_386.7184m/z	neg
3561	0.73_401.6984n	neg
3562	0.73_442.6787m/z	neg
3563	0.73_444.6752m/z	neg
3564	0.73_459.6571n	neg
3565	0.73_460.6470m/z	neg
3566	0.73_475.6305n	neg
3567	0.73_476.6200m/z	neg

3568	0.73_478.6164m/z	neg
3569	0.73_481.6565n	neg
3570	0.73_500.6367m/z	neg
3571	0.73_502.6336m/z	neg
3572	0.73_518.6057m/z	neg
3573	0.73_532.5827m/z	neg
3574	0.73_535.6266n	neg
3575	0.73_558.5958m/z	neg
3576	0.73_575.5751n	neg
3577	0.73_579.5696n	neg
3578	0.73_581.6069n	neg
3579	0.73_590.5420m/z	neg
3580	0.73_595.5829n	neg
3581	0.73_606.5231m/z	neg
3582	0.73_611.5562n	neg
3583	0.73_619.5957n	neg
3584	0.73_626.5324m/z	neg
3585	0.73_631.5302n	neg
3586	0.73_633.5351n	neg
3587	0.73_635.5321n	neg
3588	0.73_640.5173m/z	neg
3589	0.73_649.5115n	neg
3590	0.73_655.5397n	neg
3591	0.73_664.4948m/z	neg
3592	0.73_668.4748m/z	neg

3593	0.73_668.5058m/z	pos
3594	0.73_671.5129n	neg
3595	0.73_691.5067n	neg
3596	0.73_700.4742m/z	neg
3597	0.73_711.4621n	neg
3598	0.73_712.4539m/z	neg
3599	0.73_715.4636n	neg
3600	0.73_725.4830n	neg
3601	0.73_745.4476n	neg
3602	0.73_749.4546n	neg
3603	0.73_759.4327m/z	neg
3604	0.73_767.4277n	neg
3605	0.73_771.4587n	neg
3606	0.73_772.4192m/z	neg
3607	0.73_782.4083m/z	neg
3608	0.73_796.4137m/z	neg
3609	0.73_805.3793n	pos
3610	0.73_805.4118n	neg
3611	0.73_807.4063n	neg
3612	0.73_809.4069n	neg
3613	0.73_810.3977m/z	neg
3614	0.73_811.3879n	pos
3615	0.73_813.3854n	pos
3616	0.73_819.3882n	pos
3617	0.73_826.3880m/z	pos

3618	0.73_827.3613n	pos
3619	0.73_830.3660m/z	pos
3620	0.73_832.3638m/z	pos
3621	0.73_832.3681m/z	neg
3622	0.73_835.4063n	neg
3623	0.73_841.3601n	neg
3624	0.73_843.3927n	neg
3625	0.73_845.3900n	neg
3626	0.73_847.3872n	neg
3627	0.73_849.3859n	neg
3628	0.73_857.3781n	neg
3629	0.73_860.3220m/z	neg
3630	0.73_870.3509m/z	neg
3631	0.73_874.3325m/z	neg
3632	0.73_881.3467n	neg
3633	0.73_883.3562n	neg
3634	0.73_885.3392n	neg
3635	0.73_891.3537n	neg
3636	0.73_898.3183m/z	neg
3637	0.73_901.3202n	neg
3638	0.73_902.3061m/z	neg
3639	0.73_904.3104m/z	neg
3640	0.73_907.3202m/z	neg
3641	0.73_921.3050m/z	neg
3642	0.73_933.3043n	neg

3643	0.73_934.3012m/z	neg
3644	0.73_941.3150n	neg
3645	0.73_943.3267n	neg
3646	0.73_945.2945n	neg
3647	0.73_948.2866m/z	neg
3648	0.73_960.2649m/z	neg
3649	0.73_970.2799m/z	neg
3650	0.73_972.2724m/z	neg
3651	0.73_982.2689m/z	neg
3652	0.73_984.2754m/z	neg
3653	0.73_999.2800n	neg
3654	0.75_108.9008m/z	neg
3655	0.75_154.8803m/z	neg
3656	0.75_184.0251n	pos
3657	0.75_191.0412m/z	pos
3658	0.75_234.8658m/z	neg
3659	0.75_260.9904m/z	neg
3660	0.75_294.8215m/z	neg
3661	0.75_308.7994m/z	neg
3662	0.75_309.9971n	pos
3663	0.75_325.9596n	pos
3664	0.75_350.7833m/z	neg
3665	0.75_352.7809m/z	neg
3666	0.75_368.7550m/z	neg
3667	0.75_368.9602m/z	pos

3668	0.75_427.7604n	neg
3669	0.75_429.7568n	neg
3670	0.75_468.6954m/z	neg
3671	0.75_484.6710m/z	neg
3672	0.76_106.0513m/z	pos
3673	0.76_124.0063m/z	neg
3674	0.76_146.0684n	neg
3675	0.76_152.0328m/z	pos
3676	0.76_154.9270m/z	neg
3677	0.76_158.8458m/z	neg
3678	0.76_160.8418m/z	neg
3679	0.76_162.8387m/z	neg
3680	0.76_164.8354m/z	neg
3681	0.76_166.8334m/z	neg
3682	0.76_168.0512n	pos
3683	0.76_183.9623m/z	neg
3684	0.76_189.1357m/z	pos
3685	0.76_193.8144m/z	neg
3686	0.76_195.8110m/z	neg
3687	0.76_197.8078m/z	neg
3688	0.76_199.8048m/z	neg
3689	0.76_201.8012m/z	neg
3690	0.76_236.8629m/z	neg
3691	0.76_265.0184m/z	neg
3692	0.76_292.8242m/z	neg

3693	0.76_310.7968m/z	neg
3694	0.76_351.1379m/z	pos
3695	0.76_400.0522m/z	pos
3696	0.76_610.1887m/z	pos
3697	0.77_147.0518n	neg
3698	0.77_153.0581m/z	pos
3699	0.77_156.9242m/z	neg
3700	0.77_170.8996m/z	neg
3701	0.77_177.8452m/z	neg
3702	0.77_192.1164n	pos
3703	0.77_203.7981m/z	neg
3704	0.77_207.8359m/z	neg
3705	0.77_212.8849m/z	neg
3706	0.77_217.9348n	neg
3707	0.77_224.8564n	neg
3708	0.77_243.9641m/z	neg
3709	0.77_250.8397m/z	neg
3710	0.77_263.0289m/z	pos
3711	0.77_265.0273m/z	pos
3712	0.77_271.1024m/z	neg
3713	0.77_271.9782m/z	pos
3714	0.77_272.1352m/z	pos
3715	0.77_272.8403m/z	neg
3716	0.77_275.8933n	neg
3717	0.77_282.0052m/z	pos

3718	0.77_286.0594m/z	neg
3719	0.77_298.0027n	pos
3720	0.77_301.9203m/z	neg
3721	0.77_306.2179m/z	pos
3722	0.77_308.1091m/z	pos
3723	0.77_322.1476m/z	pos
3724	0.77_333.9740m/z	pos
3725	0.77_337.0886m/z	neg
3726	0.77_347.7806n	neg
3727	0.77_350.0282m/z	neg
3728	0.77_354.0701m/z	neg
3729	0.77_372.1451n	pos
3730	0.77_382.9868m/z	pos
3731	0.77_396.9167m/z	pos
3732	0.77_397.1087m/z	neg
3733	0.77_409.9785m/z	neg
3734	0.77_454.0781n	pos
3735	0.77_484.1068n	pos
3736	0.78_138.9130m/z	neg
3737	0.78_141.8673m/z	neg
3738	0.78_144.8688m/z	neg
3739	0.78_146.8659m/z	neg
3740	0.78_148.8631m/z	neg
3741	0.78_159.8584m/z	neg
3742	0.78_168.0277m/z	neg

3743	0.78_168.8572m/z	neg
3744	0.78_172.8977m/z	neg
3745	0.78_174.0875m/z	neg
3746	0.78_211.0809m/z	neg
3747	0.78_224.8193m/z	neg
3748	0.78_225.9856m/z	neg
3749	0.78_228.8556m/z	neg
3750	0.78_230.8540m/z	neg
3751	0.78_262.9916m/z	neg
3752	0.78_266.0030m/z	neg
3753	0.78_275.0077m/z	neg
3754	0.78_287.9842m/z	neg
3755	0.78_288.8132m/z	neg
3756	0.78_290.9793m/z	neg
3757	0.78_327.1395m/z	neg
3758	0.78_339.0699m/z	neg
3759	0.78_367.1029m/z	neg
3760	0.78_370.1389m/z	neg
3761	0.78_394.9667m/z	neg
3762	0.79_146.0592n	pos
3763	0.79_158.0695n	pos
3764	0.79_182.0822n	pos
3765	0.79_194.5735m/z	pos
3766	0.79_199.0920m/z	pos
3767	0.79_204.4439m/z	pos

3768	0.79_214.0643m/z	pos
3769	0.79_234.1603n	pos
3770	0.79_293.6032m/z	pos
3771	0.79_365.1651m/z	pos
3772	0.79_386.1417n	pos
3773	0.79_394.1417m/z	pos
3774	0.79_416.1018n	pos
3775	0.79_417.0715n	pos
3776	0.79_431.0857n	pos
3777	0.79_497.0526m/z	pos
3778	0.79_515.0513n	pos
3779	0.79_518.0930n	pos
3780	0.79_522.0713n	pos
3781	0.80_125.8723m/z	neg
3782	0.80_131.0814m/z	neg
3783	0.80_134.8938m/z	neg
3784	0.80_136.8918m/z	neg
3785	0.80_161.6206m/z	pos
3786	0.80_171.0831n	pos
3787	0.80_180.5170m/z	neg
3788	0.80_184.0788m/z	neg
3789	0.80_189.0465n	neg
3790	0.80_197.2009m/z	neg
3791	0.80_202.5628m/z	pos
3792	0.80_216.3812m/z	neg

3793	0.80_218.3722m/z	neg
3794	0.80_253.0088m/z	neg
3795	0.80_267.9602m/z	neg
3796	0.80_269.9593m/z	neg
3797	0.80_271.9564m/z	neg
3798	0.80_283.9430m/z	neg
3799	0.80_303.9217m/z	neg
3800	0.80_305.9347m/z	neg
3801	0.80_307.9325m/z	neg
3802	0.80_309.9308m/z	neg
3803	0.80_323.2208m/z	pos
3804	0.80_323.9768m/z	neg
3805	0.80_339.8967m/z	neg
3806	0.80_341.9046m/z	neg
3807	0.80_343.9049m/z	neg
3808	0.80_344.1343n	pos
3809	0.80_358.9784m/z	neg
3810	0.80_383.9705m/z	pos
3811	0.80_427.0813m/z	pos
3812	0.80_429.0924m/z	neg
3813	0.80_434.1877m/z	pos
3814	0.80_442.0856n	pos
3815	0.80_445.0586m/z	neg
3816	0.80_483.0976m/z	neg
3817	0.80_483.1955n	pos

3818	0.80_485.0306m/z	neg
3819	0.80_487.0249m/z	neg
3820	0.80_491.0254m/z	neg
3821	0.80_512.9887m/z	neg
3822	0.80_514.0337n	neg
3823	0.80_516.0366n	neg
3824	0.80_535.1769m/z	pos
3825	0.80_541.0565m/z	neg
3826	0.80_549.0670n	pos
3827	0.80_549.2014m/z	pos
3828	0.80_551.1815m/z	pos
3829	0.80_580.1341m/z	pos
3830	0.80_602.1979m/z	neg
3831	0.80_665.1191m/z	pos
3832	0.80_709.2373m/z	pos
3833	0.80_844.1778n	pos
3834	0.80_846.1788n	pos
3835	0.81_116.9274m/z	neg
3836	0.81_146.0810m/z	neg
3837	0.81_149.0445m/z	neg
3838	0.81_240.0825n	neg
3839	0.81_258.0701m/z	neg
3840	0.81_372.9995m/z	neg
3841	0.81_377.9282m/z	neg
3842	0.81_401.9518m/z	neg

3843	0.81_402.0897n	neg
3844	0.81_425.0461m/z	neg
3845	0.81_433.0170m/z	neg
3846	0.81_434.9984m/z	neg
3847	0.81_439.0571m/z	neg
3848	0.81_452.1867m/z	neg
3849	0.81_456.0757n	neg
3850	0.81_468.1365m/z	neg
3851	0.81_469.9853m/z	neg
3852	0.81_474.1665m/z	neg
3853	0.81_511.9985n	neg
3854	0.81_518.0719n	neg
3855	0.81_521.0297m/z	neg
3856	0.81_523.0268m/z	neg
3857	0.81_540.2043m/z	neg
3858	0.81_548.0545m/z	neg
3859	0.81_568.9496m/z	neg
3860	0.81_570.9471m/z	neg
3861	0.81_573.9907n	neg
3862	0.81_598.1628m/z	neg
3863	0.81_600.1644m/z	neg
3864	0.81_656.1199m/z	neg
3865	0.81_681.1548m/z	neg
3866	0.81_695.1510m/z	neg
3867	0.81_710.1072m/z	neg

3868	0.81_717.1307m/z	neg
3869	0.81_739.1144m/z	neg
3870	0.81_746.0838m/z	neg
3871	0.81_768.0664m/z	neg
3872	0.81_781.1906m/z	neg
3873	0.81_788.2417m/z	neg
3874	0.81_839.1491m/z	neg
3875	0.81_841.1494m/z	neg
3876	0.81_971.2667m/z	neg
3877	0.82_234.0966m/z	pos
3878	0.82_283.5904m/z	pos
3879	0.82_314.0757n	pos
3880	0.82_498.1320n	pos
3881	0.82_527.0848n	pos
3882	0.82_601.1447m/z	pos
3883	0.82_606.1680n	pos
3884	0.82_628.1461n	pos
3885	0.82_658.2082n	pos
3886	0.82_659.0775n	pos
3887	0.82_680.1902n	pos
3888	0.82_682.1681n	pos
3889	0.82_688.2669n	pos
3890	0.82_689.1388n	pos
3891	0.82_734.1125m/z	pos
3892	0.82_748.1149m/z	pos

3893	0.82_750.0853m/z	pos
3894	0.82_762.1910n	pos
3895	0.82_831.2125m/z	pos
3896	0.82_843.1468m/z	pos
3897	0.82_990.1294m/z	pos
3898	0.83_227.1225m/z	pos
3899	0.83_238.9857m/z	neg
3900	0.83_240.0565m/z	pos
3901	0.83_302.0222m/z	pos
3902	0.83_307.1116m/z	neg
3903	0.83_308.1206n	pos
3904	0.83_315.0821m/z	pos
3905	0.83_316.0533n	pos
3906	0.83_338.0948m/z	pos
3907	0.83_338.5962m/z	pos
3908	0.83_341.1067m/z	neg
3909	0.83_348.0182m/z	pos
3910	0.83_406.1186m/z	neg
3911	0.83_412.0353m/z	neg
3912	0.83_433.1157m/z	neg
3913	0.83_463.9466m/z	neg
3914	0.83_513.1288m/z	pos
3915	0.83_631.1312m/z	pos
3916	0.83_653.0745m/z	neg
3917	0.83_673.1678m/z	neg

3918	0.83_675.1799m/z	pos
3919	0.83_684.0507m/z	pos
3920	0.83_688.1252m/z	neg
3921	0.83_725.1100m/z	neg
3922	0.83_744.2790m/z	neg
3923	0.83_751.1646m/z	pos
3924	0.83_756.0410m/z	neg
3925	0.83_766.2605m/z	neg
3926	0.83_797.1821m/z	neg
3927	0.83_808.0834m/z	neg
3928	0.83_923.2268m/z	pos
3929	0.83_969.1801n	pos
3930	0.83_974.1613m/z	pos
3931	0.83_982.2303m/z	pos
3932	0.84_159.5754m/z	pos
3933	0.84_298.0075m/z	neg
3934	0.84_365.0825m/z	neg
3935	0.84_608.1466n	pos
3936	0.84_624.1324m/z	neg
3937	0.84_624.1671n	pos
3938	0.84_637.2376m/z	pos
3939	0.84_638.1060m/z	pos
3940	0.84_651.0948m/z	neg
3941	0.84_653.1244n	pos
3942	0.84_658.0670m/z	neg

3943	0.84_662.0559m/z	neg
3944	0.84_665.0898m/z	neg
3945	0.84_680.0479m/z	neg
3946	0.84_696.0198m/z	neg
3947	0.84_703.0506n	neg
3948	0.84_703.1259m/z	neg
3949	0.84_915.2468m/z	pos
3950	0.84_922.2103m/z	neg
3951	0.84_925.2070m/z	pos
3952	0.84_930.1981m/z	pos
3953	0.84_941.2072m/z	neg
3954	0.84_943.1881m/z	neg
3955	0.84_945.2053n	pos
3956	0.84_950.1572m/z	neg
3957	0.84_957.1794m/z	neg
3958	0.84_963.1876m/z	neg
3959	0.84_965.1697m/z	neg
3960	0.84_972.1386m/z	neg
3961	0.84_981.1421m/z	neg
3962	0.84_988.1110m/z	neg
3963	0.84_994.1189m/z	neg
3964	0.84_996.1010m/z	neg
3965	0.85_341.9983m/z	neg
3966	0.85_368.0574m/z	neg
3967	0.85_605.1560m/z	neg

3968	0.85_636.08 52m/z	neg
3969	0.85_674.03 97m/z	neg
3970	0.85_710.97 90m/z	neg
3971	0.85_726.95 24m/z	neg
3972	0.85_959.16 11m/z	neg
3973	0.86_901.25 04m/z	pos
3974	0.86_983.11 75m/z	pos
3975	0.87_145.09 66m/z	neg
3976	0.87_157.09 73m/z	neg
3977	0.87_226.97 53m/z	neg
3978	0.87_227.98 29m/z	neg
3979	0.87_246.33 22m/z	neg
3980	0.87_272.23 08m/z	neg
3981	0.87_291.19 30n	neg
3982	0.87_291.93 73m/z	neg
3983	0.87_292.49 21m/z	neg
3984	0.87_300.00 42m/z	neg
3985	0.87_345.00 19n	neg
3986	0.87_352.99 76m/z	neg
3987	0.87_354.99 27m/z	neg
3988	0.87_356.99 13m/z	neg
3989	0.87_414.96 22m/z	neg
3990	0.87_415.96 14m/z	neg
3991	0.87_420.96 33m/z	neg
3992	0.87_584.20 29n	pos

3993	0.87_603.2742m/z	neg
3994	0.87_656.0697m/z	neg
3995	0.87_667.0695m/z	neg
3996	0.87_669.0575m/z	neg
3997	0.87_671.0712m/z	neg
3998	0.87_687.0048m/z	neg
3999	0.87_699.9984m/z	neg
4000	0.87_708.9877m/z	neg
4001	0.88_292.2438m/z	neg
4002	0.88_292.5973m/z	neg
4003	0.88_342.9317m/z	neg
4004	0.88_348.0022m/z	neg
4005	0.88_418.9611m/z	neg
4006	0.88_634.0895m/z	neg
4007	0.89_348.9975m/z	neg
4008	0.89_850.1802m/z	neg
4009	0.89_966.1289m/z	neg
4010	0.91_339.9990m/z	neg
4011	0.91_505.1765m/z	neg
4012	0.91_551.1883m/z	neg
4013	0.91_603.1452m/z	neg
4014	0.91_799.3476m/z	pos
4015	0.91_819.2503m/z	neg
4016	0.91_913.2192m/z	neg
4017	0.91_919.2278m/z	neg

4018	0.91_935.1987m/z	neg
4019	0.92_269.0793m/z	neg
4020	0.92_369.0030m/z	neg
4021	0.92_396.9597m/z	neg
4022	0.92_457.1808m/z	pos
4023	0.92_506.1847n	pos
4024	0.93_146.0283m/z	pos
4025	0.93_168.0627n	neg
4026	0.93_192.0342m/z	pos
4027	0.93_207.0501m/z	neg
4028	0.93_319.0250n	pos
4029	0.93_328.1346n	neg
4030	0.93_329.0363m/z	neg
4031	0.93_332.0564m/z	pos
4032	0.93_345.0045n	pos
4033	0.93_358.0098m/z	neg
4034	0.93_482.1024m/z	neg
4035	0.93_504.0858m/z	neg
4036	0.93_526.0696m/z	neg
4037	0.93_541.1528m/z	neg
4038	0.93_543.1498m/z	neg
4039	0.93_568.1713m/z	neg
4040	0.93_668.2370n	neg
4041	0.93_703.2038m/z	neg
4042	0.94_192.0223m/z	neg

4043	0.94_363.1031m/z	neg
4044	0.94_431.1384m/z	neg
4045	0.94_547.1490m/z	neg
4046	0.95_343.0087n	pos
4047	0.96_141.0659m/z	neg
4048	0.96_154.9961m/z	neg
4049	0.96_185.8446m/z	neg
4050	0.96_191.0179n	pos
4051	0.96_242.0205m/z	pos
4052	0.96_342.0176m/z	pos
4053	0.96_366.9850m/z	neg
4054	0.96_393.0049m/z	neg
4055	0.96_556.9997m/z	neg
4056	0.96_600.0603m/z	neg
4057	0.96_659.0789n	pos
4058	0.96_677.0564m/z	pos
4059	0.96_690.0082n	pos
4060	0.96_702.0285m/z	neg
4061	0.97_129.0561m/z	pos
4062	0.97_144.9651m/z	neg
4063	0.97_158.0046m/z	pos
4064	0.97_277.0407m/z	neg
4065	0.97_285.9883m/z	neg
4066	0.97_318.0531m/z	neg
4067	0.97_324.0050m/z	pos

4068	0.97_367.0024n	pos
4069	0.97_376.0593m/z	neg
4070	0.97_385.9743m/z	pos
4071	0.97_398.0401m/z	neg
4072	0.97_401.9524m/z	neg
4073	0.97_413.0008m/z	pos
4074	0.97_414.0112m/z	neg
4075	0.97_454.9976n	neg
4076	0.97_463.9435m/z	neg
4077	0.97_479.9133m/z	neg
4078	0.98_183.8476m/z	neg
4079	0.98_218.8165m/z	neg
4080	0.98_220.8144m/z	neg
4081	0.98_241.9983m/z	neg
4082	0.98_245.0348m/z	neg
4083	0.98_373.0035m/z	neg
4084	0.98_451.9672m/z	neg
4085	0.98_486.9311m/z	neg
4086	0.99_448.0021n	pos
4087	0.99_530.1792m/z	pos
4088	1.00_101.0590m/z	pos
4089	1.00_146.9641m/z	neg
4090	1.00_157.0865m/z	pos
4091	1.00_186.8589m/z	neg
4092	1.00_267.0384m/z	pos

4093	1.00_411.9812m/z	neg
4094	1.00_422.9769n	neg
4095	1.00_442.9788m/z	pos
4096	1.00_450.9983m/z	pos
4097	1.00_473.9516m/z	neg
4098	1.00_513.9475m/z	neg
4099	1.01_175.9889n	neg
4100	1.01_419.9649m/z	pos
4101	1.01_465.9683m/z	pos
4102	1.01_489.9247m/z	neg
4103	1.03_249.8466m/z	neg
4104	1.03_262.9977m/z	neg
4105	1.03_269.9587m/z	neg
4106	1.03_518.1356n	pos
4107	1.04_166.8329m/z	neg
4108	1.04_183.9701m/z	pos
4109	1.04_233.9561m/z	neg
4110	1.04_475.9955m/z	pos
4111	1.06_193.8145m/z	neg
4112	1.06_460.9203m/z	neg
4113	1.07_145.1216m/z	pos
4114	1.07_334.0436m/z	pos
4115	1.08_112.9563m/z	pos
4116	1.08_150.9821m/z	neg
4117	1.08_168.9479m/z	neg

4118	1.08_176.9732m/z	neg
4119	1.08_180.9731m/z	neg
4120	1.08_191.9457m/z	neg
4121	1.08_205.8387m/z	neg
4122	1.08_207.8359m/z	neg
4123	1.08_308.1170m/z	pos
4124	1.08_358.0095m/z	neg
4125	1.08_89.9893n	neg
4126	1.09_164.9279m/z	neg
4127	1.09_185.8756m/z	neg
4128	1.09_209.8329m/z	neg
4129	1.09_217.8654m/z	neg
4130	1.09_235.9253m/z	neg
4131	1.10_360.0276m/z	pos
4132	1.10_372.0661m/z	pos
4133	1.10_400.9404m/z	pos
4134	1.11_102.0144m/z	pos
4135	1.11_130.9655m/z	neg
4136	1.11_206.9715m/z	neg
4137	1.11_225.9254m/z	neg
4138	1.11_243.2349m/z	pos
4139	1.12_146.0290m/z	pos
4140	1.12_192.0354m/z	pos
4141	1.12_195.9153m/z	pos
4142	1.12_211.9991m/z	neg

4143	1.12_273.9476m/z	neg
4144	1.12_308.2824m/z	pos
4145	1.12_786.2586m/z	pos
4146	1.13_259.8153m/z	neg
4147	1.13_361.0864m/z	neg
4148	1.15_124.9912m/z	pos
4149	1.15_156.9767m/z	pos
4150	1.15_166.9959m/z	neg
4151	1.15_252.0382m/z	pos
4152	1.16_183.9718m/z	pos
4153	1.17_184.0586m/z	neg
4154	1.17_187.8613m/z	neg
4155	1.17_198.0135n	neg
4156	1.17_217.0031n	neg
4157	1.17_236.0031m/z	neg
4158	1.17_240.9355m/z	neg
4159	1.17_242.9341m/z	neg
4160	1.17_246.0287m/z	neg
4161	1.17_249.0304m/z	neg
4162	1.17_255.1321m/z	neg
4163	1.17_260.0014m/z	neg
4164	1.17_265.9095m/z	neg
4165	1.17_268.9624m/z	neg
4166	1.17_275.9671m/z	neg
4167	1.17_281.9844m/z	neg

4168	1.17_294.9738m/z	neg
4169	1.17_299.8898m/z	neg
4170	1.17_316.9566m/z	neg
4171	1.17_330.0485m/z	neg
4172	1.17_335.0765n	neg
4173	1.17_366.1093m/z	neg
4174	1.17_373.0369m/z	neg
4175	1.17_389.0130m/z	neg
4176	1.18_158.0014m/z	pos
4177	1.19_250.1184n	pos
4178	1.19_258.0313m/z	pos
4179	1.19_263.9123m/z	neg
4180	1.19_275.0119m/z	pos
4181	1.19_301.8869m/z	neg
4182	1.19_310.9781m/z	pos
4183	1.19_331.1209n	pos
4184	1.19_332.0614m/z	neg
4185	1.19_334.1881m/z	pos
4186	1.19_444.0873m/z	pos
4187	1.19_459.0659m/z	pos
4188	1.19_488.3973m/z	pos
4189	1.19_646.1778m/z	pos
4190	1.19_710.2408m/z	pos
4191	1.20_101.0234m/z	neg
4192	1.20_350.0579m/z	neg

4193	1.21_113.0231m/z	neg
4194	1.21_115.0755m/z	pos
4195	1.21_116.9648m/z	pos
4196	1.21_119.0340m/z	neg
4197	1.21_123.0074n	pos
4198	1.21_142.1001n	pos
4199	1.21_144.0398n	pos
4200	1.21_151.0027n	pos
4201	1.21_168.5229m/z	pos
4202	1.21_173.0126m/z	pos
4203	1.21_174.9936m/z	pos
4204	1.21_187.9806n	pos
4205	1.21_203.5026m/z	pos
4206	1.21_209.0569m/z	pos
4207	1.21_242.5652m/z	pos
4208	1.21_262.0817n	pos
4209	1.21_289.0138m/z	neg
4210	1.21_308.9474m/z	neg
4211	1.21_310.1290n	pos
4212	1.21_312.1440n	pos
4213	1.21_322.0421m/z	neg
4214	1.21_329.9674m/z	neg
4215	1.21_336.0593n	pos
4216	1.21_378.0320n	pos
4217	1.21_385.0354m/z	pos

4218	1.21_395.9643m/z	neg
4219	1.21_404.0510n	pos
4220	1.21_416.0141m/z	neg
4221	1.21_441.1142m/z	pos
4222	1.21_448.0637n	pos
4223	1.21_452.1368n	pos
4224	1.21_460.0344m/z	neg
4225	1.21_468.1240m/z	pos
4226	1.21_477.0853n	neg
4227	1.21_482.1001m/z	neg
4228	1.21_491.0771n	neg
4229	1.21_514.0829m/z	neg
4230	1.21_515.1334n	pos
4231	1.21_524.1429m/z	pos
4232	1.21_529.1756m/z	pos
4233	1.21_543.0238n	pos
4234	1.21_550.0366m/z	pos
4235	1.21_552.0405m/z	neg
4236	1.21_555.9894m/z	neg
4237	1.21_582.0072m/z	pos
4238	1.21_590.0027m/z	neg
4239	1.21_597.1846m/z	pos
4240	1.21_672.1171n	pos
4241	1.21_674.7046m/z	pos
4242	1.21_678.2133m/z	pos

4243	1.21_682.0711m/z	pos
4244	1.21_682.1115m/z	neg
4245	1.21_693.0923m/z	neg
4246	1.21_704.0913m/z	neg
4247	1.21_770.2130m/z	pos
4248	1.21_830.2962n	pos
4249	1.21_919.3137m/z	neg
4250	1.21_991.3355m/z	neg
4251	1.21_993.3593m/z	pos
4252	1.21_997.3104m/z	neg
4253	1.22_110.0706m/z	pos
4254	1.22_110.9764m/z	pos
4255	1.22_115.9592m/z	pos
4256	1.22_178.0367n	pos
4257	1.22_204.0223n	pos
4258	1.22_282.1446n	pos
4259	1.22_336.1212n	pos
4260	1.22_347.0681n	pos
4261	1.22_358.0263m/z	pos
4262	1.22_360.0568n	pos
4263	1.22_379.0109n	pos
4264	1.22_457.0194n	pos
4265	1.22_478.0063m/z	pos
4266	1.22_531.0445n	pos
4267	1.22_553.0530m/z	pos

4268	1.22_559.0163m/z	pos
4269	1.22_841.1545m/z	pos
4270	1.23_114.9973m/z	pos
4271	1.23_124.0144m/z	neg
4272	1.23_143.9507m/z	neg
4273	1.23_155.9512m/z	neg
4274	1.23_166.5098m/z	neg
4275	1.23_167.7979m/z	neg
4276	1.23_185.0305n	neg
4277	1.23_186.0452m/z	neg
4278	1.23_187.9780m/z	neg
4279	1.23_227.9727m/z	neg
4280	1.23_229.9696m/z	neg
4281	1.23_231.9669m/z	neg
4282	1.23_234.9136m/z	neg
4283	1.23_243.9672m/z	neg
4284	1.23_264.9877m/z	neg
4285	1.23_267.9559n	neg
4286	1.23_286.9720m/z	neg
4287	1.23_303.9356m/z	neg
4288	1.23_306.9341m/z	neg
4289	1.23_312.0322n	neg
4290	1.23_335.9803m/z	pos
4291	1.23_374.0137n	neg
4292	1.23_384.9316m/z	neg

4293	1.23_393.0196m/z	neg
4294	1.23_393.9871m/z	pos
4295	1.23_399.9953m/z	neg
4296	1.23_404.0475n	neg
4297	1.23_421.9767m/z	neg
4298	1.23_434.0018m/z	neg
4299	1.23_435.9804m/z	pos
4300	1.23_454.0162m/z	neg
4301	1.23_476.9877m/z	neg
4302	1.23_477.9939m/z	neg
4303	1.23_488.0605n	neg
4304	1.23_499.9733m/z	neg
4305	1.23_525.0543m/z	neg
4306	1.23_534.0033m/z	neg
4307	1.23_541.0241m/z	neg
4308	1.23_556.9965m/z	neg
4309	1.23_563.0129m/z	neg
4310	1.23_566.0579n	neg
4311	1.23_571.0145m/z	neg
4312	1.23_668.0021m/z	neg
4313	1.23_720.0617m/z	neg
4314	1.23_724.0170m/z	neg
4315	1.23_737.0475m/z	neg
4316	1.24_171.9458m/z	neg
4317	1.24_411.9604m/z	neg

4318	1.24_430.0598m/z	neg
4319	1.24_516.0633n	neg
4320	1.25_133.0090m/z	pos
4321	1.25_212.0098m/z	pos
4322	1.25_216.9959m/z	pos
4323	1.25_217.0045m/z	neg
4324	1.25_234.9991m/z	pos
4325	1.25_237.0003m/z	pos
4326	1.25_305.0360m/z	pos
4327	1.25_334.9810m/z	pos
4328	1.25_396.9965m/z	neg
4329	1.25_419.0429m/z	neg
4330	1.25_437.9762m/z	pos
4331	1.25_450.9882m/z	pos
4332	1.25_456.0043m/z	neg
4333	1.25_615.0413m/z	pos
4334	1.26_252.9749m/z	neg
4335	1.26_285.9869m/z	pos
4336	1.26_292.2016m/z	pos
4337	1.26_310.9681m/z	neg
4338	1.26_319.0021m/z	pos
4339	1.26_345.9646m/z	neg
4340	1.26_348.9966m/z	pos
4341	1.26_418.9854m/z	pos
4342	1.26_444.9637m/z	neg

4343	1.26_452.9889m/z	pos
4344	1.26_619.0352m/z	neg
4345	1.27_162.9971n	pos
4346	1.27_191.9824m/z	pos
4347	1.27_198.9841m/z	pos
4348	1.27_243.9610n	pos
4349	1.27_287.9834m/z	pos
4350	1.28_130.0259n	neg
4351	1.28_190.4339m/z	neg
4352	1.28_199.9398m/z	neg
4353	1.28_263.0109m/z	neg
4354	1.28_275.0074m/z	neg
4355	1.28_279.9069m/z	neg
4356	1.28_315.8904m/z	neg
4357	1.28_318.8985m/z	neg
4358	1.28_325.0136m/z	neg
4359	1.28_326.9888m/z	neg
4360	1.28_333.9615m/z	neg
4361	1.28_355.9424m/z	neg
4362	1.28_360.0105n	neg
4363	1.28_403.9708m/z	neg
4364	1.28_407.0050m/z	neg
4365	1.28_420.9971m/z	neg
4366	1.28_428.9798m/z	neg
4367	1.28_435.9574m/z	neg

4368	1.28_442.9785m/z	neg
4369	1.28_450.0246n	neg
4370	1.28_450.9687m/z	neg
4371	1.28_457.9388m/z	neg
4372	1.28_480.9281m/z	neg
4373	1.28_524.9786m/z	neg
4374	1.28_613.0185m/z	neg
4375	1.29_104.0088m/z	pos
4376	1.29_270.9586m/z	neg
4377	1.29_277.9729m/z	neg
4378	1.29_291.9538m/z	pos
4379	1.29_291.9616m/z	neg
4380	1.29_297.9779m/z	neg
4381	1.29_313.9507m/z	neg
4382	1.29_460.9682m/z	pos
4383	1.30_136.0402n	pos
4384	1.30_144.9585m/z	neg
4385	1.30_181.9921m/z	pos
4386	1.30_205.9391m/z	neg
4387	1.30_385.9731m/z	pos
4388	1.30_389.9932m/z	pos
4389	1.32_133.0084m/z	neg
4390	1.32_169.8986m/z	neg
4391	1.32_176.9972m/z	neg
4392	1.32_193.9880m/z	neg

4393	1.32_218.9824m/z	neg
4394	1.33_213.8882m/z	neg
4395	1.33_259.8154m/z	neg
4396	1.35_187.8610m/z	neg
4397	1.35_235.9710m/z	neg
4398	1.37_102.0135m/z	pos
4399	1.42_243.8992m/z	neg
4400	1.42_256.9542m/z	neg
4401	1.42_376.0151m/z	pos
4402	1.43_246.9804n	pos
4403	1.43_312.0069n	pos
4404	1.43_475.0539m/z	pos
4405	1.44_200.9744m/z	pos
4406	1.44_214.0035m/z	pos
4407	1.44_227.9848m/z	pos
4408	1.44_348.9942m/z	pos
4409	1.45_156.9755m/z	pos
4410	1.45_230.9910m/z	pos
4411	1.46_139.0027m/z	pos
4412	1.46_242.0204m/z	neg
4413	1.46_288.8760m/z	neg
4414	1.46_290.8728m/z	neg
4415	1.47_110.0088m/z	pos
4416	1.47_128.0342m/z	neg
4417	1.47_196.0217m/z	neg

4418	1.47_202.9874n	neg
4419	1.47_212.9943n	neg
4420	1.47_216.0089m/z	neg
4421	1.47_217.9305m/z	neg
4422	1.47_227.9590m/z	neg
4423	1.47_252.8998m/z	neg
4424	1.47_254.8964m/z	neg
4425	1.47_263.0214n	neg
4426	1.47_264.9539m/z	neg
4427	1.47_274.9827m/z	neg
4428	1.47_279.0588m/z	neg
4429	1.47_292.8700m/z	neg
4430	1.47_296.0380n	neg
4431	1.47_310.9903n	neg
4432	1.47_331.0001m/z	neg
4433	1.47_339.9928m/z	neg
4434	1.47_358.0185m/z	neg
4435	1.47_424.0654m/z	neg
4436	1.48_182.9695m/z	pos
4437	1.48_307.9442m/z	neg
4438	1.48_373.9900m/z	neg
4439	1.56_174.9920m/z	pos
4440	1.63_154.9670m/z	pos
4441	1.74_308.2825m/z	pos
4442	1.75_174.9920m/z	pos

4443	1.77_105.93 63m/z	pos
4444	1.78_163.98 57m/z	pos
4445	1.79_257.25 00m/z	pos
4446	1.86_195.91 38m/z	pos
4447	1.91_144.95 96m/z	pos
4448	1.92_404.01 58m/z	neg
4449	1.95_204.99 27m/z	neg
4450	1.95_243.89 67m/z	neg
4451	1.96_172.95 42m/z	neg
4452	1.96_201.99 47m/z	neg
4453	1.96_217.96 10m/z	neg
4454	1.96_232.98 81m/z	neg
4455	1.96_233.93 70m/z	neg
4456	1.96_241.88 67m/z	neg
4457	1.96_277.86 03m/z	neg
4458	1.96_281.85 49m/z	neg
4459	1.96_360.02 61m/z	pos
4460	1.98_135.90 66m/z	pos
4461	1.98_234.99 34m/z	pos
4462	1.98_244.07 09n	pos
4463	1.98_271.97 77m/z	pos
4464	1.98_273.97 48m/z	pos
4465	1.98_567.18 01n	pos
4466	10.01_182.9 621m/z	pos
4467	10.01_280.2 605m/z	pos

4468	10.01_356.0018n	neg
4469	10.01_364.9888m/z	neg
4470	10.01_603.1435m/z	pos
4471	10.02_522.3426m/z	neg
4472	10.03_179.1089m/z	pos
4473	10.03_373.2774m/z	pos
4474	10.04_181.1234m/z	pos
4475	10.04_375.2512n	neg
4476	10.04_375.2564n	pos
4477	10.04_407.2979m/z	pos
4478	10.04_412.3088m/z	pos
4479	10.04_434.3156m/z	pos
4480	10.04_516.3691m/z	pos
4481	10.05_430.2515m/z	pos
4482	10.05_582.2593m/z	pos
4483	10.07_484.3712m/z	pos
4484	10.07_581.2503m/z	pos
4485	10.08_370.3007m/z	pos
4486	10.08_416.2703m/z	pos
4487	10.09_191.1443m/z	pos
4488	10.09_443.2815m/z	pos
4489	10.11_194.1324n	pos
4490	10.11_378.0103n	neg
4491	10.11_414.3295n	pos
4492	10.11_418.9927m/z	neg

4493	10.11_445.2949m/z	neg
4494	10.11_447.3138m/z	pos
4495	10.11_468.3084n	pos
4496	10.11_490.2942m/z	pos
4497	10.11_529.2888m/z	neg
4498	10.12_151.1130m/z	pos
4499	10.12_180.1499m/z	pos
4500	10.12_211.1350m/z	pos
4501	10.12_234.1354m/z	pos
4502	10.12_238.1141m/z	pos
4503	10.12_238.3605m/z	pos
4504	10.12_325.9977n	neg
4505	10.12_440.9955m/z	neg
4506	10.12_505.0032m/z	neg
4507	10.12_542.0168n	neg
4508	10.13_159.1395m/z	pos
4509	10.13_271.0876n	pos
4510	10.13_418.2527m/z	pos
4511	10.13_430.8883m/z	pos
4512	10.13_458.2989m/z	pos
4513	10.14_304.9863m/z	neg
4514	10.14_392.9878m/z	neg
4515	10.14_416.0020n	neg
4516	10.14_556.0211n	neg
4517	10.15_313.1814m/z	pos

4518	10.15_391.2867m/z	pos
4519	10.15_529.2139m/z	pos
4520	10.16_155.1099m/z	pos
4521	10.16_244.2679m/z	pos
4522	10.16_289.2310n	pos
4523	10.16_455.3085n	pos
4524	10.17_203.1807m/z	pos
4525	10.17_343.1674m/z	neg
4526	10.18_441.2361m/z	neg
4527	10.18_446.2275m/z	neg
4528	10.19_246.2364n	pos
4529	10.19_330.2480m/z	pos
4530	10.19_379.2521n	pos
4531	10.19_477.2067m/z	pos
4532	10.20_313.2232m/z	pos
4533	10.20_458.9919m/z	neg
4534	10.21_294.1784m/z	neg
4535	10.22_197.1555m/z	pos
4536	10.22_209.1910m/z	pos
4537	10.22_227.5861m/z	pos
4538	10.22_410.1709m/z	pos
4539	10.22_414.2081n	pos
4540	10.22_440.3413m/z	pos
4541	10.22_507.1055m/z	pos
4542	10.22_623.3444m/z	pos

4543	10.23_316.9881m/z	neg
4544	10.23_413.9374m/z	pos
4545	10.23_426.2650m/z	pos
4546	10.23_428.2822m/z	pos
4547	10.23_455.2311n	pos
4548	10.23_460.2720m/z	pos
4549	10.23_476.1754n	pos
4550	10.23_483.2522m/z	pos
4551	10.23_488.3034m/z	pos
4552	10.23_555.3221m/z	pos
4553	10.24_101.9501m/z	pos
4554	10.24_186.1864m/z	pos
4555	10.25_476.0118n	neg
4556	10.26_502.2699m/z	neg
4557	10.26_529.2892m/z	neg
4558	10.27_214.1954n	pos
4559	10.27_245.1894n	pos
4560	10.27_253.6341m/z	pos
4561	10.27_261.6193m/z	pos
4562	10.27_267.2216n	pos
4563	10.27_466.8687m/z	pos
4564	10.27_551.2577n	pos
4565	10.28_288.2564m/z	pos
4566	10.30_165.1284m/z	pos
4567	10.30_251.1717m/z	pos

4568	10.31_115.9624m/z	pos
4569	10.31_540.2632m/z	neg
4570	10.31_561.2180n	pos
4571	10.32_209.1551m/z	pos
4572	10.32_307.2436m/z	pos
4573	10.32_401.3073m/z	pos
4574	10.32_462.3260m/z	pos
4575	10.34_320.9808m/z	neg
4576	10.34_348.9914m/z	pos
4577	10.34_353.9958n	neg
4578	10.34_404.0077n	neg
4579	10.34_431.0846m/z	pos
4580	10.34_440.0129n	neg
4581	10.34_474.2681m/z	neg
4582	10.35_182.9625m/z	pos
4583	10.35_321.2247m/z	pos
4584	10.35_360.0075n	neg
4585	10.35_446.0020n	neg
4586	10.35_480.3123m/z	pos
4587	10.35_490.0032n	neg
4588	10.36_223.1723m/z	pos
4589	10.36_381.2690n	pos
4590	10.36_479.2183m/z	pos
4591	10.36_486.3857m/z	pos
4592	10.37_282.9825m/z	neg

4593	10.38_225.1869m/z	pos
4594	10.38_260.1959m/z	pos
4595	10.38_286.6312m/z	pos
4596	10.39_384.3180m/z	pos
4597	10.39_392.0089n	neg
4598	10.39_410.7645m/z	pos
4599	10.39_452.0127n	neg
4600	10.39_456.3566m/z	pos
4601	10.39_470.2945m/z	pos
4602	10.39_500.0147n	neg
4603	10.40_603.3055m/z	neg
4604	10.41_298.6265m/z	pos
4605	10.41_417.2212m/z	pos
4606	10.41_429.3031m/z	pos
4607	10.41_454.1739m/z	pos
4608	10.41_464.3177n	pos
4609	10.41_508.3274n	neg
4610	10.41_540.0164n	neg
4611	10.41_541.3217n	pos
4612	10.41_556.3259m/z	pos
4613	10.42_254.2151m/z	pos
4614	10.42_277.2175m/z	pos
4615	10.42_290.6425m/z	pos
4616	10.42_437.3755m/z	pos
4617	10.42_511.3326n	pos

4618	10.43_208.1848n	pos
4619	10.43_253.2205m/z	pos
4620	10.43_372.3195m/z	pos
4621	10.43_440.3257m/z	pos
4622	10.43_490.3614m/z	pos
4623	10.44_484.0156n	neg
4624	10.44_496.0131n	neg
4625	10.44_504.0149n	neg
4626	10.44_508.9951m/z	neg
4627	10.44_592.0177n	neg
4628	10.45_260.2154n	pos
4629	10.45_318.2429m/z	pos
4630	10.45_334.2187m/z	pos
4631	10.45_464.3133m/z	pos
4632	10.45_528.3775m/z	pos
4633	10.46_271.1356m/z	pos
4634	10.46_407.2635m/z	pos
4635	10.47_155.8651m/z	neg
4636	10.47_422.3310m/z	pos
4637	10.47_457.3374m/z	pos
4638	10.48_348.9894m/z	neg
4639	10.49_122.9639m/z	pos
4640	10.49_197.1898m/z	pos
4641	10.49_396.3136m/z	pos
4642	10.49_409.2787m/z	pos

4643	10.49_446.3322m/z	pos
4644	10.49_603.5386m/z	pos
4645	10.50_610.2453m/z	neg
4646	10.51_266.6418m/z	pos
4647	10.51_274.6263m/z	pos
4648	10.51_306.2657m/z	pos
4649	10.51_351.1620m/z	neg
4650	10.51_492.8050m/z	pos
4651	10.51_528.2860m/z	neg
4652	10.51_548.2464m/z	pos
4653	10.51_555.3052m/z	neg
4654	10.51_577.2733n	pos
4655	10.51_584.2235m/z	pos
4656	10.51_594.2443m/z	pos
4657	10.52_312.1611m/z	pos
4658	10.52_414.2090n	pos
4659	10.52_417.2217m/z	pos
4660	10.52_473.2702m/z	pos
4661	10.52_498.3644m/z	pos
4662	10.52_504.3296m/z	pos
4663	10.52_555.3224m/z	pos
4664	10.52_640.1687n	pos
4665	10.52_758.4655m/z	neg
4666	10.53_195.1390m/z	pos
4667	10.53_458.2514m/z	pos

4668	10.53_583.2159m/z	pos
4669	10.54_264.3310m/z	neg
4670	10.54_512.0049n	neg
4671	10.54_516.0173n	neg
4672	10.54_527.0002m/z	neg
4673	10.54_536.0180n	neg
4674	10.54_543.0011m/z	neg
4675	10.54_550.0098n	neg
4676	10.54_553.0020m/z	neg
4677	10.54_583.0041m/z	neg
4678	10.54_586.0212n	neg
4679	10.54_590.0198n	neg
4680	10.54_605.0047m/z	neg
4681	10.54_619.0077m/z	neg
4682	10.54_652.0240n	neg
4683	10.55_214.1824m/z	pos
4684	10.55_258.1853n	pos
4685	10.55_323.1488n	pos
4686	10.55_405.1364m/z	pos
4687	10.55_436.0131n	neg
4688	10.55_459.2716m/z	neg
4689	10.55_613.0078m/z	neg
4690	10.56_243.1853n	pos
4691	10.57_234.1365m/z	pos
4692	10.57_376.1935n	pos

4693	10.57_413.2815m/z	pos
4694	10.57_442.3565m/z	pos
4695	10.57_448.2882n	pos
4696	10.57_588.2531m/z	pos
4697	10.58_520.0147n	neg
4698	10.59_275.2030m/z	pos
4699	10.59_297.1528m/z	neg
4700	10.59_310.2168n	pos
4701	10.59_345.2560n	pos
4702	10.59_529.1919n	pos
4703	10.61_141.1139m/z	pos
4704	10.61_254.2131m/z	pos
4705	10.61_286.2397m/z	pos
4706	10.61_386.3290m/z	pos
4707	10.61_398.3334m/z	pos
4708	10.61_477.3078n	pos
4709	10.61_526.0139n	neg
4710	10.61_887.5656m/z	pos
4711	10.63_231.1460n	pos
4712	10.63_268.1243m/z	pos
4713	10.63_293.2362n	pos
4714	10.63_399.7690m/z	pos
4715	10.63_406.7500m/z	pos
4716	10.63_440.2870m/z	pos
4717	10.63_618.0222n	neg

4718	10.64_126.9667m/z	pos
4719	10.64_239.2031m/z	pos
4720	10.64_248.6732n	pos
4721	10.64_539.3226m/z	pos
4722	10.65_505.3122m/z	neg
4723	10.65_567.3404m/z	neg
4724	10.65_581.3212m/z	neg
4725	10.65_636.2612m/z	neg
4726	10.65_649.3086m/z	neg
4727	10.66_260.6411m/z	pos
4728	10.66_268.6272m/z	pos
4729	10.66_279.6498m/z	pos
4730	10.66_288.1370m/z	pos
4731	10.66_462.9929m/z	neg
4732	10.66_481.3228n	pos
4733	10.66_501.2896n	pos
4734	10.66_516.2854m/z	neg
4735	10.66_518.7392m/z	pos
4736	10.66_535.3111n	pos
4737	10.66_571.3577m/z	pos
4738	10.66_583.2771m/z	pos
4739	10.66_594.3026m/z	neg
4740	10.66_614.3372m/z	neg
4741	10.66_619.2602n	pos
4742	10.66_668.1786n	pos

4743	10.66_796.5175m/z	neg
4744	10.67_320.2566m/z	pos
4745	10.67_374.1407m/z	pos
4746	10.67_392.2950n	pos
4747	10.67_661.2611m/z	pos
4748	10.68_294.2224n	pos
4749	10.68_304.3021m/z	pos
4750	10.68_307.2424m/z	pos
4751	10.68_313.2403m/z	pos
4752	10.68_324.2760m/z	pos
4753	10.68_360.2404m/z	pos
4754	10.68_475.2996m/z	pos
4755	10.68_488.2457m/z	pos
4756	10.68_542.6642m/z	pos
4757	10.68_598.2612m/z	pos
4758	10.68_634.2381m/z	pos
4759	10.69_427.2606m/z	neg
4760	10.69_459.2718m/z	neg
4761	10.69_578.3012m/z	neg
4762	10.69_605.3211m/z	neg
4763	10.69_656.3187m/z	neg
4764	10.70_221.1190m/z	pos
4765	10.70_297.1526m/z	neg
4766	10.70_372.2692n	pos
4767	10.70_408.3084m/z	pos

4768	10.70_410.9893m/z	neg
4769	10.70_418.7504m/z	pos
4770	10.70_430.9918m/z	neg
4771	10.70_438.3780m/z	pos
4772	10.70_627.5397m/z	pos
4773	10.71_257.2307m/z	pos
4774	10.71_450.2684n	pos
4775	10.71_462.3401n	pos
4776	10.71_470.3357m/z	pos
4777	10.71_603.5397m/z	pos
4778	10.71_753.5664m/z	pos
4779	10.71_882.4984m/z	pos
4780	10.72_100.9554m/z	pos
4781	10.72_197.1549m/z	pos
4782	10.72_241.8846m/z	pos
4783	10.72_281.2500m/z	pos
4784	10.72_292.2059n	pos
4785	10.72_299.2615m/z	pos
4786	10.72_314.0892n	pos
4787	10.72_339.2608m/z	pos
4788	10.72_348.9931m/z	pos
4789	10.72_411.2690m/z	pos
4790	10.72_419.2544m/z	pos
4791	10.72_472.3465m/z	pos
4792	10.72_498.9334m/z	neg

4793	10.72_537.5394m/z	pos
4794	10.72_871.5797m/z	neg
4795	10.72_967.4044m/z	pos
4796	10.72_969.4272m/z	pos
4797	10.74_390.9858m/z	neg
4798	10.74_397.3151m/z	pos
4799	10.74_405.2526n	pos
4800	10.74_477.2893n	pos
4801	10.74_488.1649n	pos
4802	10.74_509.3385m/z	pos
4803	10.74_532.2151m/z	pos
4804	10.74_580.2149m/z	pos
4805	10.74_617.1926m/z	pos
4806	10.74_742.5656m/z	pos
4807	10.75_263.2324m/z	pos
4808	10.75_266.6116m/z	pos
4809	10.75_303.6504m/z	pos
4810	10.75_476.8239m/z	pos
4811	10.75_566.5922m/z	pos
4812	10.75_596.3002n	pos
4813	10.75_622.2602n	pos
4814	10.75_657.2331m/z	pos
4815	10.75_658.2349m/z	pos
4816	10.75_703.1675m/z	pos
4817	10.75_715.1898m/z	pos

4818	10.75_824.5594m/z	pos
4819	10.75_955.5893m/z	pos
4820	10.75_979.5862m/z	pos
4821	10.76_200.2022m/z	pos
4822	10.76_249.1866m/z	pos
4823	10.76_311.6361m/z	pos
4824	10.76_366.9866m/z	neg
4825	10.76_412.2197m/z	pos
4826	10.76_448.3487m/z	pos
4827	10.76_526.7822m/z	pos
4828	10.76_530.7949m/z	pos
4829	10.76_538.2792m/z	pos
4830	10.76_538.7794m/z	pos
4831	10.76_563.3152m/z	pos
4832	10.76_563.8179m/z	pos
4833	10.76_567.3375n	pos
4834	10.76_585.2553m/z	neg
4835	10.76_619.2624n	pos
4836	10.76_627.3020m/z	pos
4837	10.76_678.4172m/z	pos
4838	10.76_680.3178m/z	neg
4839	10.76_692.4569m/z	pos
4840	10.76_712.3326m/z	pos
4841	10.76_808.5016n	pos
4842	10.76_997.6356m/z	pos

4843	10.77_512.9976m/z	neg
4844	10.77_559.0006m/z	neg
4845	10.77_563.0003m/z	neg
4846	10.77_602.3010m/z	neg
4847	10.77_607.0060m/z	neg
4848	10.77_609.0067m/z	neg
4849	10.77_622.0214n	neg
4850	10.77_623.0031m/z	neg
4851	10.77_639.0088m/z	neg
4852	10.77_646.0259n	neg
4853	10.77_648.0151n	neg
4854	10.77_672.0245n	neg
4855	10.77_674.0220n	neg
4856	10.77_675.0112m/z	neg
4857	10.77_684.0270n	neg
4858	10.77_684.2635m/z	neg
4859	10.77_688.0257n	neg
4860	10.77_707.0126m/z	neg
4861	10.77_800.4730m/z	neg
4862	10.77_899.0186m/z	neg
4863	10.78_248.6708n	pos
4864	10.78_278.6295m/z	pos
4865	10.78_279.6499m/z	pos
4866	10.78_286.6368m/z	pos
4867	10.78_335.1681m/z	pos

4868	10.78_424.3465m/z	pos
4869	10.78_468.2397m/z	pos
4870	10.78_518.7402m/z	pos
4871	10.78_519.3398n	pos
4872	10.78_520.8427n	pos
4873	10.78_534.3243m/z	pos
4874	10.78_539.8202m/z	pos
4875	10.78_547.3055m/z	pos
4876	10.78_547.8055m/z	pos
4877	10.78_548.3319n	pos
4878	10.78_548.8343n	pos
4879	10.78_551.8199m/z	pos
4880	10.78_554.3022m/z	neg
4881	10.78_558.3049m/z	neg
4882	10.78_558.8006m/z	pos
4883	10.78_562.5788m/z	neg
4884	10.78_574.2617m/z	pos
4885	10.78_575.0029m/z	neg
4886	10.78_577.0038m/z	neg
4887	10.78_581.3216m/z	neg
4888	10.78_612.0137n	neg
4889	10.78_615.2654m/z	pos
4890	10.78_616.2932m/z	neg
4891	10.78_624.2873m/z	neg
4892	10.78_636.2612m/z	neg

4893	10.78_643.2897m/z	neg
4894	10.78_644.0260n	neg
4895	10.78_647.4394m/z	pos
4896	10.78_649.3077m/z	neg
4897	10.78_660.0262n	neg
4898	10.78_665.2645n	neg
4899	10.78_680.0173n	neg
4900	10.78_685.0094m/z	neg
4901	10.78_696.0279n	neg
4902	10.78_701.0106m/z	neg
4903	10.78_705.2555m/z	neg
4904	10.78_712.2625m/z	neg
4905	10.78_715.0115m/z	neg
4906	10.78_717.0114m/z	neg
4907	10.78_723.0132m/z	neg
4908	10.78_728.0301n	neg
4909	10.78_739.0141m/z	neg
4910	10.78_765.0157m/z	neg
4911	10.78_783.9585n	pos
4912	10.78_787.0155m/z	neg
4913	10.78_796.0347n	neg
4914	10.78_798.9899m/z	pos
4915	10.78_799.4880m/z	pos
4916	10.78_807.4734m/z	pos
4917	10.78_808.0337n	neg

4918	10.78_810.9810m/z	pos
4919	10.78_812.0359n	neg
4920	10.78_818.9726m/z	pos
4921	10.78_819.4716m/z	pos
4922	10.78_943.6819m/z	pos
4923	10.79_104.1066m/z	pos
4924	10.79_291.6505m/z	pos
4925	10.79_299.6360m/z	pos
4926	10.79_542.6652m/z	pos
4927	10.79_598.2628m/z	pos
4928	10.79_620.3002n	pos
4929	10.80_190.9928m/z	neg
4930	10.80_391.2251m/z	neg
4931	10.80_428.8954m/z	pos
4932	10.80_430.8907m/z	pos
4933	10.80_432.8877m/z	pos
4934	10.80_434.8876m/z	pos
4935	10.80_570.0193n	neg
4936	10.80_586.5033m/z	neg
4937	10.80_622.2902m/z	neg
4938	10.80_643.2802m/z	pos
4939	10.80_667.3094n	neg
4940	10.80_668.0240n	neg
4941	10.80_681.0096m/z	neg
4942	10.80_694.2885m/z	neg

4943	10.80_700.3053m/z	neg
4944	10.80_704.2517m/z	neg
4945	10.80_768.0220n	neg
4946	10.80_810.0350n	neg
4947	10.80_815.0162m/z	neg
4948	10.80_825.0171m/z	neg
4949	10.80_838.0353n	neg
4950	10.80_840.0356n	neg
4951	10.80_886.0372n	neg
4952	10.81_486.9952m/z	neg
4953	10.81_578.3017m/z	neg
4954	10.81_594.3406m/z	neg
4955	10.81_605.3210m/z	neg
4956	10.81_640.2905m/z	neg
4957	10.81_660.2613m/z	neg
4958	10.81_673.3077m/z	neg
4959	10.81_691.0104m/z	neg
4960	10.81_799.0151m/z	neg
4961	10.81_801.0154m/z	neg
4962	10.81_823.0179m/z	neg
4963	10.82_284.2625m/z	pos
4964	10.82_316.3261m/z	pos
4965	10.83_242.2095m/z	pos
4966	10.83_379.2537m/z	pos
4967	10.83_416.2187m/z	pos

4968	10.84_197.1551m/z	pos
4969	10.84_266.1738m/z	pos
4970	10.84_269.2159m/z	pos
4971	10.84_317.2151m/z	pos
4972	10.84_473.2821m/z	neg
4973	10.86_100.9554m/z	pos
4974	10.86_234.1365m/z	pos
4975	10.86_293.1779m/z	pos
4976	10.86_297.2682n	pos
4977	10.86_406.7495m/z	pos
4978	10.86_410.7643m/z	pos
4979	10.86_490.2877m/z	pos
4980	10.86_491.2947m/z	pos
4981	10.86_575.5047m/z	pos
4982	10.86_875.4897m/z	pos
4983	10.86_901.4990m/z	pos
4984	10.86_967.4102m/z	pos
4985	10.87_306.2264n	pos
4986	10.87_347.2251m/z	pos
4987	10.87_384.3269m/z	pos
4988	10.87_565.0004m/z	neg
4989	10.88_435.3105m/z	neg
4990	10.89_304.2531m/z	pos
4991	10.89_363.2782m/z	pos
4992	10.89_382.3252m/z	pos

4993	10.89_466.3389m/z	pos
4994	10.89_467.3272m/z	pos
4995	10.89_637.2595m/z	pos
4996	10.89_679.1731m/z	pos
4997	10.89_828.5504m/z	pos
4998	10.90_184.0743m/z	pos
4999	10.90_222.1638n	pos
5000	10.90_226.1815m/z	pos
5001	10.90_236.6721n	pos
5002	10.90_256.2303m/z	pos
5003	10.90_267.6493m/z	pos
5004	10.90_296.6496m/z	pos
5005	10.90_484.8394n	pos
5006	10.90_494.8146m/z	pos
5007	10.90_495.3373n	pos
5008	10.90_515.8205m/z	pos
5009	10.90_562.2612n	pos
5010	10.90_586.2397m/z	pos
5011	10.90_590.2575n	pos
5012	10.90_595.2594n	pos
5013	10.90_615.2591m/z	pos
5014	10.90_666.4169m/z	pos
5015	10.90_770.9709m/z	pos
5016	10.90_771.4734m/z	pos
5017	10.90_991.6804m/z	pos

5018	10.91_196.1540m/z	pos
5019	10.91_402.2304m/z	pos
5020	10.91_530.3018m/z	neg
5021	10.91_538.6537m/z	neg
5022	10.91_557.3214m/z	neg
5023	10.91_592.2933m/z	neg
5024	10.91_598.2895m/z	neg
5025	10.91_612.2616m/z	neg
5026	10.91_640.2563m/z	neg
5027	10.91_676.3057m/z	neg
5028	10.91_688.2642m/z	neg
5029	10.93_528.3147m/z	pos
5030	10.93_530.2941m/z	pos
5031	10.93_627.5393m/z	pos
5032	10.94_253.1813m/z	pos
5033	10.94_276.2110n	pos
5034	10.94_291.1982m/z	pos
5035	10.94_312.2323n	pos
5036	10.94_369.1589m/z	pos
5037	10.94_397.3169m/z	pos
5038	10.94_415.3236m/z	pos
5039	10.94_444.3760m/z	pos
5040	10.94_481.3514m/z	pos
5041	10.94_607.3357m/z	neg
5042	10.95_462.3023m/z	pos

5043	10.95_467.3656n	pos
5044	10.97_118.1248n	pos
5045	10.97_241.8847m/z	pos
5046	10.97_242.2100m/z	pos
5047	10.97_406.2755n	pos
5048	10.97_431.3241m/z	pos
5049	10.97_536.5300n	pos
5050	10.97_756.5629m/z	pos
5051	10.98_116.9272m/z	neg
5052	10.98_182.9653m/z	pos
5053	10.98_253.2178m/z	pos
5054	10.98_324.2330n	pos
5055	10.98_400.3473m/z	pos
5056	10.98_523.3635m/z	pos
5057	10.98_614.3423m/z	pos
5058	10.98_764.5555m/z	neg
5059	10.98_792.5354m/z	neg
5060	10.98_794.5468m/z	neg
5061	10.98_816.5395m/z	neg
5062	10.98_837.5235n	pos
5063	10.98_882.4990m/z	pos
5064	10.98_887.5553m/z	neg
5065	10.98_894.5485m/z	neg
5066	10.98_899.4905m/z	pos
5067	10.98_901.5010m/z	pos

5068	10.99_280.2471m/z	pos
5069	10.99_295.2509n	pos
5070	10.99_339.2446n	pos
5071	10.99_380.2568m/z	pos
5072	10.99_418.7499m/z	pos
5073	10.99_421.3024m/z	pos
5074	10.99_434.3005m/z	pos
5075	10.99_449.3372m/z	pos
5076	10.99_530.3645n	pos
5077	10.99_603.5409m/z	pos
5078	10.99_741.5369n	pos
5079	10.99_774.4753m/z	pos
5080	10.99_871.5787m/z	neg
5081	10.99_874.4796m/z	pos
5082	10.99_875.4880m/z	pos
5083	10.99_887.5617m/z	pos
5084	11.01_172.1713m/z	pos
5085	11.01_189.1285m/z	pos
5086	11.01_212.1479m/z	pos
5087	11.01_454.3335n	pos
5088	11.01_520.2648m/z	neg
5089	11.01_706.5811m/z	pos
5090	11.01_776.5677m/z	pos
5091	11.01_820.5811m/z	pos
5092	11.01_824.5660m/z	pos

5093	11.01_866.5380m/z	pos
5094	11.02_198.1291m/z	pos
5095	11.02_436.3679n	pos
5096	11.02_502.7824m/z	pos
5097	11.02_504.3216m/z	pos
5098	11.02_537.2554m/z	neg
5099	11.02_552.2027m/z	neg
5100	11.02_585.2326m/z	pos
5101	11.02_587.2387m/z	pos
5102	11.02_603.3016m/z	pos
5103	11.02_613.2545m/z	pos
5104	11.02_642.1824n	pos
5105	11.02_688.3304m/z	pos
5106	11.02_947.6122m/z	neg
5107	11.03_296.6499m/z	pos
5108	11.03_496.9873m/z	pos
5109	11.03_534.7941m/z	pos
5110	11.03_550.2620m/z	pos
5111	11.03_554.3971m/z	pos
5112	11.03_576.2803n	pos
5113	11.03_578.2687m/z	pos
5114	11.03_591.2671m/z	pos
5115	11.03_596.2741n	pos
5116	11.03_754.9962m/z	pos
5117	11.03_766.4965m/z	pos

5118	11.03_948.6252n	pos
5119	11.04_152.0633m/z	neg
5120	11.04_239.0652m/z	neg
5121	11.04_706.0140n	neg
5122	11.04_711.0121m/z	neg
5123	11.04_738.0273n	neg
5124	11.04_750.0284n	neg
5125	11.04_766.0235n	neg
5126	11.04_778.0244n	neg
5127	11.04_792.0244n	neg
5128	11.04_805.0196m/z	neg
5129	11.05_134.8939m/z	neg
5130	11.05_139.3758m/z	pos
5131	11.05_139.6413m/z	pos
5132	11.05_139.9030m/z	pos
5133	11.05_151.7988m/z	neg
5134	11.05_267.6528m/z	pos
5135	11.05_273.1603n	pos
5136	11.05_275.6362m/z	pos
5137	11.05_409.2353m/z	neg
5138	11.05_478.8193m/z	neg
5139	11.05_494.8143m/z	pos
5140	11.05_507.8302m/z	pos
5141	11.05_511.3289m/z	pos
5142	11.05_515.3222m/z	pos

5143	11.05_515.8199m/z	pos
5144	11.05_522.8019m/z	pos
5145	11.05_523.8048m/z	pos
5146	11.05_527.3246m/z	pos
5147	11.05_530.3023m/z	neg
5148	11.05_538.6542m/z	neg
5149	11.05_555.5907m/z	neg
5150	11.05_570.3405m/z	neg
5151	11.05_579.3411m/z	neg
5152	11.05_582.3371m/z	neg
5153	11.05_592.2947m/z	neg
5154	11.05_612.2615m/z	neg
5155	11.05_640.2562m/z	neg
5156	11.05_688.2621m/z	neg
5157	11.05_761.0140m/z	neg
5158	11.05_762.9894m/z	pos
5159	11.05_763.4869m/z	pos
5160	11.05_768.0211n	neg
5161	11.05_769.0125m/z	neg
5162	11.05_770.9744m/z	pos
5163	11.05_787.0153m/z	neg
5164	11.05_794.0345n	neg
5165	11.05_798.0343n	neg
5166	11.05_808.0340n	neg
5167	11.05_816.0250n	neg

5168	11.05_821.0196m/z	neg
5169	11.05_825.0188m/z	neg
5170	11.05_828.0360n	neg
5171	11.05_833.0202m/z	neg
5172	11.05_836.0359n	neg
5173	11.05_847.0203m/z	neg
5174	11.05_849.0187m/z	neg
5175	11.05_860.0380n	neg
5176	11.05_864.0361n	neg
5177	11.05_975.6432m/z	neg
5178	11.05_988.6064m/z	pos
5179	11.05_991.6798m/z	pos
5180	11.06_104.1062m/z	pos
5181	11.06_607.3362m/z	neg
5182	11.06_624.2925m/z	neg
5183	11.06_819.0163m/z	neg
5184	11.06_867.0214m/z	neg
5185	11.06_869.0218m/z	neg
5186	11.06_876.0409n	neg
5187	11.06_877.0206m/z	neg
5188	11.06_890.0390n	neg
5189	11.06_901.0238m/z	neg
5190	11.08_580.3199m/z	neg
5191	11.08_598.2896m/z	neg
5192	11.08_625.3073m/z	neg

5193	11.08_676.3055m/z	neg
5194	11.09_529.2120m/z	pos
5195	11.09_790.5603m/z	neg
5196	11.10_279.2337m/z	pos
5197	11.10_426.3629m/z	pos
5198	11.11_295.2529n	pos
5199	11.11_563.2695n	pos
5200	11.11_569.3473m/z	pos
5201	11.11_585.2295m/z	pos
5202	11.11_595.2613n	pos
5203	11.11_615.2571m/z	pos
5204	11.12_583.3368m/z	neg
5205	11.13_280.6568m/z	pos
5206	11.13_288.6416m/z	pos
5207	11.13_445.3380m/z	pos
5208	11.13_476.3797m/z	pos
5209	11.13_520.7478m/z	pos
5210	11.14_128.1082m/z	pos
5211	11.14_203.1450m/z	pos
5212	11.14_204.1407m/z	pos
5213	11.14_421.2675m/z	pos
5214	11.14_774.5324m/z	neg
5215	11.15_146.9687m/z	neg
5216	11.15_175.1476m/z	pos
5217	11.15_384.3297m/z	pos

5218	11.17_124.9646m/z	pos
5219	11.17_174.1294m/z	pos
5220	11.17_234.1355m/z	pos
5221	11.17_323.2151m/z	pos
5222	11.17_348.9926m/z	pos
5223	11.17_368.2971n	pos
5224	11.17_406.2776n	pos
5225	11.17_412.3020n	pos
5226	11.17_418.7503m/z	pos
5227	11.17_430.3110n	pos
5228	11.17_451.3395m/z	pos
5229	11.17_461.3331m/z	pos
5230	11.17_464.3526n	pos
5231	11.18_189.1313m/z	pos
5232	11.18_409.7560n	pos
5233	11.18_420.7646m/z	pos
5234	11.18_454.3327n	pos
5235	11.18_481.3509m/z	neg
5236	11.18_540.9953m/z	neg
5237	11.18_603.5395m/z	pos
5238	11.18_871.5790m/z	neg
5239	11.18_876.4957m/z	pos
5240	11.18_885.5156n	pos
5241	11.18_887.5576m/z	pos
5242	11.18_894.5485m/z	neg

5243	11.19_100.9555m/z	pos
5244	11.19_118.1247n	pos
5245	11.19_262.1947m/z	pos
5246	11.19_398.3434m/z	pos
5247	11.19_406.7502m/z	pos
5248	11.19_539.3635n	pos
5249	11.19_547.1650m/z	pos
5250	11.19_969.4237m/z	pos
5251	11.19_971.4306m/z	pos
5252	11.21_126.9667m/z	pos
5253	11.21_191.0876m/z	pos
5254	11.21_209.1559m/z	pos
5255	11.21_241.8853m/z	pos
5256	11.21_309.2087m/z	pos
5257	11.21_343.2898m/z	pos
5258	11.21_379.2548m/z	pos
5259	11.21_777.5688m/z	pos
5260	11.21_880.4956m/z	pos
5261	11.21_882.4988m/z	pos
5262	11.21_898.5019n	pos
5263	11.22_235.1707m/z	pos
5264	11.22_255.2331m/z	pos
5265	11.22_371.3417n	pos
5266	11.22_491.2971m/z	pos
5267	11.22_531.3545m/z	pos

5268	11.22_536.5304n	pos
5269	11.22_605.2951m/z	pos
5270	11.22_718.5786m/z	pos
5271	11.22_893.5616m/z	pos
5272	11.23_332.3340m/z	pos
5273	11.23_413.3596n	pos
5274	11.23_479.3056n	pos
5275	11.23_532.3748m/z	pos
5276	11.23_596.3764m/z	pos
5277	11.23_668.1961n	pos
5278	11.23_705.1934m/z	pos
5279	11.23_798.6541n	pos
5280	11.24_546.2805m/z	neg
5281	11.25_265.1837m/z	pos
5282	11.25_298.2533n	pos
5283	11.25_568.3444m/z	pos
5284	11.25_959.6489m/z	pos
5285	11.26_249.6781n	pos
5286	11.26_250.1828n	pos
5287	11.26_279.6388m/z	pos
5288	11.26_438.3022m/z	pos
5289	11.26_510.8577n	pos
5290	11.26_558.3165m/z	neg
5291	11.26_564.5881m/z	neg
5292	11.26_606.3084m/z	pos

5293	11.26_614.2513m/z	pos
5294	11.26_616.2508m/z	pos
5295	11.26_617.2816m/z	pos
5296	11.26_618.3086m/z	neg
5297	11.26_621.2756n	pos
5298	11.26_624.3045m/z	neg
5299	11.26_626.2786m/z	pos
5300	11.26_626.3003m/z	neg
5301	11.26_629.3173m/z	pos
5302	11.26_638.2771m/z	neg
5303	11.26_641.2759m/z	pos
5304	11.26_650.3083m/z	neg
5305	11.26_651.3234m/z	neg
5306	11.26_680.4317m/z	pos
5307	11.26_702.3208m/z	neg
5308	11.26_832.5205m/z	neg
5309	11.27_104.1061m/z	pos
5310	11.27_280.6582m/z	pos
5311	11.27_288.1502m/z	pos
5312	11.27_418.2567m/z	pos
5313	11.27_504.3464m/z	pos
5314	11.27_520.7495m/z	pos
5315	11.27_526.3085n	pos
5316	11.27_528.3155m/z	pos
5317	11.27_528.8165m/z	pos

5318	11.27_529.8197m/z	pos
5319	11.27_533.3572m/z	pos
5320	11.27_537.3262n	pos
5321	11.27_541.3341m/z	pos
5322	11.27_541.8357m/z	pos
5323	11.27_554.3538m/z	pos
5324	11.27_625.4648m/z	pos
5325	11.27_802.0053m/z	pos
5326	11.27_809.9975m/z	pos
5327	11.28_514.3057m/z	neg
5328	11.28_516.3186m/z	neg
5329	11.28_543.3408m/z	neg
5330	11.28_594.3389m/z	neg
5331	11.28_819.0140m/z	neg
5332	11.28_821.0181m/z	neg
5333	11.28_823.0177m/z	neg
5334	11.28_833.0186m/z	neg
5335	11.28_845.0164m/z	neg
5336	11.29_268.6457m/z	pos
5337	11.29_479.3401n	pos
5338	11.29_482.3641m/z	pos
5339	11.30_276.2327m/z	pos
5340	11.31_253.1821m/z	pos
5341	11.31_515.3760m/z	pos
5342	11.33_379.2570m/z	pos

5343	11.33_616.4654m/z	pos
5344	11.34_291.2541m/z	pos
5345	11.34_297.2457m/z	pos
5346	11.34_303.2359m/z	pos
5347	11.34_467.3700m/z	pos
5348	11.34_549.3788m/z	pos
5349	11.35_256.2347m/z	pos
5350	11.35_334.2717n	pos
5351	11.35_571.3682n	pos
5352	11.36_571.3366m/z	neg
5353	11.37_294.2210n	pos
5354	11.37_396.3344m/z	pos
5355	11.37_440.3619m/z	pos
5356	11.37_481.3512m/z	neg
5357	11.37_899.4905m/z	pos
5358	11.38_179.1439m/z	pos
5359	11.38_234.1372m/z	pos
5360	11.38_241.8861m/z	pos
5361	11.38_420.7644m/z	pos
5362	11.38_822.5788m/z	pos
5363	11.40_311.1681m/z	neg
5364	11.40_884.5111m/z	pos
5365	11.41_100.9326m/z	neg
5366	11.41_161.0974m/z	pos
5367	11.41_203.1084m/z	pos

5368	11.41_262.1470m/z	pos
5369	11.41_286.2405m/z	pos
5370	11.41_664.4688m/z	pos
5371	11.41_969.4296m/z	pos
5372	11.42_100.9556m/z	pos
5373	11.42_319.2412m/z	pos
5374	11.42_319.2635m/z	neg
5375	11.42_329.2495m/z	pos
5376	11.42_364.2640n	pos
5377	11.42_421.3329m/z	pos
5378	11.42_603.5409m/z	pos
5379	11.42_620.4423m/z	pos
5380	11.42_860.5167m/z	pos
5381	11.44_365.2720m/z	pos
5382	11.44_371.3371n	pos
5383	11.44_466.2928m/z	neg
5384	11.44_468.3176m/z	pos
5385	11.44_576.4159m/z	pos
5386	11.45_330.2443n	pos
5387	11.45_434.3096n	pos
5388	11.46_253.2179m/z	pos
5389	11.46_282.6453m/z	pos
5390	11.46_357.2850m/z	pos
5391	11.46_472.3463m/z	pos
5392	11.46_487.3512n	pos

5393	11.46_508.7864m/z	pos
5394	11.46_571.3365m/z	neg
5395	11.47_462.2982m/z	neg
5396	11.47_622.3340m/z	neg
5397	11.48_409.2917m/z	pos
5398	11.48_444.3346m/z	pos
5399	11.48_464.3313m/z	pos
5400	11.48_483.2537m/z	pos
5401	11.48_492.3341m/z	pos
5402	11.48_599.2510m/z	pos
5403	11.48_609.3521m/z	neg
5404	11.48_632.2690m/z	pos
5405	11.49_484.3385m/z	pos
5406	11.49_547.3675n	pos
5407	11.50_281.6538m/z	pos
5408	11.50_436.3237n	pos
5409	11.50_470.3568m/z	pos
5410	11.50_507.3732n	pos
5411	11.51_569.3571m/z	neg
5412	11.52_542.3373m/z	neg
5413	11.53_302.2246m/z	pos
5414	11.53_305.2529m/z	pos
5415	11.53_406.2733n	pos
5416	11.53_428.3777m/z	pos
5417	11.54_177.1645m/z	pos

5418	11.54_287.2312m/z	pos
5419	11.54_385.3200m/z	pos
5420	11.54_410.7640m/z	pos
5421	11.54_452.3440m/z	pos
5422	11.54_552.4065n	pos
5423	11.54_622.4098m/z	pos
5424	11.56_234.1368m/z	pos
5425	11.56_592.9979m/z	neg
5426	11.57_213.1865m/z	pos
5427	11.57_225.1862m/z	pos
5428	11.57_298.2756m/z	pos
5429	11.57_320.2576m/z	pos
5430	11.57_434.2471n	pos
5431	11.57_573.4555m/z	pos
5432	11.57_590.3864m/z	pos
5433	11.57_598.3158n	pos
5434	11.57_598.3866m/z	pos
5435	11.57_631.4629m/z	pos
5436	11.58_223.1707m/z	pos
5437	11.60_267.1979m/z	pos
5438	11.60_876.4959m/z	pos
5439	11.61_126.9669m/z	pos
5440	11.61_253.2186m/z	pos
5441	11.61_537.5378m/z	pos
5442	11.63_559.4776m/z	pos

5443	11.63_576.5030m/z	pos
5444	11.63_816.5401m/z	neg
5445	11.64_296.1322m/z	pos
5446	11.64_339.1632m/z	pos
5447	11.64_439.3417m/z	neg
5448	11.64_454.3932m/z	pos
5449	11.64_639.4062n	pos
5450	11.65_360.2175n	pos
5451	11.65_404.3294n	pos
5452	11.66_463.3415m/z	neg
5453	11.66_570.9980m/z	neg
5454	11.66_764.5549m/z	neg
5455	11.66_848.5847m/z	neg
5456	11.67_388.1068n	pos
5457	11.67_627.5391m/z	pos
5458	11.68_148.5724m/z	pos
5459	11.68_278.1542n	pos
5460	11.68_379.0871m/z	pos
5461	11.68_419.2593m/z	pos
5462	11.68_462.3379n	pos
5463	11.68_620.3924m/z	pos
5464	11.69_278.0497m/z	pos
5465	11.71_452.3271m/z	pos
5466	11.72_267.1980m/z	pos
5467	11.72_377.2729m/z	pos

5468	11.72_511.3404m/z	neg
5469	11.72_586.4556m/z	pos
5470	11.73_285.1739m/z	pos
5471	11.73_415.3556m/z	pos
5472	11.73_542.4290m/z	pos
5473	11.73_811.0117m/z	neg
5474	11.74_808.0274n	neg
5475	11.74_825.0126m/z	neg
5476	11.75_281.6648m/z	pos
5477	11.75_289.6513m/z	pos
5478	11.75_522.7593m/z	pos
5479	11.75_523.3678n	pos
5480	11.75_623.2913n	pos
5481	11.75_643.2904m/z	pos
5482	11.76_260.2155n	pos
5483	11.76_353.1986m/z	neg
5484	11.76_444.2910n	pos
5485	11.76_558.3329m/z	neg
5486	11.76_570.3676m/z	neg
5487	11.76_585.3521m/z	neg
5488	11.76_636.3498m/z	neg
5489	11.76_777.0110m/z	neg
5490	11.77_331.2663m/z	pos
5491	11.77_366.2844n	pos
5492	11.77_410.3543m/z	pos

5493	11.77_717.5591m/z	pos
5494	11.78_387.2508m/z	neg
5495	11.79_255.2329m/z	pos
5496	11.79_364.2649n	pos
5497	11.79_531.3694m/z	pos
5498	11.79_574.3882m/z	pos
5499	11.79_776.5681m/z	pos
5500	11.80_252.1625m/z	pos
5501	11.80_319.2632m/z	neg
5502	11.80_319.2654m/z	pos
5503	11.80_382.2999m/z	pos
5504	11.80_471.3505m/z	pos
5505	11.80_533.3833m/z	pos
5506	11.80_838.6129n	pos
5507	11.81_654.4263m/z	pos
5508	11.81_893.5605m/z	pos
5509	11.83_438.3398n	pos
5510	11.83_462.3388n	pos
5511	11.83_534.3325n	pos
5512	11.83_630.4808m/z	pos
5513	11.83_871.5787m/z	neg
5514	11.84_405.3308m/z	pos
5515	11.84_424.3589m/z	pos
5516	11.84_436.3248n	pos
5517	11.84_461.3261m/z	neg

5518	11.84_467.3622m/z	pos
5519	11.84_559.4029m/z	pos
5520	11.84_586.4553m/z	pos
5521	11.84_856.5823m/z	pos
5522	11.84_865.5338m/z	pos
5523	11.84_869.5204n	pos
5524	11.84_881.4918n	pos
5525	11.84_901.4992m/z	pos
5526	11.84_909.4794n	pos
5527	11.86_292.2031n	pos
5528	11.86_361.2782m/z	pos
5529	11.86_377.3183m/z	pos
5530	11.86_379.2802m/z	pos
5531	11.86_435.3106m/z	neg
5532	11.86_542.4294m/z	pos
5533	11.86_930.5007m/z	pos
5534	11.87_241.8875m/z	pos
5535	11.87_278.1760m/z	pos
5536	11.87_492.3326m/z	pos
5537	11.87_499.3821m/z	pos
5538	11.87_604.3915m/z	pos
5539	11.87_646.4102m/z	pos
5540	11.89_412.3161n	pos
5541	11.89_422.2264m/z	pos
5542	11.89_450.3622m/z	pos

5543	11.89_580.2339m/z	neg
5544	11.89_748.5375m/z	pos
5545	11.89_753.5653m/z	pos
5546	11.89_763.5451n	pos
5547	11.89_799.6610m/z	pos
5548	11.89_846.5544m/z	pos
5549	11.90_295.2293m/z	pos
5550	11.90_366.3232m/z	pos
5551	11.90_389.2680m/z	neg
5552	11.90_391.2884m/z	pos
5553	11.90_410.3506m/z	pos
5554	11.90_480.8469m/z	pos
5555	11.90_536.4111n	pos
5556	11.90_548.2968m/z	neg
5557	11.90_720.5897m/z	pos
5558	11.90_963.6490m/z	pos
5559	11.91_631.3348m/z	pos
5560	11.91_643.2897m/z	pos
5561	11.92_628.3181m/z	neg
5562	11.93_290.1544m/z	pos
5563	11.93_311.6645m/z	pos
5564	11.93_535.3664m/z	pos
5565	11.93_535.8617m/z	pos
5566	11.93_538.3624m/z	pos
5567	11.93_558.3333m/z	neg

5568	11.93_566.5974m/z	neg
5569	11.93_585.3529m/z	neg
5570	11.93_598.3716m/z	neg
5571	11.93_619.2959m/z	pos
5572	11.93_623.2943n	pos
5573	11.93_626.3011m/z	pos
5574	11.93_652.3236m/z	neg
5575	11.93_718.2899m/z	neg
5576	11.93_768.0159n	neg
5577	11.93_770.0266n	neg
5578	11.93_779.0097m/z	neg
5579	11.93_783.0097m/z	neg
5580	11.93_790.0277n	neg
5581	11.93_837.0117m/z	neg
5582	11.93_842.0303n	neg
5583	11.93_847.0137m/z	neg
5584	11.93_863.0144m/z	neg
5585	11.93_875.0160m/z	neg
5586	11.93_879.0135m/z	neg
5587	11.94_281.6661m/z	pos
5588	11.94_289.1581m/z	pos
5589	11.94_289.6524m/z	pos
5590	11.94_522.7588m/z	pos
5591	11.94_523.3704n	pos
5592	11.94_543.8503m/z	pos

5593	11.94_551.8375m/z	pos
5594	11.94_552.3357m/z	pos
5595	11.94_555.3563m/z	pos
5596	11.94_612.3762m/z	neg
5597	11.94_620.3240m/z	neg
5598	11.94_626.3208m/z	neg
5599	11.94_708.2783m/z	neg
5600	11.94_716.2934m/z	neg
5601	11.94_813.0198m/z	pos
5602	11.94_813.5215m/z	pos
5603	11.94_843.0129m/z	neg
5604	11.94_878.0238n	neg
5605	11.94_897.0163m/z	neg
5606	11.94_914.0343n	neg
5607	11.94_930.0356n	neg
5608	11.95_104.1063m/z	pos
5609	11.96_481.3513m/z	neg
5610	11.96_505.3524m/z	neg
5611	11.96_704.3366m/z	neg
5612	11.96_794.0189n	neg
5613	11.96_848.5750m/z	neg
5614	11.96_905.0175m/z	neg
5615	11.96_907.0164m/z	neg
5616	11.98_634.4590m/z	pos
5617	11.99_324.2912m/z	pos

5618	11.99_590.4310m/z	pos
5619	12.00_304.2573n	pos
5620	12.00_480.3505n	pos
5621	12.00_718.5713m/z	pos
5622	12.00_730.5443m/z	pos
5623	12.00_816.5410m/z	neg
5624	12.02_353.2729m/z	pos
5625	12.02_397.3341m/z	pos
5626	12.02_398.3446m/z	pos
5627	12.02_462.3395n	pos
5628	12.03_644.4261n	pos
5629	12.04_130.0885m/z	pos
5630	12.04_259.1712m/z	pos
5631	12.04_366.2806n	pos
5632	12.04_414.3256m/z	pos
5633	12.04_458.3515m/z	pos
5634	12.04_504.3209n	pos
5635	12.07_390.2816n	pos
5636	12.07_636.4245m/z	pos
5637	12.07_887.5518m/z	pos
5638	12.08_382.2999m/z	pos
5639	12.08_441.3396m/z	pos
5640	12.08_465.3564m/z	neg
5641	12.08_537.5422m/z	pos
5642	12.08_764.5535m/z	neg

5643	12.08_845.5402n	pos
5644	12.09_491.3728m/z	neg
5645	12.10_124.9647m/z	pos
5646	12.10_279.2345m/z	pos
5647	12.10_329.2734m/z	pos
5648	12.10_475.3024m/z	pos
5649	12.10_603.5409m/z	pos
5650	12.11_198.1295m/z	pos
5651	12.11_298.2540n	pos
5652	12.11_361.2762m/z	pos
5653	12.11_379.2867m/z	pos
5654	12.11_419.3181m/z	pos
5655	12.11_531.3702m/z	pos
5656	12.12_319.2824m/z	pos
5657	12.12_471.3512m/z	pos
5658	12.12_538.4276n	pos
5659	12.13_537.4152m/z	neg
5660	12.14_212.1470m/z	pos
5661	12.14_549.3855n	pos
5662	12.15_776.5754m/z	pos
5663	12.15_823.6265m/z	pos
5664	12.15_824.5700m/z	pos
5665	12.16_255.2335m/z	pos
5666	12.16_490.3613m/z	pos
5667	12.16_519.4067m/z	pos

5668	12.18_317.2738m/z	pos
5669	12.19_281.2496m/z	pos
5670	12.19_421.3362m/z	pos
5671	12.19_422.3342n	pos
5672	12.19_502.4089m/z	pos
5673	12.20_289.2440m/z	pos
5674	12.22_456.3972m/z	pos
5675	12.22_893.5617m/z	pos
5676	12.23_392.2959n	pos
5677	12.23_626.4652n	pos
5678	12.23_633.4776m/z	pos
5679	12.24_340.2894m/z	pos
5680	12.24_350.3121m/z	pos
5681	12.24_410.3323m/z	pos
5682	12.26_489.3571m/z	neg
5683	12.26_582.4387n	pos
5684	12.26_808.0920m/z	pos
5685	12.27_273.1029m/z	pos
5686	12.27_908.5133m/z	pos
5687	12.28_489.2313m/z	pos
5688	12.28_510.3954m/z	pos
5689	12.28_538.3921m/z	pos
5690	12.28_538.4120n	pos
5691	12.30_494.3855n	pos
5692	12.32_236.2124n	pos

5693	12.32_278.2265m/z	pos
5694	12.32_450.3582n	pos
5695	12.32_451.3652m/z	pos
5696	12.32_484.2922n	pos
5697	12.32_608.3907m/z	pos
5698	12.34_195.1239m/z	pos
5699	12.34_318.2900n	pos
5700	12.34_376.3195m/z	pos
5701	12.34_406.3324n	pos
5702	12.34_650.4439m/z	pos
5703	12.35_175.1478m/z	pos
5704	12.35_179.1326n	pos
5705	12.35_299.2611m/z	pos
5706	12.35_362.3060n	pos
5707	12.35_421.3756m/z	pos
5708	12.35_458.3626n	pos
5709	12.36_391.2894m/z	pos
5710	12.36_428.3403m/z	pos
5711	12.36_456.3548n	pos
5712	12.38_314.2725m/z	pos
5713	12.38_552.4111n	pos
5714	12.39_352.3260m/z	pos
5715	12.39_375.2925m/z	pos
5716	12.39_389.3438m/z	pos
5717	12.39_532.3785n	pos

5718	12.40_419.3183m/z	pos
5719	12.41_518.4000n	pos
5720	12.42_466.3617n	pos
5721	12.43_799.6501m/z	pos
5722	12.45_286.2171n	pos
5723	12.45_303.2347m/z	pos
5724	12.45_480.3709m/z	pos
5725	12.45_622.4098m/z	pos
5726	12.47_275.2603m/z	pos
5727	12.47_299.2638m/z	pos
5728	12.47_538.3918m/z	pos
5729	12.48_410.3349m/z	pos
5730	12.49_357.2790n	pos
5731	12.49_650.4424m/z	pos
5732	12.52_497.3418m/z	pos
5733	12.52_579.4074m/z	pos
5734	12.54_278.2270n	pos
5735	12.54_412.3252n	pos
5736	12.54_690.4384m/z	pos
5737	12.55_489.3572m/z	neg
5738	12.56_237.1864m/z	pos
5739	12.56_268.2679m/z	pos
5740	12.56_452.4003n	pos
5741	12.58_295.2299m/z	pos
5742	12.60_433.3370m/z	pos

5743	12.60_487.4007m/z	pos
5744	12.61_198.1293m/z	pos
5745	12.61_342.7826m/z	pos
5746	12.61_352.3242m/z	pos
5747	12.61_426.3282m/z	pos
5748	12.62_283.2716m/z	pos
5749	12.62_342.2813m/z	pos
5750	12.62_356.2966m/z	pos
5751	12.62_762.5418m/z	neg
5752	12.64_555.4355m/z	pos
5753	12.65_441.3630m/z	pos
5754	12.65_509.3840m/z	neg
5755	12.65_700.5561n	pos
5756	12.66_230.2210m/z	pos
5757	12.66_452.3550n	pos
5758	12.66_454.3656m/z	pos
5759	12.66_728.5873n	pos
5760	12.66_848.5608m/z	pos
5761	12.68_357.2804n	pos
5762	12.68_496.3904m/z	pos
5763	12.69_410.3327m/z	pos
5764	12.69_808.0923m/z	pos
5765	12.70_477.3894m/z	pos
5766	12.70_491.3729m/z	neg
5767	12.70_536.4116n	pos

5768	12.72_289.2795m/z	pos
5769	12.72_462.1269n	pos
5770	12.72_464.1352m/z	pos
5771	12.73_640.4814n	pos
5772	12.74_562.4270m/z	pos
5773	12.75_451.3754m/z	pos
5774	12.76_899.4960m/z	pos
5775	12.77_567.4684m/z	pos
5776	12.79_294.2837m/z	pos
5777	12.80_748.5404m/z	pos
5778	12.81_281.2502m/z	pos
5779	12.81_552.4099m/z	pos
5780	12.81_596.4546n	pos
5781	12.83_464.3745n	pos
5782	12.83_480.9212m/z	pos
5783	12.84_279.2393m/z	pos
5784	12.85_133.0875m/z	pos
5785	12.85_239.1503m/z	pos
5786	12.85_283.2458m/z	neg
5787	12.85_462.3612m/z	pos
5788	12.85_552.4275n	pos
5789	12.87_490.3680n	pos
5790	12.87_799.6504m/z	pos
5791	12.88_508.4014n	pos
5792	12.91_219.2124m/z	pos

5793	12.91_254.2261n	pos
5794	12.91_352.1833m/z	pos
5795	12.91_427.3221m/z	pos
5796	12.92_309.2445m/z	pos
5797	12.92_495.4042m/z	pos
5798	12.92_499.3826m/z	pos
5799	12.93_420.3483n	pos
5800	12.95_151.0974m/z	pos
5801	12.95_332.2948n	pos
5802	12.95_376.3215n	pos
5803	12.95_403.3248m/z	pos
5804	12.97_313.2767m/z	pos
5805	12.97_383.3364m/z	pos
5806	12.99_677.5602m/z	pos
5807	12.99_860.5155m/z	pos
5808	13.00_499.3837m/z	pos
5809	13.00_517.3916m/z	pos
5810	13.00_536.4133n	pos
5811	13.00_808.0925m/z	pos
5812	13.00_896.4572m/z	pos
5813	13.03_495.4068m/z	pos
5814	13.03_859.5981m/z	pos
5815	13.03_909.5233n	pos
5816	13.05_304.2418n	pos
5817	13.05_533.3854m/z	pos

5818	13.06_551.4008n	pos
5819	13.07_645.5025m/z	pos
5820	13.08_656.4331n	pos
5821	13.09_462.3623m/z	pos
5822	13.09_485.3942m/z	pos
5823	13.09_503.4111m/z	pos
5824	13.12_170.1552m/z	pos
5825	13.12_247.2435m/z	pos
5826	13.12_265.2543m/z	pos
5827	13.12_281.2742n	pos
5828	13.12_281.4137m/z	pos
5829	13.13_293.2509m/z	pos
5830	13.13_918.5826m/z	pos
5831	13.14_403.3596m/z	pos
5832	13.14_490.3678n	pos
5833	13.14_575.4707m/z	pos
5834	13.14_636.3032n	pos
5835	13.14_681.2968m/z	neg
5836	13.14_695.3695m/z	pos
5837	13.16_286.2345m/z	pos
5838	13.16_512.4098n	pos
5839	13.17_269.2286m/z	pos
5840	13.17_279.2345m/z	pos
5841	13.17_876.6948m/z	pos
5842	13.18_100.9334m/z	neg

5843	13.18_239.2408m/z	pos
5844	13.20_134.8941m/z	neg
5845	13.20_138.1428n	pos
5846	13.20_175.1491m/z	pos
5847	13.20_182.1329n	pos
5848	13.20_184.1478n	pos
5849	13.20_188.1438n	pos
5850	13.20_196.1483n	pos
5851	13.20_210.1632n	pos
5852	13.20_225.1864m/z	pos
5853	13.20_245.2285m/z	pos
5854	13.20_280.2427n	pos
5855	13.20_347.2195m/z	neg
5856	13.20_560.4845n	pos
5857	13.20_581.4543m/z	neg
5858	13.20_720.5908m/z	pos
5859	13.20_742.5783m/z	pos
5860	13.20_756.2276m/z	pos
5861	13.20_773.5621n	pos
5862	13.20_778.1412m/z	pos
5863	13.20_778.5509m/z	pos
5864	13.20_780.1515m/z	pos
5865	13.20_808.0929m/z	pos
5866	13.20_819.5496n	pos
5867	13.20_820.6140m/z	pos

5868	13.20_825.5926n	pos
5869	13.20_832.5888m/z	pos
5870	13.20_844.6315m/z	pos
5871	13.21_151.1108m/z	pos
5872	13.21_329.2476m/z	neg
5873	13.21_364.2098m/z	neg
5874	13.21_536.4124n	pos
5875	13.21_578.4283m/z	pos
5876	13.21_718.5773m/z	pos
5877	13.24_455.3758m/z	pos
5878	13.24_896.4542m/z	pos
5879	13.27_716.5516m/z	pos
5880	13.27_795.0803m/z	pos
5881	13.28_340.3603m/z	pos
5882	13.28_770.0696m/z	pos
5883	13.30_447.3493m/z	pos
5884	13.32_494.3964m/z	pos
5885	13.33_533.3888m/z	pos
5886	13.33_608.4794m/z	pos
5887	13.33_633.4660m/z	pos
5888	13.36_566.4433n	pos
5889	13.36_781.0582m/z	pos
5890	13.37_353.3329n	pos
5891	13.38_522.4156n	pos
5892	13.38_575.4722m/z	pos

5893	13.40_223.1712m/z	pos
5894	13.41_478.3896n	pos
5895	13.42_417.3395m/z	pos
5896	13.44_239.1505m/z	pos
5897	13.44_434.3634n	pos
5898	13.45_195.1240m/z	pos
5899	13.45_390.3369n	pos
5900	13.45_487.3481m/z	pos
5901	13.46_364.3446m/z	pos
5902	13.46_369.3023m/z	pos
5903	13.46_874.4930m/z	neg
5904	13.48_537.4156m/z	neg
5905	13.49_415.0397m/z	pos
5906	13.49_446.3421n	pos
5907	13.49_484.4047m/z	pos
5908	13.49_537.4222m/z	pos
5909	13.51_223.1711m/z	pos
5910	13.51_403.3600m/z	pos
5911	13.52_519.4072m/z	pos
5912	13.55_279.2344m/z	pos
5913	13.57_346.3130n	pos
5914	13.58_857.5139n	pos
5915	13.61_377.2720m/z	neg
5916	13.62_261.2236m/z	neg
5917	13.64_537.4159m/z	neg

5918	13.65_379.2872m/z	pos
5919	13.65_397.3408m/z	pos
5920	13.65_432.3627n	pos
5921	13.65_468.3844n	pos
5922	13.65_538.4270n	pos
5923	13.66_503.4133m/z	pos
5924	13.66_535.4023m/z	pos
5925	13.67_720.5900m/z	pos
5926	13.68_884.5179m/z	pos
5927	13.69_770.0696m/z	pos
5928	13.69_889.5352m/z	pos
5929	13.69_948.4769m/z	pos
5930	13.71_590.4604n	pos
5931	13.71_904.4848m/z	pos
5932	13.72_515.4348m/z	pos
5933	13.72_519.4072m/z	pos
5934	13.72_781.0582m/z	pos
5935	13.74_635.4900m/z	pos
5936	13.76_281.2500m/z	pos
5937	13.76_317.2755m/z	pos
5938	13.76_565.4460m/z	pos
5939	13.78_475.3807m/z	pos
5940	13.78_872.5600m/z	pos
5941	13.78_876.6920m/z	pos
5942	13.79_256.2466n	pos

5943	13.79_395.2256m/z	pos
5944	13.79_778.1413m/z	pos
5945	13.79_778.5478m/z	pos
5946	13.79_790.5887m/z	pos
5947	13.79_799.5777n	pos
5948	13.79_804.5577m/z	pos
5949	13.79_823.6518n	pos
5950	13.80_825.5705n	pos
5951	13.82_878.5925m/z	neg
5952	13.83_279.2339m/z	pos
5953	13.83_795.0740m/z	pos
5954	13.84_554.1802m/z	pos
5955	13.85_433.3687m/z	pos
5956	13.85_468.3851n	pos
5957	13.85_566.4607n	pos
5958	13.87_535.4045m/z	pos
5959	13.88_523.4402m/z	pos
5960	13.88_577.4885m/z	pos
5961	13.89_383.3421n	pos
5962	13.89_483.3525m/z	pos
5963	13.90_338.3446m/z	pos
5964	13.95_317.2761m/z	pos
5965	13.95_567.4650m/z	pos
5966	13.97_281.2506m/z	pos
5967	13.97_519.4074m/z	pos

5968	13.97_589.4526m/z	pos
5969	13.97_591.4705m/z	pos
5970	13.97_592.4755n	pos
5971	13.98_100.9334m/z	neg
5972	13.98_227.2042m/z	pos
5973	13.98_842.6075m/z	pos
5974	13.99_283.2598m/z	neg
5975	14.00_247.2466m/z	pos
5976	14.00_282.2590n	pos
5977	14.00_778.1464m/z	pos
5978	14.00_780.5579m/z	pos
5979	14.00_789.5791n	pos
5980	14.00_833.6083n	pos
5981	14.01_523.4425m/z	pos
5982	14.01_717.5911m/z	pos
5983	14.01_831.5833n	pos
5984	14.01_840.6218m/z	pos
5985	14.02_473.3609m/z	neg
5986	14.06_284.2969m/z	pos
5987	14.08_577.4879m/z	pos
5988	14.09_475.3800m/z	pos
5989	14.09_539.4372m/z	pos
5990	14.10_740.5510m/z	pos
5991	14.13_696.5400m/z	pos
5992	14.13_805.5681n	pos

5993	14.15_519.4072m/z	pos
5994	14.15_610.5102m/z	pos
5995	14.15_794.0667m/z	pos
5996	14.16_523.4394m/z	pos
5997	14.16_592.4747n	pos
5998	14.17_652.5029m/z	pos
5999	14.18_765.5526n	pos
6000	14.19_608.4796m/z	pos
6001	14.19_804.0768m/z	pos
6002	14.21_492.4080m/z	pos
6003	14.21_799.6604m/z	pos
6004	14.22_590.4586m/z	pos
6005	14.26_411.2803m/z	pos
6006	14.26_519.4084m/z	pos
6007	14.26_524.4524n	pos
6008	14.28_867.5517m/z	neg
6009	14.29_523.4399m/z	pos
6010	14.29_578.4948m/z	pos
6011	14.29_711.5206m/z	pos
6012	14.33_391.3600m/z	pos
6013	14.33_784.5909m/z	pos
6014	14.34_695.5161m/z	pos
6015	14.36_667.4865m/z	pos
6016	14.37_358.3122n	pos
6017	14.38_573.4601m/z	pos

6018	14.39_928.4837m/z	pos
6019	14.40_517.3937m/z	pos
6020	14.43_284.2980m/z	pos
6021	14.44_360.2916m/z	pos
6022	14.44_563.4394m/z	pos
6023	14.44_623.4591m/z	pos
6024	14.46_523.4396m/z	pos
6025	14.49_748.5568m/z	pos
6026	14.50_336.3289m/z	pos
6027	14.52_519.4060m/z	pos
6028	14.52_948.4769m/z	pos
6029	14.54_103.0538m/z	pos
6030	14.54_415.0385m/z	pos
6031	14.55_848.6521m/z	pos
6032	14.56_129.1285m/z	pos
6033	14.56_748.5416m/z	pos
6034	14.57_559.4765m/z	pos
6035	14.58_107.0508m/z	pos
6036	14.60_407.3937m/z	pos
6037	14.61_748.5339m/z	pos
6038	14.63_932.5179m/z	pos
6039	14.65_594.4912n	pos
6040	14.68_508.4626n	pos
6041	14.70_596.4224m/z	pos
6042	14.73_123.7126m/z	pos

6043	14.73_546.4314n	pos
6044	14.73_876.6867m/z	pos
6045	14.74_569.4179m/z	pos
6046	14.74_616.5111n	pos
6047	14.75_279.2365m/z	pos
6048	14.76_319.2649m/z	pos
6049	14.76_336.2918m/z	pos
6050	14.76_591.4682m/z	pos
6051	14.79_435.3516m/z	pos
6052	14.79_576.4799n	pos
6053	14.79_594.4907n	pos
6054	14.79_594.4909n	pos
6055	14.80_405.3792m/z	pos
6056	14.80_573.4571m/z	pos
6057	14.80_653.5569m/z	pos
6058	14.81_429.3759m/z	pos
6059	14.81_521.4236m/z	pos
6060	14.81_592.4756n	pos
6061	14.81_908.5129m/z	pos
6062	14.82_344.0089m/z	pos
6063	14.82_575.4711m/z	pos
6064	14.84_368.3466n	pos
6065	14.84_699.5438m/z	pos
6066	14.85_522.4328n	pos
6067	14.91_435.3540m/z	pos

6068	14.91_577.4872m/z	pos
6069	14.93_593.4796m/z	neg
6070	14.96_128.0634m/z	pos
6071	14.96_279.2351m/z	pos
6072	14.96_361.0216m/z	pos
6073	14.98_574.4630n	pos
6074	15.00_285.2807m/z	pos
6075	15.02_782.5745m/z	pos
6076	15.03_191.1807m/z	pos
6077	15.04_123.7139m/z	pos
6078	15.04_628.1990m/z	pos
6079	15.07_294.2575n	pos
6080	15.22_153.0460m/z	pos
6081	15.24_437.3660m/z	pos
6082	15.25_138.0670m/z	pos
6083	15.27_636.5629m/z	pos
6084	15.29_547.4442m/z	pos
6085	15.29_579.5034m/z	pos
6086	15.33_261.1501m/z	pos
6087	15.33_584.4325m/z	pos
6088	15.33_803.5478m/z	pos
6089	15.35_138.0671m/z	pos
6090	15.37_312.3288m/z	pos
6091	15.37_624.4523m/z	pos
6092	15.39_595.4987m/z	pos

6093	15.39_904.4817m/z	pos
6094	15.40_121.0658m/z	pos
6095	15.40_153.0461m/z	pos
6096	15.41_564.4018m/z	pos
6097	15.41_580.5049m/z	pos
6098	15.43_476.3680m/z	pos
6099	15.43_656.4369m/z	pos
6100	15.45_584.4272m/z	pos
6101	15.46_320.3115n	pos
6102	15.46_574.4655n	pos
6103	15.46_575.4712m/z	pos
6104	15.46_675.6811m/z	pos
6105	15.48_115.0509m/z	pos
6106	15.48_566.4162m/z	pos
6107	15.48_587.5543m/z	pos
6108	15.49_473.2825m/z	neg
6109	15.49_617.4810m/z	pos
6110	15.49_850.6353m/z	pos
6111	15.51_474.2936n	pos
6112	15.51_548.3869m/z	pos
6113	15.52_565.5726m/z	pos
6114	15.53_107.0508m/z	pos
6115	15.53_619.4966m/z	pos
6116	15.55_191.1806m/z	pos
6117	15.60_579.5037m/z	pos

6118	15.60_597.5101m/z	pos
6119	15.64_191.1813m/z	pos
6120	15.75_341.0200m/z	pos
6121	15.75_776.2379m/z	pos
6122	15.77_326.9687m/z	pos
6123	15.85_429.3759m/z	pos
6124	15.86_201.1649m/z	pos
6125	15.87_107.0499m/z	pos
6126	15.87_131.9029m/z	pos
6127	15.88_283.0348m/z	pos
6128	15.88_355.0727m/z	pos
6129	15.89_267.0005m/z	pos
6130	15.91_117.0709m/z	pos
6131	15.91_119.0860m/z	pos
6132	15.91_128.0626m/z	pos
6133	15.91_203.1799m/z	pos
6134	15.93_207.0336m/z	pos
6135	15.93_371.1039m/z	pos
6136	15.93_430.8881m/z	pos
6137	15.97_191.1807m/z	pos
6138	15.98_125.9688m/z	pos
6139	2.00_135.6647m/z	pos
6140	2.00_179.5151m/z	neg
6141	2.00_238.0245m/z	neg
6142	2.00_249.0604n	neg

6143	2.00_266.0508n	neg
6144	2.00_282.0277n	neg
6145	2.00_296.0356m/z	neg
6146	2.00_300.0060m/z	neg
6147	2.00_305.9380m/z	neg
6148	2.00_307.5869m/z	pos
6149	2.00_332.9572m/z	neg
6150	2.00_334.9552m/z	neg
6151	2.00_340.9074m/z	neg
6152	2.00_348.9987m/z	pos
6153	2.00_359.0287m/z	neg
6154	2.00_359.9768m/z	neg
6155	2.00_367.9267m/z	neg
6156	2.00_369.9234m/z	neg
6157	2.00_378.1145n	pos
6158	2.00_441.1167n	pos
6159	2.00_451.0364m/z	neg
6160	2.00_453.0536m/z	pos
6161	2.00_461.0635m/z	neg
6162	2.00_468.0981m/z	neg
6163	2.00_476.0841n	pos
6164	2.00_478.0549m/z	neg
6165	2.00_483.0576m/z	neg
6166	2.00_483.0999m/z	pos
6167	2.00_484.1150m/z	pos

6168	2.00_494.0990m/z	neg
6169	2.00_527.0888m/z	pos
6170	2.00_786.2586m/z	pos
6171	2.01_202.0480m/z	neg
6172	2.01_235.1211m/z	pos
6173	2.01_240.0244m/z	neg
6174	2.01_264.0240m/z	neg
6175	2.01_266.0343m/z	pos
6176	2.01_280.9977n	neg
6177	2.01_316.9893m/z	neg
6178	2.01_324.9892m/z	neg
6179	2.01_347.9839m/z	neg
6180	2.01_364.9775m/z	neg
6181	2.03_158.0046m/z	pos
6182	2.03_265.1544n	pos
6183	2.04_115.0764m/z	pos
6184	2.04_154.9673m/z	pos
6185	2.04_260.0289m/z	neg
6186	2.04_385.1306m/z	pos
6187	2.04_440.2301m/z	pos
6188	2.05_104.0089m/z	pos
6189	2.05_124.9913m/z	pos
6190	2.05_180.0330n	pos
6191	2.05_189.0085m/z	pos
6192	2.05_259.0823m/z	pos

6193	2.05_274.0984m/z	pos
6194	2.05_351.1025m/z	pos
6195	2.06_171.0266m/z	neg
6196	2.06_202.9643m/z	neg
6197	2.06_232.9975m/z	neg
6198	2.06_256.1156m/z	neg
6199	2.06_318.0856m/z	neg
6200	2.07_200.0012m/z	pos
6201	2.08_227.9042m/z	neg
6202	2.08_229.0689m/z	neg
6203	2.08_229.9013m/z	neg
6204	2.09_177.1063m/z	pos
6205	2.12_111.0438m/z	pos
6206	2.12_220.0800m/z	pos
6207	2.12_341.1522n	pos
6208	2.12_399.3487m/z	pos
6209	2.12_417.2445n	pos
6210	2.12_431.2323n	pos
6211	2.12_549.3462m/z	pos
6212	2.12_724.2564m/z	pos
6213	2.13_131.7025m/z	pos
6214	2.13_294.2285m/z	pos
6215	2.13_319.1350m/z	pos
6216	2.13_458.1051m/z	pos
6217	2.13_459.0955n	pos

6218	2.13_502.4126m/z	pos
6219	2.14_188.0455m/z	neg
6220	2.14_198.0741m/z	neg
6221	2.14_215.0644m/z	neg
6222	2.14_230.0115m/z	neg
6223	2.14_250.0193m/z	neg
6224	2.14_252.0137m/z	neg
6225	2.14_254.9513m/z	neg
6226	2.14_265.9892m/z	neg
6227	2.14_267.0048m/z	neg
6228	2.14_281.9725m/z	neg
6229	2.14_283.1631m/z	neg
6230	2.14_293.0067m/z	neg
6231	2.14_295.0345n	neg
6232	2.14_361.1053m/z	neg
6233	2.14_430.2214m/z	neg
6234	2.14_462.2380m/z	neg
6235	2.15_145.0357m/z	pos
6236	2.15_226.8235m/z	neg
6237	2.15_256.1161m/z	neg
6238	2.15_332.1528n	neg
6239	2.15_379.1157n	neg
6240	2.15_446.1942m/z	neg
6241	2.21_151.0623m/z	pos
6242	2.22_136.0391m/z	pos

6243	2.22_185.8751m/z	neg
6244	2.24_164.9289m/z	neg
6245	2.24_252.9937m/z	neg
6246	2.26_200.0027m/z	pos
6247	2.27_130.0083m/z	pos
6248	2.27_146.0163n	pos
6249	2.27_181.9898m/z	pos
6250	2.27_189.0096m/z	pos
6251	2.27_221.0894m/z	pos
6252	2.27_262.0144m/z	pos
6253	2.28_158.9751m/z	neg
6254	2.28_171.0254m/z	neg
6255	2.28_192.9358m/z	neg
6256	2.28_221.9821n	neg
6257	2.28_224.9658m/z	neg
6258	2.28_227.9044m/z	neg
6259	2.28_229.9016m/z	neg
6260	2.28_232.9977m/z	neg
6261	2.28_235.9986m/z	neg
6262	2.28_249.9881m/z	neg
6263	2.28_261.0054m/z	neg
6264	2.28_285.9854m/z	neg
6265	2.28_308.0292m/z	neg
6266	2.29_363.1043m/z	neg
6267	2.33_235.1430n	pos

6268	2.35_144.0259n	pos
6269	2.40_166.0888m/z	pos
6270	2.45_196.1545m/z	pos
6271	2.47_145.0340m/z	pos
6272	2.48_162.0626m/z	pos
6273	2.50_105.9343m/z	pos
6274	2.52_179.1068m/z	pos
6275	2.56_252.1393n	pos
6276	2.59_308.2835m/z	pos
6277	2.64_212.0012m/z	pos
6278	2.65_244.9972m/z	neg
6279	2.72_229.1460m/z	pos
6280	2.85_342.2123m/z	pos
6281	2.91_104.0079m/z	pos
6282	2.95_308.2830m/z	pos
6283	2.98_119.0735m/z	pos
6284	2.98_520.2037m/z	pos
6285	2.99_511.2036n	pos
6286	2.99_519.1955n	pos
6287	2.99_536.4008m/z	pos
6288	2.99_786.2574m/z	pos
6289	3.00_102.0472n	pos
6290	3.00_165.5822m/z	pos
6291	3.00_421.0638m/z	pos
6292	3.01_233.0659n	neg

6293	3.01_249.0495m/z	neg
6294	3.01_265.0032n	neg
6295	3.01_280.0406m/z	neg
6296	3.01_285.9968m/z	neg
6297	3.01_288.9355m/z	neg
6298	3.01_289.9428m/z	neg
6299	3.01_294.0289m/z	neg
6300	3.01_297.0307m/z	neg
6301	3.01_300.9895m/z	neg
6302	3.01_315.9571m/z	neg
6303	3.01_316.9622m/z	neg
6304	3.01_328.0123n	neg
6305	3.01_347.0262n	neg
6306	3.01_429.0739m/z	neg
6307	3.02_266.0134m/z	pos
6308	3.02_269.0346m/z	neg
6309	3.02_285.0287n	neg
6310	3.02_419.0465m/z	neg
6311	3.02_445.1173m/z	neg
6312	3.02_462.1095m/z	neg
6313	3.05_162.9823m/z	neg
6314	3.05_308.2841m/z	pos
6315	3.31_105.9349m/z	pos
6316	3.36_142.0356m/z	pos
6317	3.54_105.9338m/z	pos

6318	3.74_105.9365m/z	pos
6319	3.83_253.1467m/z	pos
6320	3.97_219.0542m/z	pos
6321	4.00_142.0883m/z	pos
6322	4.00_145.1223m/z	pos
6323	4.07_125.0982m/z	pos
6324	4.08_142.0873m/z	pos
6325	4.08_176.0750m/z	pos
6326	4.09_117.0547m/z	neg
6327	4.09_202.0327m/z	neg
6328	4.09_247.0128m/z	neg
6329	4.09_264.0033m/z	neg
6330	4.09_280.9941m/z	neg
6331	4.10_172.9903m/z	neg
6332	4.10_216.9801m/z	neg
6333	4.16_171.0114m/z	neg
6334	4.17_124.0498m/z	pos
6335	4.18_116.0541m/z	neg
6336	4.18_187.4973m/z	pos
6337	4.18_202.4592m/z	neg
6338	4.18_226.0713n	neg
6339	4.18_257.9685m/z	neg
6340	4.18_263.0397m/z	neg
6341	4.18_278.0185n	neg
6342	4.18_288.0592m/z	neg

6343	4.18_303.0024m/z	neg
6344	4.18_305.0353n	neg
6345	4.18_338.0700n	neg
6346	4.18_338.9845m/z	neg
6347	4.18_340.0000m/z	neg
6348	4.18_341.9979m/z	neg
6349	4.18_355.9732m/z	neg
6350	4.18_366.0017m/z	neg
6351	4.18_368.0295n	neg
6352	4.18_368.9711m/z	neg
6353	4.18_399.0637m/z	neg
6354	4.18_402.0095n	neg
6355	4.18_432.8859m/z	pos
6356	4.18_446.1340n	neg
6357	4.18_514.1307m/z	neg
6358	4.18_524.0870m/z	neg
6359	4.18_704.1541m/z	pos
6360	4.18_786.2560m/z	pos
6361	4.19_114.0474n	pos
6362	4.19_126.0464n	pos
6363	4.19_145.6176m/z	pos
6364	4.19_230.9937n	pos
6365	4.19_283.0523n	pos
6366	4.19_289.0518m/z	pos
6367	4.19_290.0788n	pos

6368	4.19_294.9958m/z	pos
6369	4.19_394.0583n	pos
6370	4.19_462.1016n	pos
6371	4.19_522.1176n	pos
6372	4.19_532.0956n	pos
6373	4.19_575.4098m/z	pos
6374	4.19_598.1499n	pos
6375	4.19_636.2295n	pos
6376	4.19_885.2903m/z	pos
6377	4.20_124.8908m/z	neg
6378	4.20_162.9825m/z	neg
6379	4.21_204.4722m/z	pos
6380	4.22_204.1394m/z	pos
6381	4.22_238.1441n	pos
6382	4.22_284.2085m/z	pos
6383	4.22_471.2247m/z	pos
6384	4.24_136.0512n	neg
6385	4.24_181.0063m/z	neg
6386	4.24_183.0030m/z	neg
6387	4.24_187.8606m/z	neg
6388	4.24_200.0301m/z	neg
6389	4.24_267.0269n	neg
6390	4.24_268.0168m/z	neg
6391	4.24_285.0064m/z	neg
6392	4.24_316.9678m/z	pos

6393	4.24_318.9775n	neg
6394	4.24_322.8963m/z	neg
6395	4.24_329.9876m/z	neg
6396	4.24_347.0076n	neg
6397	4.24_350.9233m/z	neg
6398	4.24_419.0537m/z	neg
6399	4.24_421.0508m/z	neg
6400	4.24_486.9680m/z	neg
6401	4.24_492.9814m/z	pos
6402	4.24_496.9961m/z	neg
6403	4.24_518.9972m/z	neg
6404	4.24_634.0593m/z	neg
6405	4.24_640.0751m/z	neg
6406	4.24_652.0300m/z	neg
6407	4.24_666.0762m/z	neg
6408	4.24_668.0738m/z	neg
6409	4.24_691.0090m/z	pos
6410	4.24_852.0901m/z	pos
6411	4.24_874.0675n	pos
6412	4.24_891.0440m/z	pos
6413	4.24_894.0597n	pos
6414	4.25_102.0459m/z	pos
6415	4.25_117.0576m/z	pos
6416	4.25_117.7091m/z	pos
6417	4.25_136.0087n	pos

6418	4.25_137.0158m/z	neg
6419	4.25_138.0064n	pos
6420	4.25_140.0035n	pos
6421	4.25_143.0073m/z	pos
6422	4.25_153.5758m/z	pos
6423	4.25_182.0144n	pos
6424	4.25_184.0121n	pos
6425	4.25_197.4248m/z	neg
6426	4.25_199.4378m/z	pos
6427	4.25_222.0304m/z	pos
6428	4.25_224.0291m/z	pos
6429	4.25_236.9953n	neg
6430	4.25_255.9907m/z	neg
6431	4.25_257.9881m/z	neg
6432	4.25_260.0305m/z	pos
6433	4.25_284.0024m/z	pos
6434	4.25_288.0025m/z	neg
6435	4.25_289.9478m/z	pos
6436	4.25_298.9825m/z	neg
6437	4.25_300.9831m/z	neg
6438	4.25_300.9913m/z	pos
6439	4.25_302.9884m/z	pos
6440	4.25_315.9996m/z	neg
6441	4.25_316.9745n	neg
6442	4.25_319.9633m/z	neg

6443	4.25_333.0214n	neg
6444	4.25_334.9409n	neg
6445	4.25_334.9508m/z	neg
6446	4.25_336.9476m/z	neg
6447	4.25_352.9205m/z	neg
6448	4.25_354.9183m/z	neg
6449	4.25_358.8714m/z	neg
6450	4.25_360.8702m/z	neg
6451	4.25_361.9744n	neg
6452	4.25_362.8671m/z	neg
6453	4.25_377.9415m/z	neg
6454	4.25_436.0345n	neg
6455	4.25_438.0321n	neg
6456	4.25_452.0030n	pos
6457	4.25_465.0464m/z	neg
6458	4.25_468.0476n	neg
6459	4.25_470.9948m/z	neg
6460	4.25_477.0436n	pos
6461	4.25_480.1996m/z	pos
6462	4.25_488.9654m/z	neg
6463	4.25_488.9862m/z	pos
6464	4.25_490.9627m/z	neg
6465	4.25_504.0307m/z	neg
6466	4.25_506.0284m/z	neg
6467	4.25_515.0078n	neg

6468	4.25_515.9851m/z	neg
6469	4.25_526.0531m/z	pos
6470	4.25_528.0482m/z	pos
6471	4.25_530.0312m/z	neg
6472	4.25_532.0279m/z	neg
6473	4.25_570.3617m/z	pos
6474	4.25_622.0912m/z	pos
6475	4.25_635.0719n	pos
6476	4.25_636.0569m/z	neg
6477	4.25_637.0706n	pos
6478	4.25_646.0723m/z	pos
6479	4.25_652.0513m/z	pos
6480	4.25_654.0485m/z	pos
6481	4.25_661.0796n	pos
6482	4.25_685.0636m/z	neg
6483	4.25_821.1326m/z	pos
6484	4.25_823.1297m/z	pos
6485	4.25_865.0921m/z	pos
6486	4.25_867.0948m/z	pos
6487	4.26_155.5666m/z	pos
6488	4.29_134.0973m/z	pos
6489	4.29_512.7932m/z	pos
6490	4.31_208.1334n	pos
6491	4.31_253.1897n	pos
6492	4.32_409.2513m/z	pos

6493	4.32_409.58<br>13m/z	pos
6494	4.32_464.76<br>57m/z	pos
6495	4.32_465.26<br>84m/z	pos
6496	4.39_300.01<br>73m/z	neg
6497	4.39_338.13<br>90m/z	pos
6498	4.40_199.98<br>91m/z	pos
6499	4.41_243.99<br>75m/z	pos
6500	4.41_437.72<br>59m/z	pos
6501	4.42_104.00<br>87m/z	pos
6502	4.42_282.17<br>01n	pos
6503	4.42_375.70<br>25m/z	pos
6504	4.42_500.59<br>75m/z	pos
6505	4.43_218.99<br>57m/z	neg
6506	4.44_304.91<br>30m/z	neg
6507	4.44_328.23<br>41m/z	pos
6508	4.44_333.00<br>61m/z	neg
6509	4.46_296.14<br>73n	pos
6510	4.46_342.21<br>34m/z	pos
6511	4.46_448.23<br>84m/z	pos
6512	4.47_156.03<br>91m/z	pos
6513	4.47_166.08<br>83m/z	pos
6514	4.47_203.00<br>13m/z	neg
6515	4.47_623.12<br>77m/z	pos
6516	4.48_138.05<br>52m/z	neg
6517	4.49_129.05<br>49m/z	neg

6518	4.49_187.0094m/z	neg
6519	4.49_276.0029m/z	neg
6520	4.49_289.8963m/z	neg
6521	4.50_118.0658m/z	pos
6522	4.50_246.1392n	pos
6523	4.50_254.0009m/z	pos
6524	4.50_455.0445m/z	pos
6525	4.50_460.2144m/z	pos
6526	4.50_635.3442m/z	pos
6527	4.51_141.0582n	neg
6528	4.51_248.9915m/z	neg
6529	4.51_281.9473m/z	neg
6530	4.51_291.8930m/z	neg
6531	4.51_292.9927m/z	neg
6532	4.51_308.9658m/z	neg
6533	4.51_343.0696m/z	neg
6534	4.52_137.0236m/z	neg
6535	4.52_270.0037n	pos
6536	4.52_296.9792m/z	neg
6537	4.52_379.7094m/z	pos
6538	4.52_475.0148m/z	pos
6539	4.52_475.5125m/z	pos
6540	4.53_341.9599m/z	neg
6541	4.55_253.1611m/z	pos
6542	4.55_298.2246m/z	pos

6543	4.57_448.5647m/z	pos
6544	4.57_448.9000m/z	pos
6545	4.58_125.9870m/z	pos
6546	4.63_318.1920m/z	pos
6547	4.64_340.1749n	pos
6548	4.66_410.5495m/z	pos
6549	4.67_213.1150m/z	pos
6550	4.69_222.1492n	pos
6551	4.69_268.2131m/z	pos
6552	4.70_272.9251m/z	neg
6553	4.70_308.9015m/z	neg
6554	4.70_310.8989m/z	neg
6555	4.70_335.9203m/z	neg
6556	4.70_337.9182m/z	neg
6557	4.70_346.9685m/z	neg
6558	4.70_350.0076n	neg
6559	4.70_362.9400m/z	neg
6560	4.70_364.9406m/z	neg
6561	4.71_263.0122n	pos
6562	4.71_527.0525m/z	pos
6563	4.72_129.0549m/z	neg
6564	4.72_197.0422m/z	neg
6565	4.72_276.0028m/z	neg
6566	4.72_343.0702m/z	neg
6567	4.72_360.0596m/z	neg

6568	4.73_226.0177m/z	pos
6569	4.73_250.0124n	neg
6570	4.73_265.9743m/z	neg
6571	4.73_281.9473m/z	neg
6572	4.73_289.8962m/z	neg
6573	4.73_291.8932m/z	neg
6574	4.73_294.0034n	neg
6575	4.73_308.9659m/z	neg
6576	4.73_325.8599m/z	neg
6577	4.74_248.0953m/z	pos
6578	4.74_253.9993m/z	pos
6579	4.74_416.2863m/z	pos
6580	4.74_460.2127m/z	pos
6581	4.75_231.0228m/z	pos
6582	4.75_327.8568m/z	neg
6583	4.76_348.0805m/z	neg
6584	4.78_384.2026n	pos
6585	4.79_304.1826m/z	pos
6586	4.79_338.8916m/z	neg
6587	4.79_643.3417m/z	pos
6588	4.81_733.3372m/z	pos
6589	4.83_743.3932m/z	pos
6590	4.84_703.9599m/z	pos
6591	4.86_125.9860m/z	pos
6592	4.86_414.2460n	pos

6593	4.86_460.3114m/z	pos
6594	4.86_880.2001m/z	pos
6595	4.87_703.7600m/z	pos
6596	4.89_266.1739n	pos
6597	4.89_312.2396m/z	pos
6598	4.89_704.1607m/z	pos
6599	4.89_879.9486m/z	pos
6600	4.90_377.1349m/z	neg
6601	4.91_304.1326m/z	pos
6602	4.91_428.2295n	pos
6603	4.95_171.1395m/z	pos
6604	4.95_213.1157m/z	pos
6605	4.95_323.9009m/z	neg
6606	4.95_569.3003m/z	pos
6607	4.95_569.8008m/z	pos
6608	4.96_236.0050m/z	pos
6609	4.96_458.2731n	pos
6610	4.96_504.3379m/z	pos
6611	4.96_531.6309m/z	pos
6612	4.98_234.0081m/z	pos
6613	5.00_130.9890m/z	neg
6614	5.01_518.3191m/z	pos
6615	5.03_123.1157m/z	pos
6616	5.03_311.9841m/z	pos
6617	5.03_315.9736m/z	neg

6618	5.03_379.1974m/z	pos
6619	5.04_111.1178m/z	pos
6620	5.04_151.1127m/z	pos
6621	5.04_171.1403m/z	pos
6622	5.04_226.9705m/z	pos
6623	5.04_232.9878m/z	pos
6624	5.04_245.0086n	pos
6625	5.04_310.2011n	pos
6626	5.04_324.9966m/z	pos
6627	5.04_350.9917m/z	pos
6628	5.04_432.0141m/z	pos
6629	5.04_745.2883m/z	pos
6630	5.05_107.0495m/z	neg
6631	5.05_271.9841m/z	neg
6632	5.05_274.9822m/z	neg
6633	5.05_316.9646m/z	neg
6634	5.05_333.9537m/z	neg
6635	5.05_350.9443m/z	neg
6636	5.05_397.0021m/z	neg
6637	5.06_124.8903m/z	neg
6638	5.06_186.4330m/z	neg
6639	5.06_244.9652m/z	neg
6640	5.06_306.9489m/z	neg
6641	5.06_413.9835n	neg
6642	5.06_420.0775m/z	neg

6643	5.06_475.9617m/z	neg
6644	5.07_167.1084m/z	pos
6645	5.07_502.2985n	pos
6646	5.08_141.0912m/z	pos
6647	5.08_237.1725m/z	pos
6648	5.08_246.0758m/z	neg
6649	5.08_282.2286m/z	pos
6650	5.09_207.0105m/z	neg
6651	5.10_452.3391n	pos
6652	5.10_498.4047m/z	pos
6653	5.11_220.0388n	neg
6654	5.11_233.9904m/z	neg
6655	5.11_259.5119m/z	neg
6656	5.11_260.0176n	neg
6657	5.11_267.1785m/z	pos
6658	5.11_312.2354m/z	pos
6659	5.11_438.0610m/z	neg
6660	5.12_188.0036m/z	pos
6661	5.12_189.9998m/z	pos
6662	5.12_203.0044m/z	pos
6663	5.12_210.0554n	pos
6664	5.12_231.9928m/z	neg
6665	5.12_234.0095m/z	pos
6666	5.12_236.0068m/z	pos
6667	5.12_264.9752n	pos

6668	5.12_266.9743n	pos
6669	5.12_306.0084m/z	pos
6670	5.12_312.0058m/z	pos
6671	5.12_355.9916m/z	neg
6672	5.12_369.8858m/z	neg
6673	5.12_372.0656m/z	pos
6674	5.12_564.0844m/z	neg
6675	5.12_786.2569m/z	pos
6676	5.14_125.9861m/z	pos
6677	5.14_277.0317m/z	neg
6678	5.14_282.9897m/z	neg
6679	5.14_294.0223m/z	neg
6680	5.14_361.9381m/z	neg
6681	5.14_371.8831m/z	neg
6682	5.14_373.8803m/z	neg
6683	5.16_546.3254n	pos
6684	5.17_377.1338m/z	neg
6685	5.18_151.0268m/z	pos
6686	5.18_402.2722m/z	pos
6687	5.18_592.3900m/z	pos
6688	5.19_344.2075m/z	pos
6689	5.20_230.9939m/z	neg
6690	5.20_266.5198m/z	neg
6691	5.20_267.0259n	neg
6692	5.20_330.1930m/z	pos

6693	5.20_437.0538m/z	neg
6694	5.20_555.0245m/z	neg
6695	5.21_218.5282m/z	neg
6696	5.21_578.1028m/z	neg
6697	5.22_168.9890m/z	neg
6698	5.22_217.0179m/z	pos
6699	5.22_493.0538m/z	pos
6700	5.23_240.9851m/z	neg
6701	5.23_308.9016m/z	neg
6702	5.23_310.8987m/z	neg
6703	5.23_332.0053n	neg
6704	5.23_335.9205m/z	neg
6705	5.23_337.9177m/z	neg
6706	5.23_346.9687m/z	neg
6707	5.23_362.9397m/z	neg
6708	5.23_364.9412m/z	neg
6709	5.24_125.0225m/z	pos
6710	5.24_132.5126m/z	pos
6711	5.24_241.0305n	pos
6712	5.24_247.0328n	pos
6713	5.24_280.1899n	pos
6714	5.24_393.0395m/z	pos
6715	5.24_421.9942m/z	pos
6716	5.25_196.9843m/z	neg
6717	5.25_372.0655m/z	pos

6718	5.25_590.3512n	pos
6719	5.25_636.4163m/z	pos
6720	5.27_224.5642m/z	neg
6721	5.27_225.0700n	neg
6722	5.27_351.1622m/z	neg
6723	5.27_471.1119m/z	neg
6724	5.27_615.1028m/z	neg
6725	5.28_203.0018m/z	pos
6726	5.28_226.8233m/z	neg
6727	5.28_255.2147m/z	pos
6728	5.28_353.1803m/z	pos
6729	5.28_554.1896m/z	pos
6730	5.28_566.4285m/z	pos
6731	5.28_616.1144n	pos
6732	5.29_304.2503m/z	pos
6733	5.30_226.9947m/z	neg
6734	5.31_194.1168m/z	pos
6735	5.31_260.1891m/z	pos
6736	5.31_317.1900n	pos
6737	5.31_326.7109m/z	pos
6738	5.31_479.5849m/z	pos
6739	5.31_479.9201m/z	pos
6740	5.31_631.0158m/z	pos
6741	5.31_631.3516m/z	pos
6742	5.31_631.6850m/z	pos

6743	5.31_634.3773n	pos
6744	5.31_718.8737m/z	pos
6745	5.31_719.3759m/z	pos
6746	5.31_946.5184m/z	pos
6747	5.33_142.9921m/z	neg
6748	5.33_413.9119m/z	neg
6749	5.33_415.9088m/z	neg
6750	5.34_210.0540n	pos
6751	5.34_304.2173m/z	pos
6752	5.36_158.0026m/z	pos
6753	5.36_385.1677m/z	neg
6754	5.36_630.1346n	pos
6755	5.37_267.0012m/z	pos
6756	5.37_272.1893m/z	pos
6757	5.37_289.2251m/z	pos
6758	5.37_301.1044n	pos
6759	5.37_339.2029n	pos
6760	5.37_348.7236m/z	pos
6761	5.37_490.3240m/z	pos
6762	5.37_568.2058m/z	pos
6763	5.37_678.4035n	pos
6764	5.38_333.0053m/z	neg
6765	5.40_284.1903m/z	pos
6766	5.41_238.5796m/z	neg
6767	5.41_239.0857n	neg

6768	5.41_362.0451m/z	neg
6769	5.41_415.9273m/z	neg
6770	5.43_179.0719m/z	pos
6771	5.43_193.0873m/z	pos
6772	5.43_230.0828m/z	pos
6773	5.43_278.0755n	neg
6774	5.43_283.2456m/z	pos
6775	5.43_334.0397m/z	pos
6776	5.43_340.0629m/z	neg
6777	5.43_355.0102m/z	neg
6778	5.43_356.0294m/z	neg
6779	5.43_381.2135m/z	pos
6780	5.43_441.0057m/z	neg
6781	5.44_238.3625m/z	pos
6782	5.44_698.7883m/z	pos
6783	5.44_699.2911m/z	pos
6784	5.46_224.1678m/z	pos
6785	5.46_361.2163n	pos
6786	5.46_370.7369m/z	pos
6787	5.46_722.4294n	pos
6788	5.48_224.5639m/z	neg
6789	5.48_225.0697n	neg
6790	5.50_125.9859m/z	pos
6791	5.50_289.0413m/z	neg
6792	5.51_239.5873m/z	neg

6793	5.51_240.0853m/z	neg
6794	5.51_255.2134m/z	pos
6795	5.51_282.9915m/z	neg
6796	5.51_285.2649m/z	pos
6797	5.53_242.1796m/z	pos
6798	5.53_292.9883m/z	neg
6799	5.53_375.1184m/z	neg
6800	5.53_383.2291n	pos
6801	5.54_504.2080m/z	pos
6802	5.56_432.8869m/z	pos
6803	5.57_383.1522m/z	neg
6804	5.58_274.1310n	neg
6805	5.60_405.2419n	pos
6806	5.61_186.1471m/z	pos
6807	5.61_344.2094m/z	pos
6808	5.61_359.1233m/z	neg
6809	5.62_245.5691m/z	neg
6810	5.65_601.2685m/z	pos
6811	5.66_294.2059n	pos
6812	5.66_600.2830m/z	neg
6813	5.67_234.9843m/z	neg
6814	5.69_198.1288m/z	pos
6815	5.69_274.2033m/z	pos
6816	5.69_427.2550n	pos
6817	5.69_436.7753m/z	pos

6818	5.70_543.2821m/z	neg
6819	5.71_375.1185m/z	neg
6820	5.72_125.9861m/z	pos
6821	5.72_179.1073m/z	pos
6822	5.73_285.9989m/z	neg
6823	5.74_358.0490m/z	neg
6824	5.74_360.0656m/z	pos
6825	5.76_312.1966n	pos
6826	5.76_449.2684n	pos
6827	5.78_267.0015m/z	pos
6828	5.79_301.0995n	pos
6829	5.80_338.2320n	pos
6830	5.80_341.1125m/z	pos
6831	5.83_242.9899m/z	neg
6832	5.83_266.1552n	pos
6833	5.83_328.2160m/z	pos
6834	5.83_384.2992m/z	pos
6835	5.84_348.2324n	pos
6836	5.84_471.2812n	pos
6837	5.85_541.2657m/z	neg
6838	5.86_219.1393m/z	pos
6839	5.89_343.0842m/z	neg
6840	5.90_361.1461m/z	pos
6841	5.90_511.3301m/z	pos
6842	5.91_413.1979m/z	neg

6843	5.95_576.3628m/z	pos
6844	5.96_539.2489m/z	neg
6845	5.97_228.1626m/z	pos
6846	5.99_300.1182n	pos
6847	5.99_367.1604m/z	pos
6848	6.00_218.2128m/z	pos
6849	6.01_176.0730m/z	pos
6850	6.01_227.0913m/z	neg
6851	6.01_312.2145n	pos
6852	6.01_348.2321n	pos
6853	6.01_348.9927m/z	pos
6854	6.01_541.2649m/z	neg
6855	6.02_130.0655m/z	neg
6856	6.04_137.1321m/z	pos
6857	6.04_165.1131n	pos
6858	6.04_217.0417m/z	neg
6859	6.04_271.1866m/z	pos
6860	6.05_394.1241m/z	neg
6861	6.06_186.0498m/z	pos
6862	6.06_213.1147m/z	pos
6863	6.06_256.1144m/z	pos
6864	6.06_416.1059m/z	neg
6865	6.07_315.1293m/z	neg
6866	6.08_194.9897m/z	neg
6867	6.08_237.1500m/z	pos

6868	6.08_261.8884m/z	neg
6869	6.08_376.6284m/z	pos
6870	6.11_197.1425n	pos
6871	6.11_234.1352m/z	pos
6872	6.12_318.2673m/z	pos
6873	6.13_262.2397m/z	pos
6874	6.13_267.1269m/z	pos
6875	6.13_283.1170m/z	neg
6876	6.14_208.1675m/z	pos
6877	6.14_361.1466m/z	pos
6878	6.15_111.0432m/z	pos
6879	6.15_197.1534m/z	pos
6880	6.16_310.9827m/z	neg
6881	6.16_515.2855m/z	neg
6882	6.17_234.2083m/z	pos
6883	6.17_363.2195m/z	pos
6884	6.17_681.2170m/z	pos
6885	6.18_133.0650m/z	neg
6886	6.18_213.0215m/z	neg
6887	6.18_290.1132m/z	pos
6888	6.18_331.2102m/z	pos
6889	6.18_348.9922m/z	pos
6890	6.19_252.2210m/z	pos
6891	6.19_263.6279n	neg
6892	6.19_412.2886m/z	pos

6893	6.22_514.1743m/z	pos
6894	6.23_186.0499m/z	pos
6895	6.23_226.1819m/z	pos
6896	6.23_266.1775m/z	pos
6897	6.23_300.1135n	pos
6898	6.23_332.1378n	pos
6899	6.23_633.2559m/z	pos
6900	6.24_331.1280m/z	neg
6901	6.24_416.1060m/z	neg
6902	6.26_141.0907m/z	pos
6903	6.26_365.2343m/z	pos
6904	6.27_202.1817m/z	pos
6905	6.31_286.2060m/z	pos
6906	6.33_197.1529m/z	pos
6907	6.33_234.1346m/z	pos
6908	6.33_510.2563m/z	pos
6909	6.34_412.2903m/z	pos
6910	6.35_289.6268n	neg
6911	6.35_394.1245m/z	neg
6912	6.36_167.0266m/z	pos
6913	6.36_248.0339n	pos
6914	6.36_268.9853m/z	neg
6915	6.36_310.2047m/z	pos
6916	6.37_130.0078m/z	pos
6917	6.37_141.0100m/z	pos

6918	6.37_141.5147m/z	pos
6919	6.37_303.9914m/z	pos
6920	6.37_305.9887m/z	pos
6921	6.38_325.8961m/z	neg
6922	6.38_327.8931m/z	neg
6923	6.39_125.9860m/z	pos
6924	6.39_348.9912m/z	pos
6925	6.39_533.2420m/z	pos
6926	6.39_541.2651m/z	neg
6927	6.41_279.6142m/z	neg
6928	6.43_296.2458m/z	pos
6929	6.43_525.2692m/z	neg
6930	6.43_589.3033m/z	neg
6931	6.45_539.2492m/z	neg
6932	6.47_288.6197m/z	neg
6933	6.47_331.0573m/z	neg
6934	6.47_413.1978m/z	neg
6935	6.49_234.1351m/z	pos
6936	6.50_186.1471m/z	pos
6937	6.51_197.1214m/z	pos
6938	6.51_432.3136m/z	pos
6939	6.52_151.1132m/z	pos
6940	6.52_475.1693m/z	pos
6941	6.52_593.3356m/z	pos
6942	6.53_272.2174n	pos

6943	6.53_311.2273m/z	pos
6944	6.55_143.0898m/z	pos
6945	6.55_184.1163m/z	pos
6946	6.55_261.0452m/z	pos
6947	6.55_304.2491m/z	pos
6948	6.56_272.0907m/z	pos
6949	6.57_284.9561n	neg
6950	6.57_319.9253m/z	neg
6951	6.57_321.9198m/z	neg
6952	6.57_323.9192m/z	neg
6953	6.60_104.0058m/z	pos
6954	6.60_132.0045m/z	pos
6955	6.60_132.5020m/z	pos
6956	6.60_209.0567n	pos
6957	6.60_267.0016m/z	pos
6958	6.60_292.9067m/z	neg
6959	6.60_314.9962m/z	neg
6960	6.60_346.9458m/z	neg
6961	6.61_430.2979m/z	pos
6962	6.62_213.1148m/z	pos
6963	6.62_298.2340n	pos
6964	6.62_413.1993m/z	neg
6965	6.62_498.1761m/z	neg
6966	6.62_622.3229m/z	neg
6967	6.63_272.2172n	pos

6968	6.63_379.1970m/z	pos
6969	6.64_310.2047m/z	pos
6970	6.65_265.1885m/z	pos
6971	6.66_142.9922m/z	neg
6972	6.66_332.2505m/z	pos
6973	6.66_475.1689m/z	pos
6974	6.68_236.9803m/z	neg
6975	6.69_125.9865m/z	pos
6976	6.69_234.1375m/z	pos
6977	6.69_288.2208m/z	pos
6978	6.69_313.2270m/z	pos
6979	6.69_331.1277m/z	neg
6980	6.69_464.3002m/z	neg
6981	6.69_481.2417m/z	neg
6982	6.70_241.1712n	pos
6983	6.70_681.2767m/z	pos
6984	6.73_260.0525m/z	pos
6985	6.73_300.2189m/z	pos
6986	6.73_318.0285m/z	neg
6987	6.73_348.9120m/z	neg
6988	6.73_400.2919m/z	pos
6989	6.73_496.2074m/z	pos
6990	6.74_378.1486n	pos
6991	6.74_431.3055n	pos
6992	6.74_626.3548m/z	pos

6993	6.75_141.0906m/z	pos
6994	6.75_186.9976m/z	neg
6995	6.75_350.1756n	neg
6996	6.75_624.3391m/z	neg
6997	6.80_294.2445m/z	pos
6998	6.80_397.1704m/z	neg
6999	6.81_528.2249n	pos
7000	6.84_147.1168m/z	pos
7001	6.84_245.2223m/z	pos
7002	6.84_326.2028m/z	pos
7003	6.86_125.9858m/z	pos
7004	6.86_317.6344m/z	pos
7005	6.86_348.9919m/z	pos
7006	6.86_633.2567m/z	pos
7007	6.89_502.0474m/z	pos
7008	6.89_640.3346m/z	neg
7009	6.91_336.2189m/z	pos
7010	6.92_235.1012m/z	pos
7011	6.92_308.9967m/z	neg
7012	6.93_119.0498m/z	pos
7013	6.93_168.9898m/z	neg
7014	6.94_234.1353m/z	pos
7015	6.94_412.2902m/z	pos
7016	6.95_348.9914m/z	pos
7017	6.98_397.2037m/z	neg

7018	6.98_633.2606m/z	pos
7019	6.99_223.1707m/z	pos
7020	6.99_463.2757n	pos
7021	7.00_294.9833m/z	neg
7022	7.00_415.1944m/z	neg
7023	7.00_442.1630m/z	pos
7024	7.00_674.3217m/z	neg
7025	7.01_213.1156m/z	pos
7026	7.01_489.2030n	pos
7027	7.02_430.2985m/z	pos
7028	7.03_253.1474m/z	pos
7029	7.03_412.2886m/z	pos
7030	7.03_465.3135n	pos
7031	7.04_302.2339m/z	pos
7032	7.04_356.2499m/z	pos
7033	7.05_178.1011m/z	pos
7034	7.05_239.1894n	pos
7035	7.06_216.4461m/z	pos
7036	7.06_324.2422n	pos
7037	7.06_412.2137m/z	neg
7038	7.08_375.1536m/z	pos
7039	7.09_312.2216m/z	pos
7040	7.09_371.1885m/z	neg
7041	7.09_479.2717n	pos
7042	7.10_167.0744m/z	pos

7043	7.10_234.1347m/z	pos
7044	7.10_345.1445m/z	neg
7045	7.12_186.9979m/z	neg
7046	7.12_191.0849m/z	pos
7047	7.12_281.1242m/z	neg
7048	7.12_300.9902m/z	neg
7049	7.12_317.6346m/z	pos
7050	7.12_496.2078m/z	neg
7051	7.12_497.2235n	pos
7052	7.13_367.2700m/z	pos
7053	7.14_281.6298n	neg
7054	7.14_369.9070m/z	neg
7055	7.14_371.9042m/z	neg
7056	7.14_528.2625m/z	neg
7057	7.14_584.2327m/z	neg
7058	7.18_195.1054m/z	pos
7059	7.18_254.1183n	pos
7060	7.18_581.2978m/z	pos
7061	7.19_176.9970m/z	neg
7062	7.19_302.2352m/z	pos
7063	7.19_355.2669m/z	pos
7064	7.23_208.9830m/z	neg
7065	7.23_312.2194m/z	pos
7066	7.23_344.2099m/z	pos
7067	7.24_352.9928m/z	neg

7068	7.24_371.1883m/z	neg
7069	7.25_297.1259m/z	neg
7070	7.26_316.1439n	pos
7071	7.27_624.3443m/z	pos
7072	7.28_409.2756m/z	pos
7073	7.28_605.3339m/z	pos
7074	7.29_357.2810m/z	pos
7075	7.30_341.0015m/z	neg
7076	7.31_313.1183m/z	neg
7077	7.31_315.1368m/z	pos
7078	7.31_348.9906m/z	pos
7079	7.32_197.1357m/z	pos
7080	7.32_241.8980m/z	neg
7081	7.32_265.0714m/z	pos
7082	7.32_366.0221m/z	neg
7083	7.32_378.1006m/z	neg
7084	7.32_425.1146m/z	neg
7085	7.32_427.1128m/z	neg
7086	7.32_469.1153n	neg
7087	7.32_503.1303m/z	neg
7088	7.32_532.1044n	neg
7089	7.34_270.2016n	pos
7090	7.34_473.1989m/z	pos
7091	7.34_527.1909n	pos
7092	7.34_537.1112m/z	neg

7093	7.34_624.2389m/z	pos
7094	7.34_683.2558m/z	pos
7095	7.35_179.1078m/z	pos
7096	7.38_213.1156m/z	pos
7097	7.41_137.1322m/z	pos
7098	7.43_409.2750m/z	pos
7099	7.43_649.2532m/z	pos
7100	7.44_402.2877m/z	pos
7101	7.44_437.0533m/z	neg
7102	7.44_522.0301m/z	neg
7103	7.44_583.3115m/z	neg
7104	7.45_301.0403m/z	pos
7105	7.47_326.9883m/z	neg
7106	7.49_346.1549n	pos
7107	7.50_253.1475m/z	pos
7108	7.50_303.6679n	neg
7109	7.50_431.3078n	pos
7110	7.51_111.0467m/z	pos
7111	7.51_302.2353m/z	pos
7112	7.53_342.1731m/z	pos
7113	7.54_211.0133n	pos
7114	7.54_346.2608m/z	pos
7115	7.56_272.2193n	pos
7116	7.56_370.1853n	pos
7117	7.56_454.1501m/z	neg

7118	7.57_222.99 94m/z	neg
7119	7.59_181.16 04m/z	pos
7120	7.59_190.13 71n	pos
7121	7.59_243.18 50n	pos
7122	7.59_300.13 32m/z	neg
7123	7.59_326.12 85m/z	neg
7124	7.59_327.15 22m/z	neg
7125	7.59_384.30 06m/z	pos
7126	7.60_447.30 23n	pos
7127	7.60_466.31 99m/z	pos
7128	7.61_244.90 74m/z	neg
7129	7.61_246.90 43m/z	neg
7130	7.61_248.90 16m/z	neg
7131	7.61_292.98 56m/z	neg
7132	7.61_445.11 15m/z	neg
7133	7.62_313.11 84m/z	neg
7134	7.63_288.05 69n	pos
7135	7.65_223.17 05m/z	pos
7136	7.65_246.24 45m/z	pos
7137	7.65_257.23 85m/z	pos
7138	7.65_264.63 60n	neg
7139	7.65_314.23 61m/z	pos
7140	7.65_431.30 62n	pos
7141	7.65_529.27 51n	pos
7142	7.66_300.24 82n	pos

7143	7.66_381.2133m/z	pos
7144	7.67_357.0595m/z	neg
7145	7.68_338.2356m/z	pos
7146	7.68_777.6981m/z	pos
7147	7.69_775.6839m/z	neg
7148	7.70_151.0765m/z	pos
7149	7.70_442.1626m/z	pos
7150	7.72_234.1357m/z	pos
7151	7.72_286.9813m/z	neg
7152	7.72_358.2629m/z	pos
7153	7.72_371.1884m/z	neg
7154	7.74_599.2551m/z	pos
7155	7.75_190.9932m/z	neg
7156	7.76_145.0070m/z	neg
7157	7.76_372.2689n	pos
7158	7.76_584.3457m/z	pos
7159	7.76_649.2548m/z	pos
7160	7.77_181.1571m/z	pos
7161	7.78_161.1330m/z	pos
7162	7.78_402.0975n	pos
7163	7.78_469.0716m/z	neg
7164	7.80_232.1817m/z	pos
7165	7.80_338.2350m/z	pos
7166	7.81_191.1121m/z	pos
7167	7.81_222.9992m/z	neg

7168	7.81_640.3713m/z	pos
7169	7.82_155.1075m/z	pos
7170	7.82_353.2352m/z	pos
7171	7.82_358.2626m/z	pos
7172	7.82_414.3047m/z	pos
7173	7.82_431.3078n	pos
7174	7.82_529.2754n	pos
7175	7.82_550.2448m/z	neg
7176	7.83_262.2392m/z	pos
7177	7.84_412.2883m/z	pos
7178	7.86_255.6306n	neg
7179	7.86_402.3166n	pos
7180	7.86_436.2944m/z	pos
7181	7.86_511.2664n	pos
7182	7.86_532.2332m/z	neg
7183	7.86_578.3514n	pos
7184	7.86_614.3702n	pos
7185	7.87_230.2128m/z	pos
7186	7.87_308.9951m/z	neg
7187	7.88_368.0307m/z	neg
7188	7.88_370.1848n	pos
7189	7.88_430.8929m/z	pos
7190	7.88_475.2151m/z	pos
7191	7.88_508.1957n	pos
7192	7.88_530.1972m/z	pos

7193	7.88_533.1103m/z	neg
7194	7.88_610.3599m/z	neg
7195	7.90_443.1537m/z	neg
7196	7.91_285.2637m/z	pos
7197	7.91_314.2376m/z	pos
7198	7.91_430.3003m/z	pos
7199	7.91_446.1006n	pos
7200	7.91_476.3037m/z	pos
7201	7.92_246.1539m/z	pos
7202	7.92_246.9811m/z	neg
7203	7.92_327.9877m/z	neg
7204	7.94_186.9974m/z	neg
7205	7.94_357.2808m/z	pos
7206	7.94_625.3546n	pos
7207	7.94_640.3719m/z	pos
7208	7.96_321.1460m/z	neg
7209	7.96_340.0340m/z	neg
7210	7.96_356.0089m/z	neg
7211	7.96_359.9960m/z	neg
7212	7.96_363.9563m/z	neg
7213	7.96_365.9538m/z	neg
7214	7.96_386.0061m/z	neg
7215	7.96_397.9937m/z	neg
7216	7.96_399.9329m/z	neg
7217	7.96_401.9302m/z	neg

7218	7.96_408.0184m/z	neg
7219	7.96_425.0035m/z	neg
7220	7.96_501.1711m/z	neg
7221	7.97_139.0408m/z	pos
7222	7.97_153.0556m/z	pos
7223	7.97_195.1042m/z	pos
7224	7.97_238.3569m/z	neg
7225	7.97_240.1012n	pos
7226	7.97_318.9854m/z	neg
7227	7.97_329.0602m/z	neg
7228	7.97_346.0506m/z	neg
7229	7.98_187.1275m/z	pos
7230	8.00_214.9933m/z	neg
7231	8.00_280.9834m/z	neg
7232	8.02_477.2901m/z	pos
7233	8.03_269.2291m/z	pos
7234	8.03_641.3534m/z	neg
7235	8.04_417.2117m/z	neg
7236	8.06_451.3285m/z	pos
7237	8.06_642.3787n	pos
7238	8.07_111.0455m/z	pos
7239	8.07_125.9860m/z	pos
7240	8.08_284.2235m/z	pos
7241	8.08_625.3517n	pos
7242	8.08_627.3733m/z	neg

7243	8.12_252.6364m/z	pos
7244	8.12_663.2666m/z	pos
7245	8.13_264.9910m/z	neg
7246	8.13_510.2508m/z	neg
7247	8.14_253.1472m/z	pos
7248	8.14_350.9908m/z	pos
7249	8.14_464.2849m/z	pos
7250	8.14_529.2114m/z	pos
7251	8.14_532.2869m/z	neg
7252	8.14_544.2698m/z	pos
7253	8.14_549.2767m/z	neg
7254	8.14_665.2494m/z	pos
7255	8.15_226.6276m/z	pos
7256	8.15_235.6348m/z	pos
7257	8.15_414.3024m/z	pos
7258	8.15_449.3159n	pos
7259	8.15_568.3518m/z	pos
7260	8.16_663.2299m/z	neg
7261	8.17_234.1349m/z	pos
7262	8.17_511.3007m/z	neg
7263	8.17_516.2928m/z	neg
7264	8.18_633.2583m/z	pos
7265	8.19_130.9912m/z	neg
7266	8.19_327.9880m/z	neg
7267	8.19_337.2061m/z	pos

7268	8.21_321.14 61m/z	neg
7269	8.22_175.12 54m/z	pos
7270	8.24_360.28 12m/z	pos
7271	8.24_465.24 69m/z	neg
7272	8.24_484.29 36m/z	pos
7273	8.26_268.98 66m/z	neg
7274	8.26_601.26 87m/z	pos
7275	8.28_174.99 26m/z	pos
7276	8.28_328.25 09m/z	pos
7277	8.28_373.27 63m/z	pos
7278	8.30_255.21 52m/z	pos
7279	8.30_296.99 62m/z	neg
7280	8.30_633.25 94m/z	pos
7281	8.31_340.25 21m/z	pos
7282	8.32_389.98 64m/z	neg
7283	8.32_460.29 00m/z	neg
7284	8.32_679.26 17m/z	neg
7285	8.32_681.28 02m/z	pos
7286	8.33_234.13 52m/z	pos
7287	8.33_268.27 08m/z	pos
7288	8.33_360.28 01m/z	pos
7289	8.35_316.25 28m/z	pos
7290	8.37_321.14 62m/z	neg
7291	8.38_272.21 91n	pos
7292	8.38_285.26 45m/z	pos

7293	8.38_368.1924m/z	pos
7294	8.39_512.3402m/z	pos
7295	8.40_376.9869m/z	neg
7296	8.40_627.3732m/z	neg
7297	8.41_161.1329m/z	pos
7298	8.41_381.3183m/z	pos
7299	8.43_481.2892n	pos
7300	8.43_498.2871m/z	neg
7301	8.43_500.3078m/z	pos
7302	8.43_624.2392m/z	pos
7303	8.44_518.3328m/z	pos
7304	8.46_628.3778m/z	neg
7305	8.47_191.1082m/z	pos
7306	8.47_234.1356m/z	pos
7307	8.48_125.9859m/z	pos
7308	8.48_287.6118m/z	neg
7309	8.48_384.2853m/z	pos
7310	8.48_497.2729m/z	neg
7311	8.50_174.9951m/z	pos
7312	8.50_181.1234m/z	pos
7313	8.50_278.9879m/z	neg
7314	8.50_663.2672m/z	pos
7315	8.51_629.2242m/z	neg
7316	8.52_255.2154m/z	pos
7317	8.54_681.2807m/z	pos

7318	8.56_242.2869m/z	pos
7319	8.59_232.0783m/z	pos
7320	8.59_280.2705m/z	pos
7321	8.59_324.9896m/z	neg
7322	8.61_509.2735m/z	neg
7323	8.61_627.3724m/z	neg
7324	8.62_202.1802m/z	pos
7325	8.62_314.1454m/z	pos
7326	8.62_331.2502m/z	pos
7327	8.62_512.2672m/z	neg
7328	8.62_549.2037m/z	pos
7329	8.63_213.1160m/z	pos
7330	8.63_295.2291m/z	pos
7331	8.63_340.2527m/z	pos
7332	8.64_329.2322m/z	neg
7333	8.65_125.9872m/z	pos
7334	8.65_276.2007m/z	pos
7335	8.65_494.3330m/z	pos
7336	8.65_556.3253m/z	neg
7337	8.66_426.9756m/z	neg
7338	8.67_137.0977m/z	pos
7339	8.67_235.0991m/z	pos
7340	8.69_143.1051m/z	pos
7341	8.69_387.1828m/z	pos
7342	8.70_111.0819m/z	pos

7343	8.71_135.9051m/z	pos
7344	8.71_141.0910m/z	pos
7345	8.71_243.2330m/z	pos
7346	8.73_133.9050m/z	pos
7347	8.73_210.1635n	pos
7348	8.73_210.5269m/z	pos
7349	8.73_234.2052m/z	pos
7350	8.73_273.2558m/z	pos
7351	8.73_312.1507m/z	pos
7352	8.73_344.2916m/z	pos
7353	8.74_133.5762m/z	pos
7354	8.74_214.1814m/z	pos
7355	8.74_266.1560m/z	pos
7356	8.74_326.9888m/z	neg
7357	8.75_290.1962m/z	neg
7358	8.75_294.6197m/z	neg
7359	8.76_181.1246m/z	pos
7360	8.77_384.2783m/z	pos
7361	8.77_466.2551m/z	neg
7362	8.77_601.2728m/z	pos
7363	8.78_317.9512m/z	neg
7364	8.78_460.2900m/z	neg
7365	8.78_556.3296m/z	neg
7366	8.79_125.9860m/z	pos
7367	8.79_462.3152m/z	pos

7368	8.80_341.2870m/z	pos
7369	8.80_415.3137n	pos
7370	8.80_633.2586m/z	pos
7371	8.81_256.6385n	neg
7372	8.81_282.1693n	pos
7373	8.81_534.2497m/z	neg
7374	8.81_552.2335m/z	pos
7375	8.81_593.2659m/z	pos
7376	8.83_330.2664m/z	pos
7377	8.85_430.3195m/z	pos
7378	8.87_285.2632m/z	pos
7379	8.89_294.2433m/z	pos
7380	8.89_329.2488m/z	pos
7381	8.91_158.1547m/z	pos
7382	8.91_345.9811m/z	neg
7383	8.91_353.2431m/z	pos
7384	8.91_609.3273m/z	neg
7385	8.92_282.9840m/z	neg
7386	8.92_408.2950m/z	neg
7387	8.93_318.3042m/z	pos
7388	8.93_454.3218m/z	pos
7389	8.93_628.3748m/z	pos
7390	8.94_616.2688n	pos
7391	8.95_237.1517m/z	pos
7392	8.95_362.3295m/z	pos

7393	8.95_447.3024n	pos
7394	8.95_585.2741m/z	pos
7395	8.97_228.1041m/z	pos
7396	8.97_386.1762n	pos
7397	8.97_432.2414m/z	pos
7398	8.98_208.9829m/z	neg
7399	8.98_242.2174n	pos
7400	8.98_256.9854m/z	neg
7401	8.98_258.9816m/z	neg
7402	8.99_373.2783m/z	pos
7403	9.01_207.0336m/z	pos
7404	9.01_223.0272m/z	neg
7405	9.01_386.2978m/z	pos
7406	9.02_227.5005m/z	pos
7407	9.02_263.0681m/z	pos
7408	9.02_409.2751m/z	pos
7409	9.03_295.2288m/z	pos
7410	9.03_313.1186m/z	neg
7411	9.03_313.2401m/z	pos
7412	9.03_321.1149n	pos
7413	9.03_329.2326m/z	neg
7414	9.03_480.0700m/z	pos
7415	9.05_265.1431m/z	neg
7416	9.05_600.2618n	pos
7417	9.06_230.9862m/z	neg

7418	9.06_315.2564m/z	pos
7419	9.07_414.3160n	pos
7420	9.07_474.3134m/z	pos
7421	9.07_626.3720n	pos
7422	9.09_151.1138m/z	pos
7423	9.09_330.9835m/z	neg
7424	9.10_111.0432m/z	pos
7425	9.10_366.2676m/z	pos
7426	9.12_135.9054m/z	pos
7427	9.12_246.0823m/z	pos
7428	9.12_261.0458m/z	pos
7429	9.12_287.2255m/z	pos
7430	9.12_434.3321m/z	pos
7431	9.12_610.3658m/z	pos
7432	9.14_111.0838m/z	pos
7433	9.14_234.1362m/z	pos
7434	9.14_254.1535n	pos
7435	9.14_300.2187m/z	pos
7436	9.14_342.2681m/z	pos
7437	9.16_161.1330m/z	pos
7438	9.16_330.2675m/z	pos
7439	9.16_350.9877m/z	pos
7440	9.18_233.1843m/z	pos
7441	9.20_111.0463m/z	pos
7442	9.20_255.8214m/z	neg

7443	9.20_316.1400n	pos
7444	9.20_317.6352m/z	pos
7445	9.20_469.1095m/z	pos
7446	9.20_515.2284m/z	pos
7447	9.20_631.3021m/z	pos
7448	9.20_632.2514n	pos
7449	9.20_653.2212m/z	neg
7450	9.21_367.2917m/z	pos
7451	9.21_386.2960m/z	pos
7452	9.22_288.2484m/z	pos
7453	9.22_296.9831m/z	neg
7454	9.22_351.2201n	pos
7455	9.22_372.2697n	pos
7456	9.22_399.3520m/z	pos
7457	9.23_318.9856m/z	neg
7458	9.24_223.0932n	pos
7459	9.24_271.2162n	pos
7460	9.26_236.9813m/z	neg
7461	9.27_151.1142m/z	pos
7462	9.27_191.1081m/z	pos
7463	9.27_316.2873m/z	pos
7464	9.27_529.2116m/z	pos
7465	9.27_664.2417n	pos
7466	9.29_231.9108m/z	pos
7467	9.30_263.1905n	pos

7468	9.30_366.2684m/z	pos
7469	9.30_479.1935m/z	pos
7470	9.30_479.5298m/z	pos
7471	9.30_718.2910m/z	pos
7472	9.30_718.7874m/z	pos
7473	9.31_625.3583m/z	neg
7474	9.32_624.2398m/z	pos
7475	9.33_336.2517m/z	pos
7476	9.33_338.2596n	pos
7477	9.33_342.2702m/z	pos
7478	9.34_142.0885m/z	pos
7479	9.34_314.9881m/z	neg
7480	9.35_315.1360m/z	neg
7481	9.36_346.2441m/z	pos
7482	9.37_527.2916m/z	pos
7483	9.38_516.2375n	pos
7484	9.39_273.2693n	pos
7485	9.39_307.2316m/z	pos
7486	9.40_288.2489m/z	pos
7487	9.40_386.2920m/z	pos
7488	9.40_620.3430m/z	neg
7489	9.42_125.9859m/z	pos
7490	9.42_246.1893m/z	pos
7491	9.42_264.1987m/z	pos
7492	9.42_362.3284m/z	pos

7493	9.42_473.33 85n	pos
7494	9.43_189.09 22m/z	pos
7495	9.44_330.17 02n	pos
7496	9.45_286.98 44m/z	neg
7497	9.46_195.17 60m/z	pos
7498	9.46_223.17 11m/z	pos
7499	9.46_240.19 84m/z	pos
7500	9.46_253.14 73m/z	pos
7501	9.46_286.14 50n	pos
7502	9.46_332.20 71m/z	pos
7503	9.46_578.41 56m/z	pos
7504	9.47_280.12 48m/z	neg
7505	9.47_367.34 20m/z	pos
7506	9.47_444.29 09n	pos
7507	9.47_498.34 50m/z	pos
7508	9.47_581.23 96m/z	neg
7509	9.47_582.25 02n	pos
7510	9.47_583.76 02m/z	pos
7511	9.48_290.27 09m/z	pos
7512	9.48_548.21 65m/z	pos
7513	9.50_430.89 41m/z	pos
7514	9.51_235.63 49m/z	pos
7515	9.51_243.63 22n	pos
7516	9.51_338.26 08n	pos
7517	9.51_413.02 89m/z	pos

7518	9.51_414.3033m/z	pos
7519	9.51_440.3427m/z	pos
7520	9.51_468.2905n	pos
7521	9.51_471.2983n	pos
7522	9.51_516.2928m/z	neg
7523	9.51_533.2831m/z	neg
7524	9.51_546.2737m/z	neg
7525	9.51_550.2607n	pos
7526	9.51_593.3372m/z	pos
7527	9.51_635.2646m/z	pos
7528	9.51_898.6338n	pos
7529	9.52_119.0511m/z	pos
7530	9.52_213.5709m/z	pos
7531	9.52_385.9931m/z	pos
7532	9.52_386.1766n	pos
7533	9.52_437.7301m/z	pos
7534	9.52_447.2275n	pos
7535	9.52_460.2724m/z	pos
7536	9.52_471.1354m/z	pos
7537	9.52_474.2530m/z	pos
7538	9.52_488.1250m/z	pos
7539	9.52_503.2806m/z	pos
7540	9.52_772.3509n	pos
7541	9.52_858.4761m/z	pos
7542	9.53_533.2732n	neg

7543	9.54_165.1284m/z	pos
7544	9.54_284.2233m/z	pos
7545	9.54_432.2455m/z	pos
7546	9.56_182.0466m/z	pos
7547	9.56_323.1304n	pos
7548	9.56_323.1424m/z	pos
7549	9.56_380.3061n	pos
7550	9.56_482.0508m/z	pos
7551	9.56_484.0488m/z	pos
7552	9.56_646.2609n	pos
7553	9.58_171.1469m/z	pos
7554	9.58_202.0600m/z	pos
7555	9.58_374.2847n	pos
7556	9.58_464.2462m/z	pos
7557	9.59_378.9952m/z	neg
7558	9.59_412.3099m/z	pos
7559	9.60_181.1600m/z	pos
7560	9.60_214.1816m/z	pos
7561	9.60_241.1818m/z	pos
7562	9.60_257.2010n	pos
7563	9.60_303.2662m/z	pos
7564	9.60_397.3125m/z	pos
7565	9.61_607.3480m/z	neg
7566	9.62_151.1128m/z	pos
7567	9.62_302.2731m/z	pos

7568	9.63_315.2550m/z	pos
7569	9.64_268.9873m/z	neg
7570	9.64_290.9888m/z	neg
7571	9.64_298.9860m/z	neg
7572	9.65_262.2547m/z	pos
7573	9.65_348.0064n	neg
7574	9.65_358.0103n	neg
7575	9.65_377.2361n	pos
7576	9.65_439.2201m/z	neg
7577	9.65_444.2119m/z	neg
7578	9.65_461.2024m/z	neg
7579	9.65_510.3297m/z	pos
7580	9.66_137.0610m/z	pos
7581	9.66_161.0976m/z	pos
7582	9.66_178.5817m/z	pos
7583	9.66_220.1115n	pos
7584	9.66_262.1471m/z	pos
7585	9.66_292.2712m/z	neg
7586	9.66_294.1858n	pos
7587	9.66_313.2400m/z	pos
7588	9.66_358.1894m/z	pos
7589	9.66_374.9890m/z	neg
7590	9.66_423.1327m/z	neg
7591	9.66_440.1226m/z	neg
7592	9.67_334.2386m/z	pos

	9.67_368.28	
7593	39m/z	pos
	9.68_222.99	
7594	86m/z	neg
	9.69_254.15	
7595	38n	pos
	9.69_388.31	
7596	25m/z	pos
	9.70_153.05	
7597	80m/z	pos
	9.70_219.13	
7598	93m/z	pos
	9.70_268.13	
7599	29n	pos
	9.70_330.08	
7600	10n	pos
	9.70_332.13	
7601	91m/z	pos
	9.70_368.06	
7602	64m/z	neg
	9.70_575.21	
7603	79m/z	pos
	9.72_511.30	
7604	05m/z	neg
	9.72_516.29	
7605	34m/z	neg
	9.73_234.13	
7606	51m/z	pos
	9.73_235.63	
7607	52m/z	pos
	9.73_244.64	
7608	08m/z	pos
	9.73_248.17	
7609	94n	pos
	9.73_344.28	
7610	36m/z	pos
	9.73_414.30	
7611	28m/z	pos
	9.73_449.31	
7612	69n	pos
	9.73_519.31	
7613	66m/z	neg
	9.74_147.08	
7614	70m/z	pos
	9.74_230.24	
7615	91m/z	pos
	9.74_237.11	
7616	51m/z	pos
	9.75_318.22	
7617	30n	pos

7618	9.75_359.2236m/z	pos
7619	9.75_464.3417m/z	pos
7620	9.76_264.9904m/z	neg
7621	9.76_345.9945m/z	neg
7622	9.76_364.2248m/z	neg
7623	9.77_328.2334m/z	pos
7624	9.77_380.3061n	pos
7625	9.78_179.1077m/z	pos
7626	9.78_399.3487m/z	pos
7627	9.78_540.3710m/z	pos
7628	9.79_215.1267m/z	pos
7629	9.79_304.0120n	neg
7630	9.80_458.3518m/z	pos
7631	9.80_485.0588m/z	pos
7632	9.81_334.9853m/z	neg
7633	9.82_442.3407m/z	pos
7634	9.82_447.3139m/z	pos
7635	9.82_603.5384m/z	pos
7636	9.83_252.1716m/z	pos
7637	9.83_430.2951m/z	neg
7638	9.83_482.2925n	pos
7639	9.84_312.2326n	pos
7640	9.85_280.9851m/z	neg
7641	9.85_377.1997m/z	neg
7642	9.86_151.1133m/z	pos

7643	9.86_285.1386n	pos
7644	9.86_426.2516m/z	pos
7645	9.86_524.2317m/z	pos
7646	9.86_583.2599m/z	pos
7647	9.87_406.9920m/z	neg
7648	9.87_414.2082n	pos
7649	9.87_418.2262m/z	pos
7650	9.87_460.2729m/z	pos
7651	9.87_488.3397m/z	pos
7652	9.87_555.3233m/z	pos
7653	9.88_383.3325m/z	pos
7654	9.88_387.1797n	pos
7655	9.88_389.1905m/z	pos
7656	9.88_431.1703m/z	neg
7657	9.88_432.2414m/z	pos
7658	9.89_611.3803m/z	neg
7659	9.90_147.1174m/z	pos
7660	9.90_174.9929m/z	pos
7661	9.90_210.1633n	pos
7662	9.90_230.8973m/z	pos
7663	9.90_242.2131m/z	pos
7664	9.90_251.0017m/z	pos
7665	9.90_297.2571n	pos
7666	9.90_322.9931m/z	neg
7667	9.90_326.0035n	pos

7668	9.90_328.0007n	pos
7669	9.90_329.9974n	pos
7670	9.90_358.1960m/z	pos
7671	9.90_412.9653m/z	pos
7672	9.90_419.0677m/z	pos
7673	9.90_427.9532m/z	pos
7674	9.90_428.2830m/z	pos
7675	9.91_312.9920m/z	neg
7676	9.91_415.0721m/z	pos
7677	9.91_417.0706m/z	pos
7678	9.91_478.1975m/z	neg
7679	9.91_480.2157m/z	pos
7680	9.91_647.2777m/z	pos
7681	9.92_395.2428m/z	neg
7682	9.93_419.2520m/z	pos
7683	9.94_213.1183m/z	pos
7684	9.94_330.2690m/z	pos
7685	9.94_334.2187n	pos
7686	9.94_338.0022n	neg
7687	9.95_330.9879m/z	neg
7688	9.96_386.9905m/z	neg
7689	9.97_191.1442m/z	pos
7690	9.97_651.1624m/z	pos
7691	9.98_252.1720m/z	pos
7692	9.98_265.3050n	pos

7693	9.98_339.2705m/z	pos
7694	9.98_374.2832n	pos
7695	9.99_455.2461m/z	neg
7696	9.99_488.2460m/z	pos
